# Effectiveness of combining psychological prevention interventions with interventions that address the social determinants of mental health in low- and middle-income countries: a systematic review and meta-analysis

**DOI:** 10.1136/bmjment-2025-301573

**Published:** 2025-05-26

**Authors:** Eleonora Prina, Beatrice Bano, Rakesh Singh, Emiliano Albanese, Daniela Trujillo, Maria Cecilia Dedios Sanguineti, Katherine Sorsdahl, Nagendra Luitel, Emily Garman, Marianna Purgato, Corrado Barbui, Mark Jordans, Crick Lund

**Affiliations:** 1 WHO Collaborating Centre for Research and Training in Mental Health and Service Evaluation, Section of Psychiatry, Department of Neuroscience, Biomedicine and Movement Sciences, University of Verona, Verona, Italy; 2 Faculty of Biomedical Sciences, Institute of Public Health, Università della Svizzera italiana, Lugano, Switzerland; 3 Transcultural Psychosocial Organization Nepal (TPO Nepal), Kathmandu, Nepal; 4 Centre for Global Mental Health, Health Service and Population Research Department, Institute of Psychiatry, Psychology and Neuroscience, King’s College London, London, UK; 5 Department of Psychiatry, University of Geneva, Geneva, Switzerland; 6 Innovations for Poverty Action, Bogotà, Colombia; 7 School of Government, Los Andes University, Bogotá, Colombia; 8 Alan J Flisher Centre for Public Mental Health, Department of Psychiatry and Mental Health, University of Cape Town, Cape Town, South Africa; 9 Cochrane Global Mental Health, University of Verona, Verona, Italy

**Keywords:** PSYCHIATRY, Child & adolescent psychiatry, Anxiety disorders, Adult psychiatry, Depression

## Abstract

**Question:**

Task-shared preventive psychological interventions combined with interventions addressing social determinants of mental health may prevent common mental health conditions (CMHCs), particularly in low- and middle-income countries (LMICs). However, an evidence synthesis of their combination has not yet been investigated. We aimed to systematically assess the effectiveness of these combined interventions in LMICs.

**Study selection and analysis:**

We searched Epistemonikos, CENTRAL, MEDLINE, Embase, PsycINFO, CINAHL, GIM, ClinicalTrials.gov and WHO ICTRP until 2 September 2024. Two reviewers independently abstracted the data and evaluated the risk of bias of included studies using the Cochrane Risk of Bias 2 tool. We performed random-effects meta-analyses to assess the primary outcome, which was the incidence of CMHCs, and rated the certainty of evidence using the Grading of Recommendations Assessment, Development, and Evaluation approach. The protocol was registered in PROSPERO (CRD42023451072).

**Findings:**

Of the 21 780 records identified from electronic sources, we included 31 randomised controlled trials from 21 LMICs involving 35 885 participants. Combined interventions were effective in reducing the incidence of depression and post-traumatic stress disorders at postintervention compared with control conditions for adults (risk ratio (RR) 0.82, 95% CI 0.73 to 0.93) and children (RR 0.70, 95% CI 0.49 to 0.99). At 7–24 months, we only found beneficial effects of combined interventions for depressive symptoms in children (standardised mean difference −0.41, 95% CI −0.63 to –0.18). No data were available on the incidence of anxiety.

**Conclusions:**

Combined task-shared interventions are effective in mostly short-term prevention of CMHCs in LMICs. Combining strategies targeting social determinants with psychological prevention approaches offers a potential opportunity to reduce the global mental health burden. Future research should focus on key intervention components and head-to-head comparisons between different interventions and between their components.

**PROSPERO registration number:**

CRD42023451072.

**WHAT IS ALREADY KNOWN ON THIS TOPIC**

- Common mental health conditions (CMHCs), such as depression, anxiety and post-traumatic stress disorder (PTSD), are highly prevalent in low- and middle-income countries (LMICs) and significantly contribute to the global disability burden. Psychological interventions and task-sharing approaches, also addressing social determinants of mental health, are key strategies for preventing CMHCs in LMICs. However, while preliminary evidence supports their effectiveness, no comprehensive synthesis of trials combining social and psychological intervention components is currently available.

**WHAT THIS STUDY ADDS**

- This is the first systematic review and meta-analysis focused on preventive interventions that combine psychological intervention with intervention components targeting social determinants of mental health. We included 31 studies from 21 LMICs involving adults and children/adolescents randomly assigned to task-shared preventive interventions or control conditions. Most interventions employed cognitive–behavioural techniques and addressed demographic, social and cultural domains of Lund’s framework.^5^ Primary outcomes showed that combined interventions effectively reduced depression and/or PTSD postintervention across all population groups. For secondary outcomes, there was a beneficial effect on depressive symptoms in the youth population at 7–24 months.

**HOW THIS STUDY MIGHT AFFECT RESEARCH, PRACTICE OR POLICY**

These findings highlight the increasing evidence supporting preventive interventions in global mental health, while also indicating that many social determinants are modifiable factors that should be integrated into the design and evaluation of interventions. The collected evidence provides a framework for planning evidence-based prevention strategies involving end- users, providers and policy makers. Future research should prioritise examining the individual components and mechanisms of combined approaches to better understand their specific contributions to improving mental health and psychological well-being.

## Background

 Common mental health conditions (CMHCs), defined here as depression, anxiety and post-traumatic stress disorder (PTSD), are highly prevalent in low- and middle-income countries (LMICs) and contribute significantly to the global burden of disease and disability.^1 2^ Moreover, the WHO estimates that the prevalence of CMHCs in conflict settings is at 22%,^3^ which is approximately five times higher than the prevalence in the general population, and in LMICs, a range of social factors, including poverty, urban violence and conflict exposure, malnutrition, gender-based violence and social inequalities, also influence the prevalence of CMHCs. Described as social determinants of mental health, these factors are the social and economic conditions—such as income inequality, forced migration and low levels of education—that may directly influence the onset, trajectories and severity of mental health conditions in the life course.^4^,^6^ Evidence indicates that CMHCs are strongly socially determined among populations worldwide,^7^ suggesting that approaches addressing the social determinants of mental health may be effective in preventing CMHCs.^4 5^

In LMICs, the prevention, diagnostic and treatment gaps remain wide. Most mental health needs remain largely unmet, with wide prevention, diagnostic and treatment gaps associated with CMHCs.^8^ Although these gaps are ubiquitous, reports from the WHO highlight persistent disparities in mental health resource availability, accessibility and adequacy between and within countries, regions and settings. This report emphasises the need for sustained national-level investment and improvements in mental health policies, plans, services and monitoring systems.^9^ Additionally, the global mental health community advocates for the implementation of prevention strategies as an important opportunity for addressing the high burden that mental health conditions pose for societies.^10^

According to the Institute of Medicine (IOM) framework, prevention interventions are aimed at reducing the likelihood of future disorders in the general population (universal prevention), or among those identified as at risk of a disorder (selective prevention), or among those displaying subclinical symptoms (indicated prevention).^11^ In LMICs, these preventive strategies are often implemented as brief, low-intensity psychosocial interventions, mostly delivered through a task-sharing approach. Task sharing is defined by the WHO as ‘*the rational redistribution of tasks among health workforce teams’*.^12^ Specific functions are shifted and reallocated, where appropriate, from highly qualified health professionals (such as psychiatrists and psychotherapists) to health workers with shorter training and fewer qualifications. Psychological interventions can be and are used across the prevention spectrum to address CMHCs,^13^ coherently with the WHO Mental Health Gap Action Programme Intervention Guide.^14^

A recent Lancet Commission sought to align global mental health efforts with Sustainable Development Goals (SDGs) and emphasised the importance of efforts to prevent mental health disorders.^10^ However, evidence on prevention is inconclusive. In particular, while there is preliminary evidence supporting the effectiveness of psychological interventions^13^ and programmes targeting the social determinants of mental health^6^ in preventing mental health disorders, a synthesis and critical appraisal of trials combining social (ie, seeking to change or improve a social determinant of mental health) and psychological intervention components is not available.

### Objective

The present systematic review and meta-analysis aims to comprehensively evaluate the effectiveness of combined interventions, which include both psychological components and components that address the social determinants of mental health, in preventing CMHCs in LMICs.

## Methods

We designed and conducted this systematic review following the Cochrane Handbook for Systematic Review of Interventions,^15^ and we comply with the Preferred Reporting Items for Systematic Reviews and Meta-Analyses 2020 guidelines^16^ (online supplemental appendix A). The review was guided by the conceptual framework developed by Lund and colleagues,^4^ which maps social determinants to five domains linked to the SDGs.

### Search strategy and study selection

We designed our search strategy according to the following criteria for eligible randomised controlled trials (RCTs). (1) They were conducted in LMICs, defined according to the World Bank criteria at the time the study was conducted,^17^ and (2) included participants of any age, gender, ethnicity and religion without any diagnosis of mental disorders at the time of recruitment (ie, at screening for inclusion/exclusion). The lack of a diagnosis at baseline had to be determined by a diagnostic tool or by a score below a clinical cut-off on a symptom checklist. (3) Experimental interventions were focused both on the social determinants of mental health and on psychological components, integrated into a single intervention; and/or interventions in which the social and psychological components were provided separately but whose combined effects were evaluated. (4) Interventions were delivered by primary health workers (PHWs), such as nurses and general practitioners, and/or community workers (CWs), including peers and volunteers, through a task-sharing approach.^18^ (5) Interventions were compared versus any type of control groups (ie, no treatment, waiting list or usual care).

We systematically searched Epistemonikos, Cochrane Controlled Trials Register (CENTRAL), MEDLINE, Embase, PsycINFO, CINAHL, Global Index Medicus (GMI), ClinicalTrials.gov (Ctgov) and WHO International Clinical Trials Registry Platform (ICTRP) without language or start date restrictions, until 2 September 2024. We adapted and cross-checked our search strategy with the search of a recent Cochrane review on prevention interventions in LMICs.^13^ Search terms included ‘RCTs’, ‘LMICs’, ‘psychosocial interventions’, ‘task-sharing’ and ‘mental health conditions’. The full search strings are reported in online supplemental appendix B. The reference lists of relevant reviews and the articles citing the included articles were manually examined to identify additional publications.

Our primary outcome was the composite incidence of any CMHC, defined here as depression, anxiety or PTSD at postintervention (≤1 month after finishing the intervention), as determined by a formal diagnostic tool (ie, the Diagnostic and Statistical Manual of Mental Disorders, International Classification of Diseases) or a cut-off on validated rating scale(s), even if studies themselves treated these as secondary outcomes. Secondary outcomes included the incidence of depression, anxiety or PTSD at 1–6 months postintervention, and at 7–24 months postintervention; change in symptoms of depression, anxiety and psychological distress measured with validated rating scales at each timepoint; and changes in service utilisation and contact coverage and resource use as qualitatively reported in studies.

Pairs of reviewers (EP, BB, RS) independently screened the records, selected the studies, reviewed the full texts, extracted the relevant information from the included trials and appraised the primary and secondary outcomes of the included studies using the Cochrane Risk of Bias 2 (RoB 2) tool for RCTs^19^ (online supplemental appendix G). Any discrepancies were double-checked and resolved by discussion with an experienced reviewer/mental health expert (CL and MP). We employed the Grading of Recommendations Assessment, Development, and Evaluation (GRADE) approach^20^ to assess the overall certainty of evidence and to interpret findings for the primary outcome (online supplemental appendix R). The study protocol for this systematic review was registered in PROSPERO (CRD42023451072) and published in a peer-reviewed journal.^21^

### Data analysis

We applied random-effects model meta-analyses using the Cochrane Review Manager V.5.4 software.^22^ For dichotomous data, we calculated risk ratio (RR) with 95% CI; for continuous data, we used mean difference (MD) or standardised mean difference (SMD), together with the 95% CI.^23^ We contacted the study authors for missing or unclear data. When the proportion of participants with depression or anxiety or PTSD at postintervention (primary outcome) was not reported in the study report(s), or authors did not respond to our data requests, we applied commonly employed cut-off scores of continuous measures of depression, anxiety and PTSD symptoms. We imputed the proportions of participants with a diagnosis of depression, anxiety or PTSD (online supplemental appendix P) using an established methodology.^24^ Data from cluster RCTs were adjusted according to the intracluster correlation coefficient (ICC). If the ICC was unavailable, we assumed it to be 0.05.^23 25^ We developed a dedicated data extraction form for economic data based on the format of the National Health Service Economic Evaluation Database^26^ and presented the results narratively (online supplemental appendix Q).

We conducted separate meta-analyses for children/adolescents (≤19 years)^27^ and adults (>19 years) according to the protocol.^21^ RCTs including mixed-population groups were allocated to one category or another according to the proportion of participants belonging to the groups (≥50%). Forest plots and I^2^ statistics were used to investigate statistical heterogeneity. We interpreted I^2^ estimates according to Cochrane methodology: 0–40% might not be important; 30–60% may represent moderate heterogeneity; 50–90% may represent substantial heterogeneity; 75–100% considerable heterogeneity.^23 24^ In meta-analyses including at least 10 RCTs, we performed subgroup analyses for the primary outcome, accounting for the social determinants domain according to the theoretical framework of Lund and colleagues (demographic, economic, neighbourhood, environmental events and sociocultural)^4 10^ (online supplemental appendix T). RCTs were linked to specific social determinant domains by EP and MP supervised by CL. Additionally, we analysed subgroups according to the type of prevention based on the IOM framework (universal, selective, indicated),^11 13^ type of providers (PHWs, CWs, mixed), gender, country income at the time of study implementation (low or lower middle or upper middle), humanitarian setting (vs non-humanitarian setting), as defined by Tol *et al*,^11^ and study design (cluster vs individual RCT). We performed sensitivity analyses excluding trials with a high risk of bias according to the Cochrane RoB 2, excluding trials with methodological characteristics, such as outcome measurement tools that might generate the highest heterogeneity in meta-analyses (I^2^>75%), particularly those tools for which a cultural adaptation was not conducted. We examined publication bias through funnel plots when we identified 10 or more RCTs, as per Cochrane standards.^23^

## Findings

**Figure 1:**
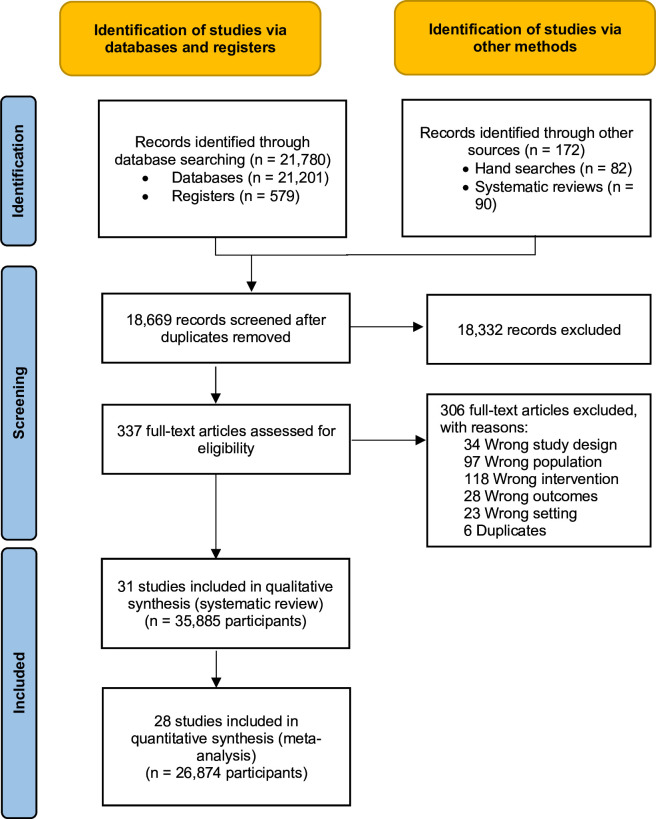
Preferred Reporting Items for Systematic Reviews and Meta-Analyses (PRISMA) flow chart diagram.

The search strategy yielded 21 780 records from electronic sources up to 2 September 2024. After removing duplicates (n=3283), 18 669 titles and abstracts were assessed for eligibility, 337 of which fulfilled the eligibility criteria. After inspection of full texts, a total of 31 RCTs with 35 885 participants were included in the systematic review (online supplemental appendix D). Of these, 28 RCTs, with 26 874 participants (n=11 151 after ICC correction), provided data for the meta-analysis (figure 1 and online supplemental appendices D and E).

We contacted four trial authors for additional information, and three replied to our requests (see the Acknowledgements section). We excluded 306 studies due to wrong study design, type of participants, interventions, outcomes and settings. The most common reason for exclusion was ineligible intervention (online supplemental appendix E). Of the 31 included studies, 14 were cluster-randomised trials. Four studies were carried out in South Africa, three in Uganda, two in Nepal, two in Kenya, two in Tanzania, two in Thailand, two in India and one each in Colombia, Zimbabwe, Lebanon, China, Bosnia and Herzegovina, Democratic Republic of Congo, Haiti, Jordan, Burundi, Jamaica, Sri Lanka, Brazil, Pakistan, Ecuador and Panamá (online supplemental appendix U). Nine studies focused on children, eight on adults and five specifically on women. 10 studies focused on caregiver-child dyads, three on family members (eg, parents and siblings) and one on teachers and students.

Various cadres of providers were employed: CWs (26 studies), PHWs (three studies) or both (one study). The intervention was delivered online in one study, and the specific cadres were not described.

Included studies tested indicated (n=14), selective (n=14) and universal (n=3) prevention interventions (online supplemental appendix C).

Classifying the interventions into the five domains of the social determinants of mental health,^11^ nine RCTs tested prevention interventions on the demographic domain, for example, targeting child maltreatment in the household or improving maternal mental health of women victims of domestic violence. The prevention interventions in four studies addressed the economic domain (ie, poverty, unemployment and income inequality); for example, family economic empowerment. In four studies, interventions addressed the neighbourhood domain (ie, safety and social deprivation), such as reducing drug use and associated sexual risks. In six studies, prevention interventions addressed the environmental domain, targeting war-exposed populations and people affected by natural disasters such as disaster preparedness. In eight studies, the prevention interventions addressed the social and cultural domain. For example, learning skills to improve study abilities and increase academic performance.

Most psychological components were based on cognitive–behavioural therapy, followed by the problem-solving approach, psychoeducation and counselling (online supplemental appendix F).

Among the included studies, two reported economic evaluations summarised using a narrative approach in online supplemental appendix Q.

Control conditions were waiting list in nine trials, usual care in 21 trials and no treatment in one trial, and other psychosocial interventions in two trials. Results on efficacy are presented separately for children and adults (table 1, online supplemental appendices H–M).

**Table 1:** Random-effects meta-analyses of combined interventions compared with control conditions at different timepoints

	Adults					Children and adolescents				
Outcome	Number of studies(participants)	Metric	Estimate	95% CI	I^2^	Number of studies(participants)	Metric	Estimate	95% CI	I^2^
**Postintervention**
Depressive symptoms	7 (992)	SMD	−0.43	−0.71 to −0.15	71%	4 (387)	SMD	−0.28	−0.48 to −0.08	0%
Anxiety symptoms	1 (128)	MD	−0.55	−0.90 to −0.20	NA	1 (103)	MD	−0.24	−0.63 to 0.15	NA
PTSD symptoms	8 (1490)	SMD	−0.19	−0.43 to 0.05	78%	2 (212)	SMD	−0.35	−0.62 to −0.08	0%
**1–6 months**										
Diagnosis of depression	1 (117)	RR	1.03	0.38 to 2.78	NA	–	–	–	–	–
Depressive symptoms	–	–	–	–	–	4 (896)	SMD	−0.10	−0.30 to 0.10	49%
Anxiety symptoms	1 (2026)	MD	−0.07	−0.15 to 0.02	NA	1 (176)	MD	−0.25	−0.55 to 0.05	NA
PTSD symptoms	3 (644)	SMD	−0.10	−0.26 to 0.06	3%	1 (213)	MD	−0.05	−0.32 to 0.22	NA
**7–24 months**										
Diagnosis of depression	–	–	–	–	–	1 (52)	RR	0.86	0.24 to 3.06	NA
Depressive symptoms	4 (1519)	SMD	−0.91	−2.01 to 0.19	99%	4 (1372)	SMD	−0.47	−0.77 to −0.16	78%
Anxiety symptoms	–	–	–	–	–	1 (52)	MD	−0.22	−0.77 to 0.33	NA
PTSD symptoms	2 (574)	SMD	−0.10	−0.74 to 0.53	93%	2 (572)	SMD	−0.04	−0.20 to 0.13	0%

SMD <0 favours psychosocial interventions.

RR <1 favours psychosocial interventions.

MD <0 favours psychosocial interventions.

Symbol ‘–’ denotes data not available.

MD, mean difference; PTSD, post-traumatic stress disorder; RR, risk ratio; SMD, standardised mean difference.

The proportions of participants with a diagnosis of depression, anxiety or PTSD (online supplemental appendix P) were dichotomised for 16 out of 22 outcomes. For the remaining six, one study already specified the outcome, while imputation was not possible for five outcomes due to the lack of a validated, culturally relevant cut-off for the scale (online supplemental appendix P).

**Figure 2:**
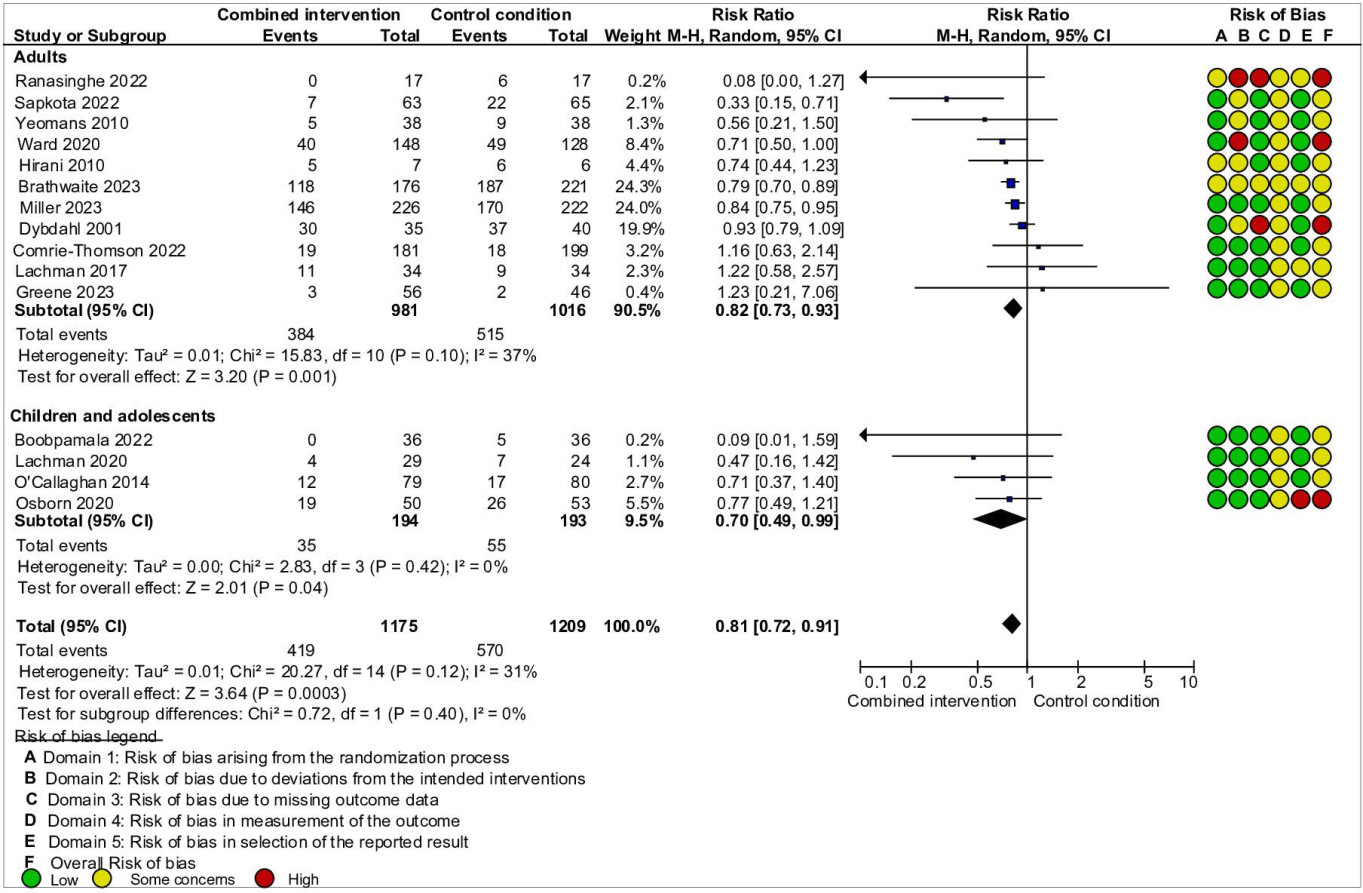
Efficacy of combined interventions in preventing the onset of depression and post-traumatic stress disorder (PTSD) at postintervention.

At postintervention, combined interventions decreased the diagnosis of depression and PTSD among adults (11 RCTs, 1997 participants, RR 0.82, 95% CI 0.73 to 0.93, I^2^=37%, GRADE: low certainty due to study limitations and indirectness) and children (4 RCTs, 387 participants, RR 0.70, 95% CI 0.49 to 0.99, I^2^=0%, GRADE: low certainty due to study limitations and indirectness) (figure 2). No data were available on the diagnosis of anxiety for both children and adults. Interventions significantly decreased depressive symptoms (7 RCTs, 992 participants, SMD −0.43, 95% CI −0.71 to –0.15, I^2^=71%) and anxiety symptoms (1 RCT, 128 participants, MD −0.55, 95% CI −0.90 to –0.20) but not PTSD symptoms (8 RCTs, 1490 participants, SMD −0.19, 95% CI −0.43 to 0.05, I^2^=78%) among adults (table 1, online supplemental appendix H). Meta-analyses showed a significant beneficial effect of combined interventions for depressive (4 RCTs, 387 participants, SMD −0.28, 95% CI −0.48 to –0.08, I^2^=0%) and PTSD symptoms (2 RCTs, 212 participants, SMD −0.35, 95% CI −0.62 to –0.08, I^2^=0%) but not for anxiety symptoms (1 RCT, 103 participants, MD −0.24, 95% CI −0.63 to 0.15) among children (table 1, online supplemental appendix I).

At 1–6 months, no effect was found for the diagnosis of depression, symptoms of anxiety or PTSD symptoms among adults (table 1, online supplemental appendix J). No data were available on depressive symptoms, diagnosis of anxiety and PTSD among adults. No effect was found for depressive, anxiety and PTSD symptoms among children (table 1, online supplemental appendix K). No data were available on the diagnosis of depression, anxiety or PTSD among children.

At 7–24 months, no effect was found for depressive and PTSD symptoms among adults (table 1, online supplemental appendix L). No data were available on diagnosis of depression, anxiety or PTSD, nor on anxiety symptoms. Meta-analyses showed a significant beneficial effect of combined interventions for reducing depression symptoms (5 RCTs, 1659 participants, SMD −0.41, 95% CI −0.63 to –0.18, I^2^=71%) among children. We found no significant effect in reducing the frequency of depression (1 RCT, 60 participants, RR 0.86, 95% CI 0.24 to 3.06), anxiety symptoms (1 RCT, 52 participants, MD −0.22, 95% CI −0.77 to 0.33) and PTSD symptoms (2 RCTs, 572 participants, SMD −0.04, 95% CI −0.20 to 0.13, I^2^=0%) among children (table 1, online supplemental appendix K).

We applied the Cochrane RoB 2 to the included RCTs. 13 outcomes from seven RCTs showed a high risk of bias in one or two domains (domain 5 or 3). The remaining outcomes were judged as ‘some concerns’. High risk emerged for domain 2 (risk of bias due to deviations from the intended interventions) and domain 4 (risk of bias in measurement of the outcome). Risk of bias summary and tables are provided in online supplemental appendix G.

To check the risk of publication bias, we visually inspected funnel plots for asymmetry in the comparisons between combined interventions and control conditions in preventing CMHCs at postintervention in adults, without identifying any asymmetry in the distribution of studies (online supplemental appendix S).

### Subgroup and sensitivity analyses

The limited number of RCTs included in this review allowed the preplanned subgroup analyses to be performed only for the adult population (online supplemental appendix N). For the primary outcome, exploratory subgroup analyses by country income showed a beneficial effect of the interventions in low- and upper middle-income countries, while no difference was observed between intervention and control in lower middle-income countries (p=0.60). Subgroup analysis considering RCTs with mixed-gender participants and RCTs with only female participants showed no difference across the subgroups (p=0.27). For males, no data were available. Subgroup analysis accounting for humanitarian versus no humanitarian settings showed no difference between subgroups (p=0.25). Regarding the type of social determinants domain, subgroup analysis showed no difference (p=0.55). Subgroup analysis for the type of control group showed the effect of combined interventions over usual care and waiting list (p=0.22). Regarding the type of provider, the test for subgroup differences was statistically significant (p=0.04), indicating that PHWs and CWs were separately effective in preventing CMHCs, while no differences were identified for mixed-provider groups. Subgroup analysis accounting for the type of prevention strategies showed that selective prevention was effective in preventing depression and PTSD at postintervention, while no difference was identified for universal and indicated prevention (p=0.80). Similarly, subgroup analysis considering the variability in study design showed no difference for cluster and individual randomisation (p=0.84).

We conducted a sensitivity analysis excluding studies (n=3) with a high risk of bias (online supplemental appendix O). The effect of combined interventions for adults on the incidence of a diagnosis of CMHC was significant at postintervention, also after the exclusion of studies with a high risk of bias (8 RCTs, 1612 participants, RR 0.81, 95% CI 0.71 to 0.92, I^2^=24%). We did not perform sensitivity analyses for the child population because we did not identify 10 or more RCTs for our primary outcome.

### Conclusion and clinical implications

To the best of our knowledge, this is the first systematic review and meta-analysis focused on preventive interventions that combine psychological intervention with intervention components targeting social determinant components. We included 31 studies from 21 countries, 13 testing family interventions delivered by CWs to adults. With regard to primary outcomes, combined preventive interventions were effective in reducing the frequency of depression and/or PTSD at postintervention in adults and children. For secondary outcomes, we found no differences between interventions versus controls, except for a beneficial effect of combined interventions on depressive and anxiety symptoms at postintervention for both age groups, and on depressive symptoms at 7–24 months in children. The subgroup findings indicate a short-term preventive effect of combined interventions specifically targeted at high-risk adult populations (ie, selective prevention), including refugees, people living in war-affected areas and women in the perinatal period. These selective prevention interventions demonstrate greater effectiveness when delivered by trained PHWs or CWs. This task-sharing approach enhances the accessibility of evidence-based interventions and strengthens the capacity of primary and community care systems.

Most interventions employed cognitive–behavioural techniques, targeting the demographic, social and cultural domains of the framework developed by Lund and colleagues.^4^ The primary aim of social determinant interventions in most of the included RCTs was preventing domestic violence towards children and women, and/or enhancing social capital, education and literacy levels. The interventions tested in the included primary studies were identified as priority strategies by the Academy of Medical Sciences and the InterAcademy Partnership for addressing the social determinants of mental health in a global setting.^5^

Our results on the primary outcomes align with those of a Cochrane review of 113 RCTs on the effectiveness of mental health preventive task-sharing interventions for people in LMICs.^13^ The review found a beneficial effect of preventive interventions in reducing the diagnoses of CMHCs at postinterventions for adults (RR 0.65). However, we observed a lack of long-term follow-ups and assessments, similar to other systematic reviews on psychological interventions in LMICs.^13^ This limited evidence on the long-term outcomes offers only a partial view of interventions’ effectiveness.

An additional insight from our review is that the evidence on children is growing, as highlighted by a mapping systematic review of psychosocial support interventions for children in LMICs.^28^ Yu and colleagues collected 697 studies and reviews from 78 LMICs and found that 61% of intervention research was conducted in schools. However, when it comes to the efficacy of these interventions, the literature presents mixed results. A systematic review by Grande and colleagues^29^ assessed the effectiveness of school-based interventions for the treatment of mental health conditions among children aged 6–18 in LMICs. Among the 39 studies included, when outcomes were analysed by condition, the pooled effects of interventions for anxiety, attention-deficit hyperactivity disorder and depression were not clinically significant.

Findings from our review should be interpreted considering some limitations. First, we labelled prevention interventions according to the IOM framework and after discussion with experts in our research team. However, the partial reporting of the conceptual framework in primary studies and the heterogeneity in the outcome measures made the classification challenging (ie, multiple rounds of discussion). For instance, many of the studies in our meta-analysis measured mental health outcomes using symptom severity measures rather than incidence using diagnostic tools. Second, we found moderate to high levels of statistical heterogeneity across studies, likely reflecting the complexity of conducting research with preventive purposes. This heterogeneity has been attributed to wide differences in implementation settings, intervention components, dosage, administration mode and country income level. Third, combining age groups with differing exposures to risk factors may explain the heterogeneity observed in results for some outcomes. Additionally, the optimal timing for a preventive intervention remains uncertain, as studies varied in when interventions were implemented relative to risk exposure. This may contribute to the observed heterogeneity in results. Fourth, for the outcomes of anxiety symptoms and long-term changes in CMHC symptoms, it was not possible to perform any meta-analyses as only one study was available. This limits the possibility of estimating the interventions’ effect for this outcome, but at the same time offers important insights on future research directions. Fifth, study limitations and indirectness were the main reasons contributing to the downgrading of the evidence to a low level of certainty according to the GRADE criteria. This means that more randomised evidence with robust methodologies is needed to assess the preventive effects of combined interventions for people living in LMICs.

Our review also holds several strengths. This is the first systematic review collecting evidence on integrating mental health strategies into broader development policies and practices in LMICs by addressing the social determinants of mental health, aligning with the United Nations SDGs.^10^ Our findings provide support to a novel approach to psychosocial interventions emphasising the social aetiology of mental health conditions.^7^ This approach is relevant in low-income settings, where factors such as poverty, food insecurity, gender-based violence, global income inequalities and humanitarian emergencies have a significant impact on mental health.^4^ Our approach has the potential to be expanded beyond the field of mental health to other areas of public health, including those not directly related to psychological well-being. Furthermore, its implementation could be broadened to encompass a wider range of social determinants of health beyond those discussed in this review. For instance, it could address unemployment by facilitating access to competitive job sectors, enhance neighbourhood infrastructures to promote healthier living conditions and tackle environmental determinants linked to climate change. Additionally, future studies should be designed with a clear prevention focus (ie, incidence) and use socioculturally appropriate and validated instruments to measure outcomes, thereby enabling better differentiation between distinct common mental health (CMH) diagnoses.

Overall, our findings highlight the effectiveness of combined interventions delivered through a task-sharing approach in preventing depression and PTSD in the short term for adults and children, though not for anxiety and the longer term (except preventing depressive symptoms among children). These findings support that many social determinants are modifiable factors that should be integrated into the design and evaluation of interventions. We acknowledge that our findings are limited to interventions delivered using a task-sharing model. Nevertheless, the task-sharing model emerges as a promising and scalable implementation strategy, particularly suited for delivering preventive interventions in underserved areas where access to specialised mental healthcare professionals may be limited. By redistributing responsibilities among trained non-specialist providers, this approach maximises available resources, enhances service reach and strengthens community-based healthcare systems.^10^ This highlights the importance of adopting an interdisciplinary, intersectoral and integrated approach in global mental health. Future research should prioritise examining the individual components and mechanisms of combined approaches to understand better their specific contributions to improving mental health and psychological well-being. Additionally, it is crucial to investigate the differential effects in preventing CMHCs when comparing combined and non-combined interventions, as demonstrated in the ALIVE programme.^30^ This would help identify which intervention components are most effective and why.

## Supplementary material

10.1136/bmjment-2025-301573 online supplemental file 1

## Data Availability

Data are available upon reasonable request.
