# Supplementary material for: Effectiveness of combining psychological prevention interventions with interventions that address the social determinants of mental health in low- and middle-income countries: a systematic review and meta-analysis
Source: BMJ Ment Health. 2025 May 26;28(1):e301573. doi: 10.1136/bmjment-2025-301573 (PMC12107591; doi:10.1136/bmjment-2025-301573)
Supplement: online supplemental file 1 [file bmjment-28-1-s001.docx]

**Effectiveness of combining task-sharing prevention psychological interventions with interventions that address the social determinants of mental health in low- and middle- income countries: a systematic review and meta-analysis**

**Supplemental material**

| **Appendix** |  | PRISMA checklist | p. 2 |
| --- | --- | --- | --- |
| **Appendix** |  | Search strategy | p. 5 |
| **Appendix** |  | Explanation of prevention classification for included interventions | p. 26 |
| **Appendix** |  | Trials and publications included in the systematic review | p. 29 |
| **Appendix** |  | Trials excluded from the systematic review, with reasons | p. 32 |
| **Appendix** |  | Characteristics of the included studies | p. 38 |
| **Appendix** |  | Risk of bias of the included studies | p. 50 |
| **Appendix** |  | Analyses 1 – Combined interventions compared to control group in preventing mental conditions among adults at post-intervention | p. 232 |
| **Appendix** |  | Analyses 2 – Combined interventions compared to control group in preventing mental conditions among children and adolescents at post-intervention | p. 235 |
| **Appendix** |  | Analyses 3 - Combined interventions compared to control group in preventing mental conditions among adults at 1 to 6 months post-intervention | p. 237 |
| **Appendix** |  | Analyses 4 - Combined interventions compared to control group in preventing mental conditions among children and adolescents at 1 to 6 months post-intervention | p. 238 |
| **Appendix** |  | Analyses 5 - Combined interventions compared to control group in preventing mental conditions among adults at 7 to 24 months post-intervention | p. 239 |
| **Appendix** |  | Analyses 6 - Combined interventions compared to control group in preventing mental conditions among children and adolescents at 7 to 24 months post-intervention | p. 240 |
| **Appendix** |  | Subgroup analysis – Combined interventions compared to control group in preventing mental conditions among adults at post-intervention | p. 242 |
| **Appendix** |  | Sensitivity analysis – Combined interventions compared to control group in preventing mental conditions among adults at post-intervention | p. 246 |
| **Appendix** |  | Dichotomization of depressive, anxiety and PTSD symptoms at post intervention (depression, anxiety, PTSD) | p. 247 |
| **Appendix** |  | Narrative economic analysis | p. 251 |
| **Appendix** |  | GRADE Table | p. 252 |
| **Appendix** |  | Publication bias | p. 253 |
| **Appendix** |  | List of key social determinants of mental health based on Lund's theoretical framework | p. 254 |
| **Appendix** |  | Characteristics of included studies by country, population, social determinants’ domain, interventions’ components, and outcomes. | p. 255 |

**Appendix A – PRISMA checklist**

| **Section and Topic** | **Item #** | **Checklist item** | **Location where item**  **is reported** |
| --- | --- | --- | --- |
| **TITLE** | | |  |
| Title | 1 | Identify the report as a systematic review. | p. 1 |
| **ABSTRACT** | | |  |
| Abstract | 2 | See the PRISMA 2020 for Abstracts checklist. | p. 2 |
| **INTRODUCTION** | | |  |
| Rationale | 3 | Describe the rationale for the review in the context of existing knowledge. | p. 3, 4 |
| Objectives | 4 | Provide an explicit statement of the objective(s) or question(s) the review addresses. | p. 4 |
| **METHODS** | | |  |
| Eligibility criteria | 5 | Specify the inclusion and exclusion criteria for the review and how studies were grouped for the syntheses. | p. 5-7 |
| Information sources | 6 | Specify all databases, registers, websites, organisations, reference lists and other sources searched or consulted to identify studies. Specify the date when each source was last searched or consulted. | p. 5, 6; Appendix B |
| Search strategy | 7 | Present the full search strategies for all databases, registers and websites, including any filters and limits used. | Appendix B |
| Selection process | 8 | Specify the methods used to decide whether a study met the inclusion criteria of the review, including how many reviewers screened each record and each report retrieved, whether they worked independently, and if applicable, details of automation tools used in the process. | p. 5, 6 |
| Data collection process | 9 | Specify the methods used to collect data from reports, including how many reviewers collected data from each report, whether they worked independently, any processes for obtaining or confirming data from study investigators, and if applicable, details of automation tools used in the process. | p. 6 |
| Data items | 10a | List and define all outcomes for which data were sought. Specify whether all results that were compatible with each outcome domain in each study were sought (e.g. for all measures, time points, analyses), and if not, the methods used to decide which results to collect. | p. 6 |
|  | 10b | List and define all other variables for which data were sought (e.g. participant and intervention characteristics, funding sources). Describe any assumptions made about any missing or unclear information. | p. 6 |
| Study risk of bias assessment | 11 | Specify the methods used to assess risk of bias in the included studies, including details of the tool(s) used, how many reviewers assessed each study and whether they worked independently, and if applicable, details of automation tools used in the process. | p. 6 |
| Effect measures | 12 | Specify for each outcome the effect measure(s) (e.g. risk ratio, mean difference) used in the synthesis or presentation of results. | p. 7 |
| Synthesis methods | 13a | Describe the processes used to decide which studies were eligible for each synthesis (e.g. tabulating the study intervention characteristics and comparing against the planned groups for each synthesis (item #5)). | p. 6 |
|  | 13b | Describe any methods required to prepare the data for presentation or synthesis, such as handling of missing summary statistics, or data conversions. | p. 6, 7 |
|  | 13c | Describe any methods used to tabulate or visually display results of individual studies and syntheses. | p. 7, 8 |
|  | 13d | Describe any methods used to synthesize results and provide a rationale for the choice(s). If meta-analysis was performed, describe the model(s), method(s) to identify the presence and extent of statistical heterogeneity, and software package(s) used. | p. 7, 8 |
|  | 13e | Describe any methods used to explore possible causes of heterogeneity among study results (e.g. subgroup analysis, meta-regression). | p. 7, 8 |
|  | 13f | Describe any sensitivity analyses conducted to assess robustness of the synthesized results. | p. 8 |
| Reporting bias assessment | 14 | Describe any methods used to assess risk of bias due to missing results in a synthesis (arising from reporting biases). | p. 6 |
| Certainty assessment | 15 | Describe any methods used to assess certainty (or confidence) in the body of evidence for an outcome. | p. 6 |
| **Section and Topic** | **Item**  **#** | **Checklist item** | **Location where item**  **is reported** |
| **RESULTS** | | |  |
| Study selection | 16a | Describe the results of the search and selection process, from the number of records identified in the search to the number of studies included in the review, ideally using a flow diagram. | p. 9; Figure 1 |
|  | 16b | Cite studies that might appear to meet the inclusion criteria, but which were excluded, and explain why they were excluded. | p. 9; Appendix E |
| Study characteristics | 17 | Cite each included study and present its characteristics. | p.9, 10; Appendix D, F |
| Risk of bias in studies | 18 | Present assessments of risk of bias for each included study. | p. 12; Appendix G |
| Results of individual studies | 19 | For all outcomes, present, for each study: (a) summary statistics for each group (where appropriate) and (b) an effect estimate and its precision (e.g. confidence/credible interval), ideally using structured tables or plots. | p. 10-12; Figure 2; Table 2; Appendix H-M |
| Results of syntheses | 20a | For each synthesis, briefly summarise the characteristics and risk of bias among contributing studies. | p. 10-12 |
|  | 20b | Present results of all statistical syntheses conducted. If meta-analysis was done, present for each the summary estimate and its precision (e.g. confidence/credible interval) and measures of statistical heterogeneity. If comparing groups, describe the direction of the effect. | p. Appendix H-M |
|  | 20c | Present results of all investigations of possible causes of heterogeneity among study results. | p. 10-12 |
|  | 20d | Present results of all sensitivity analyses conducted to assess the robustness of the synthesized results. | p. 13; Appendix O |
| Reporting biases | 21 | Present assessments of risk of bias due to missing results (arising from reporting biases) for each synthesis assessed. | p. 12; Appendix G |
| Certainty of evidence | 22 | Present assessments of certainty (or confidence) in the body of evidence for each outcome assessed. | p.10-12; Appendix R |
| **DISCUSSION** | | |  |
| Discussion | 23a | Provide a general interpretation of the results in the context of other evidence. | p. 13, 14 |
|  | 23b | Discuss any limitations of the evidence included in the review. | p. 14, 15 |
|  | 23c | Discuss any limitations of the review processes used. | p. 14, 15 |
|  | 23d | Discuss implications of the results for practice, policy, and future research. | p. 15 |
| **OTHER INFORMATION** | | |  |
| Registration and protocol | 24a | Provide registration information for the review, including register name and registration number, or state that the review was not registered. | p. 6 |
|  | 24b | Indicate where the review protocol can be accessed, or state that a protocol was not prepared. | p. 6 |
|  | 24c | Describe and explain any amendments to information provided at registration or in the protocol. | NA |
| Support | 25 | Describe sources of financial or non-financial support for the review, and the role of the funders or sponsors in the review. | p. 8 |
| Competing interests | 26 | Declare any competing interests of review authors. | p. 16 |
| Availability of data, code and other materials | 27 | Report which of the following are publicly available and where they can be found: template data collection forms; data extracted from included studies; data used for all analyses; analytic code; any other materials used in the review. | p. 16 |

# Appendix B - Search strategy

Last update: September 2^nd^ 2024

**Epistemonikos, Epistemonikos Foundation (www.epistemonikos.org/)**

| psych* OR mental OR mentally |
| --- |
| AND |
| "task shift" OR task-shift OR "task shifting" OR task-shifting OR "shifting tasks" OR "task sharing" OR task-sharing OR "sharing tasks" OR "delegating tasks" OR "task delegation" |
| Limited to Publication type: Broad Synthesis, Structured Summary, Systematic Review |

**Cochrane Central Register of Controlled Trials (CENTRAL)**

|  | Search |
| --- | --- |
| #1 | MeSH descriptor: [Psychosocial Intervention] this term only |
| #2 | [mh “mental disorders”] |
| #3 | [mh “mania”] |
| #4 | [mh “mental health”] |
| #5 | [mh “depression”] |
| #6 | [mh "child development"] |
| #7 | [mh “mentally disabled persons”] |
| #8 | [mh "self-injurious behavior"] |
| #9 | ((mental next health*) or (mental* next ill*) or (mental* next disorder*) or (mental* next well*)):ti,ab,kw |
| #10 | ((substance or alcohol or opioid or morphine or marijuana or heroin or cocaine) near/2 (disorder* or illness* or dependence or abuse or misuse or use)):ti,ab,kw |
| #11 | (depressi* near/2 (sign* or symptom* or disorder*)):ti,ab,kw |
| #12 | (depress* near/3 (acute or clinical* or diagnos* or disorder* or major or unipolar or illness or scale* or score* or adult* or child* or adolesc* or teen* or youth* or elder* or late* life* or patient* or participant* or people or inpatient* or (in next patient*) or outpatient* or (out next patient*))):ti,ab,kw |
| #13 | ((depress* or distress*) near/3 (postnatal* or post natal* or maternal*)):ti,ab,kw |
| #14 | (depression or anxiety or alzheimer* or schizoaffective or mania or manic or "borderline personality" or (stress near/2 disorder*) or (adjustment next disorder*) or (psychological next/1 trauma*) or schizophrenia or psychoses or psychosis or (stress next syndrome*) or (distress next syndrome*) or (combat next disorder*) or (war next disorder*) or ptsd or dementia):ti,ab,kw |
| #15 | (((post next trauma*) or posttrauma*) near/3 (stress* or disorder*)):ti,ab,kw |
| #16 | ((psychological next trauma) or psychotrauma*):ti,ab,kw |
| #17 | (alcoholism or alcoholic* or (drug next addict*) or (drug next abus*) or (drug next misuse) or (drug next user*)):ti,ab,kw |
| #18 | ((learning or mental* or intellectual) next (disabled or disabilit* or disorder* or difficult*)):ti,ab,kw |
| #19 | ((dissociative near/3 (disorder* or reaction*)) or dissociation):ti,ab,kw |
| #20 | ((bipolar or behavioral or beahavioural or obsessive or panic or mood or delusional) near/2 (disorder* or illness* or disease*)):ti,ab,kw |
| #21 | (trichotillomani* or OCD or (obsess* next compulsi*) or GAD or (stress next reaction*) or "acute stress" or neurosis or neuroses or neurotic or mania):ti,ab,kw |
| #22 | (affective* next (disorder* or disease* or illness* or symptom*)):ti,ab,kw |
| #23 | ((mental or psychological or emotional or (psycho next social) or psychosocial) next (stress* or distress*)):ti,ab,kw |
| #24 | ((sub-syndrom* or sub-threshold or sub-clinical or subsyndrom* or subthreshold or subclinical or minor or brief) next (symptom* or disorder* or condition* or depress* or anxiety)):ti,ab,kw |
| #25 | ("mental relapse" or fatigue or (somatic next symptom*) or worry or worries or panic or (low next mood*) or (mood next problem*)):ti,ab,kw |
| #26 | ((anxiety next disorder*) or agoraphobi* or (general* next anxi*) or "separation anxiety" or "neurocirculatory asthenia" or (neurotic next disorder*) or (social next phobi*) or (self next harm*) or (self next injur*) or suicid*):ti,ab,kw |
| #27 | (slow* next (thought* or think*)):ti,ab,kw |
| #28 | (mental* next develop*):ti,ab,kw |
| #29 | {or #2-#28} |
| #30 | [mh “primary health care”] |
| #31 | [mh “physicians, family”] |
| #32 | [mh “physicians, primary care”] |
| #33 | [mh “general practitioners”] |
| #34 | [mh “general practice”] |
| #35 | [mh “family practice”] |
| #36 | [mh “social support”] |
| #37 | [mh “community health workers”] |
| #38 | [mh "allied health personnel"] |
| #39 | [mh “community health services”] |
| #40 | [mh “schools”] |
| #41 | [mh “school health services”] or [mh “school mental health services”] |
| #42 | [mh “rural health”] |
| #43 | [mh “rural population”] |
| #44 | [mh “nurses, community health”] |
| #45 | [mh “nurses, public health”] |
| #46 | [mh “family nursing”] |
| #47 | [mh “primary care nursing”] |
| #48 | [mh “rural nursing”] |
| #49 | [mh “community health nursing”] |
| #50 | [mh “school nursing”] |
| #51 | (primary near/5 (care or health*)):ti,ab,kw |
| #52 | ((family next practi*) or (family next doctor*) or (family next physician*) or gp* or (general next practi*)):ti,ab,kw |
| #53 | (school* or teacher* or rural* or community):ti,ab,kw |
| #54 | ((non next specialist*) or nonspecialist* or (social next worker*) or trainer*):ti,ab,kw |
| #55 | (psycho next social or psychosocial):ti,ab,kw |
| #56 | (caregiver* or (care next giver*) or layperson*):ti,ab,kw |
| #57 | (paraprofessional* or auxiliar* or paramedics or (para next professional*) or (non next physician*) or (non next clinician*)):ti,ab,kw |
| #58 | (allied near/2 (professional* or person* or staff or worker*)):ti,ab,kw |
| #59 | (lay near/2 (heal* or person* or counsellor* or counselor* or worker* or therapist*)):ti,ab,kw |
| #60 | (midwife or midwive* or pharmacist* or pharmacy or pharmacies or (practice next nurs*) or (district next nurs*) or (health next visitor*)):ti,ab,kw |
| #61 | (psychological-intervention* or task-shift* or shifting-tasks or task-sharing or sharing-tasks or delegating-tasks or task-delegation):ti,ab,kw |
| #62 | {or #30-#61} |
| #63 | #29 and #62 |
| #64 | (afghan* OR africa* OR albania* OR algeria* OR angola* OR antigua* OR barbuda* OR argentin* OR armenia* OR aruba* OR azerbaijan* OR bahrain* OR bangladesh* OR bengal* OR bangal* OR barbados* OR barbadian* OR bajan OR bajans OR belarus* OR belorus* OR byelarus* OR byelorus* OR belize* OR benin* OR dahomey OR bhutan* OR bolivia* OR bosnia* OR herzegovin* OR botswan* OR batswan* OR bechuanaland OR brazil* OR brasil* OR bulgaria* OR burkina* OR burkinese* OR upper-volta* OR burundi* OR urundi* OR cabo-verde* OR cape-verde* OR cambodia* OR kampuchea* OR khmer* OR cameroon* OR cameroun* OR ubangi-shari* OR chad* OR chile* OR china* OR chinese OR colombia* OR comoro* OR comore* OR comorian* OR mayotte* OR congo* OR zaire* OR costa-rica* OR (cote* AND *ivoir*) OR ivory-coast* OR ivorian* OR croatia* OR cuba* OR cyprus* OR cypriot* OR czech* OR djibouti* OR french-somaliland* OR dominica* OR ecuador* OR egypt* OR united-arab-republic* OR el-salvador* OR salvadoran* OR guinea* OR equatoguinea* OR eritrea* OR estonia* OR eswatini* OR swaziland* OR swazi* OR swati* OR ethiopia* OR fiji* OR gabon* OR gabonese* OR gabonaise* OR gambia* OR ((georgia OR georgian OR georgians) NOT (atlanta OR california OR florida)) OR ghana* OR gibraltar* OR greece* OR greek* OR grecian* OR grenada* OR grenadian* OR guam* OR guatemala* OR guyana* OR guiana* OR guyanese* OR haiti* OR hispaniola* OR hondura* OR hungary* OR hungarian* OR india* OR indonesia* OR iran* OR iraq* OR isle-of-man* OR jamaica* OR jordan* OR kazakh* OR kenya* OR karabati* OR korea* OR kosovo* OR kosova* OR kyrgyz* OR kirgiz* OR kirghiz* OR laos OR lao OR laotian* OR latvia* OR lebanon* OR lebanese* OR lesotho* OR lesothan* OR lesothonian* OR basutoland* OR mosotho* OR basotho* OR liberia* OR libya* OR jamahiriya* OR lithuania* OR macedonia* OR madagasca* OR malagasy* OR malawi* OR nyasaland* OR malaysia* OR malay-federation OR malaya-federation OR malayan-federation OR maldives* OR maldivian* OR indian-ocean* OR mali* OR malta* OR maltese* OR micronesia* OR marshallese* OR kiribati* OR marshall-island* OR nauru OR nauran OR nauruans OR nauran* OR mariana OR marianas OR palau OR paluan* OR tuvalu* OR mauritania* OR mauritan* OR mauritius* OR mexico* OR mexican* OR moldova* OR moldovia* OR mongol* OR montenegr* OR morocco* OR moroccan* OR ifni OR mozambique* OR mozambican* OR myanmar* OR burma* OR burmese OR namibia* OR nepal* OR new-caledonia* OR netherlands-antill* OR nicaragua* OR niger* OR oman* OR pakistan* OR palestin* OR gaza* OR west-bank* OR panama* OR paraguay* OR peru* OR philippine* OR philipine* OR phillipine* OR phillippine* OR filipino* OR filipina* OR poland* OR polish OR pole OR poles OR portugal* OR portuguese OR puerto-ric* OR romania* OR russia* OR ussr* OR soviet* OR rwanda* OR rwandese OR ruanda* OR ruandese OR samoa* OR navigator-island* OR pacific-island* OR polynesia* OR sao-tome* OR santomean* OR saudi-arabia* OR saudi OR saudis OR senegal* OR serbia* OR seychell* OR sierra-leone* OR slovak* OR sloven* OR melanesia* OR solomon-island* OR norfolk-island* OR somali* OR sri-lanka* OR ceylon* OR saint-kitts OR st-kitts OR kittian* OR nevisian* OR saint-lucia* OR st-lucia* OR saint-vincent* OR st-vincent* OR vincentian* OR grenadine* OR sudan* OR surinam* OR syria* OR tajik* OR tadjik* OR tadzhik* OR tanzania* OR tanganyika* OR thai* OR timor-leste* OR east-timor* OR timorese* OR togo* OR tonga* OR trinidad* OR tobago* OR tunisia* OR turkiy* OR turkey* OR turk OR turks OR turkish OR turkmen* OR uganda* OR ukrain* OR uruguay* OR uzbek* OR vanuatu* OR new-hebrides OR venezuela* OR vietnam* OR viet-nam* OR yemen* OR yugoslav* OR zambia* OR zimbabwe* OR rhodesia* OR arab-countr* OR arabic-countr* OR middle-east* OR global-south OR sahara* OR subsahara* OR magreb* OR maghrib* OR west-indies* OR caribbean* OR central-america* OR latin-america* OR south-america* OR asia-central OR central-asia* OR asia-northern OR north-asia* OR northern-asia* OR asia-southeastern OR southeastern-asia* OR south-eastern-asia* OR southeast-asia* OR south-east-asia* OR asia-western OR west-asia* OR western-asia* OR europe-eastern OR east-europe* OR eastern-europe* OR developing-countr* OR developing-nation* OR developing-population* OR developing-world OR less-developed-countr* OR less-developed-nation* OR less-developed-world OR lesser-developed-countr* OR lesser-developed-nation* OR lesser-developed-world OR under-developed-countr* OR under-developed-nation* OR under-developed-world OR underdeveloped-countr* OR underdeveloped-nation* OR underdeveloped-world OR middle-income-countr* OR middle-income-nation* OR middle-income-population* OR low-income-countr* OR low-income-nation* OR low-income-population* OR lower-income-countr* OR lower-income-nation* OR lower-income-population* OR underserved-countr* OR underserved-nation* OR underserved-population* OR under-served-population* OR under-served-nation* OR under-served-population* OR deprived-countr* OR deprived-population* OR high-burden-countr* OR high-burden-nation* OR countdown-countr* OR countdown-nation* OR poor-countr* OR poor-nation* OR poor-population* OR poor-world OR poorer-countr* OR poorer-nation* OR poorer-population* OR poorer-world OR developing-econom* OR less developed-econom* OR underdeveloped-econom* OR under-developed-econom* OR middle-income-econom* OR low-income-econom* OR lower-income-econom* OR low-gdp OR low-gnp OR low-gross-domestic OR low-gross-national OR lower-gdp OR lower-gnp OR lower-gross-domestic OR lower-gross-national OR lmic OR lmics OR third-world OR lami-countr* OR transitional-countr* OR emerging-econom* OR emerging-nation*):ti,ab,kw |
| #65 | (#1 or #63) and #64 |
| #66 | #62 and #64 with 'Dementia and Cognitive Improvement', 'Schizophrenia', 'Common Mental Disorders', 'Drugs and Alcohol', 'Developmental, Psychosocial and Learning Problems' in Cochrane Groups |
| #67 | #65 or #66 |
| #68 | #67 |
| #69 | #67 |
| #70 | #68 or #69 in Trials |

**Ovid MEDLINE**

| 1 | psychosocial intervention/ |
| --- | --- |
| 2 | exp mental disorders/ |
| 3 | mania/ |
| 4 | mental health/ |
| 5 | depression/ |
| 6 | child development/ |
| 7 | mentally disabled persons/ |
| 8 | exp self-injurious behavior/ |
| 9 | (mental health* or mental* ill* or mental* disorder* or mental* well*).ti,ab,kf. |
| 10 | ((substance or alcohol or opioid or morphine or marijuana or heroin or cocaine) adj2 (disorder* or illness* or dependence or abuse or misuse or "use")).ti,ab,kf. |
| 11 | (depressi* adj2 (sign* or symptom* or disorder*)).ti,ab,kf. |
| 12 | (depress* adj3 (acute or clinical* or diagnos* or disorder* or major or unipolar or illness or scale* or score* or adult* or child* or adolesc* or teen* or youth* or elder* or late* life* or patient* or participant* or people or inpatient* or in-patient* or outpatient* or out-patient*)).ti,ab,kf. |
| 13 | ((depress* or distress*) adj3 (postnatal* or post natal* or maternal*)).ti,ab,kf. |
| 14 | (depression or anxiety or alzheimer* or schizoaffective or mania or manic or borderline personality or (stress adj2 disorder*) or adjustment disorder* or (psychological adj1 trauma*) or schizophrenia or psychoses or psychosis or stress syndrome* or distress syndrome* or combat disorder* or war disorder* or ptsd or dementia).ti,ab,kf. |
| 15 | ((post-trauma* or posttrauma*) adj3 (stress* or disorder*)).ti,ab,kf. |
| 16 | (psychological trauma or psychotrauma*).ti,ab,kf. |
| 17 | (alcoholism or alcoholic* or drug addict* or drug abus* or drug misuse or drug user*).ti,ab,kf. |
| 18 | ((learning or mental* or intellectual) adj (disabled or disabilit* or disorder* or difficult*)).ti,ab,kf. |
| 19 | ((dissociative adj3 (disorder* or reaction*)) or dissociation).ti,ab,kf. |
| 20 | ((bipolar or behavio?ral or obsessive or panic or mood or delusional) adj2 (disorder* or illness* or disease*)).ti,ab,kf. |
| 21 | (trichotillomani* or OCD or obsess*-compulsi* or GAD or stress reaction* or acute stress or neuros#s or neurotic).ti,ab,kf. |
| 22 | (affective* adj (disorder* or disease* or illness* or symptom*)).ti,ab,kf. |
| 23 | ((mental or psychological or emotional or psycho-social or psychosocial) adj (stress* or distress*)).ti,ab,kf. |
| 24 | ((sub-syndrom* or sub-threshold or sub-clinical or subsyndrom* or subthreshold or subclinical or minor or brief) adj (symptom* or disorder* or condition* or depress* or anxiety)).ti,ab,kf. |
| 25 | (mental relapse or fatigue or somatic symptom* or worry or worries or panic or low mood* or mood problem*).ti,ab,kf. |
| 26 | (anxiety disorder* or agoraphobi* or general* anxi* or separation anxiety or neurocirculatory asthenia or neurotic disorder* or social phobi* or self-harm* or self-injur* or suicid*).ti,ab,kf. |
| 27 | (slow* adj (thought* or think*)).ti,ab,kf. |
| 28 | (mental* adj develop*).ti,ab,kf. |
| 29 | or/2-28 |
| 30 | primary health care/ |
| 31 | physicians, family/ |
| 32 | physicians, primary care/ |
| 33 | general practitioners/ |
| 34 | general practice/ |
| 35 | family practice/ |
| 36 | exp social support/ |
| 37 | community health workers/ |
| 38 | exp allied health personnel/ |
| 39 | exp community health services/ |
| 40 | schools/ |
| 41 | school health services/ or school mental health services/ |
| 42 | rural health/ |
| 43 | rural population/ |
| 44 | nurses, community health/ |
| 45 | nurses, public health/ |
| 46 | family nursing/ |
| 47 | primary care nursing/ |
| 48 | rural nursing/ |
| 49 | community health nursing/ |
| 50 | school nursing/ |
| 51 | (primary adj5 (care or health*)).ti,ab,kf. |
| 52 | (family practi* or family doctor* or family physician* or gp* or general practi*).ti,ab,kf. |
| 53 | (school* or teacher* or rural* or community).ti,ab,kf. |
| 54 | (non-specialist* or nonspecialist* or social worker* or trainer*).ti,ab,kf. |
| 55 | (psycho-social or psychosocial).ti,ab,kf. |
| 56 | (caregiver* or care giver* or layperson*).ti,ab,kf. |
| 57 | (lay adj2 (heal* or person* or counsellor* or counselor* or worker* or therapist*)).ti,ab,kf. |
| 58 | (paraprofessional* or para-professional* or auxiliar* or paramedics or (allied health* adj (professional* or person* or staff or worker*)) or non-physician* or non-clinician*).ti,ab,kf. |
| 59 | (midwife or midwive* or pharmacist* or pharmacy or pharmacies or practice nurs* or district nurs* or health visitor*).ti,ab,kf. |
| 60 | (psychological intervention* or task shift* or shifting tasks or task sharing or sharing tasks or (delegate adj2 task*)).ti,ab,kf. |
| 61 | or/30-60 |
| 62 | 1 or (29 and 61) |
| 63 | (afghan* or africa* or albania* or algeria* or angola* or antigua* or barbuda* or argentin* or armenia* or aruba* or azerbaijan* or bahrain* or bangladesh* or bengal* or bangal* or barbados* or barbadian* or bajan or bajans or belarus* or belorus* or byelarus* or byelorus* or belize* or benin* or dahomey or bhutan* or bolivia* or bosnia* or herzegovin* or botswan* or batswan* or bechuanaland* or brazil* or brasil* or bulgaria* or burkina* or burkinese* or upper volta* or burundi* or urundi* or cabo verde* or cape verde* or cambodia* or kampuchea* or khmer* or cameroon* or cameroun* or ubangi shari* or chad* or chile* or china* or chinese or colombia* or comoro* or comore* or comorian* or mayotte* or congo* or zaire* or costa rica* or "cote d'ivoir*" or "cote d' ivoir*" or cote divoir* or cote d ivoir* or ivory coast* or ivorian* or croatia* or cuba or cuban or cubans or "cuba's" or cyprus* or cypriot* or czech* or djibouti* or french somaliland* or dominica* or ecuador* or egypt* or united arab republic* or el salvador* or salvadoran* or guinea* or equatoguinea* or eritrea* or estonia* or eswatini* or swaziland* or swazi* or swati* or ethiopia* or fiji* or gabon* or gabonese* or gabonaise* or gambia* or ((georgia or georgian or georgians) not (atlanta or california or florida)) or ghana* or gibraltar* or greece* or greek* or grecian* or grenada* or grenadian* or guam* or guatemala* or guyana* or guiana* or guyanese* or haiti* or hispaniola* or hondura* or hungary* or hungarian* or india* or indonesia* or iran* or iraq* or isle of man* or jamaica* or jordan* or kazakh* or kenya* or karabati* or korea* or kosovo* or kosova* or kyrgyz* or kirgiz* or kirghiz* or laos or lao or laotian* or latvia* or lebanon* or lebanese* or lesotho* or lesothan* or lesothonian* or basutoland* or mosotho* or basotho* or liberia* or libya* or jamahiriya* or lithuania* or macedonia* or madagasca* or malagasy* or malawi* or nyasaland* or malaysia* or malay* federation or maldives* or maldivian* or indian ocean or mali or malian* or "mali's" or malta or maltese* or "malta's" or micronesia* or marshallese* or kiribati* or marshall island* or nauru or nauran or nauruans or "naurian's" or mariana or marianas or palau or paluan* or tuvalu* or mauritania* or mauritan* or mauritius* or mexico* or mexican* or moldova* or moldovia* or mongol* or montenegr* or morocco* or moroccan* or ifni or mozambique* or mozambican* or myanmar* or burma* or burmese or namibia* or nepal* or new caledonia* or netherlands antill* or nicaragua* or niger* or oman or omani or omanis or "oman's" or pakistan* or palestin* or gaza* or west bank* or panama* or paraguay* or peru or peruvian* or "peru's" or philippine* or philipine* or phillipine* or phillippine* or filipino* or filipina* or poland* or polish or pole or poles or portugal* or portuguese or puerto ric* or romania* or russia* or ussr* or soviet* or rwanda* or rwandese or ruanda* or ruandese or samoa* or navigator island* or pacific island* or polynesia* or sao tome* or santomean* or saudi arabia* or saudi or saudis or senegal* or serbia* or seychell* or sierra leone* or slovak* or sloven* or melanesia* or solomon island* or norfolk island* or somali* or sri lanka* or ceylon* or saint kitts or st kitts or kittian* or nevisian* or saint lucia* or st lucia* or saint vincent* or st vincent* or vincentian* or grenadine* or sudan* or surinam* or syria* or tajik* or tadjik* or tadzhik* or tanzania* or tanganyika* or thai* or timor leste* or east timor* or timorese* or togo or togoles* or "togo's" or tonga* or trinidad* or tobago* or tunisia* or turkiy* or turkey* or turk or turks or turkish or turkmen* or uganda* or ukrain* or uruguay* or uzbek* or vanuatu* or new hebrides* or venezuela* or vietnam* or viet nam* or yemen* or yugoslav* or zambia* or zimbabwe* or rhodesia* or arab* countr* or middle east* or global south or sahara* or subsahara* or magreb* or maghrib* or west indies* or caribbean* or central america* or latin america* or south america* or asia central or central asia* or asia northern or north asia* or northern asia* or asia southeastern or southeastern asia* or south eastern asia* or southeast asia* or south east asia* or asia western or west asia* or western asia* or europe eastern or east europe* or eastern europe* or developing countr* or developing nation* or developing population* or developing world or less developed countr* or less developed nation* or less developed world or lesser developed countr* or lesser developed nation* or lesser developed world or under developed countr* or under developed nation* or under developed world or underdeveloped countr* or underdeveloped nation* or underdeveloped world or middle income countr* or middle income nation* or middle income population* or low income countr* or low income nation* or low income population* or lower income countr* or lower income nation* or lower income population* or underserved countr* or underserved nation* or underserved population* or under served population* or under served nation* or under served population* or deprived countr* or deprived population* or high burden countr* or high burden nation* or countdown countr* or countdown nation* or poor countr* or poor nation* or poor population* or poor world or poorer countr* or poorer nation* or poorer population* or poorer world or developing econom* or less developed econom* or underdeveloped econom* or under developed econom* or middle income econom* or low income econom* or lower income econom* or low gdp or low gnp or low gross domestic or low gross national or lower gdp or lower gnp or lower gross domestic or lower gross national or lmic or lmics or third world or lami countr* or transitional countr* or emerging econom* or emerging nation*).ti,ab,hw,kf. |
| 64 | exp randomized controlled trial/ |
| 65 | controlled clinical trial.pt. |
| 66 | randomi#ed.ti,ab. |
| 67 | placebo.ab. |
| 68 | randomly.ti,ab. |
| 69 | Clinical Trials as topic.sh. |
| 70 | trial.ti. |
| 71 | or/64-70 |
| 72 | exp animals/ not humans/ |
| 73 | 71 not 72 [Methods filter] |
| 74 | 62 and 63 and 73 |
| 75 | 2023*.dt,dp,ed,ep. |
| 76 | "2023".yr. |
| 77 | 75 or 76 [All other RCTs from MEDLINE should be identified in CENTRAL] |
| 78 | 74 and 77 |

**Embase**

| 1 | psychosocial intervention/ |
| --- | --- |
| 2 | exp *mental disease/ |
| 3 | exp *mental health/ |
| 4 | *mentally disabled person/ |
| 5 | *child development/ |
| 6 | *automutilation/ |
| 7 | (mental health* or mental* ill* or mental* disorder* or mental* well*).ti,ab,kf. |
| 8 | ((substance or alcohol or opioid or morphine or marijuana or heroin or cocaine) adj2 (disorder* or illness* or dependence or abuse or misuse or "use")).ti,ab,kf. |
| 9 | (depressi* adj2 (sign* or symptom* or disorder*)).ti,ab,kf. |
| 10 | (depress* adj3 (acute or clinical* or diagnos* or disorder* or major or unipolar or illness or scale* or score* or adult* or child* or adolesc* or teen* or youth* or elder* or late* life* or patient* or participant* or people or inpatient* or in-patient* or outpatient* or out-patient*)).ti,ab,kf. |
| 11 | ((depress* or distress*) adj3 (postnatal* or post natal* or maternal*)).ti,ab,kf. |
| 12 | (depression or anxiety or alzheimer* or schizoaffective or mania or manic or borderline personality or (stress adj2 disorder*) or adjustment disorder* or (psychological adj1 trauma*) or schizophrenia or psychoses or psychosis or stress syndrome* or distress syndrome* or combat disorder* or war disorder* or ptsd or dementia).ti,ab,kf. |
| 13 | ((post-trauma* or posttrauma*) adj3 (stress* or disorder*)).ti,ab,kf. |
| 14 | (psychological trauma or psychotrauma*).ti,ab,kf. |
| 15 | (alcoholism or alcoholic* or drug addict* or drug abus* or drug misuse or drug user*).ti,ab,kf. |
| 16 | ((learning or mental* or intellectual) adj (disabled or disabilit* or disorder* or difficult*)).ti,ab,kf. |
| 17 | ((dissociative adj3 (disorder* or reaction*)) or dissociation).ti,ab,kf. |
| 18 | ((bipolar or behavio?ral or obsessive or panic or mood or delusional) adj2 (disorder* or illness* or disease*)).ti,ab,kf. |
| 19 | (trichotillomani* or OCD or obsess*-compulsi* or GAD or stress reaction* or acute stress or neuros#s or neurotic).ti,ab,kf. |
| 20 | (affective* adj (disorder* or disease* or illness* or symptom*)).ti,ab,kf. |
| 21 | ((mental or psychological or emotional or psycho-social or psychosocial) adj (stress* or distress*)).ti,ab,kf. |
| 22 | ((sub-syndrom* or sub-threshold or sub-clinical or subsyndrom* or subthreshold or subclinical or minor or brief) adj (symptom* or disorder* or condition* or depress* or anxiety)).ti,ab,kf. |
| 23 | (mental relapse or fatigue or somatic symptom* or worry or worries or panic or low mood* or mood problem*).ti,ab,kf. |
| 24 | (anxiety disorder* or agoraphobi* or general* anxi* or separation anxiety or neurocirculatory asthenia or neurotic disorder* or social phobi* or self-harm* or self-injur* or suicid*).ti,ab,kf. |
| 25 | (slow* adj (thought* or think*)).ti,ab,kf. |
| 26 | (mental* adj develop*).ti,ab,kf. |
| 27 | or/2-26 |
| 28 | exp *primary health care/ |
| 29 | *general practitioner/ |
| 30 | *general practice/ |
| 31 | exp *social support/ |
| 32 | exp *health auxiliary/ |
| 33 | exp *community care/ |
| 34 | exp *paramedical personnel/ |
| 35 | *family nursing/ |
| 36 | *rural health nursing/ |
| 37 | exp *school/ |
| 38 | exp *school health service/ |
| 39 | *rural health care/ |
| 40 | *rural health/ |
| 41 | *rural population/ |
| 42 | (primary adj5 (care or health*)).ti,ab,kf. |
| 43 | (family practi* or family doctor* or family physician* or gp* or general practi*).ti,ab,kf. |
| 44 | (school* or teacher* or rural* or community).ti,ab,kf. |
| 45 | (non-specialist* or nonspecialist* or social worker* or trainer*).ti,ab,kf. |
| 46 | (psycho-social or psychosocial).ti,ab,kf. |
| 47 | (caregiver* or care giver* or layperson*).ti,ab,kf. |
| 48 | (paraprofessional* or para-professional* or auxiliar* or paramedics or (allied health* adj (professional* or person* or staff or worker*)) or non-physician* or non-clinician*).ti,ab,kf. |
| 49 | (lay adj2 (heal* or person* or counsellor* or counselor* or worker* or therapist*)).ti,ab,kf. |
| 50 | (midwife or midwive* or pharmacist* or pharmacy or pharmacies or practice nurs* or district nurs* or health visitor*).ti,ab,kf. |
| 51 | (psychological intervention* or task shift* or shifting tasks or task sharing or sharing tasks or (delegate adj2 task*)).ti,ab,kf. |
| 52 | or/28-51 |
| 53 | 27 and 52 |
| 54 | random*.ti,ab. |
| 55 | factorial*.ti,ab. |
| 56 | (crossover* or cross over*).ti,ab. |
| 57 | ((doubl* or singl*) adj blind*).ti,ab. |
| 58 | (assign* or allocat* or volunteer* or placebo*).ti,ab. |
| 59 | crossover procedure/ |
| 60 | single blind procedure/ |
| 61 | randomized controlled trial/ |
| 62 | double blind procedure/ |
| 63 | or/54-62 |
| 64 | exp animal/ not human/ |
| 65 | 63 not 64 |
| 66 | (afghan* or africa* or albania* or algeria* or angola* or antigua* or barbuda* or argentin* or armenia* or aruba* or azerbaijan* or bahrain* or bangladesh* or bengal* or bangal* or barbados* or barbadian* or bajan or bajans or belarus* or belorus* or byelarus* or byelorus* or belize* or benin* or dahomey or bhutan* or bolivia* or bosnia* or herzegovin* or botswan* or batswan* or bechuanaland* or brazil* or brasil* or bulgaria* or burkina* or burkinese* or upper volta* or burundi* or urundi* or cabo verde* or cape verde* or cambodia* or kampuchea* or khmer* or cameroon* or cameroun* or ubangi shari* or chad* or chile* or china* or chinese or colombia* or comoro* or comore* or comorian* or mayotte* or congo* or zaire* or costa rica* or "cote d'ivoir*" or "cote d' ivoir*" or cote divoir* or cote d ivoir* or ivory coast* or ivorian* or croatia* or cuba or cuban or cubans or "cuba's" or cyprus* or cypriot* or czech* or djibouti* or french somaliland* or dominica* or ecuador* or egypt* or united arab republic* or el salvador* or salvadoran* or guinea* or equatoguinea* or eritrea* or estonia* or eswatini* or swaziland* or swazi* or swati* or ethiopia* or fiji* or gabon* or gabonese* or gabonaise* or gambia* or ((georgia or georgian or georgians) not (atlanta or california or florida)) or ghana* or gibraltar* or greece* or greek* or grecian* or grenada* or grenadian* or guam* or guatemala* or guyana* or guiana* or guyanese* or haiti* or hispaniola* or hondura* or hungary* or hungarian* or india* or indonesia* or iran* or iraq* or isle of man* or jamaica* or jordan* or kazakh* or kenya* or karabati* or korea* or kosovo* or kosova* or kyrgyz* or kirgiz* or kirghiz* or laos or lao or laotian* or latvia* or lebanon* or lebanese* or lesotho* or lesothan* or lesothonian* or basutoland* or mosotho* or basotho* or liberia* or libya* or jamahiriya* or lithuania* or macedonia* or madagasca* or malagasy* or malawi* or nyasaland* or malaysia* or malay* federation or maldives* or maldivian* or indian ocean or mali or malian* or "mali's" or malta or maltese* or "malta's" or micronesia* or marshallese* or kiribati* or marshall island* or nauru or nauran or nauruans or "naurian's" or mariana or marianas or palau or paluan* or tuvalu* or mauritania* or mauritan* or mauritius* or mexico* or mexican* or moldova* or moldovia* or mongol* or montenegr* or morocco* or moroccan* or ifni or mozambique* or mozambican* or myanmar* or burma* or burmese or namibia* or nepal* or new caledonia* or netherlands antill* or nicaragua* or niger* or oman or omani or omanis or "oman's" or pakistan* or palestin* or gaza* or west bank* or panama* or paraguay* or peru or peruvian* or "peru's" or philippine* or philipine* or phillipine* or phillippine* or filipino* or filipina* or poland* or polish or pole or poles or portugal* or portuguese or puerto ric* or romania* or russia* or ussr* or soviet* or rwanda* or rwandese or ruanda* or ruandese or samoa* or navigator island* or pacific island* or polynesia* or sao tome* or santomean* or saudi arabia* or saudi or saudis or senegal* or serbia* or seychell* or sierra leone* or slovak* or sloven* or melanesia* or solomon island* or norfolk island* or somali* or sri lanka* or ceylon* or saint kitts or st kitts or kittian* or nevisian* or saint lucia* or st lucia* or saint vincent* or st vincent* or vincentian* or grenadine* or sudan* or surinam* or syria* or tajik* or tadjik* or tadzhik* or tanzania* or tanganyika* or thai* or timor leste* or east timor* or timorese* or togo or togoles* or "togo's" or tonga* or trinidad* or tobago* or tunisia* or turkiy* or turkey* or turk or turks or turkish or turkmen* or uganda* or ukrain* or uruguay* or uzbek* or vanuatu* or new hebrides* or venezuela* or vietnam* or viet nam* or yemen* or yugoslav* or zambia* or zimbabwe* or rhodesia* or arab* countr* or middle east* or global south or sahara* or subsahara* or magreb* or maghrib* or west indies* or caribbean* or central america* or latin america* or south america* or asia central or central asia* or asia northern or north asia* or northern asia* or asia southeastern or southeastern asia* or south eastern asia* or southeast asia* or south east asia* or asia western or west asia* or western asia* or europe eastern or east europe* or eastern europe* or developing countr* or developing nation* or developing population* or developing world or less developed countr* or less developed nation* or less developed world or lesser developed countr* or lesser developed nation* or lesser developed world or under developed countr* or under developed nation* or under developed world or underdeveloped countr* or underdeveloped nation* or underdeveloped world or middle income countr* or middle income nation* or middle income population* or low income countr* or low income nation* or low income population* or lower income countr* or lower income nation* or lower income population* or underserved countr* or underserved nation* or underserved population* or under served population* or under served nation* or under served population* or deprived countr* or deprived population* or high burden countr* or high burden nation* or countdown countr* or countdown nation* or poor countr* or poor nation* or poor population* or poor world or poorer countr* or poorer nation* or poorer population* or poorer world or developing econom* or less developed econom* or underdeveloped econom* or under developed econom* or middle income econom* or low income econom* or lower income econom* or low gdp or low gnp or low gross domestic or low gross national or lower gdp or lower gnp or lower gross domestic or lower gross national or lmic or lmics or third world or lami countr* or transitional countr* or emerging econom* or emerging nation*).ti,ab,hw,kf. |
| 67 | (1 or 53) and 65 and 66 |
| 68 | limit 67 to embase |
| 69 | 2023*.em. |
| 70 | "2023".yr. |
| 71 | 69 or 70 [Limited to last months and year to identify Embase records not yet transferred to CENTRAL, Cochrane Library. RCTs from Embase, added to the database (em) |
| 72 | 68 and 71 |

**APA PsycINFO**

| 1 | exp mental disorders/ |
| --- | --- |
| 2 | exp mental health/ |
| 3 | "depression (emotion)"/ |
| 4 | childhood development/ |
| 5 | exp intellectual development disorder/ |
| 6 | exp learning disorders/ |
| 7 | exp learning disabilities/ |
| 8 | exp self-injurious behavior/ |
| 9 | exp suicide/ |
| 10 | (mental health* or mental* ill* or mental* disorder* or mental* well*).ti,ab. |
| 11 | ((substance or alcohol or opioid or morphine or marijuana or heroin or cocaine) adj2 (disorder* or illness* or dependence or abuse or misuse or "use")).ti,ab. |
| 12 | (depressi* adj2 (sign* or symptom* or disorder*)).ti,ab. |
| 13 | (depress* adj3 (acute or clinical* or diagnos* or disorder* or major or unipolar or illness or scale* or score* or adult* or child* or adolesc* or teen* or youth* or elder* or late* life* or patient* or participant* or people or inpatient* or in-patient* or outpatient* or out-patient*)).ti,ab. |
| 14 | ((depress* or distress*) adj3 (postnatal* or post natal* or maternal*)).ti,ab. |
| 15 | (depression or anxiety or alzheimer* or schizoaffective or mania or manic or borderline personality or (stress adj2 disorder*) or adjustment disorder* or (psychological adj1 trauma*) or schizophrenia or psychoses or psychosis or stress syndrome* or distress syndrome* or combat disorder* or war disorder* or ptsd or dementia).ti,ab. |
| 16 | ((post-trauma* or posttrauma*) adj3 (stress* or disorder*)).ti,ab. |
| 17 | (psychological trauma or psychotrauma*).ti,ab. |
| 18 | (alcoholism or alcoholic* or drug addict* or drug abus* or drug misuse or drug user*).ti,ab. |
| 19 | ((learning or mental* or intellectual) adj (disabled or disabilit* or disorder* or difficult*)).ti,ab. |
| 20 | ((dissociative adj3 (disorder* or reaction*)) or dissociation).ti,ab. |
| 21 | ((bipolar or behavio?ral or obsessive or panic or mood or delusional) adj2 (disorder* or illness* or disease*)).ti,ab. |
| 22 | (trichotillomani* or OCD or obsess*-compulsi* or GAD or stress reaction* or acute stress or neuros#s or neurotic).ti,ab. |
| 23 | (affective* adj (disorder* or disease* or illness* or symptom*)).ti,ab. |
| 24 | ((mental or psychological or emotional or psycho-social or psychosocial) adj (stress* or distress*)).ti,ab. |
| 25 | ((sub-syndrom* or sub-threshold or sub-clinical or subsyndrom* or subthreshold or subclinical or minor or brief) adj (symptom* or disorder* or condition* or depress* or anxiety)).ti,ab. |
| 26 | (mental relapse or fatigue or somatic symptom* or worry or worries or panic or low mood* or mood problem*).ti,ab. |
| 27 | (anxiety disorder* or agoraphobi* or general* anxi* or separation anxiety or neurocirculatory asthenia or neurotic disorder* or social phobi* or self-harm* or self-injur* or suicid*).ti,ab. |
| 28 | (slow* adj (thought* or think*)).ti,ab. |
| 29 | (mental* adj develop*).ti,ab. |
| 30 | or/1-29 |
| 31 | primary health care/ |
| 32 | family physicians/ |
| 33 | general practitioners/ |
| 34 | exp allied health personnel/ |
| 35 | social support/ |
| 36 | exp community health/ |
| 37 | exp community services/ |
| 38 | exp school based intervention/ |
| 39 | exp schools/ |
| 40 | school nurses/ |
| 41 | rural environments/ |
| 42 | (primary adj5 (care or health*)).ti,ab. |
| 43 | (family practi* or family doctor* or family physician* or gp* or general practi*).ti,ab. |
| 44 | (school* or teacher* or rural* or community).ti,ab. |
| 45 | (non-specialist* or nonspecialist* or social worker* or trainer*).ti,ab. |
| 46 | (psycho-social or psychosocial).ti,ab. |
| 47 | (caregiver* or care giver* or layperson*).ti,ab. |
| 48 | (lay adj2 (heal* or person* or counsellor* or counselor* or worker* or therapist*)).ti,ab. |
| 49 | (paraprofessional* or para-professional* or auxiliar* or paramedics or (allied health* adj (professional* or person* or staff or worker*)) or non-physician* or non-clinician*).ti,ab. |
| 50 | (midwife or midwive* or pharmacist* or pharmacy or pharmacies or practice nurs* or district nurs* or health visitor*).ti,ab. |
| 51 | (psychological intervention* or task shift* or shifting tasks or task sharing or sharing tasks or (delegate adj2 task*)).ti,ab. |
| 52 | or/31-51 |
| 53 | 30 and 52 |
| 54 | exp clinical trial/ |
| 55 | random*.ti,ab. |
| 56 | ((clinical or control*) adj3 trial*).ti,ab. |
| 57 | ((singl* or doubl* or trebl* or tripl*) adj5 (blind* or mask*)).ti,ab. |
| 58 | (volunteer* or control group or controls).ti,ab. |
| 59 | placebo/ or placebo*.ti,ab. |
| 60 | or/54-59 [Methods filter] |
| 61 | 53 and 60 |
| 62 | (afghan* or africa* or albania* or algeria* or angola* or antigua* or barbuda* or argentin* or armenia* or aruba* or azerbaijan* or bahrain* or bangladesh* or bengal* or bangal* or barbados* or barbadian* or bajan or bajans or belarus* or belorus* or byelarus* or byelorus* or belize* or benin* or dahomey or bhutan* or bolivia* or bosnia* or herzegovin* or botswan* or batswan* or bechuanaland* or brazil* or brasil* or bulgaria* or burkina* or burkinese* or upper volta* or burundi* or urundi* or cabo verde* or cape verde* or cambodia* or kampuchea* or khmer* or cameroon* or cameroun* or ubangi shari* or chad* or chile* or china* or chinese or colombia* or comoro* or comore* or comorian* or mayotte* or congo* or zaire* or costa rica* or "cote d'ivoir*" or "cote d' ivoir*" or cote divoir* or cote d ivoir* or ivory coast* or ivorian* or croatia* or cuba or cuban or cubans or "cuba's" or cyprus* or cypriot* or czech* or djibouti* or french somaliland* or dominica* or ecuador* or egypt* or united arab republic* or el salvador* or salvadoran* or guinea* or equatoguinea* or eritrea* or estonia* or eswatini* or swaziland* or swazi* or swati* or ethiopia* or fiji* or gabon* or gabonese* or gabonaise* or gambia* or ((georgia or georgian or georgians) not (atlanta or california or florida)) or ghana* or gibraltar* or greece* or greek* or grecian* or grenada* or grenadian* or guam* or guatemala* or guyana* or guiana* or guyanese* or haiti* or hispaniola* or hondura* or hungary* or hungarian* or india* or indonesia* or iran* or iraq* or isle of man* or jamaica* or jordan* or kazakh* or kenya* or karabati* or korea* or kosovo* or kosova* or kyrgyz* or kirgiz* or kirghiz* or laos or lao or laotian* or latvia* or lebanon* or lebanese* or lesotho* or lesothan* or lesothonian* or basutoland* or mosotho* or basotho* or liberia* or libya* or jamahiriya* or lithuania* or macedonia* or madagasca* or malagasy* or malawi* or nyasaland* or malaysia* or malay* federation or maldives* or maldivian* or indian ocean or mali or malian* or "mali's" or malta or maltese* or "malta's" or micronesia* or marshallese* or kiribati* or marshall island* or nauru or nauran or nauruans or "naurian's" or mariana or marianas or palau or paluan* or tuvalu* or mauritania* or mauritan* or mauritius* or mexico* or mexican* or moldova* or moldovia* or mongol* or montenegr* or morocco* or moroccan* or ifni or mozambique* or mozambican* or myanmar* or burma* or burmese or namibia* or nepal* or new caledonia* or netherlands antill* or nicaragua* or niger* or oman or omani or omanis or "oman's" or pakistan* or palestin* or gaza* or west bank* or panama* or paraguay* or peru or peruvian* or "peru's" or philippine* or philipine* or phillipine* or phillippine* or filipino* or filipina* or poland* or polish or pole or poles or portugal* or portuguese or puerto ric* or romania* or russia* or ussr* or soviet* or rwanda* or rwandese or ruanda* or ruandese or samoa* or navigator island* or pacific island* or polynesia* or sao tome* or santomean* or saudi arabia* or saudi or saudis or senegal* or serbia* or seychell* or sierra leone* or slovak* or sloven* or melanesia* or solomon island* or norfolk island* or somali* or sri lanka* or ceylon* or saint kitts or st kitts or kittian* or nevisian* or saint lucia* or st lucia* or saint vincent* or st vincent* or vincentian* or grenadine* or sudan* or surinam* or syria* or tajik* or tadjik* or tadzhik* or tanzania* or tanganyika* or thai* or timor leste* or east timor* or timorese* or togo or togoles* or "togo's" or tonga* or trinidad* or tobago* or tunisia* or turkiy* or turkey* or turk or turks or turkish or turkmen* or uganda* or ukrain* or uruguay* or uzbek* or vanuatu* or new hebrides* or venezuela* or vietnam* or viet nam* or yemen* or yugoslav* or zambia* or zimbabwe* or rhodesia* or arab* countr* or middle east* or global south or sahara* or subsahara* or magreb* or maghrib* or west indies* or caribbean* or central america* or latin america* or south america* or asia central or central asia* or asia northern or north asia* or northern asia* or asia southeastern or southeastern asia* or south eastern asia* or southeast asia* or south east asia* or asia western or west asia* or western asia* or europe eastern or east europe* or eastern europe* or developing countr* or developing nation* or developing population* or developing world or less developed countr* or less developed nation* or less developed world or lesser developed countr* or lesser developed nation* or lesser developed world or under developed countr* or under developed nation* or under developed world or underdeveloped countr* or underdeveloped nation* or underdeveloped world or middle income countr* or middle income nation* or middle income population* or low income countr* or low income nation* or low income population* or lower income countr* or lower income nation* or lower income population* or underserved countr* or underserved nation* or underserved population* or under served population* or under served nation* or under served population* or deprived countr* or deprived population* or high burden countr* or high burden nation* or countdown countr* or countdown nation* or poor countr* or poor nation* or poor population* or poor world or poorer countr* or poorer nation* or poorer population* or poorer world or developing econom* or less developed econom* or underdeveloped econom* or under developed econom* or middle income econom* or low income econom* or lower income econom* or low gdp or low gnp or low gross domestic or low gross national or lower gdp or lower gnp or lower gross domestic or lower gross national or lmic or lmics or third world or lami countr* or transitional countr* or emerging econom* or emerging nation*).hw,ti,ab. |
| 63 | 61 and 62 |
| 64 | (202111* or 202112* or 2022* or 2023*).up,yr. |
| 65 | 63 and 64 |

**CINAHL**

| S61 | S59 AND S60 |
| --- | --- |
| S60 | EM 202111 |
| S59 | S52 AND S57 |
|  | [Limiters - Exclude MEDLINE records] |
| S58 | S52 AND S57 |
| S57 | S53 OR S54 OR S55 OR S56 |
| S56 | AB afghan* OR africa* OR albania* OR algeria* OR angola* OR antigua* OR barbuda* OR argentin* OR armenia* OR aruba* OR azerbaijan* OR bahrain* OR bangladesh* OR bengal* OR bangal* OR barbados* OR barbadian* OR bajan OR bajans OR belarus* OR belorus* OR byelarus* OR byelorus* OR belize* OR benin* OR dahomey OR bhutan* OR bolivia* OR bosnia* OR herzegovin* OR botswan* OR batswan* OR bechuanaland* OR brazil* OR brasil* OR bulgaria* OR burkina* OR burkinese* OR upper-volta* OR burundi* OR urundi* OR cabo-verde* OR cape-verde* OR cambodia* OR kampuchea* OR khmer* OR cameroon* OR cameroun* OR ubangi-shari* OR chad* OR chile* OR china* OR chinese OR colombia* OR comoro* OR comore* OR comorian* OR mayotte* OR congo* OR zaire* OR costa-rica* OR "cote d'ivoir*" OR "cote d' ivoir*" OR cote divoir* OR cote d ivoir* OR ivory-coast* OR ivorian* OR croatia* OR cuba OR cuban OR cubans OR "cuba's" OR cyprus* OR cypriot* OR czech* OR djibouti* OR french-somaliland* OR dominica* OR ecuador* OR egypt* OR united-arab-republic* OR el-salvador* OR salvadoran* OR guinea* OR equatoguinea* OR eritrea* OR estonia* OR eswatini* OR swaziland* OR swazi* OR swati* OR ethiopia* OR fiji* OR gabon* OR gabonese* OR gabonaise* OR gambia* OR georgia OR georgian OR georgians OR ghana* OR gibraltar* OR greece* OR greek* OR grecian* OR grenada* OR grenadian* OR guam* OR guatemala* OR guyana* OR guiana* OR guyanese* OR haiti* OR hispaniola* OR hondura* OR hungary* OR hungarian* OR india* OR indonesia* OR iran* OR iraq* OR isle-of-man* OR jamaica* OR jordan* OR kazakh* OR kenya* OR karabati* OR korea* OR kosovo* OR kosova* OR kyrgyz* OR kirgiz* OR kirghiz* OR laos OR lao OR laotian* OR latvia* OR lebanon* OR lebanese* OR lesotho* OR lesothan* OR lesothonian* OR basutoland* OR mosotho* OR basotho* OR liberia* OR libya* OR jamahiriya* OR lithuania* OR macedonia* OR madagasca* OR malagasy* OR malawi* OR nyasaland* OR malaysia* OR malay*-federation OR maldives* OR maldivian* OR indian-ocean OR mali OR malian* OR "mali's" OR malta OR maltese* OR "malta's" OR micronesia* OR marshallese* OR kiribati* OR marshall-island* OR nauru OR nauran OR nauruans OR "naurian's" OR mariana OR marianas OR palau OR paluan* OR tuvalu* OR mauritania* OR mauritan* OR mauritius* OR mexico* OR mexican* OR moldova* OR moldovia* OR mongol* OR montenegr* OR morocco* OR moroccan* OR ifni OR mozambique* OR mozambican* OR myanmar* OR burma* OR burmese OR namibia* OR nepal* OR new-caledonia* OR netherlands-antill* OR nicaragua* OR niger* OR oman OR omani OR omanis OR "oman's" OR pakistan* OR palestin* OR gaza* OR west-bank* OR panama* OR paraguay* OR peru OR peruvian* OR "peru's" OR philippine* OR philipine* OR phillipine* OR phillippine* OR filipino* OR filipina* OR poland* OR polish OR pole OR poles OR portugal* OR portuguese OR puerto-ric* OR romania* OR russia* OR ussr* OR soviet* OR rwanda* OR rwandese OR ruanda* OR ruandese OR samoa* OR navigator-island* OR pacific-island* OR polynesia* OR sao-tome* OR santomean* OR saudi-arabia* OR saudi OR saudis OR senegal* OR serbia* OR seychell* OR sierra-leone* OR slovak* OR sloven* OR melanesia* OR solomon-island* OR norfolk-island* OR somali* OR sri-lanka* OR ceylon* OR saint-kitts OR st-kitts OR kittian* OR nevisian* OR saint-lucia* OR st-lucia* OR saint-vincent* OR st-vincent* OR vincentian* OR grenadine* OR sudan* OR surinam* OR syria* OR tajik* OR tadjik* OR tadzhik* OR tanzania* OR tanganyika* OR thai* OR timor-leste* OR east-timor* OR timorese* OR togo OR togoles* OR "togo's" OR tonga* OR trinidad* OR tobago* OR tunisia* OR turkiy* OR turkey* OR turk OR turks OR turkish OR turkmen* OR uganda* OR ukrain* OR uruguay* OR uzbek* OR vanuatu* OR new-hebrides* OR venezuela* OR vietnam* OR viet-nam* OR yemen* OR yugoslav* OR zambia* OR zimbabwe* OR rhodesia* OR arab*-countr* OR middle-east* OR global-south OR sahara* OR subsahara* OR magreb* OR maghrib* OR west-indies* OR caribbean* OR central-america* OR latin-america* OR south-america* OR asia-central OR central-asia* OR asia-northern OR north-asia* OR northern-asia* OR asia-southeastern OR southeastern-asia* OR south-eastern-asia* OR southeast-asia* OR south-east-asia* OR asia-western OR west-asia* OR western-asia* OR europe-eastern OR east-europe* OR eastern-europe* OR developing-countr* OR developing-nation* OR developing-population* OR developing-world OR less-developed-countr* OR less-developed-nation* OR less-developed-world OR lesser-developed-countr* OR lesser-developed-nation* OR lesser-developed-world OR under-developed-countr* OR under-developed-nation* OR under-developed-world OR underdeveloped-countr* OR underdeveloped-nation* OR underdeveloped-world OR middle-income-countr* OR middle-income-nation* OR middle-income-population* OR low-income-countr* OR low-income-nation* OR low-income-population* OR lower-income-countr* OR lower-income-nation* OR lower-income-population* OR underserved-countr* OR underserved-nation* OR underserved-population* OR under-served-population* OR under-served-nation* OR under-served-population* OR deprived-countr* OR deprived-population* OR high-burden-countr* OR high-burden-nation* OR countdown-countr* OR countdown-nation* OR poor-countr* OR poor-nation* OR poor-population* OR poor-world OR poorer-countr* OR poorer-nation* OR poorer-population* OR poorer-world OR developing-econom* OR less-developed-econom* OR underdeveloped-econom* OR under-developed-econom* OR middle-income-econom* OR low-income-econom* OR lower-income-econom* OR low-gdp OR low-gnp OR low-gross-domestic OR low-gross-national OR lower-gdp OR lower-gnp OR lower-gross-domestic OR lower-gross-national OR lmic OR lmics OR third-world OR lami-countr* OR transitional-countr* OR emerging-econom* OR emerging-nation* |
| S55 | TI afghan* OR africa* OR albania* OR algeria* OR angola* OR antigua* OR barbuda* OR argentin* OR armenia* OR aruba* OR azerbaijan* OR bahrain* OR bangladesh* OR bengal* OR bangal* OR barbados* OR barbadian* OR bajan OR bajans OR belarus* OR belorus* OR byelarus* OR byelorus* OR belize* OR benin* OR dahomey OR bhutan* OR bolivia* OR bosnia* OR herzegovin* OR botswan* OR batswan* OR bechuanaland* OR brazil* OR brasil* OR bulgaria* OR burkina* OR burkinese* OR upper-volta* OR burundi* OR urundi* OR cabo-verde* OR cape-verde* OR cambodia* OR kampuchea* OR khmer* OR cameroon* OR cameroun* OR ubangi-shari* OR chad* OR chile* OR china* OR chinese OR colombia* OR comoro* OR comore* OR comorian* OR mayotte* OR congo* OR zaire* OR costa-rica* OR "cote d'ivoir*" OR "cote d' ivoir*" OR cote divoir* OR cote d ivoir* OR ivory-coast* OR ivorian* OR croatia* OR cuba OR cuban OR cubans OR "cuba's" OR cyprus* OR cypriot* OR czech* OR djibouti* OR french-somaliland* OR dominica* OR ecuador* OR egypt* OR united-arab-republic* OR el-salvador* OR salvadoran* OR guinea* OR equatoguinea* OR eritrea* OR estonia* OR eswatini* OR swaziland* OR swazi* OR swati* OR ethiopia* OR fiji* OR gabon* OR gabonese* OR gabonaise* OR gambia* OR georgia OR georgian OR georgians OR ghana* OR gibraltar* OR greece* OR greek* OR grecian* OR grenada* OR grenadian* OR guam* OR guatemala* OR guyana* OR guiana* OR guyanese* OR haiti* OR hispaniola* OR hondura* OR hungary* OR hungarian* OR india* OR indonesia* OR iran* OR iraq* OR isle-of-man* OR jamaica* OR jordan* OR kazakh* OR kenya* OR karabati* OR korea* OR kosovo* OR kosova* OR kyrgyz* OR kirgiz* OR kirghiz* OR laos OR lao OR laotian* OR latvia* OR lebanon* OR lebanese* OR lesotho* OR lesothan* OR lesothonian* OR basutoland* OR mosotho* OR basotho* OR liberia* OR libya* OR jamahiriya* OR lithuania* OR macedonia* OR madagasca* OR malagasy* OR malawi* OR nyasaland* OR malaysia* OR malay*-federation OR maldives* OR maldivian* OR indian-ocean OR mali OR malian* OR "mali's" OR malta OR maltese* OR "malta's" OR micronesia* OR marshallese* OR kiribati* OR marshall-island* OR nauru OR nauran OR nauruans OR "naurian's" OR mariana OR marianas OR palau OR paluan* OR tuvalu* OR mauritania* OR mauritan* OR mauritius* OR mexico* OR mexican* OR moldova* OR moldovia* OR mongol* OR montenegr* OR morocco* OR moroccan* OR ifni OR mozambique* OR mozambican* OR myanmar* OR burma* OR burmese OR namibia* OR nepal* OR new-caledonia* OR netherlands-antill* OR nicaragua* OR niger* OR oman OR omani OR omanis OR "oman's" OR pakistan* OR palestin* OR gaza* OR west-bank* OR panama* OR paraguay* OR peru OR peruvian* OR "peru's" OR philippine* OR philipine* OR phillipine* OR phillippine* OR filipino* OR filipina* OR poland* OR polish OR pole OR poles OR portugal* OR portuguese OR puerto-ric* OR romania* OR russia* OR ussr* OR soviet* OR rwanda* OR rwandese OR ruanda* OR ruandese OR samoa* OR navigator-island* OR pacific-island* OR polynesia* OR sao-tome* OR santomean* OR saudi-arabia* OR saudi OR saudis OR senegal* OR serbia* OR seychell* OR sierra-leone* OR slovak* OR sloven* OR melanesia* OR solomon-island* OR norfolk-island* OR somali* OR sri-lanka* OR ceylon* OR saint-kitts OR st-kitts OR kittian* OR nevisian* OR saint-lucia* OR st-lucia* OR saint-vincent* OR st-vincent* OR vincentian* OR grenadine* OR sudan* OR surinam* OR syria* OR tajik* OR tadjik* OR tadzhik* OR tanzania* OR tanganyika* OR thai* OR timor-leste* OR east-timor* OR timorese* OR togo OR togoles* OR "togo's" OR tonga* OR trinidad* OR tobago* OR tunisia* OR turkiy* OR turkey* OR turk OR turks OR turkish OR turkmen* OR uganda* OR ukrain* OR uruguay* OR uzbek* OR vanuatu* OR new-hebrides* OR venezuela* OR vietnam* OR viet-nam* OR yemen* OR yugoslav* OR zambia* OR zimbabwe* OR rhodesia* OR arab*-countr* OR middle-east* OR global-south OR sahara* OR subsahara* OR magreb* OR maghrib* OR west-indies* OR caribbean* OR central-america* OR latin-america* OR south-america* OR asia-central OR central-asia* OR asia-northern OR north-asia* OR northern-asia* OR asia-southeastern OR southeastern-asia* OR south-eastern-asia* OR southeast-asia* OR south-east-asia* OR asia-western OR west-asia* OR western-asia* OR europe-eastern OR east-europe* OR eastern-europe* OR developing-countr* OR developing-nation* OR developing-population* OR developing-world OR less-developed-countr* OR less-developed-nation* OR less-developed-world OR lesser-developed-countr* OR lesser-developed-nation* OR lesser-developed-world OR under-developed-countr* OR under-developed-nation* OR under-developed-world OR underdeveloped-countr* OR underdeveloped-nation* OR underdeveloped-world OR middle-income-countr* OR middle-income-nation* OR middle-income-population* OR low-income-countr* OR low-income-nation* OR low-income-population* OR lower-income-countr* OR lower-income-nation* OR lower-income-population* OR underserved-countr* OR underserved-nation* OR underserved-population* OR under-served-population* OR under-served-nation* OR under-served-population* OR deprived-countr* OR deprived-population* OR high-burden-countr* OR high-burden-nation* OR countdown-countr* OR countdown-nation* OR poor-countr* OR poor-nation* OR poor-population* OR poor-world OR poorer-countr* OR poorer-nation* OR poorer-population* OR poorer-world OR developing-econom* OR less-developed-econom* OR underdeveloped-econom* OR under-developed-econom* OR middle-income-econom* OR low-income-econom* OR lower-income-econom* OR low-gdp OR low-gnp OR low-gross-domestic OR low-gross-national OR lower-gdp OR lower-gnp OR lower-gross-domestic OR lower-gross-national OR lmic OR lmics OR third-world OR lami-countr* OR transitional-countr* OR emerging-econom* OR emerging-nation* |
| S54 | MW afghan* OR africa* OR albania* OR algeria* OR angola* OR antigua* OR barbuda* OR argentin* OR armenia* OR aruba* OR azerbaijan* OR bahrain* OR bangladesh* OR bengal* OR bangal* OR barbados* OR barbadian* OR bajan OR bajans OR belarus* OR belorus* OR byelarus* OR byelorus* OR belize* OR benin* OR dahomey OR bhutan* OR bolivia* OR bosnia* OR herzegovin* OR botswan* OR batswan* OR bechuanaland* OR brazil* OR brasil* OR bulgaria* OR burkina* OR burkinese* OR upper-volta* OR burundi* OR urundi* OR cabo-verde* OR cape-verde* OR cambodia* OR kampuchea* OR khmer* OR cameroon* OR cameroun* OR ubangi-shari* OR chad* OR chile* OR china* OR chinese OR colombia* OR comoro* OR comore* OR comorian* OR mayotte* OR congo* OR zaire* OR costa-rica* OR "cote d'ivoir*" OR "cote d' ivoir*" OR cote divoir* OR cote d ivoir* OR ivory-coast* OR ivorian* OR croatia* OR cuba OR cuban OR cubans OR "cuba's" OR cyprus* OR cypriot* OR czech* OR djibouti* OR french-somaliland* OR dominica* OR ecuador* OR egypt* OR united-arab-republic* OR el-salvador* OR salvadoran* OR guinea* OR equatoguinea* OR eritrea* OR estonia* OR eswatini* OR swaziland* OR swazi* OR swati* OR ethiopia* OR fiji* OR gabon* OR gabonese* OR gabonaise* OR gambia* OR georgia OR georgian OR georgians OR ghana* OR gibraltar* OR greece* OR greek* OR grecian* OR grenada* OR grenadian* OR guam* OR guatemala* OR guyana* OR guiana* OR guyanese* OR haiti* OR hispaniola* OR hondura* OR hungary* OR hungarian* OR india* OR indonesia* OR iran* OR iraq* OR isle-of-man* OR jamaica* OR jordan* OR kazakh* OR kenya* OR karabati* OR korea* OR kosovo* OR kosova* OR kyrgyz* OR kirgiz* OR kirghiz* OR laos OR lao OR laotian* OR latvia* OR lebanon* OR lebanese* OR lesotho* OR lesothan* OR lesothonian* OR basutoland* OR mosotho* OR basotho* OR liberia* OR libya* OR jamahiriya* OR lithuania* OR macedonia* OR madagasca* OR malagasy* OR malawi* OR nyasaland* OR malaysia* OR malay*-federation OR maldives* OR maldivian* OR indian-ocean OR mali OR malian* OR "mali's" OR malta OR maltese* OR "malta's" OR micronesia* OR marshallese* OR kiribati* OR marshall-island* OR nauru OR nauran OR nauruans OR "naurian's" OR mariana OR marianas OR palau OR paluan* OR tuvalu* OR mauritania* OR mauritan* OR mauritius* OR mexico* OR mexican* OR moldova* OR moldovia* OR mongol* OR montenegr* OR morocco* OR moroccan* OR ifni OR mozambique* OR mozambican* OR myanmar* OR burma* OR burmese OR namibia* OR nepal* OR new-caledonia* OR netherlands-antill* OR nicaragua* OR niger* OR oman OR omani OR omanis OR "oman's" OR pakistan* OR palestin* OR gaza* OR west-bank* OR panama* OR paraguay* OR peru OR peruvian* OR "peru's" OR philippine* OR philipine* OR phillipine* OR phillippine* OR filipino* OR filipina* OR poland* OR polish OR pole OR poles OR portugal* OR portuguese OR puerto-ric* OR romania* OR russia* OR ussr* OR soviet* OR rwanda* OR rwandese OR ruanda* OR ruandese OR samoa* OR navigator-island* OR pacific-island* OR polynesia* OR sao-tome* OR santomean* OR saudi-arabia* OR saudi OR saudis OR senegal* OR serbia* OR seychell* OR sierra-leone* OR slovak* OR sloven* OR melanesia* OR solomon-island* OR norfolk-island* OR somali* OR sri-lanka* OR ceylon* OR saint-kitts OR st-kitts OR kittian* OR nevisian* OR saint-lucia* OR st-lucia* OR saint-vincent* OR st-vincent* OR vincentian* OR grenadine* OR sudan* OR surinam* OR syria* OR tajik* OR tadjik* OR tadzhik* OR tanzania* OR tanganyika* OR thai* OR timor-leste* OR east-timor* OR timorese* OR togo OR togoles* OR "togo's" OR tonga* OR trinidad* OR tobago* OR tunisia* OR turkiy* OR turkey* OR turk OR turks OR turkish OR turkmen* OR uganda* OR ukrain* OR uruguay* OR uzbek* OR vanuatu* OR new-hebrides* OR venezuela* OR vietnam* OR viet-nam* OR yemen* OR yugoslav* OR zambia* OR zimbabwe* OR rhodesia* OR arab*-countr* OR middle-east* OR global-south OR sahara* OR subsahara* OR magreb* OR maghrib* OR west-indies* OR caribbean* OR central-america* OR latin-america* OR south-america* OR asia-central OR central-asia* OR asia-northern OR north-asia* OR northern-asia* OR asia-southeastern OR southeastern-asia* OR south-eastern-asia* OR southeast-asia* OR south-east-asia* OR asia-western OR west-asia* OR western-asia* OR europe-eastern OR east-europe* OR eastern-europe* OR developing-countr* OR developing-nation* OR developing-population* OR developing-world OR less-developed-countr* OR less-developed-nation* OR less-developed-world OR lesser-developed-countr* OR lesser-developed-nation* OR lesser-developed-world OR under-developed-countr* OR under-developed-nation* OR under-developed-world OR underdeveloped-countr* OR underdeveloped-nation* OR underdeveloped-world OR middle-income-countr* OR middle-income-nation* OR middle-income-population* OR low-income-countr* OR low-income-nation* OR low-income-population* OR lower-income-countr* OR lower-income-nation* OR lower-income-population* OR underserved-countr* OR underserved-nation* OR underserved-population* OR under-served-population* OR under-served-nation* OR under-served-population* OR deprived-countr* OR deprived-population* OR high-burden-countr* OR high-burden-nation* OR countdown-countr* OR countdown-nation* OR poor-countr* OR poor-nation* OR poor-population* OR poor-world OR poorer-countr* OR poorer-nation* OR poorer-population* OR poorer-world OR developing-econom* OR less-developed-econom* OR underdeveloped-econom* OR under-developed-econom* OR middle-income-econom* OR low-income-econom* OR lower-income-econom* OR low-gdp OR low-gnp OR low-gross-domestic OR low-gross-national OR lower-gdp OR lower-gnp OR lower-gross-domestic OR lower-gross-national OR lmic OR lmics OR third-world OR lami-countr* OR transitional-countr* OR emerging-econom* OR emerging-nation* |
| S53 | (MH "Developing Countries") OR (MH "Low and Middle Income Countries") |
| S52 | S45 AND S51 |
| S51 | S46 OR S47 OR S48 OR S49 OR S50 |
| S50 | (MH "Random Assignment") |
| S49 | (MH "Clinical Trials+") |
| S48 | TI ( randomis* or randomiz* or randomly) OR AB ( randomis* or randomiz* or randomly) |
| S47 | PT clinical trial |
| S46 | PT randomized controlled trial |
| S45 | S1 or (S23 AND S44) |
| S44 | S24 OR S25 OR S26 OR S27 OR S28 OR S29 OR S30 OR S31 OR S32 OR S33 OR S34 OR S35 OR S36 OR S37 OR S38 OR S39 OR S40 OR S41 OR S42 OR S43 |
| S43 | TI (midwife or midwive* or pharmacist* or pharmacy or pharmacies or practice-nurs* or district-nurs* or health-visitor*) OR AB (midwife or midwive* or pharmacist* or pharmacy or pharmacies or practice-nurs* or district-nurs* or health-visitor*) |
| S42 | TI (paraprofessional* or para-professional* or auxiliar* or paramedics or (allied-health* N0 (professional* or person* or staff or worker*) or non-physician* or non-clinician*) OR AB (paraprofessional* or para-professional* or auxiliar* or paramedics or (allied-health* N0 (professional* or person* or staff or worker*) or non-physician* or non-clinician*) |
| S41 | TI (lay N2 (heal* or person* or counsellor* or counselor* or worker* or therapist*)) OR AB (lay N2 (heal* or person* or counsellor* or counselor* or worker* or therapist*)) |
| S40 | TI (caregiver* or care-giver* or layperson*) OR AB (caregiver* or care-giver* or layperson*) |
| S39 | TI (psycho-social or psychosocial or psychological-intervention* OR task-shift* OR shifting-tasks OR task-sharing OR sharing-tasks OR delegating-tasks OR task-delegation) OR AB (psycho-social or psychosocial or psychological-intervention* OR task-shift* OR shifting-tasks OR task-sharing OR sharing-tasks OR delegating-tasks OR task-delegation) |
| S38 | TI (non-specialist* or nonspecialist* or social-worker* or trainer*) OR AB (non-specialist* or nonspecialist* or social-worker* or trainer*) |
| S37 | TI (school* or teacher* or rural* or community) OR AB (school* or teacher* or rural* or community) |
| S36 | TI (family-practi* or family-doctor* or family-physician* or gp* or general-practi*) OR AB (family-practi* or family-doctor* or family-physician* or gp* or general-practi*) |
| S35 | TI (primary N5 (care or health*)) OR AB (primary N5 (care or health*)) |
| S34 | (MH "Community Health Nursing+") OR (MH "Family Nursing") OR (MH "School Health Nursing") OR (MH "Rural Health Nursing") |
| S33 | (MH "Rural Population") |
| S32 | (MH "Rural Health") |
| S31 | (MH "School Health Services+") |
| S30 | (MH "Schools+") |
| S29 | (MH "Community Health Services+") |
| S28 | (MH "Community Health Workers") |
| S27 | (MH "Support, Psychosocial+") |
| S26 | (MH "Family Practice") |
| S25 | (MH "Physicians, Family") |
| S24 | (MH "Primary Health Care") |
| S23 | S2 OR S3 OR S4 OR S5 OR S6 OR S7 OR S8 OR S9 OR S10 OR S11 OR S12 OR S13 OR S14 OR S15 OR S16 OR S17 OR S18 OR S19 OR S20 OR S21 OR S22 |
| S22 | TI (mental*-develop*) OR AB (mental*-develop*) |
| S21 | TI (slow* N0 (thought* or think*)) OR AB (slow* N0 (thought* or think*)) |
| S20 | TI (anxiety-disorder* or agoraphobi* or general*-anxi* or separation-anxiety or neurocirculatory-asthenia or neurotic- disorder* or social-phobi* or self-harm* or self-injur* or suicid*) OR AB (anxiety-disorder* or agoraphobi* or general*-anxi* or separation-anxiety or neurocirculatory-asthenia or neurotic- disorder* or social-phobi* or self-harm* or self-injur* or suicid*) |
| S19 | TI (mental-relapse or fatigue or somatic-symptom* or worry or worries or panic or low-mood* or mood-problem*) OR AB (mental-relapse or fatigue or somatic-symptom* or worry or worries or panic or low-mood* or mood-problem*) |
| S18 | TI ((sub-syndrom* or sub-threshold or sub-clinical or subsyndrom* or subthreshold or subclinical or minor or brief) N0 (symptom* or disorder* or condition* or depress* or anxiety)) OR AB ((sub-syndrom* or sub-threshold or sub-clinical or subsyndrom* or subthreshold or subclinical or minor or brief) N0 (symptom* or disorder* or condition* or depress* or anxiety)) |
| S17 | TI ((mental or psychological or emotional or psycho-social or psychosocial) N0 (stress* or distress*)) OR AB ((mental or psychological or emotional or psycho-social or psychosocial) N0 (stress* or distress*)) |
| S16 | TI (affective* N0 (disorder* or disease* or illness* or symptom*)) OR AB (affective* N0 (disorder* or disease* or illness* or symptom*)) |
| S15 | TI (trichotillomani* or OCD or obsess*-compulsi* or GAD or stress-reaction* or acute-stress or neuros#s or neurotic) OR AB (trichotillomani* or OCD or obsess*-compulsi* or GAD or stress-reaction* or acute-stress or neuros#s or neurotic) |
| S14 | TI ((bipolar or behavio#ral or obsessive or panic or mood or delusional) N2 (disorder* or illness* or disease*)) OR AB ((bipolar or behavio#ral or obsessive or panic or mood or delusional) N2 (disorder* or illness* or disease*)) |
| S13 | TI ((dissociative N3 (disorder* or reaction*)) or dissociation) OR AB ((dissociative N3 (disorder* or reaction*)) or dissociation) |
| S12 | TI ((learning or mental* or intellectual) N0 (disabled or disabilit* or disorder* or difficult*)) OR AB ((learning or mental* or intellectual) N0 (disabled or disabilit* or disorder* or difficult*)) |
| S11 | TI (alcoholism or alcoholic* or drug-addict* or drug-abus* or drug-misuse or drug-user*) OR AB (alcoholism or alcoholic* or drug-addict* or drug-abus* or drug-misuse or drug-user*) |
| S10 | TI (psychological-trauma or psychotrauma*) OR AB (psychological-trauma or psychotrauma*) |
| S9 | TI ((post-trauma* or posttrauma*) N3 (stress* or disorder*)) OR AB ((post-trauma* or posttrauma*) N3 (stress* or disorder*)) |
| S8 | TI (depression or anxiety or alzheimer* or schizoaffective or mania or manic or borderline-personality or (stress N2 disorder*) or adjustment-disorder* or (psychological N1 trauma*) or schizophrenia or psychoses or psychosis or stress-syndrome* or distress-syndrome* or combat-disorder* or war-disorder* or ptsd or dementia) OR AB (depression or anxiety or alzheimer* or schizoaffective or mania or manic or borderline-personality or (stress N2 disorder*) or adjustment-disorder* or (psychological N1 trauma*) or schizophrenia or psychoses or psychosis or stress-syndrome* or distress-syndrome* or combat-disorder* or war-disorder* or ptsd or dementia) |
| S7 | TI ((depress* or distress*) N3 (postnatal* or post-natal* or maternal*)) OR AB ((depress* or distress*) N3 (postnatal* or post-natal* or maternal*)) |
| S6 | TI (depress* N3 (acute or clinical* or diagnos* or disorder* or major or unipolar or illness or scale* or score* or adult* or child* or adolesc* or teen* or youth* or elder* or late*-life* or patient* or participant* or people or inpatient* or in-patient* or outpatient* or out-patient*)) OR AB (depress* N3 (acute or clinical* or diagnos* or disorder* or major or unipolar or illness or scale* or score* or adult* or child* or adolesc* or teen* or youth* or elder* or late*-life* or patient* or participant* or people or inpatient* or in-patient* or outpatient* or out-patient*)) |
| S5 | TI (depressi* N2 (sign* or symptom* or disorder*)) OR AB (depressi* N2 (sign* or symptom* or disorder*)) |
| S4 | TI ((substance or alcohol or opioid or morphine or marijuana or heroin or cocaine) N2 (disorder* or illness* or dependence or abuse or misuse or use)) OR AB ((substance or alcohol or opioid or morphine or marijuana or heroin or cocaine) N2 (disorder* or illness* or dependence or abuse or misuse or "use")) |
| S3 | TI (mental-health* or mental*-ill* or mental*-disorder* or mental*-well*) OR AB (mental-health* or mental*-ill* or mental*-disorder* or mental*-well*) |
| S2 | (MH "Mental Disorders+") OR (MH "Mental Health") OR (MH "Persons with Intellectual Disabilities") OR (MH "Child Development") OR (MH "Injuries, Self-Inflicted") OR (MH "Self-Injurious Behavior") OR (MH "Suicide+") OR (MH "Mania") |
| S1 | (MH "Psychosocial Intervention") |

**Global Index Medicus (GIM), WHO**

| "substance related disorder" OR "substance related disorders" OR "substance abuse" OR "alcohol abuse" OR "alcohol dependence" OR "alcohol related" OR depressi* OR anxiety OR schizophrenia OR psychoses OR psychosis OR "stress syndrome" OR "distress syndrome" OR "combat disorder" OR "war disorder" OR "posttrauma stress" OR "post trauma stress" OR "posttraumatic stress" OR "post traumatic stress" OR ptsd OR dementia OR alcoholism OR alcoholic OR "drug addict" OR "drug abuse" OR "drug abuser" OR "drug misuse" OR "drug user" OR "drug users" OR "learning disabled" OR "learning disability" OR "learning disabilities" OR "learning disorder" OR "learning disorders" OR "learning difficulty" OR "learning difficulties" OR "mental disabled" OR "mental disability" OR "mental disabilities" OR "mental disorder" OR "mental disorders" OR "mental difficulty" OR "mental difficulties" OR "mentally disabled" OR "intellectual disability" OR "intellectual disabilities" OR "intellectual disorder" OR "intellectual disorders" OR "intellectual difficulty" OR "intellectual difficulties" OR "intellectually disabled" OR "mental health" |
| --- |
| AND |
| "primary health" OR "primary care" OR "primary healthcare" OR "community" OR school* OR teacher* OR rural OR "psycho social" OR psychosocial OR caregiver* OR paraprofessional* OR "lay counsellor" OR "lay counselor" OR "lay worker" OR "lay therapist" OR "lay counsellors" OR "lay counselors" OR "lay workers" OR "lay therapists" OR "general practice" OR "family practice" OR "midwife" OR "midwives" OR "health visitor" OR "social worker" OR "psychological intervention" OR "psychological interventions" OR "task shift" OR "task shifting" OR "shifting tasks" OR "task sharing" OR "sharing tasks" OR "delegating tasks" OR "task delegation" |
| AND: Filter: Type of study = controlled clinical trial |

**ClinicalTrials.gov**

| **Condition** | "mental health" OR "mental illness" OR "mental disorder" OR "mental disorders" |
| --- | --- |
| **AND** |  |
| **Intervention** | school OR psychosocial OR "psycho social" OR lay OR "non specialist" OR teacher OR paraprofessional OR "community based" OR "community mental health" OR "community worker" OR "primary care" OR "general practice" OR "family practice" |
| Limited to | ·         Age 18 or older |
|  | ·         Interventional Studies |

**ICTRP, WHO**

| **Condition** | mental health OR mental illness OR mental disorder OR mental disorders |
| --- | --- |
| **AND** |  |
| **Intervention** | non specialist OR non specialists OR community based OR community worker OR community workers OR primary care OR primary health care OR psychological intervention OR psychological interventions OR task shift OR task shifting OR shifting tasks OR task sharing OR sharing tasks OR delegating tasks OR task delegation |

**Appendix C – Explanation of prevention classification for included interventions***

| **Study ID** | **Classification** | **Explanation** |
| --- | --- | --- |
| Hirani 2010 | Universal prevention | All women living in the catchment area with no diagnosis of a mental health disorder were eligible |
| Comrie-Thomson 2022 | Universal prevention | All women who were pregnant or had a child aged up to two years and all men residing in intervention sites were invited to participate |
| Baker-Henningham 2019 | Universal prevention | All participants were eligible for inclusion (all grade 1 teachers in the school), and their baseline scores for CES-D were well below the cutoff for the measure |
| Bhatia 2023 | Selective prevention | Participants were included based upon the presence of a risk factor (addiction affected family members) |
|  |  |  |
| Ranasinghe 2022 | Selective prevention | Participants were included based upon the presence of a risk factor (women Diagnosed with Polycystic Ovary Syndrome) and presented with minimal levels of depressive symptoms as indicated by CES-D scores. Those with psychiatric illnesses were excluded |
| Miller 2023 | Selective prevention | Participants were included based upon the presence of a risk factor (syrian refugees’ caregiver) and presented with minimal levels of distress as indicated by K10 scores |
| Brathwaite 2023 | Selective prevention | Participants were included based upon the presence of a risk factor (children With Disruptive Behavior) with minimal levels of distress as indicated by BSI scores |
| Boobpamala 2024 | Selective prevention | Participants were included based upon the presence of a risk factor (pregnant adolescents) |
| Metzler 2023 | Selective prevention | Participants were included based upon the presence of a risk factor (South Sudanese refugee children in Uganda) |
| O’Callaghan 2014 | Selective prevention | Participants were included based upon the presence of a risk factor (living in war-affected area) and presented with some level of distress as indicated by the CRIES-8 scores that were below the cut-off for the measure |
| Dhital 2019 | Selective prevention | Adolescents from grades 6 to 8 from all the schools locat- ed in the districts that were affected by a natural disaster. Participants presented with some levels of distress which were well below the cut-off for the measure |
| Panter-Brick 2018 | Selective prevention | Participants were included based upon the presence of a risk factor. Participants presented with some level of distress as indicated by SDQ scores that were below the cut-off for the measure |
| Fabbri 2021 | Selective prevention | Participants presented with some level of distress as indicated by MFQ scores that were well below the cut-off for the measure. All students attending all schools in the refugee camp were eligible to be participants, 8.8% reported baseline levels of depression above the cut-off. |
| Langer 1996 | Selective prevention | Participants including all pregnant women with more than one risk factor |
| Byansi 2022 | Selective prevention | Adolescents were included based upon the presence of a risk factor (adolescent girls living in low-resource settings) |
| Jiang 2022 | Selective prevention | Participants were included based upon the presence of a risk factor (children affected by Parental HIV) |
| Greene 2023 | Selective prevention | Participants were eligible if they were migrant women reported no to moderate psychological distress (K6 < 13) |
| Sapkota 2022 | Indicated prevention | Participants presented with some level of depressive and anxiety symptoms as indicated by the HADS-A and HADS-D scores, which were around the cut-off |
| Yeomans 2010 | Indicated prevention | Participants presented with some level of distress as indicated by the HSCL-25 and HTQ scores |
| Lachman 2017 | Indicated prevention | Participants presented with some level of distress as indicated by the Beck Depression Inventory (BDI) scores, below the cut-off for clinically significant levels for the measure |
| Ward 2020 | Indicated prevention | Participants presented with some level of distress as indicated by the BDI scores |
| Dybdahl 2001 | Indicated prevention | Internally displaced refugees with experiences of traumatic events. They presented with some level of distress as indicated by the IES scores |
| Skar 2021 | Indicated prevention | 19.2% participants presented with some level of distress as indicated by SSQ scores |
| Lachman 2020 | Indicated prevention | Children presented with some level of distress as indicated by the CES-D scores |
| Sherman 2009 | Indicated prevention | Participants presented with some level of distress as indicated by the CES-D scores |
| Shinde 2018 | Indicated prevention | Participants presented with some level of distress as indicated by PHQ-9 scores |
| James 2019 | Indicated prevention | Participants, exposed to severe stressors. presented with some level of baseline anxiety as indicated by the BAI |
| Osborn 2020 | Indicated prevention | Participants presented with some level of distress as indicated by the PHQ-8 scores. |
| Donenberg 2021 | Indicated prevention | Participants presented with some level of distress as indicated by PHQ-9, GAD and PC-PTSD-5 scores |
| Friedberg 2023 | Indicated prevention | Adolescents were included based upon the presence of a risk factor (living in the informal settlements) and presented with some level of distress as indicated by the CPSS scores that were below the cut-off for the measure |
| Massarwi 2022 | Indicated prevention | Participants presented with some level of baseline distress as indicated by the PSS. They were included based upon the presence of a risk factor (parents living in areas suffer high rates of unemployment, poor infrastructure and high HIV/AIDS prevalence) |

***References**

Purgato M, Prina E, Ceccarelli C, et al. Primary-level and community worker interventions for the prevention of mental disorders and the promotion of well-being in low- and middle-income countries. Cochrane Database Syst Rev. 2023;10(10):CD014722. Published 2023 Oct 24. doi:10.1002/14651858.CD014722.pub2

Ceccarelli, C., Prina, E., Muneghina, O., Jordans, M., Barker, E., Miller, K., Singh, R., Acarturk, C., Sorsdhal, K., Cuijpers, P., Lund, C., Barbui, C., & Purgato, M. (2022). Adverse childhood experiences and global mental health: avenues to reduce the burden of child and adolescent mental disorders. Epidemiology and psychiatric sciences, 31, e75. <https://doi.org/10.1017/S2045796022000580>

Institute of Medicine (US) Committee on Prevention of Mental Disorders. (1994). Reducing Risks for Mental Disorders: Frontiers for Preventive Intervention Research (P. J. Mrazek & R. J. Haggerty, Eds.). National Academies Press (US). <http://www.ncbi.nlm.nih.gov/books/NBK236319/>

Purgato, M., Uphoff, E., Singh, R., Thapa Pachya, A., Abdulmalik, J., & van Ginneken, N. (2020). Promotion, prevention and treatment interventions for mental health in low- and middle-income countries through a task-shifting approach. Epidemiology and psychiatric sciences, 29, e150. https://doi.org/10.1017/S204579602000061X

Tol, W. A., Purgato, M., Bass, J. K., Galappatti, A., & Eaton, W. (2015). Mental health and psychosocial support in humanitarian settings: a public mental health perspective. Epidemiology and psychiatric sciences, 24(6), 484–494. <https://doi.org/10.1017/S2045796015000827>

William W. Eaton (2019), Public Mental Health (2nd edition), Oxford University Press.

**Appendix D - Trials included in the systematic review**

1. Baker-Henningham H, Scott Y, Bowers M, Francis T. Evaluation of a Violence-Prevention Programme with Jamaican Primary School Teachers: A Cluster Randomised Trial. IJERPH [Internet]. 2019 Aug 6 [cited 2024 Mar 1];16(15):2797. Available from: <https://www.mdpi.com/1660-4601/16/15/2797>
2. Bhatia K, Rath S, Pradhan H, Samal S, Copas A, Gagrai S, et al. Effects of community youth teams facilitating participatory adolescent groups, youth leadership activities and livelihood promotion to improve school attendance, dietary diversity and mental health among adolescent girls in rural eastern India (JIAH trial): A cluster-randomised controlled trial. SSM - Population Health [Internet]. 2023 Mar [cited 2024 Mar 1];21:101330. Available from: <https://linkinghub.elsevier.com/retrieve/pii/S2352827322003093>
3. Boobpamala S, Kongvattananon P, Griffin MTQ. Effectiveness of an Early Depression Prevention Program on Coping Skills and Depression among Pregnant Adolescents: A Randomized Controlled Trial. Pacific Rim International Journal of Nursing Research. 2022;26(2).
4. Brathwaite R, Sensoy Bahar O, Mutumba M, Byansi W, Namatovu P, Namuwonge F, et al. Short-Term Impact of “Amaka Amasanyufu” Multiple Family Group Intervention on Mental Health Functioning of Children With Disruptive Behavior Disorders in Uganda. Journal of the American Academy of Child & Adolescent Psychiatry [Internet]. 2023 Jul [cited 2024 Mar 1];62(7):777–90. Available from: <https://linkinghub.elsevier.com/retrieve/pii/S0890856723001181>
5. Byansi W, Ssewamala FM, Neilands TB, Sensoy Bahar O, Nabunya P, Namuwonge F, et al. The Short-Term Impact of a Combination Intervention on Depressive Symptoms Among School-Going Adolescent Girls in Southwestern Uganda: The Suubi4Her Cluster Randomized Trial. Journal of Adolescent Health [Internet]. 2022 Sep [cited 2024 Mar 1];71(3):301–7. Available from: <https://linkinghub.elsevier.com/retrieve/pii/S1054139X22004165>
6. Comrie-Thomson L, Webb K, Patel D, Wata P, Kapamurandu Z, Mushavi A, et al. Engaging women and men in the gender-synchronised, community-based Mbereko+Men intervention to improve maternal mental health and perinatal care-seeking in Manicaland, Zimbabwe: A cluster-randomised controlled pragmatic trial. J Glob Health [Internet]. 2022 May 21 [cited 2024 Mar 1];12:04042. Available from: <https://jogh.org/2022/jogh-12-04042>
7. Dhital R, Shibanuma A, Miyaguchi M, Kiriya J, Jimba M. Effect of psycho-social support by teachers on improving mental health and hope of adolescents in an earthquake-affected district in Nepal: A cluster randomized controlled trial. Francis JM, editor. PLoS ONE [Internet]. 2019 Oct 1 [cited 2024 Mar 1];14(10):e0223046. Available from: <https://dx.plos.org/10.1371/journal.pone.0223046>
8. Donenberg G, Merrill KG, Atujuna M, Emerson E, Bray B, Bekker LG. Mental health outcomes of a pilot 2-arm randomized controlled trial of a HIV-prevention program for South African adolescent girls and young women and their female caregivers. BMC Public Health [Internet]. 2021 Dec [cited 2024 Mar 1];21(1):2189. Available from: <https://bmcpublichealth.biomedcentral.com/articles/10.1186/s12889-021-12010-1>
9. Dybdahl R. Children and Mothers in War: An Outcome Study of a Psychosocial Intervention Program. Child Development [Internet]. 2001 Aug [cited 2024 Mar 1];72(4):1214–30. Available from: <https://srcd.onlinelibrary.wiley.com/doi/10.1111/1467-8624.00343>
10. Fabbri C, Rodrigues K, Leurent B, Allen E, Qiu M, Zuakulu M, et al. The EmpaTeach intervention for reducing physical violence from teachers to students in Nyarugusu Refugee Camp: A cluster-randomised controlled trial. Yount KM, editor. PLoS Med [Internet]. 2021 Oct 4 [cited 2024 Mar 1];18(10):e1003808. Available from: <https://dx.plos.org/10.1371/journal.pmed.1003808>
11. Friedberg R, Baiocchi M, Rosenman E, Amuyunzu-Nyamongo M, Nyairo G, Sarnquist C. Mental health and gender-based violence: An exploration of depression, PTSD, and anxiety among adolescents in Kenyan informal settlements participating in an empowerment intervention. Benetreau Y, editor. PLoS ONE [Internet]. 2023 Mar 29 [cited 2024 Mar 1];18(3):e0281800. Available from: <https://dx.plos.org/10.1371/journal.pone.0281800>
12. Greene, M. C., Bonz, A. G., Cristobal, M., Angulo, A., Armijos, A., Guevara, M. E., Vega, C., Benavides, L., Corrales, C., de la Cruz, A., Lopez, M. J., Moyano, A., Murcia, A., Noboa, M. J., Rodriguez, A., Solis, J., Vergara, D., Bollman, E. B., Andersen, L. S., Wainberg, M., & Tol, W. A. (2023). Mixed-methods evaluation of a group psychosocial intervention for refugee, migrant and host community women in Ecuador and Panamá: Results from the Entre Nosotras cluster randomized feasibility trial. *Cambridge Prisms: Global Mental Health, 10*, e42, 1–12. <https://doi.org/10.1017/gmh.2023.37>
13. Hirani SS, Karmaliani R, McFarlane J, Asad N, Madhani F, Shehzad S. TESTING A COMMUNITY DERIVED INTERVENTION TO PROMOTE WOMEN’S HEALTH: PRELIMINARY RESULTS OF A 3-ARM RANDOMIZED CONTROLLED TRIAL IN KARACHI, PAKISTAN. Southern Online Journal of Nursing Research [Internet]. 2010;10(3). Available from: <https://snrs.org/wp-content/uploads/2022/02/Vol10Num03Art06.pdf>
14. James LE, Welton-Mitchell C, Noel JR, James AS. Integrating mental health and disaster preparedness in intervention: a randomized controlled trial with earthquake and flood-affected communities in Haiti. Psychol Med [Internet]. 2020 Jan [cited 2024 Mar 1];50(2):342–52. Available from: <https://www.cambridge.org/core/product/identifier/S0033291719000163/type/journal_article>
15. Jiang Y, Li X, Harrison SE, Zhang J, Qiao S, Zhao J, et al. Effects of a Multilevel Resilience-Based Intervention on Mental Health for Children Affected by Parental HIV: A Cluster Randomized Controlled Trial. J Child Fam Stud [Internet]. 2022 Apr [cited 2024 Mar 1];31(4):1094–105. Available from: <https://link.springer.com/10.1007/s10826-022-02236-x>
16. Lachman J, Wamoyi J, Spreckelsen T, Wight D, Maganga J, Gardner F. Combining parenting and economic strengthening programmes to reduce violence against children: a cluster randomised controlled trial with predominantly male caregivers in rural Tanzania. BMJ Glob Health [Internet]. 2020 Jul [cited 2024 Mar 1];5(7):e002349. Available from: <https://gh.bmj.com/lookup/doi/10.1136/bmjgh-2020-002349>
17. Lachman JM, Cluver L, Ward CL, Hutchings J, Mlotshwa S, Wessels I, et al. Randomized controlled trial of a parenting program to reduce the risk of child maltreatment in South Africa. Child Abuse & Neglect [Internet]. 2017 Oct [cited 2024 Mar 1];72:338–51. Available from: <https://linkinghub.elsevier.com/retrieve/pii/S0145213417302922>
18. Langer A, Farnot U, Garcia C, Barros F, Victora C, Belizan JM, et al. The Latin American trial of psychosocial support during pregnancy: Effects on mother’s wellbeing and satisfaction. Social Science & Medicine [Internet]. 1996 Jun [cited 2024 Mar 1];42(11):1589–97. Available from: <https://linkinghub.elsevier.com/retrieve/pii/0277953695002626>
19. Massarwi, A. A., Cluver, L., Meinck, F., Doubt, J., & Green, O. (2022). Pathways to parenting stress reduction among parents in South Africa. *Child & Family Social Work*, *28*(1), 207–216. <https://doi.org/10.1111/cfs.12952>
20. Metzler J, Saw T, Nono D, Kadondi A, Zhang Y, Leu C, et al. Improving adolescent mental health and protection in humanitarian settings: longitudinal findings from a multi‐arm randomized controlled trial of child‐friendly spaces among South Sudanese refugees in Uganda. Child Psychology Psychiatry [Internet]. 2023 Jun [cited 2024 Mar 1];64(6):907–17. Available from: <https://acamh.onlinelibrary.wiley.com/doi/10.1111/jcpp.13746>
21. Miller KE, Chen A, Koppenol‐Gonzalez GV, Bakolis I, Arnous M, Tossyeh F, et al. Supporting parenting among Syrian refugees in Lebanon: a randomized controlled trial of the caregiver support intervention. Child Psychology Psychiatry [Internet]. 2023 Jan [cited 2024 Mar 1];64(1):71–82. Available from: <https://acamh.onlinelibrary.wiley.com/doi/10.1111/jcpp.13668>
22. O’Callaghan P, Branham L, Shannon C, Betancourt TS, Dempster M, McMullen J. A pilot study of a family focused, psychosocial intervention with war-exposed youth at risk of attack and abduction in north-eastern Democratic Republic of Congo. Child Abuse & Neglect [Internet]. 2014 Jul [cited 2024 Mar 1];38(7):1197–207. Available from: <https://linkinghub.elsevier.com/retrieve/pii/S014521341400043X>
23. Osborn TL, Rodriguez M, Wasil AR, Venturo-Conerly KE, Gan J, Alemu RG, et al. Single-session digital intervention for adolescent depression, anxiety, and well-being: Outcomes of a randomized controlled trial with Kenyan adolescents. Journal of Consulting and Clinical Psychology [Internet]. 2020 Jul [cited 2024 Mar 1];88(7):657–68. Available from: <https://doi.apa.org/doi/10.1037/ccp0000505>
24. Panter‐Brick C, Dajani R, Eggerman M, Hermosilla S, Sancilio A, Ager A. Insecurity, distress and mental health: experimental and randomized controlled trials of a psychosocial intervention for youth affected by the Syrian crisis. Child Psychology Psychiatry [Internet]. 2018 May [cited 2024 Mar 1];59(5):523–41. Available from: <https://acamh.onlinelibrary.wiley.com/doi/10.1111/jcpp.12832>
25. Ranasinghe B, Balasuriya A, Wijeyaratne C, Fernando N. The impact of peer-led support groups on health-related quality of life, coping skills and depressive symptomatology for women with PCOS. Psychology, Health & Medicine [Internet]. 2023 Mar 16 [cited 2024 Mar 1];28(3):564–73. Available from: <https://www.tandfonline.com/doi/full/10.1080/13548506.2021.2019805>
26. Sapkota D, Baird K, Saito A, Rijal P, Anderson D. Antenatal-Based Pilot Psychosocial Intervention to Enhance Mental Health of Pregnant Women Experiencing Domestic and Family Violence in Nepal. J Interpers Violence [Internet]. 2022 Mar [cited 2024 Mar 1];37(5–6):NP3605–27. Available from: <http://journals.sagepub.com/doi/10.1177/0886260520948151>
27. Sherman SG, Sutcliffe C, Srirojn B, Latkin CA, Aramratanna A, Celentano DD. Evaluation of a peer network intervention trial among young methamphetamine users in Chiang Mai, Thailand. Social Science & Medicine [Internet]. 2009 Jan [cited 2024 Mar 1];68(1):69–79. Available from: <https://linkinghub.elsevier.com/retrieve/pii/S0277953608005157>
28. Shinde S, Weiss HA, Varghese B, Khandeparkar P, Pereira B, Sharma A, et al. Promoting school climate and health outcomes with the SEHER multi-component secondary school intervention in Bihar, India: a cluster-randomised controlled trial. The Lancet [Internet]. 2018 Dec [cited 2024 Mar 1];392(10163):2465–77. Available from: <https://linkinghub.elsevier.com/retrieve/pii/S0140673618316155>
29. Skar AMS, Sherr L, Macedo A, Tetzchner SV, Fostervold KI. Evaluation of Parenting Interventions to Prevent Violence Against Children in Colombia: A Randomized Controlled Trial. J Interpers Violence [Internet]. 2021 Jan [cited 2024 Mar 1];36(1–2):NP1098–126. Available from: <http://journals.sagepub.com/doi/10.1177/0886260517736881>
30. Ward CL, Wessels IM, Lachman JM, Hutchings J, Cluver LD, Kassanjee R, et al. Parenting for Lifelong Health for Young Children: a randomized controlled trial of a parenting program in South Africa to prevent harsh parenting and child conduct problems. Child Psychology Psychiatry [Internet]. 2020 Apr [cited 2024 Mar 1];61(4):503–12. Available from: <https://acamh.onlinelibrary.wiley.com/doi/10.1111/jcpp.13129>
31. Yeomans PD, Forman EM, Herbert JD, Yuen E. A randomized trial of a reconciliation workshop with and without PTSD psychoeducation in Burundian sample. Journal of Traumatic Stress [Internet]. 2010 Jun [cited 2024 Mar 1];23(3):305–12. Available from: <https://onlinelibrary.wiley.com/doi/10.1002/jts.20531>

**Appendix E** **– Trials excluded from the systematic review, with reasons**

| **Full reference** | **Reason for exclusion** |
| --- | --- |
| Abdelrasheed N, Khalaf M. The Effectiveness of Motivational counseling in improving Psychological vitality of Teachers in Dhofar Region Schools - Sultanate of Oman. Eur Psychiatr [Internet]. 2022 Jun [cited 2024 Apr 17];65(S1):S696–S696. Available from: <https://www.cambridge.org/core/product/identifier/S0924933822017928/type/journal_article> | Wrong intervention |
| Abraham AM, Sudhir PM, Philip M, Bantwal G. Efficacy of a Brief Self-management Intervention in Type 2 Diabetes Mellitus: A Randomized Controlled Trial from India. Indian Journal of Psychological Medicine [Internet]. 2020 Nov [cited 2024 Apr 17];42(6):540–8. Available from: <http://journals.sagepub.com/doi/10.1177/0253717620932250> | Wrong intervention |
| Ahmadi SJ, Musavi Z, Samim N, Sadeqi M, Jobson L. Investigating the Feasibility, Acceptability and Efficacy of Using Modified-Written Exposure Therapy in the Aftermath of a Terrorist Attack on Symptoms of Posttraumatic Stress Disorder Among Afghan Adolescent Girls. Front Psychiatry [Internet]. 2022 Apr 8 [cited 2024 Apr 17];13:826633. Available from: <https://www.frontiersin.org/articles/10.3389/fpsyt.2022.826633/full> | Wrong outcome |
| Akhtar A, Malik A, Ghatasheh M, Aqel IS, Habashneh R, Dawson KS, et al. Feasibility trial of a brief scalable psychological intervention for Syrian refugee adolescents in Jordan. European Journal of Psychotraumatology [Internet]. 2021 Jan [cited 2024 Apr 4];12(1):1901408. Available from: <https://www.tandfonline.com/doi/full/10.1080/20008198.2021.1901408> | Wrong intervention |
| Alberty R, Čillík I. Effect of after‐school physical activity on body composition in primary school children: The Slovak “ PAD ” project. Physiological Reports [Internet]. 2023 Jan [cited 2024 Apr 4];11(1):e15540. Available from: <https://physoc.onlinelibrary.wiley.com/doi/10.14814/phy2.15540> | Wrong setting |
| Aliabadi S, Shayan A, Refaei M, Tapak L, Moradveisi L. The effect of individual counseling based on the GATHER principles on perceived stress and empowerment of the mothers with high-risk pregnancies: an experimental study. BMC Psychiatry [Internet]. 2022 Dec [cited 2024 Apr 17];22(1):396. Available from: <https://bmcpsychiatry.biomedcentral.com/articles/10.1186/s12888-022-04047-2> | Wrong intervention |
| Amato TDC, Opaleye ES, McBride N, Noto AR. Reducing alcohol-related risks among adolescents: a feasibility study of the SHAHRP program in Brazilian schools. Ciênc saúde coletiva [Internet]. 2021 Aug [cited 2024 Apr 17];26(8):3005–18. Available from: <http://www.scielo.br/scielo.php?script=sci_arttext&pid=S1413-81232021000803005&tlng=en> | Wrong outcome |
| Amoah J, Said S, Rampal L, Manaf R, Ibrahim N, Owusu-Agyei S, et al. Effects of a school-based intervention to reduce cardiovascular disease risk factors among secondary school students: A cluster-randomized, controlled trial. Abdelbasset WK, editor. PLoS ONE [Internet]. 2021 Nov 11 [cited 2024 Apr 17];16(11):e0259581. Available from: <https://dx.plos.org/10.1371/journal.pone.0259581> | Wrong outcome |
| Andreu CI, García‐Rubio C, Melcón M, Schonert‐Reichl KA, Albert J. The effectiveness of a school mindfulness‐based intervention on the neural correlates of inhibitory control in children at risk: A randomized control trial. Developmental Science [Internet]. 2023 Nov [cited 2024 Apr 17];26(6):e13403. Available from: <https://onlinelibrary.wiley.com/doi/10.1111/desc.13403> | Wrong outcome |
| Aneke AO, Ede MO, Agbigwe IB, Obumse NA, Nnamani O, Ngwoke AN, et al. Examining the impact of randomized control intervention on depressive symptoms in schoolchildren with atypical behaviors. Medicine [Internet]. 2023 Feb 17 [cited 2024 Apr 4];102(7):e32964. Available from: <https://journals.lww.com/10.1097/MD.0000000000032964> | Wrong intervention |
| Assonov D. Two-step resilience-oriented intervention for veterans with traumatic brain injury: a pilot randomized controlled trial. Clinical Neuropsychiatry [Internet]. 2021 Nov [cited 2024 Apr 17];18(5):247–59. Available from: <https://doi.org/10.36131/cnfioritieditore20210503> | Wrong intervention |
| Augsburg B, Attanasio OP, Dreibelbis R, Nketiah-Amponsah E, Phimister A, Wolf S, et al. Lively Minds: improving health and development through play–a randomised controlled trial evaluation of a comprehensive ECCE programme at scale in Ghana. BMJ Open [Internet]. 2022 Oct [cited 2024 Apr 17];12(10):e061571. Available from: <https://bmjopen.bmj.com/lookup/doi/10.1136/bmjopen-2022-061571> | Wrong intervention |
| Bayhan BB, Tarquinio C, Rydberg J, Korkmazlar Ü. The study of the group intervention containing EMDR therapy for children and mothers in the field of trauma after a mine explosion in Turkey. European Journal of Trauma & Dissociation [Internet]. 2022 Feb [cited 2024 Apr 4];6(1):100248. Available from: <https://linkinghub.elsevier.com/retrieve/pii/S246874992100048X> | Wrong intervention |
| Boele FW, Rooney AG, Bulbeck H, Sherwood P. Interventions to help support caregivers of people with a brain or spinal cord tumour. Cochrane Gynaecological, Neuro-oncology and Orphan Cancer Group, editor. Cochrane Database of Systematic Reviews [Internet]. 2019 Jul 2 [cited 2024 Apr 4]; Available from: <https://doi.wiley.com/10.1002/14651858.CD012582.pub2> | Wrong intervention |
| Booth-LaForce C, Oxford ML, O’Leary R, Buchwald DS. Promoting First Relationships® for Primary Caregivers and Toddlers in a Native Community: a Randomized Controlled Trial. Prev Sci [Internet]. 2023 Jan [cited 2024 Apr 17];24(1):39–49. Available from: <https://link.springer.com/10.1007/s11121-022-01415-y> | Wrong intervention |
| Brown FL, Taha K, Steen F, Kane J, Gillman A, Aoun M, et al. Feasibility randomised controlled trial of the Early Adolescent Skills for Emotions psychological intervention with young adolescents in Lebanon. BMC Psychiatry [Internet]. 2023 Mar 1 [cited 2024 Apr 17];23(1):131. Available from: <https://bmcpsychiatry.biomedcentral.com/articles/10.1186/s12888-023-04571-9> | Wrong intervention |
| Bryant RA, Malik A, Aqel IS, Ghatasheh M, Habashneh R, Dawson KS, et al. Effectiveness of a brief group behavioural intervention on psychological distress in young adolescent Syrian refugees: A randomised controlled trial. Grais RF, editor. PLoS Med [Internet]. 2022 Aug 12 [cited 2024 Apr 4];19(8):e1004046. Available from: <https://dx.plos.org/10.1371/journal.pmed.1004046> | Wrong population |
| Caiado B, Góis A, Pereira B, Canavarro MC, Moreira H. The Unified Protocol for Transdiagnostic Treatment of Emotional Disorders in Children (UP-C) in Portugal: Feasibility Study Results. IJERPH [Internet]. 2022 Feb 4 [cited 2024 Apr 4];19(3):1782. Available from: <https://www.mdpi.com/1660-4601/19/3/1782> | Wrong setting |
| Cetin SY, Comak E, Akman S. Proceedings of the 28th European Paediatric Rheumatology Congress (PReS 2022): Prague, Czech Republic. 20-23 September 2022. Pediatr Rheumatol [Internet]. 2022 Sep 7 [cited 2024 Apr 17];20(S2):75, s12969-022-00729-z. Available from: <https://ped-rheum.biomedcentral.com/articles/10.1186/s12969-022-00729-z> | Wrong outcome |
| Chen J, Sang G, Zhang Y, Jiang A. INTERVENTION EFFECT OF THE INTEGRATION MODEL ON NEGATIVE EMOTIONS OF ADOLESCENTS DURING THE OUTBREAK OF CORONA VIRUS DISEASE 2019. Psychiat Danub [Internet]. 2021 Apr 14 [cited 2024 Apr 4];33(1):86–94. Available from: <http://www.psychiatria-danubina.com/UserDocsImages/pdf/dnb_vol33_no1/dnb_vol33_no1_86.pdf> | Wrong intervention |
| Chu L, Zhu P, Ma C, Pan L, Shen L, Wu D, et al. Effects of Combing Group Executive Functioning and Online Parent Training on School-Aged Children With ADHD: A Randomized Controlled Trial. Front Pediatr [Internet]. 2022 Feb 11 [cited 2024 Apr 4];9:813305. Available from: <https://www.frontiersin.org/articles/10.3389/fped.2021.813305/full> | Wrong intervention |
| Coetzee BJ, Loades ME, Human S, Gericke H, Loxton H, Laning G, et al. 4 Steps To My Future (4STMF): protocol for a universal school-based pilot and feasibility study of a CBT-based psychoeducational intervention to support psychological well-being amongst young adolescents in the Western Cape, South Africa. Pilot Feasibility Stud [Internet]. 2022 Dec [cited 2024 Apr 17];8(1):99. Available from: <https://pilotfeasibilitystudies.biomedcentral.com/articles/10.1186/s40814-022-01035-x> | Wrong intervention |
| Dapari R, Bashaabidin MSM, Hassan MR, Dom NC, Rahim SSSA, Wan Mahiyuddin WR. Health Education Module Based on Information–Motivation–Behavioural Skills (IMB) for Reducing Depression, Anxiety, and Stress among Adolescents in Boarding Schools: A Clustered Randomised Controlled Trial. IJERPH [Internet]. 2022 Nov 21 [cited 2024 Apr 17];19(22):15362. Available from: <https://www.mdpi.com/1660-4601/19/22/15362> | Wrong intervention |
| Del Río NG, González-González CS, Martín-González R, Navarro-Adelantado V, Toledo-Delgado P, García-Peñalvo F. Effects of a Gamified Educational Program in the Nutrition of Children with Obesity. J Med Syst [Internet]. 2019 Jul [cited 2024 Apr 4];43(7):198. Available from: <http://link.springer.com/10.1007/s10916-019-1293-6> | Wrong study design |
| Egbegi DR, Bella-Awusah T, Omigbodun O, Ani C. A controlled trial of Cognitive Behavioural Therapy-based strategies for insomnia among in-school adolescents in southern Nigeria. Child Adolesc Psychiatry Ment Health [Internet]. 2021 Dec [cited 2024 Apr 4];15(1):52. Available from: <https://capmh.biomedcentral.com/articles/10.1186/s13034-021-00406-1> | Wrong intervention |
| Fladeboe KM, Scott S, Comiskey L, Zhou C, Yi-Frazier JP, Rosenberg AR. The Promoting Resilience in Stress Management (PRISM) intervention for adolescents and young adults receiving hematopoietic cell transplantation: a randomized controlled trial protocol. BMC Palliat Care [Internet]. 2022 Dec [cited 2024 Apr 17];21(1):82. Available from: <https://bmcpalliatcare.biomedcentral.com/articles/10.1186/s12904-022-00966-9> | Wrong setting |
| Flanagan S, Gorstein A, Nicholson M, Bradish S, Amanyire D, Gidudu A, et al. Behavioural intervention for adolescent uptake of family planning: a randomized controlled trial, Uganda. Bull World Health Org [Internet]. 2021 Nov 1 [cited 2024 Apr 4];99(11):795–804. Available from: <https://www.ncbi.nlm.nih.gov/pmc/articles/PMC8542266/pdf/BLT.20.285339.pdf> | Wrong outcome |
| Gaete J, Inzunza C, Ramírez S, Valenzuela D, Rojas C, Araya R. The Social Competence Promotion Program among Young Adolescents (SCPP-YA) in Chile (“Mi Mejor Plan”) for substance use prevention among early adolescents: study protocol for a randomized controlled trial. Trials [Internet]. 2022 Dec [cited 2024 Apr 17];23(1):542. Available from: <https://trialsjournal.biomedcentral.com/articles/10.1186/s13063-022-06472-w> | Wrong setting |
| Galasso E, Ratsifandrihamanana L, Weber AM, Hemlock C, Col M, Dieci M, et al. Integrating early stimulation and play at scale: study protocol for “MAHAY Mikolo”, a multi-arm cluster-randomized controlled trial. BMC Public Health [Internet]. 2022 Dec [cited 2024 Apr 17];22(1):265. Available from: <https://bmcpublichealth.biomedcentral.com/articles/10.1186/s12889-022-12640-z> | Wrong intervention |
| Golshiri P, Mostofi A, Rouzbahani S. The effect of problem-solving and assertiveness training on self-esteem and mental health of female adolescents: a randomized clinical trial. BMC Psychol [Internet]. 2023 Apr 9 [cited 2024 Apr 17];11(1):106. Available from: <https://bmcpsychology.biomedcentral.com/articles/10.1186/s40359-023-01154-x> | Wrong intervention |
| Gonsalves PP, Sharma R, Hodgson E, Bhat B, Jambhale A, Weiss HA, et al. A Guided Internet-Based Problem-Solving Intervention Delivered Through Smartphones for Secondary School Pupils During the COVID-19 Pandemic in India: Protocol for a Pilot Randomized Controlled Trial. JMIR Res Protoc [Internet]. 2021 Oct 6 [cited 2024 Apr 4];10(10):e30339. Available from: <https://www.researchprotocols.org/2021/10/e30339> | Wrong intervention |
| Guo J, Liu X, Huang N, Yang F, Bai Y, Zhang B, et al. School-based Psychosocial interventions on mental health among Chinese rural children with traumatic experiences: a protocol using cluster randomized controlled trial. BMC Psychol [Internet]. 2023 May 4 [cited 2024 Apr 4];11(1):148. Available from: <https://bmcpsychology.biomedcentral.com/articles/10.1186/s40359-023-01182-7> | Wrong intervention |
| Gusmoes JD, Garcia-Cerde R, Valente JY, Pinsky I, Sanchez ZM. Implementation fidelity of a Brazilian drug use prevention program and its effect among adolescents: a mixed-methods study. Subst Abuse Treat Prev Policy [Internet]. 2022 Nov 1 [cited 2024 Apr 4];17(1):71. Available from: <https://substanceabusepolicy.biomedcentral.com/articles/10.1186/s13011-022-00496-w> | Wrong outcome |
| Hasselle AJ, Howell KH, Gilliam HC. Self-Perception Among Children Exposed to Family Violence: A Pilot Randomized Controlled Trial Investigating the Effectiveness of a Strengths-Based Camp Intervention. Child Youth Care Forum [Internet]. 2024 Feb [cited 2024 Apr 4];53(1):73–94. Available from: <https://link.springer.com/10.1007/s10566-023-09744-x> | Wrong setting |
| Heizomi H, Allahverdipour H, Jafarabadi MA, Bhalla D, Nadrian H. Effects of a mental health promotion intervention on mental health of Iranian female adolescents: a school-based study. Child Adolesc Psychiatry Ment Health [Internet]. 2020 Dec [cited 2024 Apr 4];14(1):36. Available from: <https://capmh.biomedcentral.com/articles/10.1186/s13034-020-00342-6> | Wrong intervention |
| Ho KY, Lam KKW, Bressington DT, Lin J, Mak YW, Wu C, et al. Use of a positive psychology intervention (PPI) to promote the psychological well-being of children living in poverty: study protocol for a feasibility randomised controlled trial. BMJ Open [Internet]. 2022 Aug [cited 2024 Apr 17];12(8):e055506. Available from: <https://bmjopen.bmj.com/lookup/doi/10.1136/bmjopen-2021-055506> | Wrong setting |
| Hojati Abed E, Shafaroodi N, Zareiyan A, Akbarfahimi M, Parand A. The Effect of Self-Determination Activities on Communication and Interaction Skills and Academic Success (Grade Point Average) of Students at Risk of Emotional-Behavioral Disorders: A Randomized Controlled Trial. Med J Islam Republ Iran [Internet]. 2022 | Wrong outcome |
| Hosaka KRJ, Mmbaga BT, Shayo AM, Gallis JA, Turner EL, O’Donnell KE, et al. A group-based mental health intervention for Tanzanian youth living with HIV: Secondary analysis of a pilot trial. Medicine [Internet]. 2022 Feb 18 [cited 2024 Apr 17];101(7):e28693. Available from: <https://journals.lww.com/10.1097/MD.0000000000028693> | Wrong intervention |
| Husain N, Tofique S, Chaudhry IB, Kiran T, Taylor P, Williams C, et al. Youth Culturally adapted Manual Assisted Problem Solving Training (YCMAP) in Pakistani adolescent with a history of self-harm: protocol for multicentre clinical and cost-effectiveness randomised controlled trial. BMJ Open [Internet]. 2022 May [cited 2024 Apr 4];12(5):e056301. Available from: <https://bmjopen.bmj.com/lookup/doi/10.1136/bmjopen-2021-056301> | Wrong intervention |
| Janowski R, Green O, Shenderovich Y, Stern D, Clements L, Wamoyi J, et al. Optimising engagement in a digital parenting intervention to prevent violence against adolescents in Tanzania: protocol for a cluster randomised factorial trial. BMC Public Health [Internet]. 2023 Jun 23 [cited 2024 Apr 17];23(1):1224. Available from: <https://bmcpublichealth.biomedcentral.com/articles/10.1186/s12889-023-15989-x> | Wrong intervention |
| Javid N, Ahmadi A, Mirzaei M, Atghaei M. Effectiveness of Solution-Focused Group Counseling on the Mental Health of Midwifery Students. Rev Bras Ginecol Obstet [Internet]. 2019 Aug [cited 2024 Apr 4];41(08):500–7. Available from: <http://www.thieme-connect.de/DOI/DOI?10.1055/s-0039-1693741> | Wrong intervention |
| Jibunoh O, Ani C. A controlled clinical trial of a brief psycho-educational intervention for anxiety among in-school adolescents in Nigeria. International Journal of Mental Health [Internet]. 2022 Jan 2 [cited 2024 Apr 4];51(1):24–31. Available from: <https://www.tandfonline.com/doi/full/10.1080/00207411.2021.1891362> | Wrong intervention |
| Jordans MJD, Steen F, Koppenol-Gonzalez GV, El Masri R, Coetzee AR, Chamate S, et al. Evaluation of competency-driven training for facilitators delivering a psychological intervention for children in Lebanon: a proof-of-concept study. Epidemiol Psychiatr Sci [Internet]. 2022 [cited 2024 Apr 17];31:e48. Available from: <https://www.cambridge.org/core/product/identifier/S2045796022000348/type/journal_article> | Wrong outcome |
| Kallianta MDK, Katsira XE, Tsitsika AK, Vlachakis D, Chrousos G, Darviri C, et al. Stress management intervention to enhance adolescent resilience: a randomized controlled trial. EMBnet j [Internet]. 2021 Aug 23 [cited 2024 Apr 4];26(1):e967. Available from: <http://journal.embnet.org/index.php/embnetjournal/article/view/967> | Wrong intervention |
| Kansiime C, Hytti L, Nelson KA, Torondel B, Francis SC, Tanton C, et al. Menstrual health interventions, schooling, and mental health problems among Ugandan students (MENISCUS): study protocol for a school-based cluster-randomised trial. Trials [Internet]. 2022 Sep 7 [cited 2024 Apr 17];23(1):759. Available from: <https://trialsjournal.biomedcentral.com/articles/10.1186/s13063-022-06672-4> | Wrong intervention |
| Kavlakci M, Ogce F, Yavan T. The effects of playing digital games on children’s pain, fear, and anxiety levels during suturing: A randomized controlled study. Turk J Emerg Med [Internet]. 2023 [cited 2024 Apr 4];23(3):162. Available from: <https://journals.lww.com/10.4103/tjem.tjem_8_23> | Wrong intervention |
| Komro KA, Kominsky TK, Skinner JR, Livingston MD, Livingston BJ, Avance K, et al. Study protocol for a cluster randomized trial of a school, family, and community intervention for preventing drug misuse among older adolescents in the Cherokee Nation. Trials [Internet]. 2022 Dec [cited 2024 Apr 17];23(1):175. Available from: <https://trialsjournal.biomedcentral.com/articles/10.1186/s13063-022-06096-0> | Wrong setting |
| Larrivey V, Neva J, Finn K, Sikorskii A, Familiar-Lopez I, Ucheagwu V, et al. Daily Training efficiency during computerized cognitive rehabilitation training (CCRT): an analysis from a randomized trial in Ugandan children with and without severe malaria. Child Neuropsychology [Internet]. 2022 Feb 17 [cited 2024 Apr 17];28(2):197–211. Available from: <https://www.tandfonline.com/doi/full/10.1080/09297049.2021.1962266> | Wrong outcome |
| Lee MB, Yeom YO, Kim MS, Lee Y, Kim KM, Kim DH, et al. Effects of school sandplay group therapy on children victims of cyberbullying. Medicine [Internet]. 2023 Apr 7 [cited 2024 Apr 4];102(14):e33469. Available from: <https://journals.lww.com/10.1097/MD.0000000000033469> | Wrong study design |
| Osborn TL, Venturo-Conerly KE, Arango G. S, Roe E, Rodriguez M, Alemu RG, et al. Effect of Shamiri Layperson-Provided Intervention vs Study Skills Control Intervention for Depression and Anxiety Symptoms in Adolescents in Kenya: A Randomized Clinical Trial. JAMA Psychiatry [Internet]. 2021 Aug 1 [cited 2024 Apr 17];78(8):829. Available from: <https://jamanetwork.com/journals/jamapsychiatry/fullarticle/2780659> | Wrong population |
| Rojas-Barahona CA, Gaete J, Véliz M, Castillo RD, Ramírez S, Araya R. The effectiveness of a tablet-based video game that stimulates cognitive, emotional, and social skills in developing academic skills among preschoolers: study protocol for a randomized controlled trial. Trials [Internet]. 2022 Nov 9 [cited 2024 Apr 17];23(1):936. Available from: <https://trialsjournal.biomedcentral.com/articles/10.1186/s13063-022-06875-9> | Wrong intervention |
| Saw JA, Tam CL, Thanzami V, Bonn G. Contextualized School-Based Cognitive Behavioral Therapy (CBT) Intervention for Malaysian Secondary School Students. Front Psychiatry [Internet]. 2020 Dec 21 [cited 2024 Apr 17];11:565896. Available from: <https://www.frontiersin.org/articles/10.3389/fpsyt.2020.565896/full> | Wrong intervention |
| Sensoy Bahar O, Boateng A, Nartey PB, Ibrahim A, Kumbelim K, Nabunya P, et al. “ANZANSI Program Taught Me Many Things in Life”: Families’ Experiences with a Combination Intervention to Prevent Adolescent Girls’ Unaccompanied Migration for Labor. IJERPH [Internet]. 2022 Oct 13 [cited 2024 Apr 17];19(20):13168. Available from: <https://www.mdpi.com/1660-4601/19/20/13168> | Wrong outcome |
| Sun J, Liu M, Li X, Zhou Y, Li Y. Effectiveness of Group Parent-Child Interaction Therapy on Problem Behaviors in Chinese Kindergartners. IJERPH [Internet]. 2023 Feb 15 [cited 2024 Apr 17];20(4):3446. Available from: <https://www.mdpi.com/1660-4601/20/4/3446> | Wrong outcome |
| Ugwu GC, Ugwuanyi CS, Okeke CIO, Uzodinma UE, Aneke AO. Efficacy of Rational Emotive Behavior Therapy on Depression Among Children with Learning Disabilities: Implications for Evaluation in Science Teaching. J Rat-Emo Cognitive-Behav Ther [Internet]. 2022 Jun [cited 2024 Apr 17];40(2):313–33. Available from: <https://link.springer.com/10.1007/s10942-021-00417-z> | Wrong population |
| Ugwuanyi CS, Ede MO, Onyishi CN, Ossai OV, Nwokenna EN, Obikwelu LC, et al. Effect of cognitive-behavioral therapy with music therapy in reducing physics test anxiety among students as measured by generalized test anxiety scale. Medicine [Internet]. 2020 Apr [cited 2024 Apr 17];99(17):e16406. Available from: <https://journals.lww.com/10.1097/MD.0000000000016406> | Wrong intervention |
| Vanderburg JL, Dukpa C, Rauniyar AK, Giri P, Bhattarai S, Thapa A, et al. Exploring Mental Health and Academic Outcomes of Children Receiving Non-manualized, Transdiagnostic, Task-Shifted Mental Health Care From Their Teachers in a Low-and-Middle Income Country. Front Pediatr [Internet]. 2022 Mar 21 [cited 2024 Apr 17];10:807178. Available from: <https://www.frontiersin.org/articles/10.3389/fped.2022.807178/full> | Wrong intervention |
| Venturo-Conerly KE, Johnson NE, Osborn TL, Puffer ES, Rusch T, Ndetei DM, et al. Long-term health outcomes of adolescent character strength interventions: 3- to 4-year outcomes of three randomized controlled trials of the Shamiri program. Trials [Internet]. 2022 Dec [cited 2024 Apr 17];23(1):443. Available from: <https://trialsjournal.biomedcentral.com/articles/10.1186/s13063-022-06394-7> | Wrong population |
| Waechter R, Evans R, Fernandes M, Bailey B, Holmes S, Murray T, et al. A Community-based Responsive Caregiving Program Improves Neurodevelopment in Two-year Old Children in a Middle-Income Country, Grenada, West Indies. Psychosocial Intervention [Internet]. 2022 May 13 [cited 2024 Apr 17];31(2):97–107. Available from: <https://journals.copmadrid.org/pi/art/pi2022a6> | Wrong outcome |
| Zhao Y, Pan Q. Effect of social-psychological intervention on self-efficacy, social adaptability and quality of life of internet-addicted teenagers. Psychiat Danub [Internet]. 2022 Oct 17 [cited 2024 Apr 17];34(3):490–6. Available from: <https://www.psychiatria-danubina.com/UserDocsImages/pdf/dnb_vol34_no3/dnb_vol34_no3_490.pdf> | Wrong outcome |
| Watanabe, K., Tran, T. T. T., Sripo, N., Sakuraya, A., Imamura, K., Boonyamalik, P., Sasaki, N., Tienthong, T., Asaoka, H., Iida, M., Nguyen, Q. T., Nguyen, N. T., Vu, S. T., Ngo, T. T., Luyen, T. T., Nguyen, L. D., Nguyen, N. T. V., Nguyen, B. T., Matsuyama, Y., Takemura, Y., … Kawakami, N. (2024). Effectiveness of a Smartphone-Based Stress Management Program for Depression in Hospital Nurses During COVID-19 in Vietnam and Thailand: 2-Arm Parallel-Group Randomized Controlled Trial. *Journal of medical Internet research*, *26*, e50071. https://doi.org/10.2196/50071 | Wrong intervention |
| Yu, J., Wei, Z., Wells, J. C., & Fewtrell, M. (2023). Effects of relaxation therapy on maternal psychological status and infant growth following late preterm and early-term delivery: a randomized controlled trial. *The American journal of clinical nutrition*, *117*(2), 340–349. https://doi.org/10.1016/j.ajcnut.2022.12.002 | Wrong intervention |
| Skeen, S., Marlow, M., du Toit, S., Melendez-Torres, G. J., Mudekunye, L., Mapalala, E., Ngoma, K., Ntanda, B. M., Maketha, M., Grieve, C., Hartmann, L., Gordon, S., & Tomlinson, M. (2023). Using WhatsApp support groups to promote responsive caregiving, caregiver mental health and child development in the COVID-19 era: A randomised controlled trial of a fully digital parenting intervention. *Digital health*, *9*, 20552076231203893. https://doi.org/10.1177/20552076231203893 | Wrong intervention |
| Stark, L., Meinhart, M., Hermosilla, S., Kajungu, R., Cohen, F., Agaba, G. S., Obalim, G., Knox, J., & Onyango Mangen, P. (2024). Improving psychosocial well-being and parenting practices among refugees in Uganda: Results of the Journey of Life effectiveness trial. *Cambridge Prisms: Global Mental Health, 11*, e42, 1–10. <https://doi.org/10.1017/gmh.2024.38> | Wrong intervention |
| Jung, A.-R., Lee, K., & Park, E.-A. (2023). Development and evaluation of the information and communication technology-based Loneliness Alleviation Program for community-dwelling older adults: A pilot study and randomized controlled trial. *Geriatric Nursing, 53*, 204-211. https://doi.org/10.1016/j.gerinurse.2023.05.002 | Wrong setting |
| Bedendo, A., Gaume, J., McCambridge, J., Noto, A. R., & Souza-Formigoni, M. L. O. (2024). Booster effects and mechanisms of web-based personalised normative feedback alcohol intervention for college students: A pragmatic randomised controlled trial. *Drug and Alcohol Dependence, 260*, 111337. https://doi.org/10.1016/j.drugalcdep.2024.111337 | Wrong intervention |
| Glass, N., Perrin, N. A., Kohli, A., et al. (2017). Randomised controlled trial of a livestock productive asset transfer programme to improve economic and health outcomes and reduce intimate partner violence in a postconflict setting. *BMJ Global Health, 2*, e000165. https://doi.org/10.1136/bmjgh-2016-000165 | Wrong intervention |
| Nguyen, A. J., Murray, S. M., Rahaman, K. S., Lasater, M. E., Barua, S., Lee, C., Schojan, M., Tonon, B., Clouin, L., & Le Roch, K. (2024). Psychosocial impacts of Baby Friendly Spaces for Rohingya refugee mothers in Bangladesh: A pragmatic cluster-randomized controlled trial. *Cambridge Prisms: Global Mental Health, 11*, e64, 1-11. <https://doi.org/10.1017/gmh.2024.58> | Wrong intervention |
| Novotni, G., Taneska, M., Novotni, A., Fischer, J., Iloski, S., Ivanovska, A., Dimitrova, V., Novotni, L., Milutinovic, M., Joksimoski, B., Chorbev, I., Hasani, S., Dogan, V., Grimmer, T., & Kurz, A. (2024). North Macedonia interprofessional dementia care (NOMAD) – personalized care plans for people with dementia and caregiver psychoeducation delivered at home by interprofessional teams. *Frontiers in Dementia, 3*, 1391471. https://doi.org/10.3389/frdem.2024.1391471 | Wrong intervention |
| Ojonuba, H. S., Abdul Rahman, H., Zaremohzzabieh, Z., & Mohd Zulkefli, N. A. (2023). The effectiveness of an empowerment education intervention for substance use reduction among inner-city adolescents in Nigeria. *International Journal of Environmental Research and Public Health, 20*(4), 3731. <https://doi.org/10.3390/ijerph20043731> | Wrong study design |
| Ozturk, C. S., & Katikol, E. (2024). Effect of mHealth-based relaxation program on stress coping and anxiety levels in mothers of children with cancer: A randomized controlled study. *Patient education and counseling*, *123*, 108247. https://doi.org/10.1016/j.pec.2024.108247 | Wrong intervention |
| Phanasathit, M., Nimnuan, C., & Lohsoonthorn, V. (2022). The Effects of Cognitive Training in Healthy Community Residing Thai Elderly: A Randomized Controlled Trial. *Psychology research and behavior management*, *15*, 3709–3720. https://doi.org/10.2147/PRBM.S383526 | Wrong population |
| Satyanarayana, V. A., Duggal, M., Jeon, S., Singh, P., Desai, A., Chandra, P. S., & Reynolds, N. R. (2024). Exploring the feasibility, acceptability and preliminary effects of a nurse delivered mhealth intervention for women living with HIV in South India: a pilot randomized controlled trial. *Archives of women's mental health*, *27*(5), 751–763. https://doi.org/10.1007/s00737-024-01462-0 | Wrong intervention |

**Appendix F** **– Characteristics of the included studies by country, population, aim, social determinants’ domain, interventions’ components, and outcomes.**

| Study ID | Country | | Population | | Aim | Psychological component | Social determinants’ component: demographic domain | Outcomes |
| --- | --- | --- | --- | --- | --- | --- | --- | --- |
| **Lachman**  **2017** | South Africa | | Parent-child dyads living in Khayelitsha | | To reduce child maltreatment in the household | **Sinovuyo Caring Families Program for Young Children**:  12-weekly sessions, group- based programme on emotional communication; nonviolent discipline strategies, mindfulness-based techniques | **Sinovuyo Caring Families Program for Young Children**:  12-weekly sessions, group- based programme on instruction-giving and household rules, keeping children safe in communities characterized by violence | Parenting stress (parenting stress index-short form), Parental depression (BDI-II)  [Adults] |
| **Skar 2021** | Colombia | | Parents of 3- to 4-years olds | | To prevent violence against children | **International Child Development Programme (ICDP):**  12 group meetings with discussions and activities related to the three dialogs for good caregiver-child interaction, namely emotions, communication, and regulation | **Violence curriculum (VC):**  6 workshops intended to sensitize and train participants on child development, impact of violence, and responsibility in safeguarding children, along with establishing a plan to prevent violence against children | Diagnosis of mental disorders (SSQ, score above clincial cut-off) |
| **Ward 2020** | South Africa | | Child-caregiver dyads reporting some problem behaviours | | To reduce the elevated risk for harsh parenting, thereby reducing child conduct problens | **Parenting program:**  The programme designed to increase positive parenting and reduce conduct problems in children. The first half of the program focused on positive relationships building through dedicated one- on-one time and positive reinforcement of desirable behavior. | **Parenting program:**  The program aimed to diminish harsh parenting. Its latter part emphasized teaching limit-setting through instructions on household rules, daily routines, and nonviolent discipline methods, such as redirection, ignoring, time-outs, and consequences to reduce undesirable behavior. | Parentig stress (PSI-SF) |
| **Brathwaite 2023** | Uganda | | 10-20 families and other extended family members, including children/students, caregivers/guardians, siblings, uncles, aunts, and grandparents | | To improve mental health of the children with disuptive behaviours disorders (we will not include children because of the disorder) | ***Amaka Amasanyufu*** (Happy Families in Luganda, local language in the study area):  16-session manualized intervention embedded within a multiple family group model and includes 6 constructs that target family-level factors such as stress and social support (e.g., parenting stress, mental health and support system for families). | ***Amaka Amasanyufu* (Happy Families):**  16-session manualized intervention embedded within a multiple family group model and includes 6 constructs that target family-level factors such as rules, responsibility, relationships, respectful communication (target parenting factors such as family organization, discipline practices, family connectedness, support, and communication) | Depressive symptoms (BSI), parenting stress (PSI-SF) |
| **Comrie-Thomson 2022** | Zimbabwe | | Couples (mens and women who were pregnant or had a child aged up to two years) | | To improve maternal mental health | **Mbereko + Men**  **(Mbereko component):**  PLA cycles grounded in problem-solving therapy. Each woman as provided with an Action Birth CArd, a goal setting tool to support planning and reflection | **Mbereko + Men (+ Men component):**  Men participated in montly one-hour group discussions, exploring health topics to those addressed in womens’s groups, gender-related challenges, reflections on norms underpinning the gendered division of labour in domestic and care work, safe sex during pregnancy, and men’s contributions to MNCH care-seeking | Symptoms of depression and anxiety (EPDS) |
| **Miller 2023** | Lebanon | | Syrian refugees caregivers and their child | | To improve parenting | **Caregiver Support Intervention (CSI):**  Focus on caregiver wellbeing through stress management strategies and mindfulness techniques | **Caregiver Support Intervention (CSI):**  Focus on increasing positive parent-child interactions and on decreasing the use of harsh parenting practices | Caregiver psychological distress (K10) |
| **Sapkota 2022** | Nepal | | Pregnant women, victims of domestic and family violence (DFV) | | To improve the mental health of abused women | **Single session counselling and education intervention:**  Participants learned a basic problem-solving approach and common stress management techniques. The intervention involved motivational interviewing. Women could consult with the counselor for additional support in creating a safety plan or maintaining their health and well-being | **Single session counselling and education intervention:**  Participants received information on DFV, its common types, and potential mental health impacts, while enhancing their social support. Women were informed that the individual perpetrating violence should feel shame and hold responsibility for halting or preventing it, rather than expecting the victim to stay in contact or a relationship due to external circumstances | Anxiety and depressive symptoms (HADS) |
| **Donenberg 2021** | South Africa | | South African adolescent girls(15-19 yo) and their female caregivers | | to test a culturally adapted family-based HIV prevention program on anxiety, depression and trauma | **“Informed Motivated Aware and Responsible Adolescents” (IMARA-SA):**  Caregivers identify triggers (e.g., people, places, moods) of risk behavior and create personalized plans to manage them. The curriculum emphasizes the impact of mental distress on SRH and teaches strategies to manage emotions. Girls and their caregivers recognize when their emotions are in “hot zone” and practice strategies to “cool off” | **“Informed Motivated Aware and Responsible Adolescents” (IMARA-SA):**  IMARA-SA curriculum is designed to strengthen family relationships (mothers and girls) and communication about HIV prevention, safer sexual behavior, use of condoms, improve caregiver monitoring and encourage gender empowerment. Dyads discuss challenging topics to improve conflict negotiation and assertive communication, discuss healthy and unhealthy relationships. | Anxiety symptoms (GAD), Depressive symptoms (PHQ-9), PTSD symptoms (PC-PTSD-5), Depression (scores > 10 of PHQ-9) |
| **Friedberg 2023** * | Kenya | | Adolescents living in informal settlements | | to explore the effect of the empowerment intervention on mental health | **Empowerments elf-defense (ESD) program:**  The female students ’intervention involved empowerment, gender norms, techniques for achieving goals, and self-defense. | **Empowerments elf-defense (ESD) program:**  ESD program teach skills like awareness, verbal confrontations, and physical self-defense to reduce sexual assault. The male students’ intervention focused on gender norms and achieving positive social goals. | PTSD symptoms (CPSS), depressive symptoms (CDI), anxiety symptoms (BAI) |
| Study ID | Country | Population | | Aim | | Psychological component | Social determinants component: economic domain | Outcomes |
| **Lachman 2020** | Tanzania | Children (aged 3 - 17) and primary caregivers (age 18 or older) in the household | | To reduce child maltreatment in the household | | **Skillful parenting program:**  12-sessions group-based programme consisting of five sessions on parenting skills, two on child protection and five on family budgeting | **Agribusiness training program:**  Three workshops with small-holder farmer groups access to drought-resistant seeds, credit for farm inputs, advice to improve farming techniques and market connections | Parent depression (CESD), parenting stress (PSS) |
| **Hirani 2010** | Pakistan | Women | | To address the primary health problems: depression and violence | | **Group counseling**:  8-weeks counseling model with the following key components: stress and anger management, effective communication, active listening and supporting problem-solving | **Economic Skill Building:**  8-weeks community-derived intervention included skills for employment attainment and retention (e.g., effective communication, balancing personal and work life and time management, conflict resolution) | Depression (BDI-II) |
| **Byansi 2022** | Uganda | adolescent girls attending schools | | to prevent depressive symptoms among adolscent girls | | **Multiple family group (MFG) interventions:**  The intervention is organized on the four Rs (Rules, Responsibility, Relationships, and Respectful Communication) and two Ss (Stress and Social Support) targeting skills and family processes. Sessions included role-plays, group discussions, and family activities. The MFG intervention acknowledges poverty as a stressor impacting parenting and recognizes contextual challenges, including high rates of poverty, violence, and family loss. | **Family economic empowerment (FEE):**  Financial literacy sessions were designed to equip families with basic financial knowledge. Session one focused on the overview of financial literacy and budgeting, session two focused on saving, asset building, and asset accumulation, session three focused on bank services in the community, and session four focused on debt management, borrowing money, cost of borrowing, and the dangers of defaulting. | Depressive symptoms (BDI) |
| **Massarwi 2022** | South Africa | Parents and primary caregivers of adolescents | | to reduce parenting stress | | **Parenting program**  Program based on social learning theory to improve the parent–child relationship, family cohesion and har- mony, to promote non-violent discipline and to encourage family members to spend quality time together. All sessions used collaborative problem-solving techniques (not didactic methods), traditional stories, role play, modelling and stress-reduction activities. | **Parenting program**  Economic components designed to improve families’ financial conditions. These focused on (1) encouraging families to save some of their earnings by presenting a short play addressing common financial challenges, (2) teaching fundamental financial skills such as budgeting and saving through visual budgeting exercises and (3) moti- vating mental commitment to saving by clearly defining family saving goals and by making a practical family financial plan. | Depressive symptoms (CESD) and distress (PSS) |
| Study ID | Country | Population | | Aim | | Psychological component | Social determinants component: neighborhood domain | Outcomes |
| **Sherman 2009** | Thailand | Young methamphetamine users | | To reduce drug use and associated sexual risks | | **Life-skills curriculum:**  Based on a skills-building approach that was largely derived from cognitive behavioural psychology, which is focused on the causes and consequences of methamphetamine use at the individual level, which specific attention to stress in the role of drug use. | **Peer educator network intervention:**  Seven 2-hour sessions to reduce methamphetamine use and risky behavior through communication skills learned via role-plays. Sessions included interactive teaching, games, and peer education homework where specific issues were discussed with identified peers (including methamphetamine users and/or sexual partners) | Depressive symptoms (CES-D) |
| **Shinde 2018** | India | Students attending government-run secondary schools | | To enhance a positive school climate | | **SEHER intervention (individual activities):**  Actions taken on an individual student level, such as problem solving-based counselling to students who self-referred or were referred by teachers for health complaints, social difficulties, nutritional problems, and academic difficulties | **SEHER intervention (whole-school activities):**  Actions taken on a whole school level addressing themes such as hygiene, bullying, mental health, substance use, reproductive and sexual health, gender and violence, rights and responsibilities, and study skills | Depression (PHQ-9) |
| **Jiang 2022** | China | Children whose one or both biological parents were HIV-positive | | to improve mental health in children affected by parental HIV | | **Child-Caregiver-Advocacy Resilience (ChildCARE) intervention:**  Based on the resilience framework, the intervention is composed by a child-level component designed to enhance multiple intrapersonal skills, such as coping, emotional regulations, and positive thinking | **Child-Caregiver-Advocacy Resilience (ChildCARE) intervention:**  ChildCARE intervention is built upon a resilience framework, describing the dynamic process that fosters positive adaptation in children facing parental HIV and associated risk factors such as poverty, stigma, and violence. It comprises a caregiver-level component aimed at improving positive parenting skills and a community-level component, involving activities and home visits by facilitators to engage with community members. The overall objective is to decrease stigma and foster community-level support for families. | Depressive symptoms (CES-CS), anxiety symptoms (CRS) |
| **Greene 2023** | Ecuador and Panamá | Migrant women | | To address social problems (interpersonal violence, xenophobia and discrimination) and psychological problems (emotional distress and sadness) | | **Entre Nosotras**  Stress management component based on WHO SH+ intervention (included audio exercises focused on skills for managing stress)  to address emotional distress and sadness | **Entre Nosotras**  Entre Nosotras is a community- and strengths-based intervention designed to mobilize social support, strengthen community con- nectedness and stimulate collective action to promote the safety and wellbeing of migrant women | Distress (K6) |
| Study ID | Country | Population | | Aim | | Psychological component | Social determinants component: environmental domain | Outcomes |
| **Dybdahl 2001** | Bosnia and Herzegovina | War affected mothers and children | | To promote the development and well-being of young children | | **Psychosocial intervention:**  Intervention based on psychoeducational approach on trauma, with an emphasis on strengthening participants’ coping strategies | **Psychosocial intervention:**  Sustain good quality interaction between caregivers and their children and sensitize caregivers by creating a warm human environment throught group discussions between mothers affected by war. | Distress (IES) |
| **O’Callaghan 2014** | Democratic Republic of Congo | War-exposed youth at risk of attack and abduction and their caregiver | | To improve mental health and psychosocial outcomes of war affected young people | | **Family-focused psychosocial intervention:**  Some of the 8 group sessions covered: psychoeducation on trauma and related stigma, relaxation techniques, brainstorming major problems in families, effective parenting. | **Family-focused psychosocial intervention:**  Some of the 8 group sessions covered: major problems in the community (e.g., war, hunger) and how to solve them, conflict resolution in the community, youth contribution to the community | Distress (CREIES-8), depressive symptoms (AYPA) |
| **James 2019** * | Haiti | Participants selected from three disaster-affected communities | | To mitigate disaster impact | | **Mental health component:**  Day 1 includes discussion about mental health and psychosocial reactions to disaster-related stress, and teaching associated coping strategies, including skills to reduce potential avoidance of disaster-related material (e.g. self-calming through breathing, grounding, mindfulness, and muscle relaxation exercises) | **Disaster preparedness component:**  On day 2, the workshop transitions to focus on disaster preparedness, including facilitated discussions regarding links between common attributions for disasters (natural causes, God’s will) and preparedness motivation. On day 3, participants practice providing disaster and mental health-related peer support to one another, including through a “mini-disaster simulation” | Depressive symptoms (ZLDSI), PTSD symptoms (MPSS), anxiety symptoms (BAI) |
| **Dhital 2019** | Nepal | adolescents studying in grades 6,7 and 8 | | to support adolescents’ mental health and hope in an earthquake-affected district | | **Phycosocial support by teachers:**  Teacher-mediated school-based intervention that covers topic such as key concepts and principles of psychosocial support, to help the adolescents cope and overcome difficult life situations, explore the feelings of the adolescents to the difficult situations, how teachers could promote a sense of security, identity, self-esteem and hope among their students in crise. | **Psychosocial support by teachers:**  Intervention which falls under the second layer of intervention as outlined in Inter-Agency Standing Committee (IASC) guideline for humanitarian setting. The layer of the intervention is focused on the subpopulation who could uphold their mental health and psychosocial well-being with timely psychosocial support in emergencies. | Depressive symptoms (Depression self-rating scale), PTSD symptoms (CPSS) |
| **Panter-Brick 2018** * | Jordan | 12 to 18 years olds in communities affected by the Syrian crisis | | to provide psychosocial support to war-affected youth | | **Advancing Adolescents (Arabic: Nubader):**  Advancing Adolescents programme is based on profound stress attainment processes. It is community-based, nonclinical programme to meet the psychosocial needs of at-risk children and improve social interactions with participatory approaches. It focuses on the practice of attunement, for developing safe emotional spaces, managing stressors, establishing healthy relationships. | **Advancing Adolescents (Arabic: Nubader)**  Advancing Adolescents programme is a structured, 8- week psychosocial intervention for adolescents in humanitarian crises. It features three elements that are widely viewed as important to support youth adjustment in contexts of complex emergencies: (a) safety – establishment of a ‘safe space’ within the community as a base for activities and site of protection; (b) support – facilitation of social support and self-expression; and (c) structured, group-based activities. | PTSD symptoms (CRIES) |
| **Yeomans 2010** | Burundi | Participants directly victimized by violence during or since the conflict onset | | To improve mental health of war affected community | | **Workshop with psychoeducation:**  Group-based intervention for 4 days based on fostering interpersonal exchange, and games to explore themes of trauma, loss, anger, trust, and the roots of violence. Psychoeducational content on the 17 symptoms of PTSD. Coping with trauma was addressed teaching relaxation skills with emphasis on repairing relationships with community members | **Workshop with psychoeducation:**  The “Healing and Reconciniling Our Communities” workshop manual emphasized that recovery from trauma lies in the restoration of the relations between community members and draws on the need for interpersonal reconciliation by means of a “neighbor-to-neighbor healing proces”s, with include cognitive and affective engagement. | Depressive symptoms (HSCL-25), PTSD symptoms (HTQ) |
| Study ID | Country | Population | | Aim | | Psychological component | Social determinants component: social and cultural domain | Outcomes |
| **Baker-Henningham 2019** | Jamaica | Classroom teachers from primary schools | | To reduce teachers’ use of violence against children and the level of class-wide child aggression | | **Violence prevention programme - IRIE Classroom Toolbox:**  The key concepts introduced were using praise in the classroom and paying attention to positive behaviour, being proactive to prevent child behaviour problems, promoting children’s social–emotional competence. | **Violence prevention programme - IRIE Classroom Toolbox:**  The key concepts introduced were teaching rules and routines, interactive storybook reading and promoting children’s active participation in teaching and learning activities. | Depressive symptoms (CESD), distress (Teacher Burnout Scale) |
| **Osborn 2020** | Kenya | all students aged 13 to 18 years | | to alleviate depressive anxiety symptoms | | **Shamiri program:**  The intervention has three active ingredients 1) growth mindset: learn the ability to grow and adapt, fostering resilience, 2) reinforce gratitude and mindfulness strategies, 3) making goals for applying their values.  As the authors says, these components originated from the psychotherapy area. | **Shamiri program - study skills control:**  Participants learned skills designed to improve abilities to study and ultimately academic performance. Participants learned tips related to skills such as note-taking, critical reading, as essay writing, as well as how to implement a “study cycle” | Depressive symptoms (PHQ-8), anxiety symptoms (GAD) |
| **Langer 1996** | Brazil | Pregnant women | | To improve perinatal health and mothers’ psychosocial conditions | | **Psychosocial support:**  The intervention, also aiming at reducing anxiety and stress, had the following components: provision of emotional support, improvement of knowledge about pregnancy and delivery. | **Psychosocial support:**  Some of the 4 components of the programme were: reinforcement of social support network and of adequate health services utilization. The “support person” (a person selected from the participants to share with her activities, helping the woman to solve problems) participated in the home visits. | Anxiety symptoms (STAI) |
| **Bhatia 2023** | India | Family members reporting that the relative had been drinking problematically | | To improve mental health in the households | | **Supporting Addiction Affected Families Effectively (SAFE):**  The following activities were included in the intervention sessions: identifying relevant stressful situations, increasing knowledge and understanding of substance use, reducing stress arising from lack of knowledge, and identifying current coping responses by exploring advantages and disadvantages. | **Supporting Addiction Affected Families Effectively (SAFE):**  The following activities were included in the intervention sessions: discussing social support, e.g., creating a social network diagram, exploring potential new sources of support, aiming to improve communication within the family. | Distress (FMI) |
| **Ranasinghe 2022** | Sri Lanka | Women with Polycystic Ovary Syndrome (PCOS) | | To provide psychological support to women with PCOS | | **Peer-led support groups:**  Sessions of the 10 weeks support group included understanding PCOS and related psychosocial issues, learning adapted coping and problem-solving skills, learning about negative emotions such as depression, identification of own strenghts, building self-confidence and self-esteem. | **Peer-led support groups:**  Sessions of the 10 weeks support group included establishing group dynamics, ground rule to promote trust and confidentiality among participants, introducing the concept of support group, sharing thoughts about being a part f the support group and identifying ways to maintain the ongoing support. | Depressive symptoms (CESD) |
| **Metzler 2023** | Uganda | South sudanese refugee children | | to investigate the effectiveness of intervention on mental health and protection of refugee adolescents | | **Toolkit intervention:**  The intervention follows 4 themes, such as emotional learning (e.g., building emotion wheel, different ways to response), and well-being and coping “feeling good” (e.g., multitasking, tree in the wind, mindful art walk). | **Toolkit intervention:**  The intervention followed standards for design and implementation of child-friendly space (CFS), themes such as “building community”, “social support: my friends and family” conducted with a closing activity, typically an indigenous song or dance. | PTSD symptoms (CPSS-5) |
| **Boobpamala 2022** | Thailand | pregnant adolescents attending an antenatal care clinic | | to improve coping skills and prevent depression during the antenatal period among adolescents | | **Early Depression Prevention Program (EDPP):**  Participants received video clips covering physical and mental changes, self-care during pregnancy, third-trimester self-care, and complications/depressive symptoms. The EDPP empowers participants through problem-solving using five strategies: 1) “Open Mind” for reality discovery and self-esteem promotion; 2) “Reinforce Positive Power” involves critical reflection and emotional awareness; 3) “Go Together” focuses on goal setting, emotional adjustment, and commitment; 4) “Go to the Future” checks competence and reaches the destination; and 5) Evaluation assesses outcomes | **Early Depression Prevention Program (EDPP):**  The EDPP emerged from a literature review on social support theory. Participants selected a family member or friend to assist during pregnancy, jointly establishing goals, self-care guidelines, and crafting a future plan. | Depressive symptoms (Antenatal Depression Scale) |
| **Fabbri 2021** | Tanzania | Students and teachers from primary and secondary schools in refugee camp | | To reduce and prevent teachers’ use of corporal punishment in the classroom | | **EmpaTeach:**  Behavioural, self-guided teacher training intervention (12 sessions) focused on empathy-building exercises and on group work to learn self-regulation techniques (including de-escalation strategies) inspired by CBT, strategy to promote wellbeing. | **EmpaTeach:**  Sessions involved positive disciplinary methods, and classroom management strategies (i.e., learning how to co-create classroom rules with students). Intervention generated social support through the group setting so that teachers could count of peers for support and advice throughout the change process. | Depressive symptoms (MFQ) |

*data were not included in the meta-analysis because they were not provided in the right format or were not available even after attempted author contact.

**Appendix G - Risk of bias of the included studies**

**Risk of bias summary – individual randomisation**

|  | **Study ID** | **Outcome** | **Domain 1:**  **Risk of bias arising from the randomization process** | **Domain 2:**  **Risk of bias due to deviations from the intended interventions** | **Domain 3: Missing outcome data** | **Domain 4:**  **Risk of bias in measurement of the outcome** | **Domain 5:**  **Risk of bias in selection of the reported result** | **Overall Risk of Bias** |
| --- | --- | --- | --- | --- | --- | --- | --- | --- |
| individual | Bhatia 2023 | PTSD symptoms | Low | Low | Low | Some concerns | Low | Some concerns |
| individual | Boopmala 2022 | Depression diagnosis | Low | Low | Low | Some concerns | Low | Some concerns |
| individual | Boopmala 2022 | Depressive symptoms | Low | Low | Low | Some concerns | Low | Some concerns |
| individual | Donenberg 2021 | Depression diagnosis | Some concerns | Low | Low | Low | Low | Some concerns |
| individual | Donenberg 2021 | Depressive symptoms | Some concerns | Low | Low | Low | Low | Some concerns |
| individual | Donenberg 2021 | Anxiety symptoms | Some concerns | Low | Low | Low | Low | Some concerns |
| individual | Donenberg 2021 | PTSD symptoms | Some concerns | Low | Low | Low | Low | Some concerns |
| individual | Dybdahl 2001 | PTSD diagnosis | Low | High | High | Some concerns | Some concerns | High |
| individual | Dybdahl 2001 | PTSD symptoms | Low | High | High | Some concerns | Some concerns | High |
| individual | Lachman 2017 | Depression diagnosis | Low | Low | Low | Some concerns | Some concerns | Some concerns |
| individual | Lachman 2017 | Depressive symptoms | Low | Low | Low | Some concerns | Some concerns | Some concerns |
| individual | Lachman 2017 | PTSD symptoms | Low | Low | Low | Some concerns | Some concerns | Some concerns |
| individual | Langer 1996 | Anxiety symptoms | Low | Low | Low | Some concerns | Low | Some concerns |
| individual | Metzler 2023 | PTSD symptoms | Some concerns | Some concerns | High | High | Low | High |
| individual | Miller 2023 | PTSD diagnosis | Low | Low | Low | Some concerns | Low | Some concerns |
| individual | Miller 2023 | PTSD symptoms | Low | Low | Low | Some concerns | Low | Some concerns |
| individual | O'Callaghan 2014 | Depression diagnosis | Low | Low | Low | Some concerns | Low | Some concerns |
| individual | O'Callaghan 2014 | Depressive symptoms | Low | Low | Low | Some concerns | Low | Some concerns |
| individual | O'Callaghan 2014 | PTSD diagnosis | Low | Low | Low | Some concerns | Low | Some concerns |
| individual | O'Callaghan 2014 | PTSD symptoms | Low | Low | Low | Some concerns | Low | Some concerns |
| individual | Osborn 2020 | Depression diagnosis | Low | Low | Low | Some concerns | High | High |
| individual | Osborn 2020 | Depressive symptoms | Low | Low | Low | Some concerns | High | High |
| individual | Osborn 2021 | Anxiety symptoms | Low | Low | Low | Some concerns | High | High |
| individual | Ranasinghe 2023 | Depression diagnosis | High | High | High | Some concerns | Some concerns | High |
| individual | Ranasinghe 2023 | Depressive symptoms | High | High | High | Some concerns | Some concerns | High |
| individual | Sapkota 2022 | Depression diagnosis | Low | Some concerns | Low | Some concerns | Low | Some concerns |
| individual | Sapkota 2022 | Depressive symptoms | Low | Some concerns | Low | Some concerns | Low | Some concerns |
| individual | Sapkota 2022 | Anxiety symptoms | Low | Some concerns | Low | Some concerns | Low | Some concerns |
| individual | Sherman 2009 | Depressive symptoms | Some concerns | Some concerns | Low | Some concerns | Low | Some concerns |
| individual | Skar 2021 | Depression diagnosis | Some concerns | High | High | Some concerns | Low | High |
| individual | Ward 2020 | PTSD symptoms | Low | High | Low | Some concerns | Low | High |
| individual | Ward 2020 | Depression diagnosis | Low | High | Low | Some concerns | Low | High |
| individual | Ward 2020 | Depressive symptoms | Low | High | Low | Some concerns | Low | High |
| individual | Yeomans 2010 | Depression diagnosis | Low | Some concerns | Low | Some concerns | Low | Some concerns |
| individual | Yeomans 2010 | Depressive symptoms | Low | Some concerns | Low | Some concerns | Low | Some concerns |
| individual | Yeomans 2010 | PTSD diagnosis | Low | Some concerns | Low | Some concerns | Low | Some concerns |
| individual | Yeomans 2010 | PTSD symptoms | Low | Some concerns | Low | Some concerns | Low | Some concerns |

**Risk of bias summary – cluster randomisation**

|  | **Study ID** | **Outcome** | **Domain 1a:**  **Risk of bias arising from the randomization process** | **Domain 1b: Risk of bias arising from the timing of identification or recruitment of participants** | **Domain 2: Risk of bias due to deviations from the intended interventions** | **Domain 3: Missing outcome data** | **Domain 4: Risk of bias in measurement of the outcome** | **Domain 5:**  **Risk of bias in selection of the reported result** | **Overall Risk of Bias** |
| --- | --- | --- | --- | --- | --- | --- | --- | --- | --- |
| cluster | Baker-Henningham 2019 | PTSD diagnosis | Low | Low | Low | Low | Some concerns | Low | Some concerns |
| cluster | Baker-Henningham 2019 | PTSD symptoms | Low | Low | Low | Low | Some concerns | Low | Some concerns |
| cluster | Baker-Henningham 2019 | Depression diagnosis | Low | Low | Low | Low | Some concerns | Low | Some concerns |
| cluster | Baker-Henningham 2019 | Depressive symptoms | Low | Low | Low | Low | Some concerns | Low | Some concerns |
| cluster | Bhatia 2023 | PTSD symptoms | Low | Low | Low | Low | Some concerns | Low | Some concerns |
| cluster | Brathwaite 2023 | Depression diagnosis | Some concerns | Low | Low | Some concerns | Low | Some concerns | Some concerns |
| cluster | Brathwaite 2023 | Depressive symptoms | Some concerns | Low | Low | Some concerns | Low | Some concerns | Some concerns |
| cluster | Brathwaite 2023 | PTSD symptoms | Some concerns | Low | Low | Low | Low | Low | Some concerns |
| cluster | Byansi 2022 | Depressive symptoms | Low | Low | Low | Low | Low | Low | Low |
| cluster | Comrie-Thomson 2022 | Depression diagnosis | Low | Low | Low | Low | Low | Low | Low |
| cluster | Comrie-Thomson 2022 | Depressive symptoms | Low | Low | Low | Low | Low | Low | Low |
| cluster | Dhital 2019 | Depressive symptoms | Low | Low | Some concerns | Low | Some concerns | High | High |
| cluster | Dhital 2019 | PTSD symptoms | Low | Low | Some concerns | Low | Some concerns | High | High |
| cluster | Fabbri 2021 | Depressive symptoms | Low | Low | Some concerns | Low | Some concerns | Low | Some concerns |
| cluster | Greene 2023 | PTSD symptoms | Low | Low | Low | Low | Some concerns | Low | Some concerns |
| cluster | Hirani 2010 | Depression diagnosis | Some concerns | Some concerns | Some concerns | Low | Some concerns | Low | Some concerns |
| cluster | Hirani 2010 | Depressive symptoms | Some concerns | Some concerns | Some concerns | Low | Some concerns | Low | Some concerns |
| cluster | Jiang 2022 | Depressive symptoms | High | Low | Some concerns | Low | Low | Low | High |
| cluster | Jiang 2022 | Anxiety symptoms | High | Low | Some concerns | Low | Low | Low | High |
| cluster | Lachman 2020 | Depression diagnosis | Low | Low | Low | Low | Some concerns | Low | Some concerns |
| cluster | Lachman 2020 | Depressive symptoms | Low | Low | Low | Low | Some concerns | Low | Some concerns |
| cluster | Lachman 2020 | PTSD symptoms | Low | Low | Low | Low | Some concerns | Low | Some concerns |
| cluster | Massarwi 2022 | PTSD symptoms | Low | Low | Low | Low | Some concerns | Low | Some concerns |
| cluster | Massarwi 2022 | Depressive symptoms | Low | Low | Low | Low | Some concerns | Low | Some concerns |
| cluster | Shinde 2018 | Depressive symptoms | Low | Low | Low | Some concerns | Some concerns | Low | Some concerns |

**Risk of bias tables**

Notes: Y = yes; N = no; PY = probably yes; PN = probably no; NI = no information.

| **Study ID** | **Boobpmala 2022** |
| --- | --- |
| *Experimental* | **Early Depression Prevention Program** |
| *Comparator* | **Usual care** |
| Outcome | Depressive symptoms |
| **Domain 1: Risk of bias arising from the randomization process** |  |
| 1.1 | PY |
| 1.2 | PY |
| Note for 1.1&1.2 | Quote: "participants at each hospital were matched by age,  gravidarum, gestational age, planned/unplanned  pregnancy and spouse cohabitation, and randomly  assigned to the experimental or control group  equally." Quote: "This study applied a single-blind technique in which the participants and the staff were unaware of group assignments." |
| 1.3 | N |
| Note for 1.3 | Quote: "At baseline, there were no significant differences in general socio-demographic characteristics and the backgrounds between the experimental and control groups." |
| 1.0 Assessor's Judgement | Low |
| **Domain 2: Risk of bias due to deviations from the intended interventions** |  |
| 2.1 | N |
| 2.2 | N |
| Note for 2.1&2.2 | Quote: "This study applied a single-blind technique in which the participants and the staff were unaware of group assignments." |
| 2.3 | NA |
| 2.4 | NA |
| 2.5 | NA |
| 2.6 | PY |
| Note for 2.6 | No explicit mention of the analysis used to estimate the effect of assignment to intervention but 72 participants (out of 78 rendomized participants) were analyzed. |
| 2.7 | NA |
| 2.0 Assessor's Judgement | Low |
| **Domain 3: Missing outcome data** |  |
| 3.1 | Y |
| Note for 3.1 | The drop out rate was around 7% |
| 3.2 | NA |
| 3.3 | NA |
| Note for 3.3&3.4 | 0 |
| 3.4 | NA |
| 3.0 Assessor's judgement | Low |
| **Domain 4: Risk of bias in measurement of the outcome** |  |
| 4.1 | N |
| Note for 4.1 | Quote: "back-translated into Thai by Vacharaporn et al.33 and permission from this Thai translator was obtained to use the instrument  in this study. The questionnaire was revised to be suitable for a pregnant adolescent group before it was used in this study. (...) The instrument was reviewed for content validity by the above five experts. The result of CVI was .90. The internal consistency reliability was tested with 30 participants who met the same inclusion criteria as the study participants, which yielded an acceptable Cronbach’s alpha coefficient at .77 for the pilot sample, and .80 for this main study." |
| 4.2 | N |
| Note for 4.2 | No evidence to suggest differences in measurement between intervention and control |
| 4.3 | PN |
| Note for 4.3 | Outcome assessors (participants) were not aware of the intervention allocation. |
| 4.4 | NA |
| 4.5 | NA |
| 4.0 Assessor's Judgement | Low |
| **Domain 5: Risk of bias in selection of the reported result** |  |
| 5.1 | PY |
| Note for 5.1 | No protocol available but methods and results do not show discrepancies. |
| 5.2 | PN |
| Note for 5.2 | There are outcome measurements in different time points but they are all reported in the results section. |
| 5.3 | PN |
| Note for 5.3 | No evidence to suggest analyses selection. All outcomes mentioned in the methods and results were reported |
| 5.0 Assessor's Judgement | Low |
| **Overall Risk of Bias** |  |
| Assessor's overall Judgement | Low |

| **Study ID** | **Boopmala 2022** |
| --- | --- |
| *Experimental* | **Early Depression Prevention Program** |
| *Comparator* | **Usual care** |
| Outcome | Depression diagnosis |
| **Domain 1: Risk of bias arising from the randomization process** |  |
| 1.1 | PY |
| 1.2 | PY |
| Note for 1.1&1.2 | Quote: "participants at each hospital were matched by age,  gravidarum, gestational age, planned/unplanned  pregnancy and spouse cohabitation, and randomly  assigned to the experimental or control group  equally." Quote: "This study applied a single-blind technique in which the participants and the staff were unaware of group assignments." |
| 1.3 | N |
| Note for 1.3 | Quote: "At baseline, there were no significant differences in general socio-demographic characteristics and the backgrounds between the experimental and control groups." |
| 1.0 Assessor's Judgement | Low |
| **Domain 2: Risk of bias due to deviations from the intended interventions** |  |
| 2.1 | N |
| 2.2 | N |
| Note for 2.1&2.2 | Quote: "This study applied a single-blind technique in which the participants and the staff were unaware of group assignments." |
| 2.3 | NA |
| 2.4 | NA |
| 2.5 | NA |
| 2.6 | PY |
| Note for 2.6 | No explicit mention of the analysis used to estimate the effect of assignment to intervention but 72 participants (out of 78 rendomized participants) were analyzed. |
| 2.7 | NA |
| 2.0 Assessor's Judgement | Low |
| **Domain 3: Missing outcome data** |  |
| 3.1 | Y |
| Note for 3.1 | The drop out rate was around 7% |
| 3.2 | NA |
| 3.3 | NA |
| Note for 3.3&3.4 | 0 |
| 3.4 | NA |
| 3.0 Assessor's judgement | Low |
| **Domain 4: Risk of bias in measurement of the outcome** |  |
| 4.1 | N |
| Note for 4.1 | Quote: "back-translated into Thai by Vacharaporn et al.33 and permission from this Thai translator was obtained to use the instrument  in this study. The questionnaire was revised to be suitable for a pregnant adolescent group before it was used in this study. (...) The instrument was reviewed for content validity by the above five experts. The result of CVI was .90. The internal consistency reliability was tested with 30 participants who met the same inclusion criteria as the study participants, which yielded an acceptable Cronbach’s alpha coefficient at .77 for the pilot sample, and .80 for this main study." |
| 4.2 | N |
| Note for 4.2 | No evidence to suggest differences in measurement between intervention and control |
| 4.3 | Y |
| Note for 4.3 | Outcome assessors (participants) were aware of intervention allocation because the measurement is self-report |
| 4.4 | PY |
| 4.5 | PN |
| Note for 4.4&4.5 | Knowledge of the assigned intervention could influence participant-reported outcomes, but there is no evidence to believe that it did. |
| 4.0 Assessor's Judgement | Some concerns |
| **Domain 5: Risk of bias in selection of the reported result** |  |
| 5.1 | PY |
| Note for 5.1 | No protocol available but methods and results do not show discrepancies. |
| 5.2 | PN |
| Note for 5.2 | There are outcome measurements in different time points but they are all reported in the results section. |
| 5.3 | PN |
| Note for 5.3 | No evidence to suggest analyses selection. All outcomes mentioned in the methods and results were reported |
| 5.0 Assessor's Judgement | Some concerns |
| **Overall Risk of Bias** |  |
| Assessor's overall Judgement | Some concerns |

| **Study ID** | **Metzler 2023** |
| --- | --- |
| *Experimental* | **Toolkit intervention** |
| *Comparator* | **Waiting list** |
| Outcome | PTSD symptoms |
| **Domain 1: Risk of bias arising from the randomization process** |  |
| 1.1 | Y |
| 1.2 | PY |
| Note for 1.1&1.2 | Quote: "households were randomly allocated in a 1:1:1 ratio to the Toolkit group, the Standard group, or the waitlist control group using computer-based randomization." Quote: "Participants were blinded to treatment assignment for the duration of the trial." |
| 1.3 | PY |
| Note for 1.3 | Quote: "Baseline characteristics between treatment groups were mostly well-balanced", however many participants were moved to controls. Quote: " it is possible that these new participant groups differ on unmeasured variables." |
| 1.0 Assessor's Judgement | Some concerns |
| **Domain 2: Risk of bias due to deviations from the intended interventions** |  |
| 2.1 | N |
| 2.2 | Y |
| Note for 2.1&2.2 | Quote: "Participants were blinded to treatment assignment for the duration of the trial." Due to the nature of the intervention, the personnel was aware of the treatment allocation. |
| 2.3 | PY |
| Note for 2.3 | There were 101 assigned to standard treatment moved to controls, 98 assigned to the intervention moved to control.  Quote: "due to governmen-tal closures of study intervention sites to limit COVID-19 disease transmission, many participants were unable to receive the intervention" |
| 2.4 | PN |
| Note for 2.4 | Quote: "No statistical differences were found between participants retained and those lost to follow-up on outcomes under study or sample characteristics at baseline" |
| 2.5 | NA |
| 2.6 | PY |
| Note for 2.6 | The analysis was carried out on an intention to treat basis |
| 2.7 | NA |
| 2.0 Assessor's Judgement | Some concerns |
| **Domain 3: Missing outcome data** |  |
| 3.1 | N |
| Note for 3.1 | The drop out rate was 62% |
| 3.2 | PN |
| Note for 3.2 | Quote: "To correct potential bias due to missingness, multiple imputation methods with 11 imputations were employed Rubin’s formula was used to estimate standard errors." Quote: "We also conducted several subgroup analyses by gender (girls vs. boys), ethnicity (Bari vs. ethnic minorities), and developmental stage (early vs. middle adolescence)" |
| 3.3 | PY |
| Note for 3.3&3.4 | Lost at follow-up were explained as "relocated", "moved to controls" and "unavailable for interviews", and 1 "refused assent".  Quote: "Second, attendance across the study period in both interventions was low and may be an important factor inﬂuencing outcomes for adolescents." |
| 3.4 | PY |
| 3.0 Assessor's judgement | High |
| **Domain 4: Risk of bias in measurement of the outcome** |  |
| 4.1 | PN |
| Note for 4.1 | The selected outcome method is widely validated and established. |
| 4.2 | PN |
| Note for 4.2 | No evidence to suggest differences in measurement between intervention and control. |
| 4.3 | PY |
| Note for 4.3 | Quote: "the data collection team working to support quality monitoring of the intervention could have inferred treatment status of the children given differences in activities" Self-reported measures necessitated the administration through interviews. |
| 4.4 | PY |
| Note for 4.4&4.5 | Quote: "low literacy levels combined with poor ﬂuency in one language offered on the survey across the study population necessitated the administration of self-reported instruments through interviews and switching between survey languages during interviews; both of which may interfere with measurement of outcomes." |
| 4.5 | PY |
| 4.0 Assessor's Judgement | High |
| **Domain 5: Risk of bias in selection of the reported result** |  |
| 5.1 | PY |
| Note for 5.1 | No SAP available but method and results do not show discrepancies |
| 5.2 | PN |
| Note for 5.2 | No evidence to suggest outcome selection. All outcomes mentioned in the methods and results were reported |
| 5.3 | PN |
| Note for 5.3 | No evidence to suggest analyses selection. All outcomes mentioned in the methods and results were reported |
| 5.0 Assessor's Judgement | Low |
| **Overall Risk of Bias** |  |
| Assessor's overall Judgement | High |

| **Study ID** | **Donenberg 2021** |
| --- | --- |
| *Experimental* | **Informed Motivated Aware and Responsible Adolescents** |
| *Comparator* | **Active control** |
| Outcome | Depression diagnosis |
| **Domain 1: Risk of bias arising from the randomization process** |  |
| 1.1 | PY |
| 1.2 | NI |
| Note for 1.1&1.2 | Quote: "Randomization occurred by having AGYW select their program from a  paper bag without replacement to ensure equal numbers in each arm. "  It does not mention allocation concealment. |
| 1.3 | PN |
| Note for 1.3 | Quote: "Groups were generally balanced on baseline mental health variables with two exceptions." |
| 1.0 Assessor's Judgement | Some concerns |
| **Domain 2: Risk of bias due to deviations from the intended interventions** |  |
| 2.1 | PY |
| 2.2 | PY |
| Note for 2.1&2.2 | Due to the nature of the intervention, participants and personnel were most likely aware of the treatment allocation. |
| 2.3 | N |
| Note for 2.3 | There were no deviations from the intended intervention. |
| 2.4 | NA |
| 2.5 | NA |
| 2.6 | Y |
| Note for 2.6 | The analysis was carried out on an intention to treat basis |
| 2.7 | NA |
| 2.0 Assessor's Judgement | Low |
| **Domain 3: Missing outcome data** |  |
| 3.1 | Y |
| Note for 3.1 | All randomized participants were analysed. |
| 3.2 | NA |
| 3.3 | NA |
| 3.4 | NA |
| 3.0 Assessor's judgement | Low |
| **Domain 4:**  **Risk of bias in measurement of the outcome** |  |
| 4.1 | N |
| Note for 4.1 | The selected outcome method is widely validated and established, appropriate for the local context and assessed in its psychometric properties for this sample.   Quote: "The measure showed acceptable internal consistency among AGYW in this study (α= 0.78). A score of 10 or more indicates clinical levels of depression in validation studies, with good test–retest reliability in Ethiopia and Kenya." |
| 4.2 | N |
| Note for 4.2 | No evidence to suggest differences in measurement between intervention and control. |
| 4.3 | PY |
| Note for 4.3 | Only self-reported measures |
| 4.4 | PN |
| Note for 4.4&4.5 | Knowledge of the assigned intervention could influence participant-reported outcomes, but there is no reason to believe that it did. |
| 4.5 | NA |
| 4.0 Assessor's Judgement | Low |
| **Domain 5: Risk of bias in selection of the reported result** |  |
| 5.1 | PY |
| Note for 5.1 | No protocol available but methods and results do not show discrepancies. |
| 5.2 | PN |
| Note for 5.2 | No evidence to suggest outcome selection. All outcomes mentioned in the methods and results were reported |
| 5.3 | PN |
| Note for 5.3 | No evidence to suggest analyses selection. All outcomes mentioned in the methods and results were reported |
| 5.0 Assessor's Judgement | Low |
| **Overall Risk of Bias** |  |
| Assessor's overall Judgement | Some concerns |

| **Study ID** | **Donenberg 2021** |
| --- | --- |
| *Experimental* | **Informed Motivated Aware and Responsible Adolescents** |
| *Comparator* | **Active control** |
| Outcome | Depressive symptoms |
| **Domain 1: Risk of bias arising from the randomization process** |  |
| 1.1 | PY |
| 1.2 | NI |
| Note for 1.1&1.2 | Quote: "Randomization occurred by having AGYW select their program from a  paper bag without replacement to ensure equal numbers in each arm. "  It does not mention allocation concealment. |
| 1.3 | PN |
| Note for 1.3 | Quote: "Groups were generally balanced on baseline mental health variables with two exceptions." |
| 1.0 Assessor's Judgement | Some concerns |
| **Domain 2: Risk of bias due to deviations from the intended interventions** |  |
| 2.1 | PY |
| 2.2 | PY |
| Note for 2.1&2.2 | Due to the nature of the intervention, participants and personnel were most likely aware of the treatment allocation. |
| 2.3 | N |
| Note for 2.3 | There were no deviations from the intended intervention. |
| 2.4 | NA |
| 2.5 | NA |
| 2.6 | Y |
| Note for 2.6 | The analysis was carried out on an intention to treat basis |
| 2.7 | NA |
| 2.0 Assessor's Judgement | Low |
| **Domain 3: Missing outcome data** |  |
| 3.1 | Y |
| Note for 3.1 | All randomized participants were analysed. |
| 3.2 | NA |
| 3.3 | NA |
| 3.4 | NA |
| 3.0 Assessor's judgement | Low |
| **Domain 4:**  **Risk of bias in measurement of the outcome** |  |
| 4.1 | N |
| Note for 4.1 | The selected outcome method is widely validated and established, appropriate for the local context and assessed in its psychometric properties for this sample.   Quote: "The measure showed acceptable internal consistency among AGYW in this study (α= 0.78). A score of 10 or more indicates clinical levels of depression in validation studies, with good test–retest reliability in Ethiopia and Kenya." |
| 4.2 | N |
| Note for 4.2 | No evidence to suggest differences in measurement between intervention and control. |
| 4.3 | PY |
| Note for 4.3 | Only self-reported measures |
| 4.4 | PN |
| Note for 4.4&4.5 | Knowledge of the assigned intervention could influence participant-reported outcomes, but there is no reason to believe that it did. |
| 4.5 | NA |
| 4.0 Assessor's Judgement | Low |
| **Domain 5: Risk of bias in selection of the reported result** |  |
| 5.1 | PY |
| Note for 5.1 | No protocol available but methods and results do not show discrepancies. |
| 5.2 | PN |
| Note for 5.2 | No evidence to suggest outcome selection. All outcomes mentioned in the methods and results were reported |
| 5.3 | PN |
| Note for 5.3 | No evidence to suggest analyses selection. All outcomes mentioned in the methods and results were reported |
| 5.0 Assessor's Judgement | Low |
| **Overall Risk of Bias** |  |
| Assessor's overall Judgement | Some concerns |

| **Study ID** | **Donenberg 2021** |
| --- | --- |
| *Experimental* | **Informed Motivated Aware and Responsible Adolescents** |
| *Comparator* |  |
| Outcome | Anxiety symptoms |
| **Domain 1: Risk of bias arising from the randomization process** |  |
| 1.1 | PY |
| 1.2 | NI |
| Note for 1.1&1.2 | Quote: "Randomization occurred by having AGYW select their program from a  paper bag without replacement to ensure equal numbers in each arm. "  It does not mention allocation concealment. |
| 1.3 | PN |
| Note for 1.3 | Quote: "Groups were generally balanced on baseline mental health variables with two exceptions." |
| 1.0 Assessor's Judgement | Some concerns |
| **Domain 2: Risk of bias due to deviations from the intended interventions** |  |
| 2.1 | PY |
| 2.2 | PY |
| Note for 2.1&2.2 | Participants and personnel were most likely aware of the treatment allocation |
| 2.3 | N |
| Note for 2.3 | There were no deviations from the intended intervention |
| 2.4 | NA |
| 2.5 | NA |
| 2.6 | Y |
| Note for 2.6 | The analyses were carried out on an intention to treat basis |
| 2.7 | NA |
| 2.0 Assessor's Judgement | Low |
| **Domain 3: Missing outcome data** |  |
| 3.1 | Y |
| Note for 3.1 | All randomized participants were analyzed. |
| 3.2 | NA |
| 3.3 | NA |
| 3.4 | NA |
| 3.0 Assessor's judgement | Low |
| **Domain 4: Risk of bias in measurement of the outcome** |  |
| 4.1 | N |
| Note for 4.1 | The selected outcome method is widely validated and established, appropriate for the local context and assessed in its psychometric properties for this sample.   Quote: "The measure showed acceptable internal consistency among AGYW in this study (α= 0.78). A score of 10 or more indicates clinical levels of depression in validation studies, with good test–retest reliability in Ethiopia and Kenya." |
| 4.2 | N |
| Note for 4.2 | No evidence to suggest differences in measurement between intervention and control. |
| 4.3 | PY |
| Note for 4.3 | Only self-reported measures |
| 4.4 | PN |
| Note for 4.4&4.5 | Knowledge of the assigned intervention could influence participant-reported outcomes, but there is no reason to believe that it did. |
| 4.5 | NA |
| 4.0 Assessor's Judgement | Low |
| **Domain 5: Risk of bias in selection of the reported result** |  |
| 5.1 | PY |
| Note for 5.1 | No protocol available but methods and results do not show discrepancies. |
| 5.2 | PN |
| Note for 5.2 | No evidence to suggest outcome selection. All outcomes mentioned in the methods and results were reported |
| 5.3 | PN |
| Note for 5.3 | No evidence to suggest analyses selection. All outcomes mentioned in the methods and results were reported |
| 5.0 Assessor's Judgement | Low |
| **Overall Risk of Bias** |  |
| Assessor's overall Judgement | Some concerns |

| **Study ID** | **Donenberg 2021** |
| --- | --- |
| *Experimental* | **Informed Motivated Aware and Responsible Adolescents** |
| *Comparator* | **Active control** |
| Outcome | PTSD symptoms |
| **Domain 1: Risk of bias arising from the randomization process** |  |
| 1.1 | PY |
| 1.2 | NI |
| Note for 1.1&1.2 | Quote: "Randomization occurred by having AGYW select their program from a  paper bag without replacement to ensure equal numbers in each arm. "  It does not mention allocation concealment. |
| 1.3 | PN |
| Note for 1.3 | Quote: "Groups were generally balanced on baseline mental health variables with two exceptions." |
| 1.0 Assessor's Judgement | Some concerns |
| **Domain 2: Risk of bias due to deviations from the intended interventions** |  |
| 2.1 | PY |
| 2.2 | PY |
| Note for 2.1&2.2 | Due to the nature of the intervention, participants and personnel were most likely aware of the treatment allocation. |
| 2.3 | N |
| Note for 2.3 | There were no deviations from the intended intervention. |
| 2.4 | NA |
| 2.5 | NA |
| 2.6 | Y |
| Note for 2.6 | The analysis was carried out on an intention to treat basis |
| 2.7 | NA |
| 2.0 Assessor's Judgement | Low |
| **Domain 3: Missing outcome data** |  |
| 3.1 | Y |
| Note for 3.1 | All randomized participants were analysed. |
| 3.2 | NA |
| 3.3 | NA |
| 3.4 | NA |
| 3.0 Assessor's judgement | Low |
| **Domain 4: Risk of bias in measurement of the outcome** |  |
| 4.1 | N |
| Note for 4.1 | The selected outcome method (PC-PTSD-5) is widely validated and established, appropriate for the local context and assessed in its psychometric properties for this sample. |
| 4.2 | N |
| Note for 4.2 | No evidence to suggest differences in measurement between intervention and control. |
| 4.3 | PY |
| Note for 4.3 | Only self-reported measures |
| 4.4 | PN |
| Note for 4.4&4.5 | Knowledge of the assigned intervention could influence participant-reported outcomes, but there is no reason to believe that it did. |
| 4.5 | NA |
| 4.0 Assessor's Judgement | Low |
| **Domain 5: Risk of bias in selection of the reported result** |  |
| 5.1 | PY |
| Note for 5.1 | No protocol available but methods and results do not show discrepancies. |
| 5.2 | PN |
| Note for 5.2 | No evidence to suggest outcome selection. All outcomes mentioned in the methods and results were reported |
| 5.3 | PN |
| Note for 5.3 | No evidence to suggest analyses selection. All outcomes mentioned in the methods and results were reported |
| 5.0 Assessor's Judgement | Low |
| **Overall Risk of Bias** |  |
| Assessor's overall Judgement | Some concerns |

| **Study ID** | **Byansi 2022** |
| --- | --- |
| *Experimental* | **Multiple family group (MFG) interventions** and **Family economic empowerment** |
| *Comparator* | **Usual care** |
| Outcome | Depressive symptoms |
| **Domain 1a:**  **Risk of bias arising from the randomization process** |  |
| 1a.1 | PY |
| 1a.2 | PY |
| Note for 1a.1&1a.2 | Quote: "To minimize cross-condition contamination, randomization was conducted at the school level using the random assignment feature in SPSS software by the data manager to one of three study conditions. Each of the 47 secondary schools was randomly assigned to either the control arm or one of the two treatment arms: treatment 1 and treatment 2" |
| 1a.3 | PN |
| Note for 1a.3 | Baseline characteristics appeared comparable across groups. |
| 1a.0 Assessor's Judgement | Low |
| **Domain 1b: Risk of bias arising from the timing of identification or recruitment of participants** |  |
| 1b.1 | Y |
| Note for 1b.1 | Quote: "adolescents were identified and recruited from 47 public secondary schools located in five geopolitical districts of Rakai, Kyotera, Masaka, Lwengo, and Kalungu in southern Uganda. The schools included in the study were matched and randomized" |
| 1b.2 | NA |
| 1b.3 | NI |
| Note for 1b.3 | No useful information to assess this item |
| 1b.0 Assessor's Judgement | Low |
| **Domain 2: Risk of bias due to deviations from the intended interventions** |  |
| 2.1a | Y |
| Note for 2.1a | Quote: "Caregivers and parents who were willing and interested in   the study gave written consent for the adolescent girls to participate in the study" |
| 2.1b (2.1) | PY |
| 2.2 | PY |
| Note for 2.1b&2.2 | Due to the nature of the intervention, participants and personnel were most likely to be aware of the treatment allocation. |
| 2.3 | PN |
| Note for 2.3 | There is no reason to believe it.   Quote: "due to COVID-19 lockdown restrictions, the intervention was not   delivered in three of the treatment two schools." |
| 2.4 | NA |
| 2.5 | NA |
| 2.6 | PY |
| Note for 2.6 | No explicit mention of the analysis used to estimate the effect of assignment to intervention but   Quote: "All participants that were assigned to the Multiple Family Group (MFG) intervention were included in the analysis." |
| 2.7 | NA |
| 2.0 Assessor's Judgement | Low |
| **Domain 3: Missing outcome data** |  |
| 3.1a | NI |
| Note for 3.1a | The results and table present outcome data by trial condition, not by clusters. |
| 3.1b | NI |
| Note for 3.1b | No useful information to assess the drop out rate for this outcome |
| 3.2 | PY |
| Note for 3.2 | Quote: "Only three percent (n=41) of cases were missing at 12 months,which is an acceptable level of missing data (less than 10% [Bennett, 2001])" |
| 3.3 | NA |
| 3.4 | NA |
| 3.0 Assessor's judgement | Low |
| **Domain 4: Risk of bias in measurement of the outcome** |  |
| 4.1 | PN |
| Note for 4.1 | The selected outcome method is widely etablished and validated |
| 4.2 | N |
| Note for 4.2 | No evidence to suggest differences in measurement between intervention and control |
| 4.3a | Y |
| Note for 4.3a | Yes, because they signed an informed consent. |
| 4.3b | PY |
| Note for 4.3b | Only self-reported measures and participants were most likely aware of the group allocation due to the nature of the intervention |
| 4.4 | PN |
| 4.5 | NA |
| Note for 4.4&4.5 | Knowledge of the assigned intervention could influence the outcome but there is no reason to believe that it did |
| 4.0 Assessor's Judgement | Low |
| **Domain 5:**  **Risk of bias in selection of the reported result** |  |
| 5.1 | PY |
| Note for 5.1 | No protocol available, but no evidence to suggest otherwise. Methods and results do not show discrepancies. |
| 5.2 | PN |
| Note for 5.2 | No protocol available but no evidence to suggest outcome selection. All outcomes mentioned in the methods and results were reported. |
| 5.3 | PN |
| Note for 5.3 | No protocol available but no evidence to suggest analyses selection. All outcomes mentioned in the methods and results were reported. |
| 5.0 Assessor's Judgement | Low |
| **Overall Risk of Bias** |  |
| Assessor's overall Judgement | Low |

| **Study ID** | **Jiang 2022 depressive symptoms** |
| --- | --- |
| *Experimental* | **Child-Caregiver-Advocacy Resilience (ChildCARE) intervention** |
| *Comparator* | 0 |
| Outcome | Depressive symptoms |
| **Domain 1a:**  **Risk of bias arising from the randomization process** |  |
| 1a.1 | PY |
| 1a.2 | NI |
| Note for 1a.1&1a.2 | Quote: "Participating children were clustered by the schools which they were currently attending. The school clusters (n =45) then served as the unit of randomization for assignment to the control group or one of the three intervention groups". |
| 1a.3 | PY |
| Note for 1a.3 | There were significant differences between intervention and control groups for 3 baseline characteristics. Since the randomization procedure is not well explained, it could have led to those imbalances. Quote: "Although cluster randomization could help reduce contamination between the intervention and control groups, it resulted in some significant differences in baseline demographic variables (e.g., age) across intervention assignments." |
| 1a.0 Assessor's Judgement | High |
| **Domain 1b: Risk of bias arising from the timing of identification or recruitment of participants** |  |
| 1b.1 | PY |
| Note for 1b.1 | Quote: "Randomly invited eligible children and their caregivers to participate in the study. (...) When multiple children from a family met the recruitment criteria, researchers randomly selected one to participate. The recruitment process was repeated until achieving the target sample size" |
| 1b.2 | NA |
| 1b.3 | NI |
| Note for 1b.3 | No useful information to assess this item |
| 1b.0 Assessor's Judgement | Low |
| **Domain 2: Risk of bias due to deviations from the intended interventions** |  |
| 2.1a | Y |
| Note for 2.1a | Quote: "Prior to participation, appropriate informed consent was obtained from all children and their caregivers." |
| 2.1b (2.1) | PY |
| 2.2 | Y |
| Note for 2.1b&2.2 | Personnel were most likely aware to know the assigned interventions  Quote: "Standardized training and the intervention manual were provided to intervention facilitators" |
| 2.3 | NI |
| 2.4 | NA |
| 2.5 | NA |
| 2.6 | PY |
| Note for 2.6 | The analysis was carried out on an intention to treat basis |
| 2.7 | NA |
| 2.0 Assessor's Judgement | Some concerns |
| **Domain 3: Missing outcome data** |  |
| 3.1a | NI |
| Note for 3.1a | No useful information to assess this item |
| 3.1b | Y |
| Note for 3.1b | Quote: "Data for three mental health outcomes were missing for 6.8% of children at 6 months, 14.2% at 12 months, and 16.6% at 18 months. " |
| 3.2 | PY |
| Note for 3.2 | Quote: "Attrition analyses showed no signiﬁcant differences in baseline mental health outcomes by intervention assignment among children with missing data at each follow-up" |
| 3.3 | NA |
| 3.4 | NA |
| 3.0 Assessor's judgement | Low |
| **Domain 4: Risk of bias in measurement of the outcome** |  |
| 4.1 | N |
| Note for 4.1 | The selected outcome method is widely validated and established, appropriate for the ocal context and assessed in its psychometric properties for this sample.  Quote: "The reliability and validity of the CES-DC have been established in a large sample of children from urban and rural areas in China. (...) The Cronbach αs for the CES-DC were 0.62 at baseline, 0.69 at 6 months, 0.72 at 12 months, and 0.73 at 18 months." |
| 4.2 | PN |
| Note for 4.2 | No evidence to suggest differences in measurement between intervention and control |
| 4.3a | Y |
| Note for 4.3a | Quote: "Prior to participation, appropriate informed consent was obtained from all children and their caregivers." |
| 4.3b | NI |
| 4.4 | PN |
| 4.5 | NA |
| Note for 4.4&4.5 | Knowledge of the assigned intervention could influence participant-reported outcomes, but there is no reason to believe that it did |
| 4.0 Assessor's Judgement | Low |
| **Domain 5:**  **Risk of bias in selection of the reported result** |  |
| 5.1 | PY |
| Note for 5.1 | No protocol and/or SAP available, but methods and results do not show discrepancies. |
| 5.2 | PN |
| Note for 5.2 | No evidence to suggest outcome selection even if there are outcome assessments at different time points. All outcomes mentioned in the methods and results were reported. |
| 5.3 | PN |
| Note for 5.3 | No evidence to suggest analyses selection. All outcomes mentioned in the methods and results were reported. |
| 5.0 Assessor's Judgement | Low |
| **Overall Risk of Bias** |  |
| Assessor's overall Judgement | High |

| **Study ID** | **Jiang 2022 anxiety symptoms** |
| --- | --- |
| *Experimental* | **Child-Caregiver-Advocacy Resilience (ChildCARE) intervention** |
| *Comparator* | 0 |
| Outcome | Anxiety symptoms |
| **Domain 1a:**  **Risk of bias arising from the randomization process** |  |
| 1a.1 | PY |
| 1a.2 | NI |
| Note for 1a.1&1a.2 | Quote: "Participating children were clustered by the schools which they were currently attending. The school clusters (n =45) then served as the unit of randomization for assignment to the control group or one of the three intervention groups". |
| 1a.3 | PY |
| Note for 1a.3 | There were significant differences between intervention and control groups for 3 baseline characteristics. Since the randomization procedure is not well explained, it could have led to those imbalances. Quote: "Although cluster randomization could help reduce contamination between the intervention and control groups, it resulted in some significant differences in baseline demographic variables (e.g., age) across intervention assignments." |
| 1a.0 Assessor's Judgement | High |
| **Domain 1b: Risk of bias arising from the timing of identification or recruitment of participants** |  |
| 1b.1 | PY |
| Note for 1b.1 | Quote: "Randomly invited eligible children and their caregivers to participate in the study. (...) When multiple children from a family met the recruitment criteria, researchers randomly selected one to participate. The recruitment process was repeated until achieving the target sample size" |
| 1b.2 | NA |
| 1b.3 | NI |
| Note for 1b.3 | No useful information to assess this item |
| 1b.0 Assessor's Judgement | Low |
| **Domain 2: Risk of bias due to deviations from the intended interventions** |  |
| 2.1a | Y |
| Note for 2.1a | Quote: "Prior to participation, appropriate informed consent was obtained from all children and their caregivers." |
| 2.1b (2.1) | PY |
| 2.2 | Y |
| Note for 2.1b&2.2 | Personnel were most likely aware to know the assigned interventions  Quote: "Standardized training and the intervention manual were provided to intervention facilitators" |
| 2.3 | NI |
| 2.4 | NA |
| 2.5 | NA |
| 2.6 | PY |
| Note for 2.6 | The analysis was carried out on an intention to treat basis |
| 2.7 | NA |
| 2.0 Assessor's Judgement | Some concerns |
| **Domain 3: Missing outcome data** |  |
| 3.1a | NI |
| Note for 3.1a | No useful information to assess this item |
| 3.1b | PY |
| Note for 3.1b | Quote: "Data for three mental health outcomes were missing for 6.8% of children at 6 months, 14.2% at 12 months, and 16.6% at 18 months. " |
| 3.2 | PY |
| Note for 3.2 | Quote: "Attrition analyses showed no signiﬁcant differences in baseline mental health outcomes by intervention assignment among children with missing data at each follow-up" |
| 3.3 | NA |
| 3.4 | NA |
| 3.0 Assessor's judgement | Low |
| **Domain 4: Risk of bias in measurement of the outcome** |  |
| 4.1 | PN |
| Note for 4.1 | The selected outcome method has been validated in previous studies and demonstrated good psychometric properties for this sample. Quote: "School anxiety was measured using the 6-item anxiety/withdrawal subscale adapted from the Child Rating Scale. The CRS has been applied in previous studies among children affected by parental HIV in  China. (...)The Cronbach αs were 0.77 at baseline, 0.79 at 6 months, 0.83 at 12 months, and 0.84 at 18 months." |
| 4.2 | PN |
| Note for 4.2 | No evidence to suggest differences in measurement between intervention and control |
| 4.3a | Y |
| Note for 4.3a | Quote: "Prior to participation, appropriate informed consent was obtained from all children and their caregivers." |
| 4.3b | NI |
| 4.4 | PN |
| 4.5 | NA |
| Note for 4.4&4.5 | Knowledge of the assigned intervention could influence participant-reported outcomes, but there is no reason to believe that it did |
| 4.0 Assessor's Judgement | Low |
| **Domain 5:**  **Risk of bias in selection of the reported result** |  |
| 5.1 | PY |
| Note for 5.1 | No protocol and/or SAP available, but methods and results do not show discrepancies. |
| 5.2 | PN |
| Note for 5.2 | No evidence to suggest outcome selection even if there are outcome assessments at different time points. All outcomes mentioned in the methods and results were reported. |
| 5.3 | PN |
| Note for 5.3 | No evidence to suggest analyses selection. All outcomes mentioned in the methods and results were reported. |
| 5.0 Assessor's Judgement | Low |
| **Overall Risk of Bias** |  |
| Assessor's overall Judgement | High |

| **Study ID** | **Brathwaite 2023** |
| --- | --- |
| *Experimental* | ***Amaka Amasanyufu* (Happy Families in Luganda, local language in the study area)** |
| *Comparator* | 0 |
| Outcome | Depression diagnosis |
| **Domain 1a:**  **Risk of bias arising from the randomization process** |  |
| 1a.1 | PY |
| 1a.2 | NI |
| Note for 1a.1&1a.2 | Quote: "42 eligible schools was created from among those that expressed interest. A total of 30 schools were then randomly selected. To avoid contamination, entire schools were randomly assigned to 1 of the following 3 study groups" |
| 1a.3 | PN |
| Note for 1a.3 | Baseline characteristics appeared comparable across groups. |
| 1a.0 Assessor's Judgement | Some concerns |
| **Domain 1b: Risk of bias arising from the timing of identification or recruitment of participants** |  |
| 1b.1 | NI |
| Note for 1b.1 | No information provided about the recruitment procedure at individual level.The authors only said that participants were recruited within each school. |
| 1b.2 | PN |
| Note for 1b.2 | There is no reason to believe it |
| 1b.3 | PN |
| Note for 1b.3 | There is no reason to believe it |
| 1b.0 Assessor's Judgement | Low |
| **Domain 2: Risk of bias due to deviations from the intended interventions** |  |
| 2.1a | PY |
| Note for 2.1a | Quote: "caregiver provided written consent and child provided assent to participate. " |
| 2.1b (2.1) | PN |
| 2.2 | PY |
| Note for 2.1b&2.2 | Due to the nature of the intervention, participants and personnel were most likely aware of the treatment allocation. |
| 2.3 | PN |
| Note for 2.3 | Quote: "When COVID-19 started in Uganda, the study had not started baseline assessments and delivery of the intervention in 4 treatment schools" |
| 2.4 | NA |
| 2.5 | NA |
| 2.6 | PY |
| Note for 2.6 | No explicit mention of the analysis used to estimate the effect of assignment to intervention but methods and results do not show discrepancies |
| 2.7 | NA |
| 2.0 Assessor's Judgement | Low |
| **Domain 3: Missing outcome data** |  |
| 3.1a | PY |
| Note for 3.1a | No school loss at follow-up. |
| 3.1b | PY |
| Note for 3.1b | The drop out rate was 6.6% at 16weeks (T3) |
| 3.2 | NA |
| 3.3 | NA |
| 3.4 | NA |
| 3.0 Assessor's judgement | Low |
| **Domain 4: Risk of bias in measurement of the outcome** |  |
| 4.1 | PN |
| Note for 4.1 | The measure showed good psychometric properties within this sample |
| 4.2 | N |
| Note for 4.2 | No evidence to suggest differences in measurement between intervention and control |
| 4.3a | PY |
| Note for 4.3a | Quote: "caregiver provided written consent and child provided assent to participate." |
| 4.3b | PY |
| Note for 4.3b | Only self-reported measures |
| 4.4 | PY |
| 4.5 | PN |
| Note for 4.4&4.5 | Knowledge of the assigned intervention could influence the outcome but there is no reason to believe that it did |
| 4.0 Assessor's Judgement | Some concerns |
| **Domain 5:**  **Risk of bias in selection of the reported result** |  |
| 5.1 | PY |
| Note for 5.1 | No SAP available but no evidence to suggest it. Methods and results do not show discrepancies. |
| 5.2 | PN |
| Note for 5.2 | Outcome assessments at different time points but no evidence to suggest outcome selection. They are all reported. |
| 5.3 | PN |
| Note for 5.3 | No evidence to suggest analyses selection. All outcomes mentioned in the methods and results were reported. |
| 5.0 Assessor's Judgement | Low |
| **Overall Risk of Bias** |  |
| Assessor's overall Judgement | Some concerns |

| **Study ID** | **Brathwaite 2023** |
| --- | --- |
| *Experimental* | ***Amaka Amasanyufu* (Happy Families in Luganda, local language in the study area)** |
| *Comparator* | 0 |
| Outcome | Depressive symptoms |
| **Domain 1a:**  **Risk of bias arising from the randomization process** |  |
| 1a.1 | PY |
| 1a.2 | NI |
| Note for 1a.1&1a.2 | Quote: "42 eligible schools was created from among those that expressed interest. A total of 30 schools were then randomly selected. To avoid contamination, entire schools were randomly assigned to 1 of the following 3 study groups" |
| 1a.3 | PN |
| Note for 1a.3 | Baseline characteristics appeared comparable across groups. |
| 1a.0 Assessor's Judgement | Some concerns |
| **Domain 1b: Risk of bias arising from the timing of identification or recruitment of participants** |  |
| 1b.1 | NI |
| Note for 1b.1 | No information provided about the recruitment procedure at individual level.The authors only said that participants were recruited within each school. |
| 1b.2 | PN |
| Note for 1b.2 | There is no reason to believe it |
| 1b.3 | PN |
| Note for 1b.3 | There is no reason to believe it |
| 1b.0 Assessor's Judgement | Low |
| **Domain 2: Risk of bias due to deviations from the intended interventions** |  |
| 2.1a | PY |
| Note for 2.1a | Quote: "caregiver provided written consent and child provided assent to participate. " |
| 2.1b (2.1) | PN |
| 2.2 | PY |
| Note for 2.1b&2.2 | Due to the nature of the intervention, participants and personnel were most likely aware of the treatment allocation. |
| 2.3 | PN |
| Note for 2.3 | Quote: "When COVID-19 started in Uganda, the study had not started baseline assessments and delivery of the intervention in 4 treatment schools" |
| 2.4 | NA |
| 2.5 | NA |
| 2.6 | PY |
| Note for 2.6 | No explicit mention of the analysis used to estimate the effect of assignment to intervention but methods and results do not show discrepancies |
| 2.7 | NA |
| 2.0 Assessor's Judgement | Low |
| **Domain 3: Missing outcome data** |  |
| 3.1a | PY |
| Note for 3.1a | No school loss at follow-up. |
| 3.1b | PY |
| Note for 3.1b | The drop out rate was 6.6% at 16weeks (T3) |
| 3.2 | NA |
| 3.3 | NA |
| 3.4 | NA |
| 3.0 Assessor's judgement | Low |
| **Domain 4: Risk of bias in measurement of the outcome** |  |
| 4.1 | PN |
| Note for 4.1 | The measure showed good psychometric properties within this sample |
| 4.2 | N |
| Note for 4.2 | No evidence to suggest differences in measurement between intervention and control |
| 4.3a | PY |
| Note for 4.3a | Quote: "caregiver provided written consent and child provided assent to participate." |
| 4.3b | PY |
| Note for 4.3b | Only self-reported measures |
| 4.4 | PY |
| 4.5 | PN |
| Note for 4.4&4.5 | Knowledge of the assigned intervention could influence the outcome but there is no reason to believe that it did |
| 4.0 Assessor's Judgement | Some concerns |
| **Domain 5:**  **Risk of bias in selection of the reported result** |  |
| 5.1 | PY |
| Note for 5.1 | No SAP available but no evidence to suggest it. Methods and results do not show discrepancies. |
| 5.2 | PN |
| Note for 5.2 | Outcome assessments at different time points but no evidence to suggest outcome selection. They are all reported. |
| 5.3 | PN |
| Note for 5.3 | No evidence to suggest analyses selection. All outcomes mentioned in the methods and results were reported. |
| 5.0 Assessor's Judgement | Low |
| **Overall Risk of Bias** |  |
| Assessor's overall Judgement | Some concerns |

| **Study ID** | **Comrie-Thomson 2022** |
| --- | --- |
| *Experimental* | **Mbereko + Men (Mbereko component)** |
| *Comparator* |  |
| Outcome | Depressive symptoms |
| **Domain 1a:**  **Risk of bias arising from the randomization process** |  |
| 1a.1 | Y |
| 1a.2 | PY |
| Note for 1a.1&1a.2 | Quote: "The Mbereko+Men intervention was randomised (1:1) at the cluster level by an external researcher who had no other involvement in the trial, using a true random number sequence (online random number generator, www.random.org)" |
| 1a.3 | N |
| Note for 1a.3 | Quote: "Participants’ baseline socio-demographic characteristics were similar between study arms" |
| 1a.0 Assessor's Judgement | Low |
| **Domain 1b: Risk of bias arising from the timing of identification or recruitment of participants** |  |
| 1b.1 | PY |
| Note for 1b.1 | Quote: "First, eligible women participants were identified in consultation with village health workers through community meetings targeting all parents of young children. This was followed by snowball sampling to identify eligible women who had not attended the meetings. " |
| 1b.2 | NA |
| 1b.3 | PN |
| Note for 1b.3 | There is no reason to believe it |
| 1b.0 Assessor's Judgement | Low |
| **Domain 2: Risk of bias due to deviations from the intended interventions** |  |
| 2.1a | Y |
| Note for 2.1a | Quote: "All study participants provided written informed consent prior to their enrolment in the study" |
| 2.1b (2.1) | PY |
| 2.2 | PY |
| Note for 2.1b&2.2 | Due to the nature of the intervention, participants and personnel were most likely to be aware of the treatment allocation.  Quote: "Masking was not used" |
| 2.3 | PN |
| Note for 2.3 | All the participants followed the allocated intervention |
| 2.4 | NA |
| 2.5 | NA |
| 2.6 | PY |
| Note for 2.6 | No explicit mention of the analysis used to estimate the effect of assignment to intervention but all randomized participants were analyzed |
| 2.7 | NA |
| 2.0 Assessor's Judgement | Low |
| **Domain 3: Missing outcome data** |  |
| 3.1a | Y |
| Note for 3.1a | Quote: "There were no losses or exclusions after randomisation at cluster or individual level in either intervention or control arms" |
| 3.1b | Y |
| Note for 3.1b | Quote:"There were no losses or exclusions after randomisation at cluster or individual level in either intervention or control arms". No participants have been excluded from the analyses |
| 3.2 | NA |
| 3.3 | NA |
| 3.4 | NA |
| 3.0 Assessor's judgement | Low |
| **Domain 4: Risk of bias in measurement of the outcome** |  |
| 4.1 | N |
| Note for 4.1 | The selected outcome method is validated for use in the local context.  Quote: "The primary outcome measure was women’s mean score on the locally validated Shona-language version of the Edinburgh Postnatal Depression Scale (EPDS)" |
| 4.2 | PN |
| Note for 4.2 | No evidence to suggest differences in measurement between intervention and control |
| 4.3a | Y |
| Note for 4.3a | Outcome assessors (participants) provided the informed consent. |
| 4.3b | Y |
| Note for 4.3b | Only self-reported measures |
| 4.4 | PN |
| 4.5 | NA |
| Note for 4.4&4.5 | Knowledge of the assigned intervention could influence the outcome but there is no reason to believe that it did |
| 4.0 Assessor's Judgement | Low |
| **Domain 5:**  **Risk of bias in selection of the reported result** |  |
| 5.1 | PY |
| Note for 5.1 | No SAP available but no evidence to suggest it. Methods and results do not show discrepancies. |
| 5.2 | PN |
| Note for 5.2 | No evidence to suggest outcome selection. All outcomes mentioned in the methods and results were reported. |
| 5.3 | PN |
| Note for 5.3 | No evidence to suggest analyses selection. All outcomes mentioned in the methods and results were reported. |
| 5.0 Assessor's Judgement | Low |
| **Overall Risk of Bias** |  |
| Assessor's overall Judgement | Low |

| **Study ID** | **Comrie-Thomson 2022** |
| --- | --- |
| *Experimental* | **Mbereko + Men (Mbereko component)** |
| *Comparator* |  |
| Outcome | Depression diagnosis |
| **Domain 1a:**  **Risk of bias arising from the randomization process** |  |
| 1a.1 | Y |
| 1a.2 | PY |
| Note for 1a.1&1a.2 | Quote: "The Mbereko+Men intervention was randomised (1:1) at the cluster level by an external researcher who had no other involvement in the trial, using a true random number sequence (online random number generator, www.random.org)" |
| 1a.3 | N |
| Note for 1a.3 | Quote: "Participants’ baseline socio-demographic characteristics were similar between study arms" |
| 1a.0 Assessor's Judgement | Low |
| **Domain 1b: Risk of bias arising from the timing of identification or recruitment of participants** |  |
| 1b.1 | PY |
| Note for 1b.1 | Quote: "First, eligible women participants were identified in consultation with village health workers through community meetings targeting all parents of young children. This was followed by snowball sampling to identify eligible women who had not attended the meetings. " |
| 1b.2 | NA |
| Note for 1b.2 | 0 |
| 1b.3 | PN |
| Note for 1b.3 | There is no reason to believe it |
| 1b.0 Assessor's Judgement | Low |
| **Domain 2: Risk of bias due to deviations from the intended interventions** |  |
| 2.1a | Y |
| Note for 2.1a | Quote: "All study participants provided written informed consent prior to their enrolment in the study" |
| 2.1b (2.1) | PY |
| 2.2 | PY |
| Note for 2.1b&2.2 | Due to the nature of the intervention, participants and personnel were most likely to be aware of the treatment allocation.  Quote: "Masking was not used" |
| 2.3 | PN |
| Note for 2.3 | All the participants followed the allocated intervention |
| 2.4 | NA |
| 2.5 | NA |
| 2.6 | PY |
| Note for 2.6 | No explicit mention of the analysis used to estimate the effect of assignment to intervention but all randomized participants were analyzed |
| 2.7 | NA |
| 2.0 Assessor's Judgement | Low |
| **Domain 3: Missing outcome data** |  |
| 3.1a | Y |
| Note for 3.1a | Quote:"There were no losses or exclusions after randomisation at  cluster or individual level in either intervention or control arms" |
| 3.1b | Y |
| Note for 3.1b | Quote:"There were no losses or exclusions after randomisation at  cluster or individual level in either intervention or control arms" |
| 3.2 | NA |
| 3.3 | NA |
| 3.4 | NA |
| 3.0 Assessor's judgement | Low |
| **Domain 4: Risk of bias in measurement of the outcome** |  |
| 4.1 | N |
| Note for 4.1 | The selected outcome method is validated for use in the local context.  Quote: "The primary outcome measure was women’s mean score on the locally validated Shona-language version of the Edinburgh Postnatal Depression Scale (EPDS)" |
| 4.2 | PN |
| Note for 4.2 | No evidence to suggest differences in measurement between intervention and control |
| 4.3a | Y |
| Note for 4.3a | Outcome assessors (participants) provided the informed consent. |
| 4.3b | Y |
| Note for 4.3b | Only self-reported measures |
| 4.4 | PN |
| 4.5 | NA |
| Note for 4.4&4.5 | Knowledge of the assigned intervention could influence the outcome but there is no reason to believe that it did |
| 4.0 Assessor's Judgement | Low |
| **Domain 5:**  **Risk of bias in selection of the reported result** |  |
| 5.1 | PY |
| Note for 5.1 | No SAP available but no evidence to suggest it. Methods and results do not show discrepancies. |
| 5.2 | PN |
| Note for 5.2 | No evidence to suggest outcome selection. All outcomes mentioned in the methods and results were reported. |
| 5.3 | PN |
| Note for 5.3 | No evidence to suggest analyses selection. All outcomes mentioned in the methods and results were reported. |
| 5.0 Assessor's Judgement | Low |
| **Overall Risk of Bias** |  |
| Assessor's overall Judgement | Low |

| **Study ID** | **Bhatia 2022** |
| --- | --- |
| *Experimental* | **Supporting Addiction Affected Families Effectively (SAFE)** |
| *Comparator* |  |
| Outcome | PTSD symptoms |
| **Domain 1: Risk of bias arising from the randomization process** |  |
| 1.1 | Y |
| 1.2 | Y |
| Note for 1.1&1.2 | Quote: "An independent researcher developed a randomisation code using computer-generated random numbers. Sequentially Numbered Opaque Sealed Envelopes were used to maximise allocation con-cealment. The envelope was opened only after the participant information was written on its cover, which elped create an audit trail. The 3-month outcome assessments were administered by research assistants, who had no previous engagement in the trial, and were ‘blind’ to the treatment allocation." |
| 1.3 | PN |
| Note for 1.3 | Quote: "Baseline characteristics were similar between the two arms, except for educational status and help-seeking by the drinking relative. A significantly higher proportion of those in the SAFE arm completed primary/secondary education compared to those in the EUC arm (90.2% v. 74%, p = 0.03). About 20% reported that their drinking relative had received treatment in the previous 3 months, and these were disproportionately recruited into the treatment arm (27% v. 10%, p = 0.02)" |
| 1.0 Assessor's Judgement | Low |
| **Domain 2: Risk of bias due to deviations from the intended interventions** |  |
| 2.1 | Y |
| 2.2 | N |
| Note for 2.1&2.2 | Quote: "participants were requested not to disclose their allocation status during the outcome interview (...) by research assistants, who had no previous engagement in the trial, and were ‘blind’ to the treatment allocation. " |
| 2.3 | N |
| Note for 2.3 | There is no evidence to believe it |
| 2.4 | NA |
| 2.5 | NA |
| 2.6 | Y |
| Note for 2.6 | The analysis was carried out on an intervention to treat basis |
| 2.7 | NA |
| 2.0 Assessor's Judgement | Low |
| **Domain 3: Missing outcome data** |  |
| 3.1 | Y |
| Note for 3.1 | The dropout rate was 91/102 (11%) |
| 3.2 | NA |
| Note for 3.2 |  |
| 3.3 | NA |
| 3.4 | NA |
| 3.0 Algorithm result | Low |
| 3.0 Assessor's judgement | Low |
| **Domain 4: Risk of bias in measurement of the outcome** |  |
| 4.1 | N |
| Note for 4.1 | Quote: "The CQ, SRT, FMI and ADF-SSS are standardised tools and have undergone rigorous psychometric testing for reliability and validity." |
| 4.2 | PN |
| Note for 4.2 | No evidence to suggest differences in measurement between intervention and control |
| 4.3 | Y |
| Note for 4.3 | Outcome assessors (participants) were aware of intervention allocation because the measurement is self-report |
| 4.4 | PY |
| Note for 4.4&4.5 | Knowledge of the assigned intervention could influence participant-reported outcomes, but there is no reason to believe that it did |
| 4.5 | PN |
| 4.0 Assessor's Judgement | Some concerns |
| **Domain 5: Risk of bias in selection of the reported result** |  |
| 5.1 | PY |
| Note for 5.1 | Protocol is available withouth analysis plan. No evidence to suggest otherwise, methods and results do not show discrepancies. |
| 5.2 | PN |
| Note for 5.2 | No SAP available, but there is no evidence to suggest outcome selection. All outcomes mentioned in the methods and results were reported. |
| 5.3 | N |
| Note for 5.3 | No evidence to suggest analyses selection. All outcomes mentioned in the methods and results were reported. |
| 5.0 Assessor's Judgement | Low |
| **Overall Risk of Bias** |  |
| Assessor's overall Judgement | Some concerns |

| **Study ID** | **Miller 2023** |
| --- | --- |
| *Experimental* | **Caregiver Support Intervention (CSI)** |
| *Comparator* | 0 |
| Outcome | PTSD symptoms |
| **Domain 1:**  **Risk of bias arising from the randomization process** |  |
| 1.1 | Y |
| 1.2 | Y |
| Note for 1.1&1.2 | Quote: " A block randomization design was used, using a participatory methodology implemented successfully in our pilot RCT. At baseline assessment, after completing the questionnaires, one caregiver from each family was asked to draw a lollipop out of an opaque bag, ﬁlled with an equal number of red and green lollipops to ensure an equal number of CSI and waitlist control participants. After baseline data were completed, a coin toss determined the meaning of each color, CSI or WLC." |
| 1.3 | NI |
| Note for 1.3 | No information provided about differences in baseline characteristics |
| 1.0 Assessor's Judgement | Low |
| **Domain 2:**  **Risk of bias due to deviations from the intended interventions** |  |
| 2.1 | Y |
| 2.2 | Y |
| Note for 2.1&2.2 | Due to the nature of the intervention, participants and personnel were aware of treatment allocation.  Quote: "Given the nature of the study, participants and group facili  tators were not blind to group assignment." |
| 2.3 | PN |
| Note for 2.3 | There is no reason to believe it |
| 2.4 | NA |
| 2.5 | NA |
| 2.6 | PY |
| Note for 2.6 | Quote: "For the effect of CSI versus WLC on parenting at three-month  follow-up, we ﬁtted a three-level random intercept linear  regression model, deﬁning the scores at endline and three-  month follow up at level one, caregivers at level two, and  families at level three. A time by group interaction was  included to allow the effect to differ at each time point." |
| 2.7 | NA |
| 2.0 Assessor's Judgement | Low |
| **Domain 3:**  **Missing outcome data** |  |
| 3.1 | Y |
| Note for 3.1 | The dropout rate was 8% |
| 3.2 | NA |
| Note for 3.2 |  |
| 3.3 | NA |
| 3.4 | NA |
| 3.0 Assessor's judgement | Low |
| **Domain 4:**  **Risk of bias in measurement of the outcome** |  |
| 4.1 | N |
| Note for 4.1 | Quote: "has been used widely in cross-  cultural mental health research. The internal consistency of  the WEMWBS in this study was good (a = .82). Test–retest  reliability established in our formative research was also good" |
| 4.2 | PN |
| Note for 4.2 | No evidence to suggest differences in measurement between intervention and control |
| 4.3 | Y |
| Note for 4.3 | Outcome assessors (participants) were aware of intervention allocation because the measurement is self-report |
| 4.4 | PY |
| Note for 4.4&4.5 | Knowledge of the assigned intervention could influence participant-reported outcomes, but there is no evidence to believe that it did. |
| 4.5 | PN |
| 4.0 Assessor's Judgement | Some concerns |
| **Domain 5:**  **Risk of bias in selection of the reported result** |  |
| 5.1 | PY |
| Note for 5.1 | Protocol is available without analysis plan. No evidence to suggest otherwise, methods and results do not show discrepancies. |
| 5.2 | N |
| Note for 5.2 | No evidence to suggest outcome selection. All outcomes mentioned in the methods and protocol were reported. |
| 5.3 | N |
| Note for 5.3 | No evidence to suggest analyses selection. All analyses mentioned in the method and results sections were reported. |
| 5.0 Assessor's Judgement | Low |
| **Overall Risk of Bias** |  |
| Assessor's overall Judgement | Some concerns |

| **Study ID** | **Miller 2023** |
| --- | --- |
| *Experimental* | **Caregiver Support Intervention (CSI)** |
| *Comparator* | 0 |
| Outcome | PTSD diagnosis |
| **Domain 1:**  **Risk of bias arising from the randomization process** |  |
| 1.1 | Y |
| 1.2 | Y |
| Note for 1.1&1.2 | Quote: " A block randomization design was used, using a participatory methodology implemented successfully in our pilot RCT. At baseline assessment, after completing the questionnaires, one caregiver from each family was asked to draw a lollipop out of an opaque bag, ﬁlled with an equal number of red and green lollipops to ensure an equal number of CSI and waitlist control participants. After baseline data were completed, a coin toss determined the meaning of each color, CSI or WLC." |
| 1.3 | NI |
| Note for 1.3 | No information provided about differences in baseline characteristics |
| 1.0 Assessor's Judgement | Low |
| **Domain 2:**  **Risk of bias due to deviations from the intended interventions** |  |
| 2.1 | Y |
| 2.2 | Y |
| Note for 2.1&2.2 | Due to the nature of the intervention, participants and personnel were aware of treatment allocation.  Quote: "Given the nature of the study, participants and group facili  tators were not blind to group assignment." |
| 2.3 | PN |
| Note for 2.3 | There is no reason to believe it |
| 2.4 | NA |
| 2.5 | NA |
| 2.6 | PY |
| Note for 2.6 | Quote: "For the effect of CSI versus WLC on parenting at three-month  follow-up, we ﬁtted a three-level random intercept linear  regression model, deﬁning the scores at endline and three-  month follow up at level one, caregivers at level two, and  families at level three. A time by group interaction was  included to allow the effect to differ at each time point." |
| 2.7 | NA |
| 2.0 Assessor's Judgement | Low |
| **Domain 3:**  **Missing outcome data** |  |
| 3.1 | Y |
| Note for 3.1 | The dropout rate was 8% |
| 3.2 | NA |
| Note for 3.2 |  |
| 3.3 | NA |
| 3.4 | NA |
| 3.0 Assessor's judgement | Low |
| **Domain 4:**  **Risk of bias in measurement of the outcome** |  |
| 4.1 | N |
| Note for 4.1 | Quote: "has been used widely in cross-cultural mental health research. The internal consistency of the WEMWBS in this study was good (a = .82). Test–retest reliability established in our formative research was also good" |
| 4.2 | PN |
| Note for 4.2 | No evidence to suggest differences in measurement between intervention and control |
| 4.3 | Y |
| Note for 4.3 | Outcome assessors (participants) were aware of intervention allocation because the measurement is self-report |
| 4.4 | PY |
| Note for 4.4&4.5 | Knowledge of the assigned intervention could influence participant-reported outcomes, but there is no evidence to believe that it did. |
| 4.5 | PN |
| 4.0 Assessor's Judgement | Some concerns |
| **Domain 5:**  **Risk of bias in selection of the reported result** |  |
| 5.1 | PY |
| Note for 5.1 | Protocol is available without analysis plan. No evidence to suggest otherwise, methods and results do not show discrepancies. |
| 5.2 | N |
| Note for 5.2 | No evidence to suggest outcome selection. All outcomes mentioned in the methods and protocol were reported. |
| 5.3 | N |
| Note for 5.3 | No evidence to suggest analyses selection. All analyses mentioned in the method and results sections were reported. |
| 5.0 Assessor's Judgement | Low |
| **Overall Risk of Bias** |  |
| Assessor's overall Judgement | Some concerns |

| **Study ID** | **Ranasinghe 2023** |
| --- | --- |
| *Experimental* | **Peer-led support groups** |
| *Comparator* |  |
| Outcome | Depression diagnosis |
| **Domain 1:**  **Risk of bias arising from the randomization process** |  |
| 1.1 | PY |
| 1.2 | NI |
| Note for 1.1&1.2 | Quote: "Allocation to intervention and control groups was by simple random sampling"  No further information about allocation concealment. |
| 1.3 | PN |
| Note for 1.3 | The observed differences are compatible with chance. Quote: "There were no statistical significant differences of demographic and clinical parameters. The only differences were on 'menstrual problems' and 'emotions'" |
| 1.0 Assessor's Judgement | Low |
| **Domain 2:**  **Risk of bias due to deviations from the intended interventions** |  |
| 2.1 | PY |
| 2.2 | Y |
| Note for 2.1&2.2 | Due to the nature of the intervention participants were most likely aware of their assigned intervention.  Quote: "The faciliattor was familiarised about the nature and objective of the support group..." |
| 2.3 | PN |
| Note for 2.3 | There is no reason to believe it |
| 2.4 | NA |
| 2.5 | NA |
| 2.6 | PN |
| Note for 2.6 | Analyses exclude eligible trial participants post randomization. |
| 2.7 | NI |
| 2.0 Assessor's Judgement | High |
| **Domain 3:**  **Missing outcome data** |  |
| 3.1 | PN |
| Note for 3.1 | The drop-out rate is 19% with no reasons explained. |
| 3.2 | N |
| Note for 3.2 | No information to suggest that results were not biased by missing outcome data |
| 3.3 | NI |
| Note for 3.3&3.4 | Reasons for missing data are not explained. |
| 3.4 | NI |
| 3.0 Assessor's judgement | High |
| **Domain 4:**  **Risk of bias in measurement of the outcome** |  |
| 4.1 | N |
| Note for 4.1 | Quote: "The CES-D has been validated and translated in Sinhala" |
| 4.2 | PN |
| Note for 4.2 | No evidence to suggest differences in measurement between intervention and control. |
| 4.3 | Y |
| Note for 4.3 | Only self-reported measures |
| 4.4 | PY |
| Note for 4.4&4.5 | Knowledge of the assigned intervention could influence participant-reported outcomes, but there is no reason to believe that it did |
| 4.5 | PN |
| 4.0 Assessor's Judgement | Some concerns |
| **Domain 5:**  **Risk of bias in selection of the reported result** |  |
| 5.1 | N |
| Note for 5.1 | No protocol available |
| 5.2 | N |
| Note for 5.2 | No evidence to suggest outcome selection. All outcomes mentioned in the methods and results were reported |
| 5.3 | N |
| Note for 5.3 | No evidence to suggest analyses selection. All outcomes mentioned in the methods and results were reported |
| 5.0 Assessor's Judgement | Some concerns |
| **Overall Risk of Bias** |  |
| Assessor's overall Judgement | High |

| **Study ID** | **Ranasinghe 2023** |
| --- | --- |
| *Experimental* | **Peer-led support groups** |
| *Comparator* |  |
| Outcome | Depressive symptoms |
| **Domain 1:**  **Risk of bias arising from the randomization process** |  |
| 1.1 | PY |
| 1.2 | NI |
| Note for 1.1&1.2 | Quote: "Allocation to intervention and control groups was by simple random sampling"  No further information about allocation concealment. |
| 1.3 | PN |
| Note for 1.3 | The observed differences are compatible with chance. Quote: "There were no statistical significant differences of demographic and clinical parameters. The only differences were on 'menstrual problems' and 'emotions'" |
| 1.0 Assessor's Judgement | Low |
| **Domain 2:**  **Risk of bias due to deviations from the intended interventions** |  |
| 2.1 | PY |
| 2.2 | Y |
| Note for 2.1&2.2 | Due to the nature of the intervention participants were most likely aware of their assigned intervention.  Quote: "The faciliattor was familiarised about the nature and objective of the support group..." |
| 2.3 | PN |
| Note for 2.3 | There is no reason to believe it |
| 2.4 | NA |
| 2.5 | NA |
| 2.6 | PN |
| Note for 2.6 | Analyses exclude eligible trial participants post randomization. |
| 2.7 | NI |
| 2.0 Assessor's Judgement | High |
| **Domain 3:**  **Missing outcome data** |  |
| 3.1 | PN |
| Note for 3.1 | The drop-out rate is 19% with no reasons explained. |
| 3.2 | N |
| Note for 3.2 | No information to suggest that results were not biased by missing outcome data |
| 3.3 | NI |
| Note for 3.3&3.4 | Reasons for missing data are not explained. |
| 3.4 | NI |
| 3.0 Assessor's judgement | High |
| **Domain 4:**  **Risk of bias in measurement of the outcome** |  |
| 4.1 | N |
| Note for 4.1 | Quote: "The CES-D has been validated and translated in Sinhala" |
| 4.2 | PN |
| Note for 4.2 | No evidence to suggest differences in measurement between intervention and control. |
| 4.3 | Y |
| Note for 4.3 | Only self-reported measures |
| 4.4 | PY |
| Note for 4.4&4.5 | Knowledge of the assigned intervention could influence participant-reported outcomes, but there is no reason to believe that it did |
| 4.5 | PN |
| 4.0 Assessor's Judgement | Some concerns |
| **Domain 5:**  **Risk of bias in selection of the reported result** |  |
| 5.1 | N |
| Note for 5.1 | No protocol available |
| 5.2 | N |
| Note for 5.2 | No evidence to suggest outcome selection. All outcomes mentioned in the methods and results were reported |
| 5.3 | N |
| Note for 5.3 | No evidence to suggest analyses selection. All outcomes mentioned in the methods and results were reported |
| 5.0 Assessor's Judgement | Some concerns |
| **Overall Risk of Bias** |  |
| Assessor's overall Judgement | High |

| **Study ID** | **Osborn 2020** |
| --- | --- |
| *Experimental* | **Shamiri program** |
| *Comparator* |  |
| Outcome | Depression diagnosis |
| **Domain 1: Risk of bias arising from the randomization process** |  |
| 1.1 | Y |
| 1.2 | PY |
| Note for 1.1&1.2 | Quote: "At the start of each session, participants were  randomly assigned to the intervention condition or study-skills condition using a random-number generator embedded in the study website. The study team was thus blind to this allocation" |
| 1.3 | PN |
| Note for 1.3 | Data between cases and controls is comparable but no formal statistical testing in reported |
| 1.0 Assessor's Judgement | Low |
| **Domain 2: Risk of bias due to deviations from the intended interventions** |  |
| 2.1 | PY |
| 2.2 | N |
| Note for 2.1&2.2 | Given the self-help internet-based nature of the intervention, participants were most likely aware of allocation. Carers were not involved in the intervention. The study team was blind to allocation. The delivery was done digitally. |
| 2.3 | PN |
| Note for 2.3 | No evidence of deviations from protocol |
| 2.4 | NA |
| 2.5 | NA |
| 2.6 | PY |
| Note for 2.6 | No loss to follow-up were reported, all randomized participants were analyzed. |
| 2.7 | NA |
| 2.0 Assessor's Judgement | Low |
| **Domain 3: Missing outcome data** |  |
| 3.1 | Y |
| Note for 3.1 | All randomized participants took part to the intervention and were assessed at baseline and follow-up. |
| 3.2 | NA |
| 3.3 | NA |
| 3.4 | NA |
| 3.0 Assessor's judgement | Low |
| **Domain 4: Risk of bias in measurement of the outcome** |  |
| 4.1 | N |
| Note for 4.1 | Quote: “The PHQ-8 has also demonstrated adequate internal consistency ( .73) and discriminant validity with Kenyan adolescents . Cronbach’s alpha for the PHQ-8 in the present study was 0.73.” |
| 4.2 | PN |
| Note for 4.2 | No evidence to suggest differences in measurement between intervention and wait list. |
| 4.3 | PN |
| Note for 4.3 | Outcome assessors (that given the RoB guidelines are the adolescents) were most likely aware of intervention allocation, research team members were blind at baseline, no information for follow-up blinding |
| 4.4 | PN |
| Note for 4.4&4.5 | Knowledge of the assigned intervention could influence participant-reported outcomes, but there is no reason to believe that it did |
| 4.5 | PN |
| 4.0 Assessor's Judgement | Some concerns |
| **Domain 5: Risk of bias in selection of the reported result** |  |
| 5.1 | PY |
| Note for 5.1 | The protocol reports analyses that are in line with those found in the methods and results sections of the journal article |
| 5.2 | PY |
| Note for 5.2 | One of the secondary outcomes found in the protocol (changes in perceived academic control; the Perceived Control Scale for Children - Academic sub scale) was not mentioned in any section of the article |
| 5.3 | PN |
| Note for 5.3 | No evidence to suggest selection of analyses. |
| 5.0 Assessor's Judgement | High |
| **Overall Risk of Bias** |  |
| Assessor's overall Judgement | High |

| **Study ID** | **Osborn 2020** |
| --- | --- |
| *Experimental* | **Shamiri program** |
| *Comparator* |  |
| Outcome | Depressive symptoms |
| **Domain 1: Risk of bias arising from the randomization process** |  |
| 1.1 | Y |
| 1.2 | PY |
| Note for 1.1&1.2 | Quote: "At the start of each session, participants were  randomly assigned to the intervention condition or study-skills condition using a random-number generator embedded in the study website. The study team was thus blind to this allocation" |
| 1.3 | PN |
| Note for 1.3 | Data between cases and controls is comparable but no formal statistical testing in reported |
| 1.0 Assessor's Judgement | Low |
| **Domain 2: Risk of bias due to deviations from the intended interventions** |  |
| 2.1 | PY |
| 2.2 | N |
| Note for 2.1&2.2 | Given the self-help internet-based nature of the intervention, participants were most likely aware of allocation. Carers were not involved in the intervention. The study team was blind to allocation. The delivery was done digitally. |
| 2.3 | PN |
| Note for 2.3 | No evidence of deviations from protocol |
| 2.4 | NA |
| 2.5 | NA |
| 2.6 | PY |
| Note for 2.6 | No loss to follow-up were reported, all randomized participants were analyzed. |
| 2.7 | NA |
| 2.0 Assessor's Judgement | Low |
| **Domain 3: Missing outcome data** |  |
| 3.1 | Y |
| Note for 3.1 | All randomized participants took part to the intervention and were assessed at baseline and follow-up. |
| 3.2 | NA |
| 3.3 | NA |
| 3.4 | NA |
| 3.0 Assessor's judgement | Low |
| **Domain 4: Risk of bias in measurement of the outcome** |  |
| 4.1 | N |
| Note for 4.1 | Quote: “The PHQ-8 has also demonstrated adequate internal consistency ( .73) and discriminant validity with Kenyan adolescents . Cronbach’s alpha for the PHQ-8 in the present study was 0.73.” |
| 4.2 | PN |
| Note for 4.2 | No evidence to suggest differences in measurement between intervention and wait list. |
| 4.3 | PN |
| Note for 4.3 | Outcome assessors (that given the RoB guidelines are the adolescents) were most likely aware of intervention allocation, research team members were blind at baseline, no information for follow-up blinding |
| 4.4 | PN |
| Note for 4.4&4.5 | Knowledge of the assigned intervention could influence participant-reported outcomes, but there is no reason to believe that it did |
| 4.5 | PN |
| 4.0 Assessor's Judgement | Some concerns |
| **Domain 5: Risk of bias in selection of the reported result** |  |
| 5.1 | PY |
| Note for 5.1 | The protocol reports analyses that are in line with those found in the methods and results sections of the journal article |
| 5.2 | PY |
| Note for 5.2 | One of the secondary outcomes found in the protocol (changes in perceived academic control; the Perceived Control Scale for Children - Academic sub scale) was not mentioned in any section of the article |
| 5.3 | PN |
| Note for 5.3 | No evidence to suggest selection of analyses. |
| 5.0 Assessor's Judgement | High |
| **Overall Risk of Bias** |  |
| Assessor's overall Judgement | High |

| **Study ID** | **Osborn 2020** |
| --- | --- |
| *Experimental* | **Shamiri program** |
| *Comparator* |  |
| Outcome | Anxiety symptoms |
| **Domain 1: Risk of bias arising from the randomization process** |  |
| 1.1 | Y |
| 1.2 | PY |
| Note for 1.1&1.2 | Quote: "At the start of each session, participants were  randomly assigned to the intervention condition or study-skills condition using a random-number generator embedded in the study website. The study team was thus blind to this allocation" |
| 1.3 | PN |
| Note for 1.3 | Data between cases and controls is comparable but no formal statistical testing in reported |
| 1.0 Assessor's Judgement | Low |
| **Domain 2: Risk of bias due to deviations from the intended interventions** |  |
| 2.1 | PY |
| 2.2 | N |
| Note for 2.1&2.2 | Given the self-help internet-based nature of the intervention, participants were most likely aware of allocation. Carers were not involved in the intervention. The study team was blind to allocation. The delivery was done digitally. |
| 2.3 | PN |
| Note for 2.3 | No evidence of deviations from protocol |
| 2.4 | NA |
| 2.5 | NA |
| 2.6 | PY |
| Note for 2.6 | No loss to follow-up were reported, all randomized participants were analyzed. |
| 2.7 | NA |
| 2.0 Assessor's Judgement | Low |
| **Domain 3: Missing outcome data** |  |
| 3.1 | Y |
| Note for 3.1 | All randomized participants took part to the intervention and were assessed at baseline and follow-up. |
| 3.2 | NA |
| 3.3 | NA |
| 3.4 | NA |
| 3.0 Assessor's judgement | Low |
| **Domain 4: Risk of bias in measurement of the outcome** |  |
| 4.1 | N |
| Note for 4.1 | Quote: “The PHQ-8 has also demonstrated adequate internal consistency ( .73) and discriminant validity with Kenyan adolescents . Cronbach’s alpha for the PHQ-8 in the present study was 0.73.” |
| 4.2 | PN |
| Note for 4.2 | No evidence to suggest differences in measurement between intervention and wait list. |
| 4.3 | PN |
| Note for 4.3 | Outcome assessors (that given the RoB guidelines are the adolescents) were most likely aware of intervention allocation, research team members were blind at baseline, no information for follow-up blinding |
| 4.4 | PN |
| Note for 4.4&4.5 | Knowledge of the assigned intervention could influence participant-reported outcomes, but there is no reason to believe that it did |
| 4.5 | PN |
| 4.0 Assessor's Judgement | Some concerns |
| **Domain 5: Risk of bias in selection of the reported result** |  |
| 5.1 | PY |
| Note for 5.1 | The protocol reports analyses that are in line with those found in the methods and results sections of the journal article |
| 5.2 | PY |
| Note for 5.2 | One of the secondary outcomes found in the protocol (changes in perceived academic control; the Perceived Control Scale for Children - Academic sub scale) was not mentioned in any section of the article |
| 5.3 | PN |
| Note for 5.3 | No evidence to suggest selection of analyses. |
| 5.0 Assessor's Judgement | High |
| **Overall Risk of Bias** |  |
| Assessor's overall Judgement | High |

| **Study ID** | **O'Callaghan 2014** |
| --- | --- |
| *Experimental* | Family focused psychosocial intervention |
| *Comparator* |  |
| Outcome | PTSD symptoms |
| **Domain 1: Risk of bias arising from the randomization process** |  |
| 1.1 | Y |
| 1.2 | PY |
| Note for 1.1&1.2 | Quote: "each member of the pair was randomly assigned to either the treatment or the control group using a computer generated random sequence (www.random.org). This sequence was supplied by one of the authors off site. The lead author then allocated participants using the randomized sequence"; Quote: "Selection bias was reduced by ensuring treatment allocation was concealed from those responsible for participant enrolment and by ensuring the person responsible for assigning participants met none of the participants prior to the group allocation". |
| 1.3 | N |
| Note for 1.3 | Quote: "There were no important differences found between groups on the outcome measures at pre-intervention"; "A total of 159 children participated in the study: 79 in the intervention group and 80 in the control group. The mean age of the children in each group was similar, as was the ratio of males: females, the ratio of children recruited from each village (see Table 1), and the proportion of children that had been abducted or experienced other events at the hands of the LRA" |
| 1.0 Assessor's Judgement | Low |
| **Domain 2: Risk of bias due to deviations from the intended interventions** |  |
| 2.1 | PY |
| 2.2 | PY |
| Note for 2.1&2.2 | Due to the natire of the intervention, participants and carers were most likely aware of their assigned intervention |
| 2.3 | PN |
| Note for 2.3 | No evidence of deviations from protocol |
| 2.4 | NA |
| 2.5 | NA |
| 2.6 | PY |
| Note for 2.6 | Limited missing (N=6); all randomized participants were included in the primary analysis according to the CONSORT chart. |
| 2.7 | NA |
| 2.0 Assessor's Judgement | Low |
| **Domain 3: Missing outcome data** |  |
| 3.1 | PY |
| Note for 3.1 | Quote: "There were six participants who failed to provide data at the post-test stage (3 participants from each group)."; "Except in the case of participants who could not be found for post or follow-up assessment, there were no other missing values for any question in this study.". A total of 159 participants were randomized in thi trial (missings: 3.77%) |
| 3.2 | NA |
| 3.3 | NA |
| 3.4 | NA |
| 3.0 Assessor's judgement | Low |
| **Domain 4: Risk of bias in measurement of the outcome** |  |
| 4.1 | N |
| Note for 4.1 | 8-item CRIES presents good psychometric properties within this population. It was "previously validated with a sample of 1,046 war-affected adolescents in eastern DR Congo (internal reliability range: 0.79–0.84; Cronbach’s alpha for the total scale: 0.93). In the current study, internal consistency was 0.557." |
| 4.2 | PN |
| Note for 4.2 | No evidence to suggest differences in measurement between intervention groups. |
| 4.3 | PY |
| Note for 4.3 | Outcome assessors (children) were probably aware of intervention allocation; those responsible for data collection were blind to the group allocation |
| 4.4 | PY |
| Note for 4.4&4.5 | Knowledge of the assigned intervention could influence participant-reported outcomes, but there is no reason to believe that it did |
| 4.5 | PN |
| 4.0 Assessor's Judgement | Some concerns |
| **Domain 5: Risk of bias in selection of the reported result** |  |
| 5.1 | PY |
| Note for 5.1 | No evidence to suggest otherwise, the protocol and manuscript do not show discrepancies |
| 5.2 | N |
| Note for 5.2 | All pre-determined outcomes found in protocol were reported. In accordance to protocol, data at 3 months follow-up was not collected for controls given the wait list nature of the group and ethical concerns. |
| 5.3 | N |
| Note for 5.3 | No evidence to suggest selection of analyses. |
| 5.0 Assessor's Judgement | Low |
| **Overall Risk of Bias** |  |
| Assessor's overall Judgement | Some concerns |

| **Study ID** | **O'Callaghan 2014** |
| --- | --- |
| *Experimental* | Family focused psychosocial intervention |
| *Comparator* |  |
| Outcome | PTSD diagnosis |
| **Domain 1: Risk of bias arising from the randomization process** |  |
| 1.1 | Y |
| 1.2 | PY |
| Note for 1.1&1.2 | Quote: "each member of the pair was randomly assigned to either the treatment or the control group using a computer generated random sequence (www.random.org). This sequence was supplied by one of the authors off site. The lead author then allocated participants using the randomized sequence"; Quote: "Selection bias was reduced by ensuring treatment allocation was concealed from those responsible for participant enrolment and by ensuring the person responsible for assigning participants met none of the participants prior to the group allocation". |
| 1.3 | N |
| Note for 1.3 | Quote: "There were no important differences found between groups on the outcome measures at pre-intervention"; "A total of 159 children participated in the study: 79 in the intervention group and 80 in the control group. The mean age of the children in each group was similar, as was the ratio of males: females, the ratio of children recruited from each village (see Table 1), and the proportion of children that had been abducted or experienced other events at the hands of the LRA" |
| 1.0 Assessor's Judgement | Low |
| **Domain 2: Risk of bias due to deviations from the intended interventions** |  |
| 2.1 | PY |
| 2.2 | PY |
| Note for 2.1&2.2 | Due to the natire of the intervention, participants and carers were most likely aware of their assigned intervention |
| 2.3 | PN |
| Note for 2.3 | No evidence of deviations from protocol |
| 2.4 | NA |
| 2.5 | NA |
| 2.6 | PY |
| Note for 2.6 | Limited missing (N=6); all randomized participants were included in the primary analysis according to the CONSORT chart. |
| 2.7 | NA |
| 2.0 Assessor's Judgement | Low |
| **Domain 3: Missing outcome data** |  |
| 3.1 | PY |
| Note for 3.1 | Quote: "There were six participants who failed to provide data at the post-test stage (3 participants from each group)."; "Except in the case of participants who could not be found for post or follow-up assessment, there were no other missing values for any question in this study.". A total of 159 participants were randomized in thi trial (missings: 3.77%) |
| 3.2 | NA |
| 3.3 | NA |
| 3.4 | NA |
| 3.0 Assessor's judgement | Low |
| **Domain 4: Risk of bias in measurement of the outcome** |  |
| 4.1 | N |
| Note for 4.1 | 8-item CRIES presents good psychometric properties within this population. It was "previously validated with a sample of 1,046 war-affected adolescents in eastern DR Congo (internal reliability range: 0.79–0.84; Cronbach’s alpha for the total scale: 0.93). In the current study, internal consistency was 0.557." |
| 4.2 | PN |
| Note for 4.2 | No evidence to suggest differences in measurement between intervention groups. |
| 4.3 | PY |
| Note for 4.3 | Outcome assessors (children) were probably aware of intervention allocation; those responsible for data collection were blind to the group allocation |
| 4.4 | PY |
| Note for 4.4&4.5 | Knowledge of the assigned intervention could influence participant-reported outcomes, but there is no reason to believe that it did |
| 4.5 | PN |
| 4.0 Assessor's Judgement | Some concerns |
| **Domain 5: Risk of bias in selection of the reported result** |  |
| 5.1 | PY |
| Note for 5.1 | No evidence to suggest otherwise, the protocol and manuscript do not show discrepancies |
| 5.2 | N |
| Note for 5.2 | All pre-determined outcomes found in protocol were reported. In accordance to protocol, data at 3 months follow-up was not collected for controls given the wait list nature of the group and ethical concerns. |
| 5.3 | N |
| Note for 5.3 | No evidence to suggest selection of analyses. |
| 5.0 Assessor's Judgement | Low |
| **Overall Risk of Bias** |  |
| Assessor's overall Judgement | Some concerns |

| **Study ID** | **O'Callaghan 2014** |
| --- | --- |
| *Experimental* | Family focused psychosocial intervention |
| *Comparator* | wait list |
| Outcome | Depressive diagnosis |
| **Domain 1: Risk of bias arising from the randomization process** |  |
| 1.1 | Y |
| 1.2 | PY |
| Note for 1.1&1.2 | Quote: "each member of the pair was randomly assigned to either the treatment or the control group using a computer generated random sequence (www.random.org). This sequence was supplied by one of the authors off site. The lead author then allocated participants using the randomized sequence"; Quote: "Selection bias was reduced by ensuring treatment allocation was concealed from those responsible for participant enrolment and by ensuring the person responsible for assigning participants met none of the participants prior to the group allocation". |
| 1.3 | N |
| Note for 1.3 | Quote: "There were no important differences found between groups on the outcome measures at pre-intervention"; "A total of 159 children participated in the study: 79 in the intervention group and 80 in the control group. The mean age of the children in each group was similar, as was the ratio of males: females, the ratio of children recruited from each village (see Table 1), and the proportion of children that had been abducted or experienced other events at the hands of the LRA" |
| 1.0 Assessor's Judgement | Low |
| **Domain 2: Risk of bias due to deviations from the intended interventions** |  |
| 2.1 | PY |
| 2.2 | PY |
| Note for 2.1&2.2 | Due to the natire of the intervention, participants and carers were most likely aware of their assigned intervention |
| 2.3 | PN |
| Note for 2.3 | No evidence of deviations from protocol |
| 2.4 | NA |
| 2.5 | NA |
| 2.6 | PY |
| Note for 2.6 | Limited missing (N=6); all randomized participants were included in the primary analysis according to the CONSORT chart. |
| 2.7 | NA |
| 2.0 Assessor's Judgement | Low |
| **Domain 3: Missing outcome data** |  |
| 3.1 | PY |
| Note for 3.1 | Quote: "There were six participants who failed to provide data at the post-test stage (3 participants from each group)."; "Except in the case of participants who could not be found for post or follow-up assessment, there were no other missing values for any question in this study.". A total of 159 participants were randomized in thi trial (missings: 3.77%) |
| 3.2 | NA |
| 3.3 | NA |
| 3.4 | NA |
| 3.0 Assessor's judgement | Low |
| **Domain 4: Risk of bias in measurement of the outcome** |  |
| 4.1 | N |
| Note for 4.1 | Quote: "The AYPA was chosen because it is the only African developed, validated questionnaire available, had been used in separate studies with war-affected children in the DR Congo"; Good psychometric properties within the sample "Test-retest reliability (carried out with a subset of 30 participants) for the AYPA was 0.91, inter-rater reliability was 0.58 (n = 26) and internal consistency ranged from 0.637 (conduct) to 0.787 (internalising symptoms)." |
| 4.2 | PN |
| Note for 4.2 | No evidence to suggest differences in measurement between intervention groups. |
| 4.3 | PY |
| Note for 4.3 | Outcome assessors (children) were probably aware of intervention allocation; those responsible for data collection were blind to the group allocation |
| 4.4 | PY |
| Note for 4.4&4.5 | Knowledge of the assigned intervention could influence participant-reported outcomes, but there is no reason to believe that it did |
| 4.5 | PN |
| 4.0 Assessor's Judgement | Some concerns |
| **Domain 5: Risk of bias in selection of the reported result** |  |
| 5.1 | PY |
| Note for 5.1 | No evidence to suggest otherwise, the protocol and manuscript do not show discrepancies |
| 5.2 | N |
| Note for 5.2 | All pre-determined outcomes found in protocol were reported. In accordance to protocol, data at 3 months follow-up was not collected for controls given the wait list nature of the group and ethical concerns. |
| 5.3 | N |
| Note for 5.3 | No evidence to suggest selection of analyses. |
| 5.0 Assessor's Judgement | Low |
| **Overall Risk of Bias** |  |
| Assessor's overall Judgement | Some concerns |

| **Study ID** | **O'Callaghan 2014** |
| --- | --- |
| *Experimental* | Family focused psychosocial intervention |
| *Comparator* | wait list |
| Outcome | Depressive symptoms |
| **Domain 1: Risk of bias arising from the randomization process** |  |
| 1.1 | Y |
| 1.2 | PY |
| Note for 1.1&1.2 | Quote: "each member of the pair was randomly assigned to either the treatment or the control group using a computer generated random sequence (www.random.org). This sequence was supplied by one of the authors off site. The lead author then allocated participants using the randomized sequence"; Quote: "Selection bias was reduced by ensuring treatment allocation was concealed from those responsible for participant enrolment and by ensuring the person responsible for assigning participants met none of the participants prior to the group allocation". |
| 1.3 | N |
| Note for 1.3 | Quote: "There were no important differences found between groups on the outcome measures at pre-intervention"; "A total of 159 children participated in the study: 79 in the intervention group and 80 in the control group. The mean age of the children in each group was similar, as was the ratio of males: females, the ratio of children recruited from each village (see Table 1), and the proportion of children that had been abducted or experienced other events at the hands of the LRA" |
| 1.0 Assessor's Judgement | Low |
| **Domain 2: Risk of bias due to deviations from the intended interventions** |  |
| 2.1 | PY |
| 2.2 | PY |
| Note for 2.1&2.2 | Due to the natire of the intervention, participants and carers were most likely aware of their assigned intervention |
| 2.3 | PN |
| Note for 2.3 | No evidence of deviations from protocol |
| 2.4 | NA |
| 2.5 | NA |
| 2.6 | PY |
| Note for 2.6 | Limited missing (N=6); all randomized participants were included in the primary analysis according to the CONSORT chart. |
| 2.7 | NA |
| 2.0 Assessor's Judgement | Low |
| **Domain 3: Missing outcome data** |  |
| 3.1 | PY |
| Note for 3.1 | Quote: "There were six participants who failed to provide data at the post-test stage (3 participants from each group)."; "Except in the case of participants who could not be found for post or follow-up assessment, there were no other missing values for any question in this study.". A total of 159 participants were randomized in thi trial (missings: 3.77%) |
| 3.2 | NA |
| 3.3 | NA |
| 3.4 | NA |
| 3.0 Assessor's judgement | Low |
| **Domain 4: Risk of bias in measurement of the outcome** |  |
| 4.1 | N |
| Note for 4.1 | Quote: "The AYPA was chosen because it is the only African developed, validated questionnaire available, had been used in separate studies with war-affected children in the DR Congo"; Good psychometric properties within the sample "Test-retest reliability (carried out with a subset of 30 participants) for the AYPA was 0.91, inter-rater reliability was 0.58 (n = 26) and internal consistency ranged from 0.637 (conduct) to 0.787 (internalising symptoms)." |
| 4.2 | PN |
| Note for 4.2 | No evidence to suggest differences in measurement between intervention groups. |
| 4.3 | PY |
| Note for 4.3 | Outcome assessors (children) were probably aware of intervention allocation; those responsible for data collection were blind to the group allocation |
| 4.4 | PY |
| Note for 4.4&4.5 | Knowledge of the assigned intervention could influence participant-reported outcomes, but there is no reason to believe that it did |
| 4.5 | PN |
| 4.0 Assessor's Judgement | Some concerns |
| **Domain 5: Risk of bias in selection of the reported result** |  |
| 5.1 | PY |
| Note for 5.1 | No evidence to suggest otherwise, the protocol and manuscript do not show discrepancies |
| 5.2 | N |
| Note for 5.2 | All pre-determined outcomes found in protocol were reported. In accordance to protocol, data at 3 months follow-up was not collected for controls given the wait list nature of the group and ethical concerns. |
| 5.3 | N |
| Note for 5.3 | No evidence to suggest selection of analyses. |
| 5.0 Assessor's Judgement | Low |
| **Overall Risk of Bias** |  |
| Assessor's overall Judgement | Some concerns |

| **Study ID** | **Lachman 2017** |
| --- | --- |
| *Experimental* | Sinovuyo Caring Families Program for Young Children |
| *Comparator* | wait list |
| Outcome | PTSD symptoms |
| **Domain 1: Risk of bias arising from the randomization process** |  |
| 1.1 | Y |
| 1.2 | Y |
| Note for 1.1&1.2 | Quote: "An external researcher not directly involved in the study conducted the randomization procedures remotely in Oxford, United Kingdom. Participants were randomly assigned on a 1:1 ratio to an intervention or wait-list control group after baseline data collection using a concealed computerized program, SealedEnvelope™."; "Our implementing partner, Clowns Without Borders South Africa, notified participants of their allocation status via telephone." |
| 1.3 | PN |
| Note for 1.3 | Quote: "Independent t-tests and chi-squared tests found no differences between the intervention and control groups for all demographics and outcome measures at baseline." |
| 1.0 Assessor's Judgement | Low |
| **Domain 2: Risk of bias due to deviations from the intended interventions** |  |
| 2.1 | Y |
| 2.2 | Y |
| Note for 2.1&2.2 | Quote: "Although program implementers and participants were aware of their allocation status, researchers conducting self-report interviews and observational assessments were blind to allocation." |
| 2.3 | PN |
| Note for 2.3 | No evidence to suggest issues to implement intervention |
| 2.4 | NA |
| 2.5 | NA |
| 2.6 | Y |
| Note for 2.6 | Quote: "Data analyses were conducted with an intention-to-treat design" |
| 2.7 | NA |
| 2.0 Assessor's Judgement | Low |
| **Domain 3: Missing outcome data** |  |
| 3.1 | Y |
| Note for 3.1 | 2 out of the 68 participants randomized were lost at follow-up (2.9%) |
| 3.2 | NA |
| 3.3 | NA |
| 3.4 | NA |
| 3.0 Assessor's judgement | Low |
| **Domain 4: Risk of bias in measurement of the outcome** |  |
| 4.1 | PN |
| Note for 4.1 | The selected outcome method is widely validated and established, appropriate for the local context, and assessed in its psychometric properties for this sample. |
| 4.2 | PN |
| Note for 4.2 | No evidence to suggest differences in measurement between intervention and control. |
| 4.3 | Y |
| Note for 4.3 | Outcome assessors (participants) were most likely aware of intervention allocation. The research assistants administering the questionnaires were blinded. |
| 4.4 | PY |
| Note for 4.4&4.5 | Knowledge of the assigned intervention could influence participant-reported outcomes, but there is no reason to believe that it did |
| 4.5 | PN |
| 4.0 Assessor's Judgement | Some concerns |
| **Domain 5: Risk of bias in selection of the reported result** |  |
| 5.1 | PY |
| Note for 5.1 | No evidence to suggest otherwise, the protocol and manuscript do not show discrepancies. |
| 5.2 | PY |
| Note for 5.2 | A few of the secondary outcomes mentioned in the protocol (e.g. ASSIST, Grover-Counter Scale of Cognitive Development) were not reported in this manuscript. |
| 5.3 | PN |
| Note for 5.3 | No evidence to suggest analyses selection. All analyses mentioned in the protocol and methods were reported in the results. |
| 5.0 Assessor's Judgement | Some concerns |
| 5.0 General note | While the algorithm suggests this section to be classified with a high risk of bias due to some evidence indicating outcome selection, the outcomes that were not reported in this specific manuscript were secondary and could have fitted better in a related secondary analysis article. This is why the judgment for domain 5 indicates "some concerns". |
| **Overall Risk of Bias** |  |
| Assessor's overall Judgement | Some concerns |

| **Study ID** | **Lachman 2017** |
| --- | --- |
| *Experimental* | Sinovuyo Caring Families Program for Young Children |
| *Comparator* | wait list |
| Outcome | Depression diagnosis |
| **Domain 1: Risk of bias arising from the randomization process** |  |
| 1.1 | Y |
| 1.2 | Y |
| Note for 1.1&1.2 | Quote: "An external researcher not directly involved in the study conducted the randomization procedures remotely in Oxford, United Kingdom. Participants were randomly assigned on a 1:1 ratio to an intervention or wait-list control group after baseline data collection using a concealed computerized program, SealedEnvelope™."; "Our implementing partner, Clowns Without Borders South Africa, notified participants of their allocation status via telephone." |
| 1.3 | PN |
| Note for 1.3 | Quote: "Independent t-tests and chi-squared tests found no differences between the intervention and control groups for all demographics and outcome measures at baseline." |
| 1.0 Assessor's Judgement | Low |
| **Domain 2: Risk of bias due to deviations from the intended interventions** |  |
| 2.1 | Y |
| 2.2 | Y |
| Note for 2.1&2.2 | Quote: "Although program implementers and participants were aware of their allocation status, researchers conducting self-report interviews and observational assessments were blind to allocation." |
| 2.3 | PN |
| Note for 2.3 | No evidence to suggest issues to implement intervention |
| 2.4 | NA |
| 2.5 | NA |
| 2.6 | Y |
| Note for 2.6 | Quote: "Data analyses were conducted with an intention-to-treat design" |
| 2.7 | NA |
| 2.0 Assessor's Judgement | Low |
| **Domain 3: Missing outcome data** |  |
| 3.1 | Y |
| Note for 3.1 | 3 out of the 68 participants randomized were lost at follow-up (2.9%) |
| 3.2 | NA |
| 3.3 | NA |
| 3.4 | NA |
| 3.0 Assessor's judgement | Low |
| **Domain 4: Risk of bias in measurement of the outcome** |  |
| 4.1 | PN |
| Note for 4.1 | The selected outcome method is widely validated and established, appropriate for the local context, and assessed in its psychometric properties for this sample. |
| 4.2 | PN |
| Note for 4.2 | No evidence to suggest differences in measurement between intervention and control. |
| 4.3 | Y |
| Note for 4.3 | Outcome assessors (participants) were most likely aware of intervention allocation. The research assistants administering the questionnaires were blinded. |
| 4.4 | PY |
| Note for 4.4&4.5 | Knowledge of the assigned intervention could influence participant-reported outcomes, but there is no reason to believe that it did |
| 4.5 | PN |
| 4.0 Assessor's Judgement | Some concerns |
| **Domain 5: Risk of bias in selection of the reported result** |  |
| 5.1 | PY |
| Note for 5.1 | No evidence to suggest otherwise, the protocol and manuscript do not show discrepancies. |
| 5.2 | PY |
| Note for 5.2 | A few of the secondary outcomes mentioned in the protocol (e.g. ASSIST, Grover-Counter Scale of Cognitive Development) were not reported in this manuscript. |
| 5.3 | PN |
| Note for 5.3 | No evidence to suggest analyses selection. All analyses mentioned in the protocol and methods were reported in the results. |
| 5.0 Assessor's Judgement | Some concerns |
| 5.0 General note | While the algorithm suggests this section to be classified with a high risk of bias due to some evidence indicating outcome selection, the outcomes that were not reported in this specific manuscript were secondary and could have fitted better in a related secondary analysis article. This is why the judgment for domain 5 indicates "some concerns". |
| **Overall Risk of Bias** |  |
| Assessor's overall Judgement | Some concerns |

| **Study ID** | **Lachman 2017** |
| --- | --- |
| *Experimental* | Sinovuyo Caring Families Program for Young Children |
| *Comparator* | wait list |
| Outcome | Depressive symptoms |
| **Domain 1: Risk of bias arising from the randomization process** |  |
| 1.1 | Y |
| 1.2 | Y |
| Note for 1.1&1.2 | Quote: "An external researcher not directly involved in the study conducted the randomization procedures remotely in Oxford, United Kingdom. Participants were randomly assigned on a 1:1 ratio to an intervention or wait-list control group after baseline data collection using a concealed computerized program, SealedEnvelope™."; "Our implementing partner, Clowns Without Borders South Africa, notified participants of their allocation status via telephone." |
| 1.3 | PN |
| Note for 1.3 | Quote: "Independent t-tests and chi-squared tests found no differences between the intervention and control groups for all demographics and outcome measures at baseline." |
| 1.0 Assessor's Judgement | Low |
| **Domain 2: Risk of bias due to deviations from the intended interventions** |  |
| 2.1 | Y |
| 2.2 | Y |
| Note for 2.1&2.2 | Quote: "Although program implementers and participants were aware of their allocation status, researchers conducting self-report interviews and observational assessments were blind to allocation." |
| 2.3 | PN |
| Note for 2.3 | No evidence to suggest issues to implement intervention |
| 2.4 | NA |
| 2.5 | NA |
| 2.6 | Y |
| Note for 2.6 | Quote: "Data analyses were conducted with an intention-to-treat design" |
| 2.7 | NA |
| 2.0 Assessor's Judgement | Low |
| **Domain 3: Missing outcome data** |  |
| 3.1 | Y |
| Note for 3.1 | 3 out of the 68 participants randomized were lost at follow-up (2.9%) |
| 3.2 | NA |
| 3.3 | NA |
| 3.4 | NA |
| 3.0 Assessor's judgement | Low |
| **Domain 4: Risk of bias in measurement of the outcome** |  |
| 4.1 | PN |
| Note for 4.1 | The selected outcome method is widely validated and established, appropriate for the local context, and assessed in its psychometric properties for this sample. |
| 4.2 | PN |
| Note for 4.2 | No evidence to suggest differences in measurement between intervention and control. |
| 4.3 | Y |
| Note for 4.3 | Outcome assessors (participants) were most likely aware of intervention allocation. The research assistants administering the questionnaires were blinded. |
| 4.4 | PY |
| Note for 4.4&4.5 | Knowledge of the assigned intervention could influence participant-reported outcomes, but there is no reason to believe that it did |
| 4.5 | PN |
| 4.0 Assessor's Judgement | Some concerns |
| **Domain 5: Risk of bias in selection of the reported result** |  |
| 5.1 | PY |
| Note for 5.1 | No evidence to suggest otherwise, the protocol and manuscript do not show discrepancies. |
| 5.2 | PY |
| Note for 5.2 | A few of the secondary outcomes mentioned in the protocol (e.g. ASSIST, Grover-Counter Scale of Cognitive Development) were not reported in this manuscript. |
| 5.3 | PN |
| Note for 5.3 | No evidence to suggest analyses selection. All analyses mentioned in the protocol and methods were reported in the results. |
| 5.0 Assessor's Judgement | Some concerns |
| 5.0 General note | While the algorithm suggests this section to be classified with a high risk of bias due to some evidence indicating outcome selection, the outcomes that were not reported in this specific manuscript were secondary and could have fitted better in a related secondary analysis article. This is why the judgment for domain 5 indicates "some concerns". |
| **Overall Risk of Bias** |  |
| Assessor's overall Judgement | Some concerns |

| **Study ID** | **Skar 2021** |
| --- | --- |
| *Experimental* | International Child Development Programme (ICDP) |
| *Comparator* | Violence Curriculum |
| Outcome | Depression diagnosis |
| **Domain 1: Risk of bias arising from the randomization process** |  |
| 1.1 | PY |
| 1.2 | PN |
| Note for 1.1&1.2 | 1.1: Title mentions RCT design. Quote: "A three-group randomized controlled design was utilized. ". 1.2.Quote: "The local project coordinator conducted the randomization by putting down all the names of the recruited families in a random order and giving them a number for each of the group conditions (1, 2, 3, 1, 2, 3, etc.).". Given that the coordinator had access to the names of the families during the randomization there is a chance that allocation was not random. |
| 1.3 | NI |
| Note for 1.3 | 1a.3 No useful information is reported to evaluate this element |
| 1.0 Assessor's Judgement | Some concerns |
| 1.0 General note | 1b.1: Y - Individual participants were identified before randomization of clusters. Overall: High |
| **Domain 2: Risk of bias due to deviations from the intended interventions** |  |
| 2.1 | NI; PY |
| 2.2 | PY |
| Note for 2.1&2.2 | 2.1 a / 2.1b / 2.2 No information provided on whether participants knew that they were in a trial, due to the nature of the intervention they (as those delivering the intervention) were aware of intervention allocation. |
| 2.3 | PN |
| Note for 2.3 | No evidence to indicate deviations from protocol |
| 2.4 | NA |
| 2.5 | NA |
| 2.6 | NI |
| Note for 2.6 | Not enough information provided to give a judjment on this item |
| 2.7 | NI |
| Note for 2.7 | Not enough information provided to give a judjment on this item |
| 2.0 Assessor's Judgement | High |
| 2.0 General Notes | Even if participants were most likely aware of allocation there was no evidence to suggest deviations from the trial context. There are not enough information to evaluate the analysis method. |
| **Domain 3: Missing outcome data** |  |
| 3.1 | PY; N |
| Note for 3.1 | 3.1a: PY - no evidence to suggest cluster loss; 3.1b: N - 147 participants out of 323 were not analyzed at endline because Quote"they had not taken part in the program or had sent someone else to complete the questionnaire, or when the ID number was missing." |
| 3.2 | PN |
| Note for 3.2 | Quote: " The consequence of the exclusion of these participants was a low n, which represents a risk for biased data." |
| 3.3 | NI |
| Note for 3.3&3.4 | Not enough information provided to give a judjment on this item |
| 3.4 | NI |
| 3.0 Assessor's judgement | High |
| 3.0 Gerenal notes | Data was not available for almost all participants, all clusters and participants seem to have been analysed. |
| **Domain 4: Risk of bias in measurement of the outcome** |  |
| 4.1 | PN |
| Note for 4.1 | The outcome measure was validated for use in other low resource settings |
| 4.2 | PN |
| Note for 4.2 | No evidence to suggest differences in measurement between intervention and control. |
| 4.3 | NI; PY |
| Note for 4.3 | No information provided on whether participants knew that they were in a trial, participants (outcome assessors) were most likely aware of the group allocation due to the nature of the intervention. |
| 4.4 | PY |
| Note for 4.4&4.5 | Knowledge of the assigned intervention could influence participant-reported outcomes, but there is no reason to believe that it did. |
| 4.5 | PN |
| 4.0 Assessor's Judgement | Some concerns |
| 4.0 General note | There is no evidence to suggest that the outcome measure was inappropriate and that measurement could have differed between groups. No information is provided on whether participants were aware that a trial was taking place, but it is likely that they were aware of their assigned intervention. There is no reason to believe that this knowledge would have influenced outcome assessment. |
| **Domain 5: Risk of bias in selection of the reported result** |  |
| 5.1 | PY |
| Note for 5.1 | No evidence to suggest otherwise, methods and results do not show discrepancies |
| 5.2 | PN |
| Note for 5.2 | No evidence to suggest outcomes selection. All outcomes mentioned in the methods and results were reported. |
| 5.3 | PN |
| Note for 5.3 | No evidence to suggest analyses selection. All outcomes mentioned in the methods and results were reported. |
| 5.0 Assessor's Judgement | Low |
| 5.0 General note | No evidence to suggest analysis or outcome selection. All outcomes and analyses mentioned in the methods and results were reported. |
| **Overall Risk of Bias** |  |
| Assessor's overall Judgement | High |

| **Study ID** | **Ward 2020** |
| --- | --- |
| *Experimental* | Parenting for Lifelong Health (PLH) for Young Children, |
| *Comparator* | usual care |
| Outcome | Depression diagnosis |
| **Domain 1: Risk of bias arising from the randomization process** |  |
| 1.1 | PY |
| 1.2 | PY |
| Note for 1.1&1.2 | Quote: "Randomization was conducted after data collection by an off-site statistician with no other contact with the trial" |
| 1.3 | PN |
| Note for 1.3 | Baseline characteristics were overall comparable across groups as demonstrated by Table 1 |
| 1.0 Assessor's Judgement | Low |
| **Domain 2: Risk of bias due to deviations from the intended interventions** |  |
| 2.1 | PY |
| 2.2 | PY |
| Note for 2.1&2.2 | Due to the nature of the intervention participants and personnel were moslt likely aware of their assigned intervention |
| 2.3 | PY |
| Note for 2.3 | There is some evidence to suggest possible contamination between the intervention and control group. Quote: "In addition, in the dense living environments of informal settlements, it is possible that there was contamination between intervention and control groups." |
| 2.4 | PY |
| Note for 2.4 | Contamination could have affected the outcomes. |
| 2.5 | PY |
| Note for 2.5 | Contamination is more liekly to affect the participants in the control group. |
| 2.6 | PY |
| Note for 2.6 | No explicit mention of the analysis used to estimate the effect of assignment to intervention. Data was analyzed for almost all randomized participants. Quote: "For caregiver self-report, the follow-up rate was 97.0% at t1 and 91.9% at t" |
| 2.7 | NA |
| 2.0 Assessor's Judgement | High |
| **Domain 3: Missing outcome data** |  |
| 3.1 | PY |
| Note for 3.1 | Data was availalble for almost all randomized participants. Quote: "For caregiver self-report, the follow-up rate was 97.0% at t1 and 91.9% at t" |
| 3.2 | NA |
| 3.3 | NA |
| 3.4 | NA |
| 3.0 Assessor's judgement | Low |
| **Domain 4: Risk of bias in measurement of the outcome** |  |
| 4.1 | PN |
| Note for 4.1 | The selected outcome method is widely validated and established across contexts and demonstrated good psychometric properties in the study |
| 4.2 | PN |
| Note for 4.2 | No evidence to suggest differences in measurement between intervention and control. |
| 4.3 | PY |
| Note for 4.3 | Outcome assessors (participants) were most likely aware of intervention allocation. |
| 4.4 | PY |
| Note for 4.4&4.5 | Knowledge of the assigned intervention could influence participant-reported outcomes, but there is no reason to believe that it did |
| 4.5 | PN |
| 4.0 Assessor's Judgement | Some concerns |
| **Domain 5: Risk of bias in selection of the reported result** |  |
| 5.1 | PY |
| Note for 5.1 | No evidence to suggest otherwise, methods and results do not show discrepancies. |
| 5.2 | PN |
| Note for 5.2 | No evidence to suggest outcome selection. |
| 5.3 | PN |
| Note for 5.3 | No evidence to suggest analyses selection. All analyses mentioned in the methods were reported. |
| 5.0 Assessor's Judgement | Low |
| **Overall Risk of Bias** |  |
| Assessor's overall Judgement | High |

| **Study ID** | **Ward 2020** |
| --- | --- |
| *Experimental* | Parenting for Lifelong Health (PLH) for Young Children |
| *Comparator* | usual care |
| Outcome | Depressive symptoms |
| **Domain 1: Risk of bias arising from the randomization process** |  |
| 1.1 | PY |
| 1.2 | PY |
| Note for 1.1&1.2 | Quote: "Randomization was conducted after data collection by an off-site statistician with no other contact with the trial" |
| 1.3 | PN |
| Note for 1.3 | Baseline characteristics were overall comparable across groups as demonstrated by Table 1 |
| 1.0 Assessor's Judgement | Low |
| **Domain 2: Risk of bias due to deviations from the intended interventions** |  |
| 2.1 | PY |
| 2.2 | PY |
| Note for 2.1&2.2 | Due to the nature of the intervention participants and personnel were moslt likely aware of their assigned intervention |
| 2.3 | PY |
| Note for 2.3 | There is some evidence to suggest possible contamination between the intervention and control group. Quote: "In addition, in the dense living environments of informal settlements, it is possible that there was contamination between intervention and control groups." |
| 2.4 | PY |
| Note for 2.4 | Contamination could have affected the outcomes. |
| 2.5 | PY |
| Note for 2.5 | Contamination is more liekly to affect the participants in the control group. |
| 2.6 | PY |
| Note for 2.6 | No explicit mention of the analysis used to estimate the effect of assignment to intervention. Data was analyzed for almost all randomized participants. Quote: "For caregiver self-report, the follow-up rate was 97.0% at t1 and 91.9% at t" |
| 2.7 | NA |
| 2.0 Assessor's Judgement | High |
| **Domain 3: Missing outcome data** |  |
| 3.1 | PY |
| Note for 3.1 | Data was availalble for almost all randomized participants. Quote: "For caregiver self-report, the follow-up rate was 97.0% at t1 and 91.9% at t" |
| 3.2 | NA |
| 3.3 | NA |
| 3.4 | NA |
| 3.0 Assessor's judgement | Low |
| **Domain 4: Risk of bias in measurement of the outcome** |  |
| 4.1 | PN |
| Note for 4.1 | The selected outcome method is widely validated and established across contexts and demonstrated good psychometric properties in the study |
| 4.2 | PN |
| Note for 4.2 | No evidence to suggest differences in measurement between intervention and control. |
| 4.3 | PY |
| Note for 4.3 | Outcome assessors (participants) were most likely aware of intervention allocation. |
| 4.4 | PY |
| Note for 4.4&4.5 | Knowledge of the assigned intervention could influence participant-reported outcomes, but there is no reason to believe that it did |
| 4.5 | PN |
| 4.0 Assessor's Judgement | Some concerns |
| **Domain 5: Risk of bias in selection of the reported result** |  |
| 5.1 | PY |
| Note for 5.1 | No evidence to suggest otherwise, methods and results do not show discrepancies. |
| 5.2 | PN |
| Note for 5.2 | No evidence to suggest outcome selection. |
| 5.3 | PN |
| Note for 5.3 | No evidence to suggest analyses selection. All analyses mentioned in the methods were reported. |
| 5.0 Assessor's Judgement | Low |
| **Overall Risk of Bias** |  |
| Assessor's overall Judgement | High |

| **Study ID** | **Ward 2020** |
| --- | --- |
| *Experimental* | Parenting for Lifelong Health (PLH) for Young Children |
| *Comparator* | usual care |
| Outcome | PTSD symptoms |
| **Domain 1: Risk of bias arising from the randomization process** |  |
| 1.1 | PY |
| 1.2 | PY |
| Note for 1.1&1.2 | Quote: "Randomization was conducted after data collection by an off-site statistician with no other contact with the trial" |
| 1.3 | PN |
| Note for 1.3 | Baseline characteristics were overall comparable across groups as demonstrated by Table 1 |
| 1.0 Assessor's Judgement | Low |
| **Domain 2: Risk of bias due to deviations from the intended interventions** |  |
| 2.1 | PY |
| 2.2 | PY |
| Note for 2.1&2.2 | Due to the nature of the intervention participants and personnel were moslt likely aware of their assigned intervention |
| 2.3 | PY |
| Note for 2.3 | There is some evidence to suggest possible contamination between the intervention and control group. Quote: "In addition, in the dense living environments of informal settlements, it is possible that there was contamination between intervention and control groups." |
| 2.4 | PY |
| Note for 2.4 | Contamination could have affected the outcomes. |
| 2.5 | PY |
| Note for 2.5 | Contamination is more liekly to affect the participants in the control group. |
| 2.6 | PY |
| Note for 2.6 | No explicit mention of the analysis used to estimate the effect of assignment to intervention. Data was analyzed for almost all randomized participants. Quote: "For caregiver self-report, the follow-up rate was 97.0% at t1 and 91.9% at t" |
| 2.7 | NA |
| 2.0 Assessor's Judgement | High |
| **Domain 3: Missing outcome data** |  |
| 3.1 | PY |
| Note for 3.1 | Data was availalble for almost all randomized participants. Quote: "For caregiver self-report, the follow-up rate was 97.0% at t1 and 91.9% at t" |
| 3.2 | NA |
| 3.3 | NA |
| 3.4 | NA |
| 3.0 Assessor's judgement | Low |
| **Domain 4: Risk of bias in measurement of the outcome** |  |
| 4.1 | PN |
| Note for 4.1 | The selected outcome method is widely validated and established across contexts and demonstrated good psychometric properties in the study |
| 4.2 | PN |
| Note for 4.2 | No evidence to suggest differences in measurement between intervention and control. |
| 4.3 | PY |
| Note for 4.3 | Outcome assessors (participants) were most likely aware of intervention allocation. |
| 4.4 | PY |
| Note for 4.4&4.5 | Knowledge of the assigned intervention could influence participant-reported outcomes, but there is no reason to believe that it did |
| 4.5 | PN |
| 4.0 Assessor's Judgement | Some concerns |
| **Domain 5: Risk of bias in selection of the reported result** |  |
| 5.1 | PY |
| Note for 5.1 | No evidence to suggest otherwise, methods and results do not show discrepancies. |
| 5.2 | PN |
| Note for 5.2 | No evidence to suggest outcome selection. |
| 5.3 | PN |
| Note for 5.3 | No evidence to suggest analyses selection. All analyses mentioned in the methods were reported. |
| 5.0 Assessor's Judgement | Low |
| **Overall Risk of Bias** |  |
| Assessor's overall Judgement | High |

| **Study ID** | **Langer 1996** |
| --- | --- |
| *Experimental* | Psychosocial support |
| *Comparator* | usual care |
| Outcome | Anxiety symptoms |
| **Domain 1: Risk of bias arising from the randomization process** |  |
| 1.1 | Y |
| 1.2 | Y |
| Note for 1.1&1.2 | Quote: "Randomization was carried out by the Data Coordinating Center in Pelotas; a computer-generated code was used for randomization within balanced blocks of 20 women, stratified according to center. A sequence of sealed, opaque envelopes including the randomization codes was used by a single investigator in each hospital to assign the women to treatment groups. A particular woman's group assignment was known only to this investigator, the study supervisor and the home visitor." |
| 1.3 | PN |
| Note for 1.3 | Quote: "The distribution of risk factors and obstetric, demographic and behavioral baseline conditions was similar in the intervention and control groups". Data reported is comparable across groups but no formal testing is reported. |
| 1.0 Assessor's Judgement | Low |
| **Domain 2: Risk of bias due to deviations from the intended interventions** |  |
| 2.1 | PY |
| 2.2 | PY |
| Note for 2.1&2.2 | Due to the nature of the intervention participants and personnel were most likely aware of allocation. |
| 2.3 | PN |
| Note for 2.3 | No evidence to suggest issues to implement intervention |
| 2.4 | NA |
| 2.5 | NA |
| 2.6 | Y |
| Note for 2.6 | Quote: "The data were analyzed on an intention-to-treat basis according to the group to which the women were randomly assigned." |
| 2.7 | NA |
| 2.0 Assessor's Judgement | Low |
| **Domain 3:**  **Missing outcome data** |  |
| 3.1 | PY |
| Note for 3.1 | Out of the 2235 randomized participants, 2026 (9.4%) were assessed at 36 weeks of gestation and 1901 (14.9%) at 40 days after delivery. |
| 3.2 | NA |
| 3.3 | NA |
| 3.4 | NA |
| 3.0 Assessor's judgement | Low |
| **Domain 4:**  **Risk of bias in measurement of the outcome** |  |
| 4.1 | PN |
| Note for 4.1 | The STAI is a widely validated and established measure, validated across contexts. |
| 4.2 | PN |
| Note for 4.2 | No evidence to suggest differences in measurement between intervention and control. |
| 4.3 | PY |
| Note for 4.3 | Outcome assessors (participants) were most likely aware of intervention allocation. |
| 4.4 | PY |
| Note for 4.4&4.5 | Knowledge of the assigned intervention could influence participant-reported outcomes, but there is no reason to believe that it did |
| 4.5 | PN |
| 4.0 Assessor's Judgement | Some concerns |
| **Domain 5:**  **Risk of bias in selection of the reported result** |  |
| 5.1 | PY |
| Note for 5.1 | No evidence to suggest otherwise, methods and results do not show discrepancies. |
| 5.2 | PN |
| Note for 5.2 | No evidence to suggest outcome selection. All outcomes mentioned in the methods are reported. |
| 5.3 | PN |
| Note for 5.3 | No evidence to suggest analyses selection. All analyses mentioned in the methods were reported in the results. |
| 5.0 Assessor's Judgement | Low |
| **Overall Risk of Bias** |  |
| Assessor's overall Judgement | Some concerns |

| **Study ID** | **Sherman 2009** |
| --- | --- |
| *Experimental* | Peer education condition |
| *Comparator* | Enhanced Usual Care - Health Advice |
| Outcome | Depressive symptoms |
| **Domain 1: Risk of bias arising from the randomization process** |  |
| 1.1 | PY |
| 1.2 | PY |
| Note for 1.1&1.2 | Quote: "Randomization of index members occurred at the end of the baseline visit. Indexes were randomised to either the peer education or the life skills condition within 45 days of their baseline visit. Randomization occurred in blocks (cohorts) once a minimum of 16 and a maximum of 24 index participants had been enrolled, and randomisation sequences for each cohort were generated by a computer program." |
| 1.3 | PY |
| Note for 1.3 | Quote: "There were few significant differences in demographic or reported drug use patterns between participants randomised to the peer education compared to the life skills condition. A significantly higher percentage of participants in the peer education condition compared to those in the life skills condition reported drinking problems (77% vs. 71%, p<0.05), condom use at last vaginal sex act (38% vs. 31%, p<0.05) and "always" using condoms in the past 30 days (22% vs. 16%, p<0.05)". The imbalances in the baseline measures for outcomes variables could result in bias in the intervention effect estimates. |
| 1.0 Assessor's Judgement | Some concerns |
| **Domain 2: Risk of bias due to deviations from the intended interventions** |  |
| 2.1 | PY |
| 2.2 | PY |
| Note for 2.1&2.2 | Due to the nature of the intervention participants and personnel were most likely aware of allocation |
| 2.3 | PY |
| Note for 2.3 | Quote: "There is the possibility that tight social networks were randomised to both control and intervention arms, leading to a high degree of contamination that resulted in a bias towards the null"; "It is highly probable that contamination occurred between the two study arms. Based on our observation at the study house, many participants enrolled in the study with or were referred to the study by their friends who could have been randomized to different study arms" |
| 2.4 | PY |
| Note for 2.4 | Contamination could have affected the outcomes. |
| 2.5 | PY |
| Note for 2.5 | Contamination is more liekly to affect the participants in the control group. |
| 2.6 | PY |
| Note for 2.6 | The analysis was carried out on an intention to treat basis |
| 2.7 | NA |
| 2.0 Assessor's Judgement | Some concerns |
| **Domain 3: Missing outcome data** |  |
| 3.1 | PY |
| Note for 3.1 | Quote: "At each of the four follow-up visits, follow-up was greater or equal to 90% (range: 89% – 95%) for index participants and 86% (range: 85% – 91%) for network participants in both arms. Among index and network members in both arms, there was at least an 89% retention rate at the 12-month follow-up" |
| 3.2 | NA |
| 3.3 | NA |
| 3.4 | NA |
| 3.0 Assessor's judgement | Low |
| **Domain 4: Risk of bias in measurement of the outcome** |  |
| 4.1 | PN |
| Note for 4.1 | The selected outcome method (CES-D) is widely validated and established, appropriate for the local context, and assessed in its psychometric properties for this sample. |
| 4.2 | PN |
| Note for 4.2 | No evidence to suggest differences in measurement between intervention and control. |
| 4.3 | PY |
| Note for 4.3 | Outcome assessors (participants) were most likely aware of intervention allocation. Interviewers were blind to allocation. |
| 4.4 | PY |
| Note for 4.4&4.5 | Knowledge of the assigned intervention could influence participant-reported outcomes, but there is no reason to believe that it did |
| 4.5 | PN |
| 4.0 Assessor's Judgement | Some concerns |
| **Domain 5: Risk of bias in selection of the reported result** |  |
| 5.1 | PY |
| Note for 5.1 | No evidence to suggest otherwise, methods and results do not show discrepancies. |
| 5.2 | PN |
| Note for 5.2 | No evidence to suggest outcome selection. All outcomes mentioned in the methods are reported. |
| 5.3 | PN |
| Note for 5.3 | No evidence to suggest analyses selection. All analyses mentioned in the methods were reported. |
| 5.0 Assessor's Judgement | Low |
| **Overall Risk of Bias** |  |
| Assessor's overall Judgement | Some concerns |

| **Study ID** | **Yeomans 2010** |
| --- | --- |
| *Experimental* | Workshop with PTSD Psychoeducation |
| *Comparator* | Wait list control group |
| Outcome | Depressive symptoms |
| **Domain 1: Risk of bias arising from the randomization process** |  |
| 1.1 | Y |
| 1.2 | NI |
| Note for 1.1&1.2 | Title mentions RCT design. No further information. |
| 1.3 | N |
| Note for 1.3 | Quote: "There were no significant baseline differences between the three treatment groups across age, gender, ethnicity, symptoms, education level, traumatic events experienced, or on prior exposure to trauma discourse" |
| 1.0 Assessor's Judgement | Low |
| **Domain 2: Risk of bias due to deviations from the intended interventions** |  |
| 2.1 | Y |
| 2.2 | PY |
| Note for 2.1&2.2 | Quote: "Participants (...) were informed of random allocation procedures". Due to the nature of the intervention, the interventionists were most likely aware of the allocation. |
| 2.3 | PN |
| Note for 2.3 | No evidence to suggest issues to implement intervention |
| 2.4 | NA |
| 2.5 | NA |
| 2.6 | NI |
| Note for 2.6 | No explicit mention of the analysis used to estimate the effect of assignment to intervention |
| 2.7 | N |
| Note for 2.7 | There was not a substantial impact of the failure to analyse participants in the group to which they were randomized |
| 2.0 Assessor's Judgement | Some concerns |
| **Domain 3: Missing outcome data** |  |
| 3.1 | Y |
| Note for 3.1 | Lost to follow-up: 7 of 120 |
| 3.2 | NA |
| 3.3 | NA |
| 3.4 | NA |
| 3.0 Assessor's judgement | Low |
| **Domain 4: Risk of bias in measurement of the outcome** |  |
| 4.1 | N |
| Note for 4.1 | The selected outcome method (HSCL-25) is widely validated and established across multiple languages. |
| 4.2 | PN |
| Note for 4.2 | No evidence to suggest differences in measurement between intervention and control. |
| 4.3 | PY |
| Note for 4.3 | Outcome assessors (participants) were most likely aware of intervention allocation. |
| 4.4 | PY |
| Note for 4.4&4.5 | Knowledge of the assigned intervention could influence participant-reported outcomes, but there is no reason to believe that it did |
| 4.5 | PN |
| 4.0 Assessor's Judgement | Some concerns |
| **Domain 5: Risk of bias in selection of the reported result** |  |
| 5.1 | PY |
| Note for 5.1 | No evidence to suggest otherwise, methods and results do not show discrepancies. |
| 5.2 | PN |
| Note for 5.2 | No evidence to suggest outcome selection. All outcomes mentioned in the methods and results were reported. |
| 5.3 | PN |
| Note for 5.3 | No evidence to suggest analyses selection. All outcomes mentioned in the methods and results were reported. |
| 5.0 Assessor's Judgement | Low |
| **Overall Risk of Bias** |  |
| Assessor's overall Judgement | Some concerns |

| **Study ID** | **Yeomans 2010** |
| --- | --- |
| *Experimental* | Workshop with PTSD Psychoeducation |
| *Comparator* | Wait list control group |
| Outcome | Depression diagnosis |
| **Domain 1: Risk of bias arising from the randomization process** |  |
| 1.1 | Y |
| 1.2 | NI |
| Note for 1.1&1.2 | Title mentions RCT design. No further information. |
| 1.3 | N |
| Note for 1.3 | Quote: "There were no significant baseline differences between the three treatment groups across age, gender, ethnicity, symptoms, education level, traumatic events experienced, or on prior exposure to trauma discourse" |
| 1.0 Assessor's Judgement | Low |
| **Domain 2: Risk of bias due to deviations from the intended interventions** |  |
| 2.1 | Y |
| 2.2 | PY |
| Note for 2.1&2.2 | Quote: "Participants (...) were informed of random allocation procedures". Due to the nature of the intervention, the interventionists were most likely aware of the allocation. |
| 2.3 | PN |
| Note for 2.3 | No evidence to suggest issues to implement intervention |
| 2.4 | NA |
| 2.5 | NA |
| 2.6 | NI |
| Note for 2.6 | No explicit mention of the analysis used to estimate the effect of assignment to intervention |
| 2.7 | N |
| Note for 2.7 | There was not a substantial impact of the failure to analyse participants in the group to which they were randomized |
| 2.0 Assessor's Judgement | Some concerns |
| **Domain 3: Missing outcome data** |  |
| 3.1 | Y |
| Note for 3.1 | Lost to follow-up: 7 of 120 |
| 3.2 | NA |
| 3.3 | NA |
| 3.4 | NA |
| 3.0 Assessor's judgement | Low |
| **Domain 4: Risk of bias in measurement of the outcome** |  |
| 4.1 | N |
| Note for 4.1 | The selected outcome method (HSCL-25) is widely validated and established across multiple languages. |
| 4.2 | PN |
| Note for 4.2 | No evidence to suggest differences in measurement between intervention and control. |
| 4.3 | PY |
| Note for 4.3 | Outcome assessors (participants) were most likely aware of intervention allocation. |
| 4.4 | PY |
| Note for 4.4&4.5 | Knowledge of the assigned intervention could influence participant-reported outcomes, but there is no reason to believe that it did |
| 4.5 | PN |
| 4.0 Assessor's Judgement | Some concerns |
| **Domain 5: Risk of bias in selection of the reported result** |  |
| 5.1 | PY |
| Note for 5.1 | No evidence to suggest otherwise, methods and results do not show discrepancies. |
| 5.2 | PN |
| Note for 5.2 | No evidence to suggest outcome selection. All outcomes mentioned in the methods and results were reported. |
| 5.3 | PN |
| Note for 5.3 | No evidence to suggest analyses selection. All outcomes mentioned in the methods and results were reported. |
| 5.0 Assessor's Judgement | Low |
| **Overall Risk of Bias** |  |
| Assessor's overall Judgement | Some concerns |

| **Study ID** | **Yeomans 2010** |
| --- | --- |
| *Experimental* | Workshop with PTSD Psychoeducation |
| *Comparator* | Wait list control group |
| Outcome | PTSD symptoms |
| **Domain 1: Risk of bias arising from the randomization process** |  |
| 1.1 | Y |
| 1.2 | NI |
| Note for 1.1&1.2 | Title mentions RCT design. No further information. |
| 1.3 | N |
| Note for 1.3 | Quote: "There were no significant baseline differences between the three treatment groups across age, gender, ethnicity, symptoms, education level, traumatic events experienced, or on prior exposure to trauma discourse" |
| 1.0 Assessor's Judgement | Low |
| **Domain 2: Risk of bias due to deviations from the intended interventions** |  |
| 2.1 | Y |
| 2.2 | PY |
| Note for 2.1&2.2 | Quote: "Participants were informed pf randm allocation procedures". Due to the nature of the intervention the interventionists were most likely aware of the allocation. |
| 2.3 | PN |
| Note for 2.3 | No evidence to suggest issues to implement intervention |
| 2.4 | NA |
| 2.5 | NA |
| 2.6 | NI |
| Note for 2.6 | No explicit mention of the analysis used to estimate the effect of assignment to intervention |
| 2.7 | N |
| Note for 2.7 | There was not a substantial impact of the failure to analyse participants in the group to which they were randomized |
| 2.0 Assessor's Judgement | Some concerns |
| **Domain 3: Missing outcome data** |  |
| 3.1 | Y |
| Note for 3.1 | Lost to follow-up: 7 of 120 |
| 3.2 | NA |
| 3.3 | NA |
| 3.4 | NA |
| 3.0 Assessor's judgement | Low |
| **Domain 4: Risk of bias in measurement of the outcome** |  |
| 4.1 | N |
| Note for 4.1 | The selected outcome method (HTQ) is widely validated and established. |
| 4.2 | PN |
| Note for 4.2 | No evidence to suggest differences in measurement between intervention and control. |
| 4.3 | PY |
| Note for 4.3 | Outcome assessors (participants) were most likely aware of intervention allocation. |
| 4.4 | PY |
| Note for 4.4&4.5 | Knowledge of the assigned intervention could influence participant-reported outcomes, but there is no reason to believe that it did |
| 4.5 | PN |
| 4.0 Assessor's Judgement | Some concerns |
| **Domain 5: Risk of bias in selection of the reported result** |  |
| 5.1 | PY |
| Note for 5.1 | No evidence to suggest otherwise, methods and results do not show discrepancies. |
| 5.2 | PN |
| Note for 5.2 | No evidence to suggest outcome selection. All outcomes mentioned in the methods and results were reported. |
| 5.3 | PN |
| Note for 5.3 | No evidence to suggest analyses selection. All outcomes mentioned in the methods and results were reported. |
| 5.0 Assessor's Judgement | Low |
| **Overall Risk of Bias** |  |
| Assessor's overall Judgement | Some concerns |

| **Study ID** | **Yeomans 2010** |
| --- | --- |
| *Experimental* | Workshop with PTSD Psychoeducation |
| *Comparator* | Wait list control group |
| Outcome | PTSD diagnosis |
| **Domain 1: Risk of bias arising from the randomization process** |  |
| 1.1 | Y |
| 1.2 | NI |
| Note for 1.1&1.2 | Title mentions RCT design. No further information. |
| 1.3 | N |
| Note for 1.3 | Quote: "There were no significant baseline differences between the three treatment groups across age, gender, ethnicity, symptoms, education level, traumatic events experienced, or on prior exposure to trauma discourse" |
| 1.0 Assessor's Judgement | Low |
| **Domain 2: Risk of bias due to deviations from the intended interventions** |  |
| 2.1 | Y |
| 2.2 | PY |
| Note for 2.1&2.2 | Quote: "Participants were informed pf randm allocation procedures". Due to the nature of the intervention the interventionists were most likely aware of the allocation. |
| 2.3 | PN |
| Note for 2.3 | No evidence to suggest issues to implement intervention |
| 2.4 | NA |
| 2.5 | NA |
| 2.6 | NI |
| Note for 2.6 | No explicit mention of the analysis used to estimate the effect of assignment to intervention |
| 2.7 | N |
| Note for 2.7 | There was not a substantial impact of the failure to analyse participants in the group to which they were randomized |
| 2.0 Assessor's Judgement | Some concerns |
| **Domain 3: Missing outcome data** |  |
| 3.1 | Y |
| Note for 3.1 | Lost to follow-up: 7 of 120 |
| 3.2 | NA |
| 3.3 | NA |
| 3.4 | NA |
| 3.0 Assessor's judgement | Low |
| **Domain 4: Risk of bias in measurement of the outcome** |  |
| 4.1 | N |
| Note for 4.1 | The selected outcome method (HTQ) is widely validated and established. |
| 4.2 | PN |
| Note for 4.2 | No evidence to suggest differences in measurement between intervention and control. |
| 4.3 | PY |
| Note for 4.3 | Outcome assessors (participants) were most likely aware of intervention allocation. |
| 4.4 | PY |
| Note for 4.4&4.5 | Knowledge of the assigned intervention could influence participant-reported outcomes, but there is no reason to believe that it did |
| 4.5 | PN |
| 4.0 Assessor's Judgement | Some concerns |
| **Domain 5: Risk of bias in selection of the reported result** |  |
| 5.1 | PY |
| Note for 5.1 | No evidence to suggest otherwise, methods and results do not show discrepancies. |
| 5.2 | PN |
| Note for 5.2 | No evidence to suggest outcome selection. All outcomes mentioned in the methods and results were reported. |
| 5.3 | PN |
| Note for 5.3 | No evidence to suggest analyses selection. All outcomes mentioned in the methods and results were reported. |
| 5.0 Assessor's Judgement | Low |
| **Overall Risk of Bias** |  |
| Assessor's overall Judgement | Some concerns |

| **Study ID** | **Dybdahl 2001** |
| --- | --- |
| *Experimental* | Psychosocial Intervention Program |
| *Comparator* | Usual care |
| Outcome | PTSD symptoms |
| **Domain 1: Risk of bias arising from the randomization process** |  |
| 1.1 | Y |
| 1.2 | PY |
| Note for 1.1&1.2 | Quote: Tha assignment was random. All the names of the mothers-child dyads were written on pieces of papaer, which were folded, mixed together, and then separated into two piles at random so that one pile formed the intervention group and the other pile formed the control group" |
| 1.3 | PN |
| Note for 1.3 | Ntot significant differences at baseline. |
| 1.0 Assessor's Judgement | Low |
| **Domain 2: Risk of bias due to deviations from the intended interventions** |  |
| 2.1 | PY |
| 2.2 | PY |
| Note for 2.1&2.2 | Due to the nature of the intervention participants were most likely aware of their assigned intervention. |
| 2.3 | NI |
| Note for 2.3 | No information provided. |
| 2.4 | NA |
| 2.5 | NA |
| 2.6 | NI |
| Note for 2.6 | No explicit mention of the analysis used to estimate the effect of assignment to intervention. |
| 2.7 | NI |
| Note for 2.7 | No information provided. |
| 2.0 Assessor's Judgement | High |
| **Domain 3: Missing outcome data** |  |
| 3.1 | PN |
| Note for 3.1 | Quote: "Twelve of the families dropped out of the study and did not participate in scheduled interventions: 7 from intervention group, and 5 from the control gorip". Quote: "Several of the mothers and children did not complete all tests at both tests periods for a variety of reasons; thus the number of participants varied from test to test". |
| 3.2 | NI |
| Note for 3.2 | No information provided. |
| 3.3 | NI |
| Note for 3.3&3.4 | No information provided. |
| 3.4 | NI |
| 3.0 Assessor's judgement | High |
| **Domain 4: Risk of bias in measurement of the outcome** |  |
| 4.1 | N |
| Note for 4.1 | The selected outcome method is widely validated and established. |
| 4.2 | NI |
| Note for 4.2 | No information provided. |
| 4.3 | N |
| Note for 4.3 | Quote: "The third and fourth teams carried out the evaluations. They Were blind with respect to which families were in the intervention or control groups, as were the physicians who provided the medican checkups". |
| 4.4 | NA |
| 4.5 | NA |
| 4.0 Assessor's Judgement | Some concerns |
| **Domain 5: Risk of bias in selection of the reported result** |  |
| 5.1 | PN |
| Note for 5.1 | No protocol available. No evidence to suggest otherwise, methods and results do not show discrepancies. |
| 5.2 | PN |
| Note for 5.2 | No evidence to suggest outcome selection. |
| 5.3 | PN |
| Note for 5.3 | No evidence to suggest analyses selection. |
| 5.0 Assessor's Judgement | Some concerns |
| **Overall Risk of Bias** |  |
| Assessor's overall Judgement | High |

| **Study ID** | **Devries 2015** |
| --- | --- |
| *Experimental* | Good School Toolkit |
| *Comparator* | Usual care |
| Outcome | PTSD diagnosis |
| **Domain 1a:**  **Risk of bias arising from the randomization process** |  |
| 1a.1 | Y |
| 1a.2 | PN |
| Note for 1a.1&1a.2 | Title mentions RCT design.  Quote: "We randomly selected 42 primary schools (clusters)".  Quote: "Owing to the nature of the intervention, it was not possible to mask participants. Allocation was not intentionally revealed to those collecting data; however, given the nature of the intervention, they should also be regarded unmasked." |
| 1a.3 | PN |
| Note for 1a.3 | Not significant differences at baseline |
| 1a.0 Assessor's Judgement | High |
| **Domain 1b: Risk of bias arising from the timing of identification or recruitment of participants** |  |
| 1b.1 | N |
| Note for 1b.1 | Participants were recruited after randomization. |
| 1b.2 | Y |
| Note for 1b.2 | Quote: "Owing to the nature of the intervention, it was not possible to mask participants" |
| 1b.0 Assessor's Judgement | High |
| **Domain 2: Risk of bias due to deviations from the intended interventions** |  |
| 2.1a | PY |
| Note for 2.1a | Participants provided the informed consent. |
| 2.1b (2.1) | Y |
| 2.2 | PY |
| Note for 2.1b&2.2 | Quote: "Owing to the nature of the intervention, it was not possible to mask participants"; "During the trial, we recorded one major incident of contamination, where an intervention school invited head teachers from three neighbouring control schools to an event about the Toolkit." |
| 2.3 | PY |
| Note for 2.3 | Quote: "No major changes to the trial protocol were made. We made changes to our child protection referral strategy after baseline survey" |
| 2.4 | PN |
| Note for 2.4 | No evidence to suggest that deviations could have affected the outcome. |
| 2.5 | NA |
| 2.6 | Y |
| Note for 2.6 | Quote: "We did an intention to treat analysis" |
| 2.7 | NA |
| 2.0 Assessor's Judgement | Some concerns |
| **Domain 3: Missing outcome data** |  |
| 3.1a | Y |
| Note for 3.1a | Quote: "No schools (clusters) left the study" |
| 3.1b | PY |
| Note for 3.1b | Quote: "At 18-month follow-up, 3820 (92.4%) of 4138 randomly sampled students participated in a cross-sectional survey" |
| 3.2 | NA |
| 3.3 | NA |
| 3.4 | NA |
| 3.0 Assessor's judgement | Low |
| **Domain 4: Risk of bias in measurement of the outcome** |  |
| 4.1 | N |
| Note for 4.1 | Quote: "All were measured with instruments widely used internationally which have been validated in a variety of settings" |
| 4.2 | PN |
| Note for 4.2 | No evidence to suggest differences in measurement between intervention and control |
| 4.3a | PY |
| Note for 4.3a | Quote: "Allocation was not intentionally revealed to those collecting dta; however, gien the nature of the intervention, they should also be regarded unmasked." |
| 4.3b | PY |
| Note for 4.3b | Quote: "Allocation was not intentionally revealed to those collecting dta; however, gien the nature of the intervention, they should also be regarded unmasked." |
| 4.4 | PY |
| 4.5 | PN |
| Note for 4.4&4.5 | Knowledge of the nature of the assigned intevrention could influence participant-reported outcomes, but probably no. |
| 4.0 Assessor's Judgement | Some concerns |
| **Domain 5:**  **Risk of bias in selection of the reported result** |  |
| 5.1 | PY |
| Note for 5.1 | Protocol is available. No evidence to suggest otherwise, protocol and manuscript do not show discrepancies. |
| 5.2 | PN |
| Note for 5.2 | No evidence to suggest outcome selection. All outcomes mentioned in the methods and protocol were reported. |
| 5.3 | PN |
| Note for 5.3 | No evidence to suggest analyses selection. All outcomes mentioned in the methods and results were reported. |
| 5.0 Assessor's Judgement | Low |
| **Overall Risk of Bias** |  |
| Assessor's overall Judgement | High |

| **Study ID** | **Devries 2015** |
| --- | --- |
| *Experimental* | Good School Toolkit |
| *Comparator* | Usual care |
| Outcome | PTSD symptoms |
| **Domain 1a:**  **Risk of bias arising from the randomization process** |  |
| 1a.1 | Y |
| 1a.2 | PN |
| Note for 1a.1&1a.2 | Title mentions RCT design.  Quote: "We randomly selected 42 primary schools (clusters)".  Quote: "Owing to the nature of the intervention, it was not possible to mask participants. Allocation was not intentionally revealed to those collecting data; however, given the nature of the intervention, they should also be regarded unmasked." |
| 1a.3 | PN |
| Note for 1a.3 | Not significant differences at baseline |
| 1a.0 Assessor's Judgement | High |
| **Domain 1b: Risk of bias arising from the timing of identification or recruitment of participants** |  |
| 1b.1 | N |
| Note for 1b.1 | Participants were recruited after randomization. |
| 1b.2 | Y |
| Note for 1b.2 | Quote: "Owing to the nature of the intervention, it was not possible to mask participants" |
| 1b.0 Assessor's Judgement | High |
| **Domain 2: Risk of bias due to deviations from the intended interventions** |  |
| 2.1a | PY |
| Note for 2.1a | Participants provided the informed consent. |
| 2.1b (2.1) | Y |
| 2.2 | PY |
| Note for 2.1b&2.2 | Quote: "Owing to the nature of the intervention, it was not possible to mask participants"; "During the trial, we recorded one major incident of contamination, where an intervention school invited head teachers from three neighbouring control schools to an event about the Toolkit." |
| 2.3 | PY |
| Note for 2.3 | Quote: "No major changes to the trial protocol were made. We made changes to our child protection referral strategy after baseline survey" |
| 2.4 | PN |
| Note for 2.4 | No evidence to suggest that deviations could have affected the outcome. |
| 2.5 | NA |
| 2.6 | Y |
| Note for 2.6 | Quote: "We did an intention to treat analysis" |
| 2.7 | NA |
| 2.0 Assessor's Judgement | Some concerns |
| **Domain 3: Missing outcome data** |  |
| 3.1a | Y |
| Note for 3.1a | Quote: "No schools (clusters) left the study" |
| 3.1b | PY |
| Note for 3.1b | Quote: "At 18-month follow-up, 3820 (92.4%) of 4138 randomly sampled students participated in a cross-sectional survey" |
| 3.2 | NA |
| 3.3 | NA |
| 3.4 | NA |
| 3.0 Assessor's judgement | Low |
| **Domain 4: Risk of bias in measurement of the outcome** |  |
| 4.1 | N |
| Note for 4.1 | Quote: "All were measured with instruments widely used internationally which have been validated in a variety of settings" |
| 4.2 | PN |
| Note for 4.2 | No evidence to suggest differences in measurement between intervention and control |
| 4.3a | PY |
| Note for 4.3a | Quote: "Allocation was not intentionally revealed to those collecting dta; however, gien the nature of the intervention, they should also be regarded unmasked." |
| 4.3b | PY |
| Note for 4.3b | Quote: "Allocation was not intentionally revealed to those collecting dta; however, gien the nature of the intervention, they should also be regarded unmasked." |
| 4.4 | PY |
| 4.5 | PN |
| Note for 4.4&4.5 | Knowledge of the nature of the assigned intevrention could influence participant-reported outcomes, but probably no. |
| 4.0 Assessor's Judgement | Some concerns |
| **Domain 5:**  **Risk of bias in selection of the reported result** |  |
| 5.1 | PY |
| Note for 5.1 | Protocol is available. No evidence to suggest otherwise, protocol and manuscript do not show discrepancies. |
| 5.2 | PN |
| Note for 5.2 | No evidence to suggest outcome selection. All outcomes mentioned in the methods and protocol were reported. |
| 5.3 | PN |
| Note for 5.3 | No evidence to suggest analyses selection. All outcomes mentioned in the methods and results were reported. |
| 5.0 Assessor's Judgement | Low |
| **Overall Risk of Bias** |  |
| Assessor's overall Judgement | High |

| **Study ID** | **Dhital 2019** |
| --- | --- |
| *Experimental* | Psycho-social support by teachers |
| *Comparator* | Usual care |
| Outcome | PTSD symptoms |
| **Domain 1a:**  **Risk of bias arising from the randomization process** |  |
| 1a.1 | Y |
| 1a.2 | PY |
| Note for 1a.1&1a.2 | Title mentions RCT design.  It was used a random component (opaque envelops). Quote: "Each pair of schools were grouped together and one school from each pair was randomly assigned to either hroup A or B" |
| 1a.3 | N |
| Note for 1a.3 | Quote: "No significant difference was identified between intervention and control group" |
| 1a.0 Assessor's Judgement | Low |
| **Domain 1b: Risk of bias arising from the timing of identification or recruitment of participants** |  |
| 1b.1 | PY |
| Note for 1b.1 | First, adolescents were considered eligible to partecipate, then were selected throught simple random sampling, and then there was a randomization process of the clusters. |
| 1b.2 | PY |
| 1b.0 Assessor's Judgement | Low |
| **Domain 2: Risk of bias due to deviations from the intended interventions** |  |
| 2.1a | PY |
| Note for 2.1a | Quote: "Written informed consnet were provided by the adolescents who agreed to participate in the study and their guardians" |
| 2.1b (2.1) | PY |
| 2.2 | PY |
| Note for 2.1b&2.2 | Quote: "Blinding was not done for the intervention because all schools were required to be informed about the intervention"  People delivering the intervention (teachers) were trained for doing that. They were most likely aware of their assigned intervention |
| 2.3 | PN |
| Note for 2.3 | No evidence to suggest deviations from the intended intervention taht arose because of the trial context. |
| 2.4 | NA |
| 2.5 | NA |
| 2.6 | NI |
| Note for 2.6 | No information provided. |
| 2.7 | PN |
| Note for 2.7 | There is no reason to believe that it did. |
| 2.0 Assessor's Judgement | Some concerns |
| **Domain 3: Missing outcome data** |  |
| 3.1a | PY |
| 3.1b | PY |
| Note for 3.1b | Dropout rate: 16% for control group; 8 % for intervention group |
| 3.2 | NA |
| 3.3 | NA |
| 3.4 | NA |
| 3.0 Assessor's judgement | Low |
| **Domain 4: Risk of bias in measurement of the outcome** |  |
| 4.1 | N |
| Note for 4.1 | Quote: "We used validated tools (CPSS) to measure the symptoms scores. (...) The scale has been validated in Nepal" |
| 4.2 | PN |
| Note for 4.2 | No evidence to suggest differences in measurement between intervention and control |
| 4.3a | Y |
| Note for 4.3a | Quote: "the research assistants were trained on data collection and ethical procedures" |
| 4.3b | PY |
| Note for 4.3b | Due to the nature of intervention, ouctome assessors (research assistants + participants) ere most likely aware of the intervention allocation. |
| 4.4 | PY |
| 4.5 | PN |
| Note for 4.4&4.5 | Knowledge of the assigned interventio could influence participant-reported outcomes, but there is no reason to believe that it did. |
| 4.0 Assessor's Judgement | Some concerns |
| **Domain 5:**  **Risk of bias in selection of the reported result** |  |
| 5.1 | PN |
| Note for 5.1 | Protocol is available. No evidence to suggest otherwise, protocol and manuscript do not show discrepancies. |
| 5.2 | Y |
| Note for 5.2 | Quote: "According to the original protocol, this study had aimed at collecting data at 3 months follow-up after the intervention. However, the 3 month follow up was cancelled at the time of data collection due to feasibility problems. |
| 5.3 | PN |
| Note for 5.3 | No evidence to suggest analyses selection. All outcomes mentioned in the methods and results were reported. |
| 5.0 Assessor's Judgement | High |
| **Overall Risk of Bias** |  |
| Assessor's overall Judgement | High |

| **Study ID** | **Dhital 2019** |
| --- | --- |
| *Experimental* | Psycho-social support by teachers |
| *Comparator* | Usual care |
| Outcome | Depressive symptoms |
| **Domain 1a:**  **Risk of bias arising from the randomization process** |  |
| 1a.1 | Y |
| 1a.2 | PY |
| Note for 1a.1&1a.2 | Title mentions RCT design.  It was used a random component (opaque envelops). Quote: "Each pair of schools were grouped together and one school from each pair was randomly assigned to either hroup A or B" |
| 1a.3 | N |
| Note for 1a.3 | Quote: "No significant difference was identified between intervention and control group" |
| 1a.0 Assessor's Judgement | Low |
| **Domain 1b: Risk of bias arising from the timing of identification or recruitment of participants** |  |
| 1b.1 | PY |
| Note for 1b.1 | First, adolescents were considered eligible to partecipate, then were selected throught simple random sampling, and then there was a randomization process of the clusters. |
| 1b.2 | PY |
| 1b.0 Assessor's Judgement | Low |
| **Domain 2: Risk of bias due to deviations from the intended interventions** |  |
| 2.1a | PY |
| Note for 2.1a | Quote: "Written informed consnet were provided by the adolescents who agreed to participate in the study and their guardians" |
| 2.1b (2.1) | PY |
| 2.2 | PY |
| Note for 2.1b&2.2 | Quote: "Blinding was not done for the intervention because all schools were required to be informed about the intervention"  People delivering the intervention (teachers) were trained for doing that. They were most likely aware of their assigned intervention |
| 2.3 | PN |
| Note for 2.3 | No evidence to suggest deviations from the intended intervention taht arose because of the trial context. |
| 2.4 | NA |
| 2.5 | NA |
| 2.6 | NI |
| Note for 2.6 | No information provided. |
| 2.7 | PN |
| Note for 2.7 | There is no reason to believe that it did. |
| 2.0 Assessor's Judgement | Some concerns |
| **Domain 3: Missing outcome data** |  |
| 3.1a | PY |
| 3.1b | PY |
| Note for 3.1b | Dropout rate: 16% for control group; 8 % for intervention group |
| 3.2 | NA |
| 3.3 | NA |
| 3.4 | NA |
| 3.0 Assessor's judgement | Low |
| **Domain 4: Risk of bias in measurement of the outcome** |  |
| 4.1 | N |
| Note for 4.1 | Quote: "We used validated tools (Depression Self-rating Scale) to measure the symptoms scores. (...) The scale has been validated in Nepal" |
| 4.2 | PN |
| Note for 4.2 | No evidence to suggest differences in measurement between intervention and control |
| 4.3a | Y |
| Note for 4.3a | Quote: "the research assistants were trained on data collection and ethical procedures" |
| 4.3b | PY |
| Note for 4.3b | Due to the nature of intervention, ouctome assessors (research assistants + participants) ere most likely aware of the intervention allocation. |
| 4.4 | PY |
| 4.5 | PN |
| Note for 4.4&4.5 | Knowledge of the assigned interventio could influence participant-reported outcomes, but there is no reason to believe that it did. |
| 4.0 Assessor's Judgement | Some concerns |
| **Domain 5:**  **Risk of bias in selection of the reported result** |  |
| 5.1 | PN |
| Note for 5.1 | Protocol is available. No evidence to suggest otherwise, protocol and manuscript do not show discrepancies. |
| 5.2 | Y |
| Note for 5.2 | Quote: "According to the original protocol, this study had aimed at collecting data at 3 months follow-up after the intervention. However, the 3 month follow up was cancelled at the time of data collection due to feasibility problems. |
| 5.3 | PN |
| Note for 5.3 | No evidence to suggest analyses selection. All outcomes mentioned in the methods and results were reported. |
| 5.0 Assessor's Judgement | High |
| **Overall Risk of Bias** |  |
| Assessor's overall Judgement | High |

| **Study ID** | **Baker Henningham 2019** |
| --- | --- |
| *Experimental* | Violence prevention |
| *Comparator* | usual care |
| Outcome | Depressive symptoms |
| **Domain 1a:**  **Risk of bias arising from the randomization process** |  |
| 1a.1 | PY |
| 1a.2 | Y |
| Note for 1a.1&1a.2 | Quote: "Schools were randomised to intervention or control group in the summer preceding the intervention. Randomisation was conducted by an independent statistician who was blind. Schools were randomised to intervention or control group in the summer preceding the schools." |
| 1a.3 | NI |
| Note for 1a.3 | No useful information is reported to evaluate this element |
| 1a.0 Assessor's Judgement | Low |
| **Domain 1b: Risk of bias arising from the timing of identification or recruitment of participants** |  |
| 1b.1 | PY |
| Note for 1b.1 | Schools and teachers were identified and rectruited before the randomization of clusters, children from the teacher's classes were randomly selected at a later stage (outcome of interest refers to teachers |
| 1b.2 | NA |
| 1b.3 | NA |
| 1b.0 Assessor's Judgement | Low |
| **Domain 2: Risk of bias due to deviations from the intended interventions** |  |
| 2.1a | NI |
| Note for 2.1a | No information provided on whether participants knew that they were in a trial |
| 2.1b (2.1) | PY |
| 2.2 | PY |
| Note for 2.1b&2.2 | Due to the nature of the intervention, participants and personnel were most likely aware of the treatment allocation. |
| 2.3 | PN |
| Note for 2.3 | No evidence to suggest deviations from the intended intervention that arose because of the trial context |
| 2.4 | NA |
| 2.5 | NA |
| 2.6 | Y |
| Note for 2.6 | Data were analysed on an intention-to-treat basis. |
| 2.7 | NA |
| 2.0 Assessor's Judgement | Low |
| 2.0 General Notes | Even if participants were most likely aware of allocation there was no evidence to suggest deviations from the trial context. An appropriate analysis to estimate the effect of assignment was conducted. |
| **Domain 3: Missing outcome data** |  |
| 3.1a | PY |
| Note for 3.1a | all 14 clusters, schools, that recruited participants were analyzed |
| 3.1b | Y |
| Note for 3.1b | there was 1 loss among teachers (out of 54), no reported loss among children |
| 3.2 | NA |
| 3.3 | NA |
| 3.4 | NA |
| 3.0 Assessor's judgement | Low |
| 3.0 General notes | Data was available for almost all participants, all 14 clusters were analysed. |
| **Domain 4: Risk of bias in measurement of the outcome** |  |
| 4.1 | PN |
| Note for 4.1 | The outcome measure (CES-D) is widely established and used across contexts. |
| 4.2 | PN |
| Note for 4.2 | No evidence to suggest that measurement or ascertainment of the outcome differed between intervention groups. |
| 4.3a | NI |
| Note for 4.3a | No information provided on whether participants knew that they were in a trial |
| 4.3b | PY |
| Note for 4.3b | Participants (outcome assessors) were most likely aware of the group allocation due to the nature of the intervention. Research assistants that were involved in data collection were blind to allocation. |
| 4.4 | PY |
| 4.5 | PN |
| Note for 4.4&4.5 | Knowledge of the assigned intervention could influence participant-reported outcomes, but there is no reason to believe that it did. |
| 4.0 Assessor's Judgement | Some concerns |
| 4.0 General note | There is no evidence to suggest that the outcome measure was inappropriate and that measurement could have differed between groups. No information is provided on whether participants were aware that a trial was taking place, but it is likely that they were aware of their assigned intervention. There is no reason to believe that this knowledge would have influenced outcome assessment. |
| **Domain 5:**  **Risk of bias in selection of the reported result** |  |
| 5.1 | PY |
| Note for 5.1 | No evidence to suggest otherwise, protocol and paper do not show discrepancies. |
| 5.2 | PN |
| Note for 5.2 | No evidence to suggest outcome selection. All outcomes mentioned in the protocol, methods and results were reported. |
| 5.3 | PN |
| Note for 5.3 | No evidence to suggest analyses selection. All analyses mentioned in the methods and results were reported. |
| 5.0 Assessor's Judgement | Low |
| 5.0 General note | No evidence to suggest analysis or outcome selection. All outcomes and analyses mentioned in the methods and results were reported. |
| **Overall Risk of Bias** |  |
| Assessor's overall Judgement | Some concerns |

| **Study ID** | **Baker Henningham 2019** |
| --- | --- |
| *Experimental* | Violence prevention |
| *Comparator* | usual care |
| Outcome | Depression diagnosis |
| **Domain 1a:**  **Risk of bias arising from the randomization process** |  |
| 1a.1 | PY |
| 1a.2 | Y |
| Note for 1a.1&1a.2 | Quote: "Schools were randomised to intervention or control group in the summer preceding the intervention. Randomisation was conducted by an independent statistician who was blind. Schools were randomised to intervention or control group in the summer preceding the schools." |
| 1a.3 | NI |
| Note for 1a.3 | No useful information is reported to evaluate this element |
| 1a.0 Assessor's Judgement | Low |
| **Domain 1b: Risk of bias arising from the timing of identification or recruitment of participants** |  |
| 1b.1 | PY |
| Note for 1b.1 | Schools and teachers were identified and rectruited before the randomization of clusters, children from the teacher's classes were randomly selected at a later stage (outcome of interest refers to teachers |
| 1b.2 | NA |
| 1b.3 | NA |
| 1b.0 Assessor's Judgement | Low |
| **Domain 2: Risk of bias due to deviations from the intended interventions** |  |
| 2.1a | NI |
| Note for 2.1a | No information provided on whether participants knew that they were in a trial |
| 2.1b (2.1) | PY |
| 2.2 | PY |
| Note for 2.1b&2.2 | Due to the nature of the intervention, participants and personnel were most likely aware of the treatment allocation. |
| 2.3 | PN |
| Note for 2.3 | No evidence to suggest deviations from the intended intervention that arose because of the trial context |
| 2.4 | NA |
| 2.5 | NA |
| 2.6 | Y |
| Note for 2.6 | Data were analysed on an intention-to-treat basis. |
| 2.7 | NA |
| 2.0 Assessor's Judgement | Low |
| 2.0 General Notes | Even if participants were most likely aware of allocation there was no evidence to suggest deviations from the trial context. An appropriate analysis to estimate the effect of assignment was conducted. |
| **Domain 3: Missing outcome data** |  |
| 3.1a | PY |
| Note for 3.1a | all 14 clusters, schools, that recruited participants were analyzed |
| 3.1b | Y |
| Note for 3.1b | there was 1 loss among teachers (out of 54), no reported loss among children |
| 3.2 | NA |
| 3.3 | NA |
| 3.4 | NA |
| 3.0 Assessor's judgement | Low |
| 3.0 General notes | Data was available for almost all participants, all 14 clusters were analysed. |
| **Domain 4: Risk of bias in measurement of the outcome** |  |
| 4.1 | PN |
| Note for 4.1 | The outcome measure (CES-D) is widely established and used across contexts. |
| 4.2 | PN |
| Note for 4.2 | No evidence to suggest that measurement or ascertainment of the outcome differed between intervention groups. |
| 4.3a | NI |
| Note for 4.3a | No information provided on whether participants knew that they were in a trial |
| 4.3b | PY |
| Note for 4.3b | Participants (outcome assessors) were most likely aware of the group allocation due to the nature of the intervention. Research assistants that were involved in data collection were blind to allocation. |
| 4.4 | PY |
| 4.5 | PN |
| Note for 4.4&4.5 | Knowledge of the assigned intervention could influence participant-reported outcomes, but there is no reason to believe that it did. |
| 4.0 Assessor's Judgement | Some concerns |
| 4.0 General note | There is no evidence to suggest that the outcome measure was inappropriate and that measurement could have differed between groups. No information is provided on whether participants were aware that a trial was taking place, but it is likely that they were aware of their assigned intervention. There is no reason to believe that this knowledge would have influenced outcome assessment. |
| **Domain 5:**  **Risk of bias in selection of the reported result** |  |
| 5.1 | PY |
| Note for 5.1 | No evidence to suggest otherwise, protocol and paper do not show discrepancies. |
| 5.2 | PN |
| Note for 5.2 | No evidence to suggest outcome selection. All outcomes mentioned in the protocol, methods and results were reported. |
| 5.3 | PN |
| Note for 5.3 | No evidence to suggest analyses selection. All analyses mentioned in the methods and results were reported. |
| 5.0 Assessor's Judgement | Low |
| 5.0 General note | No evidence to suggest analysis or outcome selection. All outcomes and analyses mentioned in the methods and results were reported. |
| **Overall Risk of Bias** |  |
| Assessor's overall Judgement | Some concerns |

| **Study ID** | **Fabbri 2021** |
| --- | --- |
| *Experimental* | The EmpaTeach intervention |
| *Comparator* |  |
| Outcome | Depressive symptoms |
| **Domain 1a:**  **Risk of bias arising from the randomization process** |  |
| 1a.1 | Y |
| 1a.2 | Y |
| Note for 1a.1&1a.2 | 1a.1 / 1a.2 Quote: “To ensure balance across arms, schools were stratified according to whether they served a Congolese or Burundian population, and were primary or secondary schools. An allocation list was generated by EA with a computer random number generator and an algorithm in Stata (version 15). Allocation took place at a public meeting where a representative of each school within each stratum was invited to place the name of their school in an opaque bag. A nominated person from each stratum then withdrew names from the bag, and schools were allocated either to receive the intervention or to the control condition in the sequence on the allocation list, recorded by MZ.” |
| 1a.3 | NI |
| Note for 1a.3 | No useful information is reported to evaluate this element |
| 1a.0 Assessor's Judgement | Low |
| **Domain 1b: Risk of bias arising from the timing of identification or recruitment of participants** |  |
| 1b.1 | PY |
| Note for 1b.1 | Flow-chart and descriptive text indicate that schools and the student population were identified, inidividual students were included at baseline and then schools were randomized to each intervention group |
| 1b.2 | NA |
| 1b.3 | NA |
| 1b.0 Assessor's Judgement | Low |
| **Domain 2: Risk of bias due to deviations from the intended interventions** |  |
| 2.1a | NI |
| Note for 2.1a | No information provided on whether participants knew that they were in a trial |
| 2.1b (2.1) | PY |
| 2.2 | PY |
| Note for 2.1b&2.2 | No information provided on whether participants knew that they were in a trial, due to the nature of the intervention they (as those delivering the intervention) were most likely aware of intervention allocation. Quote: "The intervention is behavioural in nature, and it was not possible to mask participants to allocation." |
| 2.3 | PY |
| Note for 2.3 | No evidence to suggest deviations from the intended intervention that arose because of the trial context |
| 2.4 | PN |
| Note for 2.4 | Quote: "Our adherence analyses did not detect any differential effects of the compressed versus original format intervention, but we would have been underpowered to detect a difference, as it was necessary to conduct analyses at the school level." |
| 2.5 | NA |
| 2.6 | Y |
| Note for 2.6 | Data were analysed on an intention-to-treat basis. |
| 2.7 | NA |
| 2.0 Assessor's Judgement | Some concerns |
| 2.0 General Notes | Participants were most likely aware of allocation and there was evidence to suggest deviations from the trial context, but no strong evidence to suggest that it did. An appropriate analysis to estimate the effect of assignment was conducted. |
| **Domain 3: Missing outcome data** |  |
| 3.1a | PY |
| Note for 3.1a | all included clusters were analyzed |
| 3.1b | PY |
| Note for 3.1b | data were available for nearly all participants within the cluster |
| 3.2 | NA |
| 3.3 | NA |
| 3.4 | NA |
| 3.0 Assessor's judgement | Low |
| 3.0 General notes | Data was available for almost all participants, all clusters and participants seemed to have been analysed. |
| **Domain 4: Risk of bias in measurement of the outcome** |  |
| 4.1 | PN |
| Note for 4.1 | The outcome measure (MFQ) presented good psychometric properties in the study sample |
| 4.2 | PN |
| Note for 4.2 | No evidence to suggest that measurement or ascertainment of the outcome differed between intervention groups. |
| 4.3a | NI |
| Note for 4.3a | No information provided on whether participants knew that they were in a trial |
| 4.3b | PY |
| Note for 4.3b | Participants (outcome assessors) were most likely aware of the group allocation due to the nature of the intervention. |
| 4.4 | PY |
| 4.5 | PN |
| Note for 4.4&4.5 | Knowledge of the assigned intervention could influence participant-reported outcomes, but there is no reason to believe that it did. |
| 4.0 Assessor's Judgement | Some concerns |
| 4.0 General note | There is no evidence to suggest that the outcome measure was inappropriate and that measurement could have differed between groups. No information is provided on whether participants were aware that a trial was taking place, but it is likely that they were aware of their assigned intervention. There is no reason to believe that this knowledge would have influenced outcome assessment. |
| **Domain 5:**  **Risk of bias in selection of the reported result** |  |
| 5.1 | PY |
| Note for 5.1 | No evidence to suggest otherwise, methods and results do not show discrepancies. |
| 5.2 | PN |
| Note for 5.2 | While some chages had been made to the protocol, these were explicitely reported and accounted for in the manuscript. Quote: "Minor pragmatic changes to the trial protocol were made. We had originally intended to link data on educational outcomes for students from school records to our survey data, to explore the effects of the intervention on school achievement as a secondary outcome; however, it became clear that record linkage would be too time-consuming, and we instead analysed school attendance as a secondary outcome." |
| 5.3 | PN |
| Note for 5.3 | No evidence to suggest analyses selection. All analyses mentioned in the methods and results were reported. |
| 5.0 Assessor's Judgement | Low |
| 5.0 General note | No evidence to suggest analysis or outcome selection. All outcomes and analyses mentioned in the methods and results were reported. |
| **Overall Risk of Bias** |  |
| Assessor's overall Judgement | Some concerns |

| **Study ID** | **Shinde 2018** |
| --- | --- |
| *Experimental* | SEHER intervention (individual activities) |
| *Comparator* | AEP + SHER TSM, AEP |
| Outcome | Depressive symptoms |
| **Domain 1a:**  **Risk of bias arising from the randomization process** |  |
| 1a.1 | Y |
| 1a.2 | Y |
| Note for 1a.1&1a.2 | Randomization was conducted by an independent statistician, using minimisation in a 1:1: ratio |
| 1a.3 | PY |
| Note for 1a.3 | Stratificaton on school type was used, school baseline characteristics were comparable |
| 1a.0 Assessor's Judgement | Low |
| **Domain 1b: Risk of bias arising from the timing of identification or recruitment of participants** |  |
| 1b.1 | Y |
| Note for 1b.1 | individual participants were not recruited at all, all 9th grade students of the schools who gae their consent received the intervention and were enrolled. Schools were first identified and then randomized. |
| 1b.2 | NA |
| 1b.3 | NA |
| 1b.0 Assessor's Judgement | Low |
| **Domain 2: Risk of bias due to deviations from the intended interventions** |  |
| 2.1a | NI |
| Note for 2.1a | No information provided on whether participants knew that they were in a trial |
| 2.1b (2.1) | PY |
| 2.2 | PY |
| Note for 2.1b&2.2 | No information provided on whether participants knew that they were in a trial, due to the nature of the intervention they (as those delivering the intervention) were most likely aware of intervention allocation. |
| 2.3 | PN |
| Note for 2.3 | No evidence to suggest deviations from the intended intervention that arose because of the trial context |
| 2.4 | NA |
| Note for 2.4 |  |
| 2.5 | NA |
| 2.6 | Y |
| Note for 2.6 | Data was analysed on an intention-to-treat basis. |
| 2.7 | NA |
| 2.0 Assessor's Judgement | Low |
| 2.0 General Notes | Even if participants were most likely aware of allocation there was no evidence to suggest deviations from the trial context. An appropriate analysis to estimate the effect of assignment was conducted. |
| **Domain 3: Missing outcome data** |  |
| 3.1a | PY |
| Note for 3.1a | 1 out of the 75 clusters dropped out after randomization and did not receive the intervention, |
| 3.1b | PN |
| Note for 3.1b | 60.8-70.8% of elegible participants completed the endpoint assesment |
| 3.2 | PN |
| Note for 3.2 | No evidence that the result was not biased by missing data |
| 3.3 | PY |
| 3.4 | PN |
| Note for 3.3&3.4 | Reasonons for missingness reported indicate that it could depend on its true value, but this is unlikely to influence the trial results given that absence rates from the study are comparable to those observed during the regular school year. |
| 3.0 Assessor's judgement | Some concerns |
| 3.0 General notes | All but 1 clusters were analysed. Data was not avaiable for all randomized participants but it is unlikely that missingness could be dependant on the outcome's true value |
| **Domain 4: Risk of bias in measurement of the outcome** |  |
| 4.1 | PN |
| Note for 4.1 | The outcome measure is widely established and validated for use across different contexts, included the one of the trial |
| 4.2 | PN |
| Note for 4.2 | No evidence to suggest that. |
| 4.3a | NI |
| Note for 4.3a | No information provided on whether participants knew that they were in a trial |
| 4.3b | PY |
| Note for 4.3b | Participants were most likely aware of the group allocation due to the nature of the intervention |
| 4.4 | PY |
| Note for 4.4&4.5 | Knowledge of the assigned intervention could influence participant-reported outcomes, but there is no reason to believe that it did. |
| 4.0 Assessor's Judgement | Some concerns |
| 4.0 General note | There is no evidence to suggest that the outcome measure was inappropriate and that measurement could have differed between groups. No information is provided on whether participants were aware that a trial was taking place, but it is likely that they were aware of their assigned intervention. There is no reason to believe that this knowledge would have influenced outcome assessment. |
| **Domain 5:**  **Risk of bias in selection of the reported result** |  |
| 5.1 | PY |
| Note for 5.1 | No evidence to suggest otherwise, protocol and final article not show discrepancies. |
| 5.2 | PN |
| Note for 5.2 | No evidence to suggest outcome selection. Further outcomes than those mentioned in the protocol were reported. |
| 5.3 | PN |
| Note for 5.3 | No evidence to suggest analyses selection. All analyses mentioned in the methods and results were reported. |
| 5.0 Assessor's Judgement | Low |
| 5.0 General note | No evidence to suggest analysis or outcome selection. All outcomes and analyses mentioned in the methods and results were reported. |
| **Overall Risk of Bias** |  |
| Assessor's overall Judgement | Some concerns |

| **Study ID** | **Hirani 2010** |
| --- | --- |
| *Experimental* | ESB intervention; Group counselling intervention |
| *Comparator* | Usual care |
| Outcome | Depressive symptoms |
| **Domain 1a:**  **Risk of bias arising from the randomization process** |  |
| 1a.1 | PY |
| 1a.2 | NI |
| Note for 1a.1&1a.2 | Quote: "Stratified block randomizationwas conducted to distribute schools by location and girl enrollment across Girls First e Bihar conditions (19 schools/condition)." |
| 1a.3 | NI |
| Note for 1a.3 | No useful information is reported to evaluate this element |
| 1a.0 Assessor's Judgement | Some concerns |
| **Domain 1b: Risk of bias arising from the timing of identification or recruitment of participants** |  |
| 1b.1 | NI |
| Note for 1b.1 | no information on the sequence of randomization and recruitment is provided |
| 1b.2 | PN |
| Note for 1b.2 | no evidence to suggest that selection was influenced by knowledge of allocation |
| 1b.3 | PN |
| Note for 1b.3 | Quote: "no significant differences existed in demographic characteristics between the groups". |
| 1b.0 Assessor's Judgement | Some concerns |
| **Domain 2: Risk of bias due to deviations from the intended interventions** |  |
| 2.1a | NI |
| Note for 2.1a | No information provided on whether participants knew that they were in a trial |
| 2.1b (2.1) | PY |
| 2.2 | PY |
| Note for 2.1b&2.2 | No information provided on whether participants knew that they were in a trial, due to the nature of the intervention they (as those delivering the intervention) were most likely aware of intervention allocation. |
| 2.3 | PN |
| Note for 2.3 | No evidence to suggest deviations from the intended intervention that arose because of the trial context |
| 2.4 | NA |
| 2.5 | NA |
| 2.6 | NI |
| Note for 2.6 | Not enough information provided to give a judjment on this item |
| 2.7 | PN |
| Note for 2.7 | No evidence to suggest that failure to analyzed participants in randomized groups could a substantial impact on the results |
| 2.0 Assessor's Judgement | Some concerns |
| 2.0 General Notes | Participants were most likely aware of allocation, there was no evidence to suggest deviations from the trial context. No information was provided to conclude on whether the analysis to estimate the effect of assignment was appropriate. |
| **Domain 3: Missing outcome data** |  |
| 3.1a | PY |
| Note for 3.1a | no evidence to suggest cluster loss |
| 3.1b | PY |
| Note for 3.1b | it appears that data were avaiable for almost all randomized participants , Quote: "Twenty four women completed signed informed consent and completed outcome measures" |
| 3.2 | NA |
| 3.3 | NA |
| 3.4 | NA |
| 3.0 Assessor's judgement | Low |
| 3.0 General notes | Data was available for almost all participants, all clusters and participants seemed to have been analysed. |
| **Domain 4: Risk of bias in measurement of the outcome** |  |
| 4.1 | PN |
| Note for 4.1 | The outcome measure (BDI) is widely established and used across contexts. |
| 4.2 | PN |
| Note for 4.2 | No evidence to suggest that measurement or ascertainment of the outcome differed between intervention groups. |
| 4.3a | NI |
| Note for 4.3a | No information provided on whether participants knew that they were in a trial |
| 4.3b | PY |
| Note for 4.3b | participants (outcome assessors) were most likely aware of the group allocation due to the nature of the intervention. |
| 4.4 | PY |
| 4.5 | PN |
| Note for 4.4&4.5 | Knowledge of the assigned intervention could influence participant-reported outcomes, but there is no reason to believe that it did. |
| 4.0 Assessor's Judgement | Some concerns |
| 4.0 General note | There is no evidence to suggest that the outcome measure was inappropriate and that measurement could have differed between groups. No information is provided on whether participants were aware that a trial was taking place, but it is likely that they were aware of their assigned intervention. There is no reason to believe that this knowledge would have influenced outcome assessment. |
| **Domain 5:**  **Risk of bias in selection of the reported result** |  |
| 5.1 | PY |
| Note for 5.1 | No evidence to suggest otherwise, protocol and final article not show discrepancies. |
| 5.2 | PN |
| Note for 5.2 | No evidence to suggest outcome selection. Further outcomes than those mentioned in the protocol were reported. |
| 5.3 | PN |
| Note for 5.3 | No evidence to suggest analyses selection. All analyses mentioned in the methods and results were reported. |
| 5.0 Assessor's Judgement | Low |
| 5.0 General note | No evidence to suggest analysis or outcome selection. All outcomes and analyses mentioned in the methods and results were reported. |
| **Overall Risk of Bias** |  |
| Assessor's overall Judgement | Some concerns |

| **Study ID** | **Hirani 2010** |
| --- | --- |
| *Experimental* | ESB intervention; Group counselling intervention |
| *Comparator* | usual care |
| Outcome | Depressive diagnosis |
| **Domain 1a:**  **Risk of bias arising from the randomization process** |  |
| 1a.1 | PY |
| 1a.2 | NI |
| Note for 1a.1&1a.2 | Quote: "Stratified block randomizationwas conducted to distribute schools by location and girl enrollment across Girls First e Bihar conditions (19 schools/condition)." |
| 1a.3 | NI |
| Note for 1a.3 | No useful information is reported to evaluate this element |
| 1a.0 Assessor's Judgement | Some concerns |
| **Domain 1b: Risk of bias arising from the timing of identification or recruitment of participants** |  |
| 1b.1 | NI |
| Note for 1b.1 | no information on the sequence of randomization and recruitment is provided |
| 1b.2 | PN |
| Note for 1b.2 | no evidence to suggest that selection was influenced by knowledge of allocation |
| 1b.3 | PN |
| Note for 1b.3 | Quote: "no significant differences existed in demographic characteristics between the groups". |
| 1b.0 Assessor's Judgement | Some concerns |
| **Domain 2: Risk of bias due to deviations from the intended interventions** |  |
| 2.1a | NI |
| Note for 2.1a | No information provided on whether participants knew that they were in a trial |
| 2.1b (2.1) | PY |
| 2.2 | PY |
| Note for 2.1b&2.2 | No information provided on whether participants knew that they were in a trial, due to the nature of the intervention they (as those delivering the intervention) were most likely aware of intervention allocation. |
| 2.3 | PN |
| Note for 2.3 | No evidence to suggest deviations from the intended intervention that arose because of the trial context |
| 2.4 | NA |
| 2.5 | NA |
| 2.6 | NI |
| Note for 2.6 | Not enough information provided to give a judjment on this item |
| 2.7 | PN |
| Note for 2.7 | No evidence to suggest that failure to analyzed participants in randomized groups could a substantial impact on the results |
| 2.0 Assessor's Judgement | Some concerns |
| 2.0 General Notes | Participants were most likely aware of allocation, there was no evidence to suggest deviations from the trial context. No information was provided to conclude on whether the analysis to estimate the effect of assignment was appropriate. |
| **Domain 3: Missing outcome data** |  |
| 3.1a | PY |
| Note for 3.1a | no evidence to suggest cluster loss |
| 3.1b | PY |
| Note for 3.1b | it appears that data were avaiable for almost all randomized participants , Quote: "Twenty four women completed signed informed consent and completed outcome measures" |
| 3.2 | NA |
| 3.3 | NA |
| 3.4 | NA |
| 3.0 Assessor's judgement | Low |
| 3.0 General notes | Data was available for almost all participants, all clusters and participants seemed to have been analysed. |
| **Domain 4: Risk of bias in measurement of the outcome** |  |
| 4.1 | PN |
| Note for 4.1 | The outcome measure (BDI) is widely established and used across contexts. |
| 4.2 | PN |
| Note for 4.2 | No evidence to suggest that measurement or ascertainment of the outcome differed between intervention groups. |
| 4.3a | NI |
| Note for 4.3a | No information provided on whether participants knew that they were in a trial |
| 4.3b | PY |
| Note for 4.3b | participants (outcome assessors) were most likely aware of the group allocation due to the nature of the intervention. |
| 4.4 | PY |
| 4.5 | PN |
| Note for 4.4&4.5 | Knowledge of the assigned intervention could influence participant-reported outcomes, but there is no reason to believe that it did. |
| 4.0 Assessor's Judgement | Some concerns |
| 4.0 General note | There is no evidence to suggest that the outcome measure was inappropriate and that measurement could have differed between groups. No information is provided on whether participants were aware that a trial was taking place, but it is likely that they were aware of their assigned intervention. There is no reason to believe that this knowledge would have influenced outcome assessment. |
| **Domain 5:**  **Risk of bias in selection of the reported result** |  |
| 5.1 | PY |
| Note for 5.1 | No evidence to suggest otherwise, protocol and final article not show discrepancies. |
| 5.2 | PN |
| Note for 5.2 | No evidence to suggest outcome selection. Further outcomes than those mentioned in the protocol were reported. |
| 5.3 | PN |
| Note for 5.3 | No evidence to suggest analyses selection. All analyses mentioned in the methods and results were reported. |
| 5.0 Assessor's Judgement | Low |
| 5.0 General note | No evidence to suggest analysis or outcome selection. All outcomes and analyses mentioned in the methods and results were reported. |
| **Overall Risk of Bias** |  |
| Assessor's overall Judgement | Some concerns |

| **Study ID** | **Lachman 2020** |
| --- | --- |
| *Experimental* | Parenting programme & agribusiness training |
| *Comparator* | Usual care |
| Outcome | Depressive symptoms |
| **Domain 1a:**  **Risk of bias arising from the randomization process** |  |
| 1a.1 | Y |
| 1a.2 | Y |
| Note for 1a.1&1a.2 | Quote: "Cluster randomisation was conducted at village level prior to baseline assessments, to reduce the likelihood of contamination between arms. An external researcher used concealed computer-generated codes to randomly allocate eight villages into three treatment arms and a control arm "; "The implementing partner notified the participating families of their allocation status after baseline data collection." |
| 1a.3 | NI |
| Note for 1a.3 | No useful information is reported to evaluate this element |
| 1a.0 Assessor's Judgement | Low |
| **Domain 1b: Risk of bias arising from the timing of identification or recruitment of participants** |  |
| 1b.1 | PN |
| Note for 1b.1 | randomization was conducted prior to the recruitment of participants |
| 1b.2 | PN |
| Note for 1b.2 | groups were comparable at baseline, see Table 1 |
| 1b.0 Assessor's Judgement | Low |
| **Domain 2: Risk of bias due to deviations from the intended interventions** |  |
| 2.1a | NI |
| Note for 2.1a | No information provided on whether participants knew that they were in a trial |
| 2.1b (2.1) | PY |
| 2.2 | PY |
| Note for 2.1b&2.2 | No information provided on whether participants knew that they were in a trial, due to the nature of the intervention they (as those delivering the intervention) were most likely aware of intervention allocation. |
| 2.3 | PN |
| Note for 2.3 | No evidence to suggest deviations from the protocol |
| 2.4 | NA |
| 2.5 | NA |
| 2.6 | Y |
| Note for 2.6 | Data was analysed on an intention-to-treat basis. |
| 2.7 | NA |
| 2.0 Assessor's Judgement | Low |
| 2.0 General Notes | Even if participants were most likely aware of allocation there was no evidence to suggest deviations from the trial context. Particiapnts were analyzed on an intention to treat basis. |
| **Domain 3: Missing outcome data** |  |
| 3.1a | PY |
| Note for 3.1a | all included clusters were analyzed |
| 3.1b | PY |
| Note for 3.1b | data were available for nearly all participants within the cluster.Quote:"Study retention was considerably higher than anticipated with 94.8% adults (n=235/248), 87.5% children (n=154/176) and 87.8% in their early childhood (n=122/139) assessments completed at post-treatment, with no differences between arms " |
| 3.2 | NA |
| 3.3 | NA |
| 3.4 | NA |
| 3.0 Assessor's judgement | Low |
| 3.0 General notes | Data was available for almost all participants, all clusters and participants have been analysed. |
| **Domain 4: Risk of bias in measurement of the outcome** |  |
| 4.1 | PN |
| Note for 4.1 | The outcome measure is widely established and validated for use across different contexts |
| 4.2 | PN |
| Note for 4.2 | No evidence to suggest differences in measurement between intervention and control. |
| 4.3a | NI |
| Note for 4.3a | No information provided on whether participants knew that they were in a trial |
| 4.3b | PY |
| Note for 4.3b | No information provided on whether participants knew that they were in a trial, participants (outcome assessors) were most likely aware of the group allocation due to the nature of the intervention. |
| 4.4 | PY |
| 4.5 | PN |
| Note for 4.4&4.5 | Knowledge of the assigned intervention could influence participant-reported outcomes, but there is no reason to believe that it did. |
| 4.0 Assessor's Judgement | Some concerns |
| 4.0 General note | There is no evidence to suggest that the outcome measure was inappropriate and that measurement could have differed between groups. No information is provided on whether participants were aware that a trial was taking place, but it is likely that they were aware of their assigned intervention. There is no reason to believe that this knowledge would have influenced outcome assessment. |
| **Domain 5:**  **Risk of bias in selection of the reported result** |  |
| 5.1 | PY |
| Note for 5.1 | No evidence to suggest otherwise, protocol and final article not show discrepancies. |
| 5.2 | PN |
| Note for 5.2 | No evidence to suggest outcome selection. Further outcomes than those mentioned in the protocol were reported. |
| 5.3 | PN |
| Note for 5.3 | No evidence to suggest analyses selection. All analyses mentioned in the methods and results were reported. |
| 5.0 Assessor's Judgement | Low |
| 5.0 General note | No evidence to suggest analysis or outcome selection. All outcomes and analyses mentioned in the methods and results were reported. |
| **Overall Risk of Bias** |  |
| Assessor's overall Judgement | Some concerns |

| **Study ID** | **Greene 2023** |
| --- | --- |
| *Experimental* | Entre Nosotras |
| *Comparator* | Usual care |
| Outcome | Distress |
| **Domain 1a:**  **Risk of bias arising from the randomization process** |  |
| 1a.1 | Y |
| 1a.2 | PY |
| Note for 1a.1&1a.2 | Quote: "Communities (clusters) were randomly allocated to study conditions using a random number generator in Stata by a researcher not affiliated with the project. Two pairs of communities adjacent to each other with overlapping catchment areas were combined into two clusters (versus four independent community clusters), thus leading to nine randomized community clusters." |
| 1a.3 | PN |
| Note for 1a.3 | Control and intervention groups were overall comparable at baseline (Table 1) |
| 1a.0 Assessor's Judgement | Low |
| **Domain 1b: Risk of bias arising from the timing of identification or recruitment of participants** |  |
| 1b.1 | NI |
| Note for 1b.1 | No information provided about the recruitment procedure at individual level. The authors only said that participants were recruited within communities. |
| 1b.2 | PN |
| Note for 1b.2 | There is no reason to believe it |
| 1b.0 Assessor's Judgement | Low |
| **Domain 2: Risk of bias due to deviations from the intended interventions** |  |
| 2.1a | Y |
| Note for 2.1a | Quote: "It was not possible to blind participants, intervention providers or outcome assessors to study conditions." |
| 2.1b (2.1) | Y |
| 2.2 | Y |
| Note for 2.1b&2.2 | Quote: "It was not possible to blind par- ticipants, intervention providers or outcome assessors to study conditions." |
| 2.3 | PN |
| Note for 2.3 | No evidence to suggest deviations arising from the trial context. Quote: ". The study had a slower recruitment rate and higher levels of attrition than were expected with variation observed across com- munities and over time (i.e., different stages of the COVID-19 pandemic and rapidly changing contexts). There were no other major protocol deviations or any serious adverse events detected during the study and no major baseline imbalances by study condition despite the diverse communities included in the study." |
| 2.4 | NA |
| 2.5 | NA |
| 2.6 | Y |
| Note for 2.6 | ITT analyses were conducted |
| 2.7 | NA |
| 2.0 Assessor's Judgement | Low |
| 2.0 General Notes |  |
| **Domain 3: Missing outcome data** |  |
| 3.1a | PY |
| Note for 3.1a | It appears that data is available for all 9 clusters |
| 3.1b | PY |
| Note for 3.1b | Attrition rate was no more than 20% |
| 3.2 | NA |
| 3.3 | NA |
| 3.4 | NA |
| 3.0 Assessor's judgement | Low |
| 3.0 General notes |  |
| **Domain 4: Risk of bias in measurement of the outcome** |  |
| 4.1 | N |
| Note for 4.1 | The selected outcome method is validated for use in the local context. |
| 4.2 | PN |
| Note for 4.2 | No evidence to suggest differences in measurement between intervention and control |
| 4.3a | Y |
| Note for 4.3a | Quote: "It was not possible to blind par- ticipants, intervention providers or outcome assessors to study conditions." |
| 4.3b | Y |
| Note for 4.3b | Quote: "It was not possible to blind par- ticipants, intervention providers or outcome assessors to study conditions." |
| 4.4 | PY |
| 4.5 | PN |
| Note for 4.4&4.5 | Assessment could have been influenced by knowledge of the intervention but it is not likely that it did |
| 4.0 Assessor's Judgement | Some concerns |
| 4.0 General note |  |
| **Domain 5:**  **Risk of bias in selection of the reported result** |  |
| 5.1 | Y |
| Note for 5.1 | Protocol was available |
| 5.2 | PN |
| Note for 5.2 | No evidence to suggest outcomes selection. All outcomes mentioned in the methods and protocol were reported. |
| 5.3 | PN |
| Note for 5.3 | No evidence to suggest analyses selection. All analyses mentioned in the methods and results were reported. |
| 5.0 Assessor's Judgement | Low |
| 5.0 General note |  |
| **Overall Risk of Bias** |  |
| Assessor's overall Judgement | Some concerns |

| **Study ID** | **Massarwi 2022** |
| --- | --- |
| *Experimental* | Parenting program |
| *Comparator* | Usual care |
| Outcome | Distress |
| **Domain 1a:**  **Risk of bias arising from the randomization process** |  |
| 1a.1 | Y |
| 1a.2 | Y |
| Note for 1a.1&1a.2 | Quote: "Randomization was stratified by rural/urban location and con- ducted after baseline by using random numbers generated by an inde- pendent, blinded statistician (CL). Complete randomization within strata used a 1:1 intervention to control ratio. Allocation to interven- tion and control groups was conducted randomly." |
| 1a.3 | PN |
| Note for 1a.3 | Control and intervention groups were overall comparable at baseline (Table 1) |
| 1a.0 Assessor's Judgement | Low |
| **Domain 1b: Risk of bias arising from the timing of identification or recruitment of participants** |  |
| 1b.1 | NI |
| Note for 1b.1 | No information provided about the recruitment procedure at individual level. The authors only said that primary caregivers of adolescents (aged 10–18) were recruited from rural and peri-urban settlements in the Eastern Cape province of South Africa |
| 1b.2 | PN |
| Note for 1b.2 | There is no reason to believe it |
| 1b.0 Assessor's Judgement | Low |
| **Domain 2: Risk of bias due to deviations from the intended interventions** |  |
| 2.1a | Y |
| Note for 2.1a | Quote: "Blinding of participants and programme providers was not feasible for the parenting programme." |
| 2.1b (2.1) | Y |
| 2.2 | Y |
| Note for 2.1b&2.2 | Quote: "Blinding of participants and programme providers was not feasible for the parenting programme." |
| 2.3 | PN |
| Note for 2.3 | No evidence to suggest deviations arising from the trial context. |
| 2.4 | NA |
| 2.5 | NA |
| 2.6 | Y |
| Note for 2.6 | ITT analyses were conducted |
| 2.7 | NA |
| 2.0 Assessor's Judgement | Low |
| 2.0 General Notes |  |
| **Domain 3: Missing outcome data** |  |
| 3.1a | PY |
| Note for 3.1a | It appears that data is available for all 49 clusters |
| 3.1b | Y |
| Note for 3.1b | No drop-out |
| 3.2 | NA |
| 3.3 | NA |
| 3.4 | NA |
| 3.0 Assessor's judgement | Low |
| 3.0 General notes |  |
| **Domain 4: Risk of bias in measurement of the outcome** |  |
| 4.1 | N |
| Note for 4.1 | The selected outcome method is validated for use in the local context. |
| 4.2 | PN |
| Note for 4.2 | No evidence to suggest differences in measurement between intervention and control |
| 4.3a | PY |
| Note for 4.3a | Participants (which were outcome assessors) were most likely aware of being in a trial |
| 4.3b | PY |
| Note for 4.3b | Participants (which were outcome assessors) were most likely aware of group allocation. |
| 4.4 | PY |
| 4.5 | PN |
| Note for 4.4&4.5 | Assessment could have been influenced by knowledge of the intervention but it is not likely that it did |
| 4.0 Assessor's Judgement | Some concerns |
| 4.0 General note |  |
| **Domain 5:**  **Risk of bias in selection of the reported result** |  |
| 5.1 | Y |
| Note for 5.1 | Protocol was available |
| 5.2 | PN |
| Note for 5.2 | No evidence to suggest outcomes selection. All outcomes mentioned in the methods and protocol were reported. |
| 5.3 | PN |
| Note for 5.3 | No evidence to suggest analyses selection. All analyses mentioned in the methods and results were reported. |
| 5.0 Assessor's Judgement | Low |
| 5.0 General note |  |
| **Overall Risk of Bias** |  |
| Assessor's overall Judgement | Some concerns |

| **Study ID** | **Massarwi 2022** |
| --- | --- |
| *Experimental* | Parenting program |
| *Comparator* | Usual care |
| Outcome | Depressive symptoms |
| **Domain 1a:**  **Risk of bias arising from the randomization process** |  |
| 1a.1 | Y |
| 1a.2 | Y |
| Note for 1a.1&1a.2 | Quote: "Randomization was stratified by rural/urban location and con- ducted after baseline by using random numbers generated by an inde- pendent, blinded statistician (CL). Complete randomization within strata used a 1:1 intervention to control ratio. Allocation to interven- tion and control groups was conducted randomly." |
| 1a.3 | PN |
| Note for 1a.3 | Control and intervention groups were overall comparable at baseline (Table 1) |
| 1a.0 Assessor's Judgement | Low |
| **Domain 1b: Risk of bias arising from the timing of identification or recruitment of participants** |  |
| 1b.1 | NI |
| Note for 1b.1 | No information provided about the recruitment procedure at individual level. The authors only said that primary caregivers of adolescents (aged 10–18) were recruited from rural and peri-urban settlements in the Eastern Cape province of South Africa |
| 1b.2 | PN |
| Note for 1b.2 | There is no reason to believe it |
| 1b.0 Assessor's Judgement | Low |
| **Domain 2: Risk of bias due to deviations from the intended interventions** |  |
| 2.1a | Y |
| Note for 2.1a | Quote: "Blinding of participants and programme providers was not feasible for the parenting programme." |
| 2.1b (2.1) | Y |
| 2.2 | Y |
| Note for 2.1b&2.2 | Quote: "Blinding of participants and programme providers was not feasible for the parenting programme." |
| 2.3 | PN |
| Note for 2.3 | No evidence to suggest deviations arising from the trial context. |
| 2.4 | NA |
| 2.5 | NA |
| 2.6 | Y |
| Note for 2.6 | ITT analyses were conducted |
| 2.7 | NA |
| 2.0 Assessor's Judgement | Low |
| 2.0 General Notes |  |
| **Domain 3: Missing outcome data** |  |
| 3.1a | PY |
| Note for 3.1a | It appears that data is available for all 49 clusters |
| 3.1b | Y |
| Note for 3.1b | No drop-out |
| 3.2 | NA |
| 3.3 | NA |
| 3.4 | NA |
| 3.0 Assessor's judgement | Low |
| 3.0 General notes |  |
| **Domain 4: Risk of bias in measurement of the outcome** |  |
| 4.1 | N |
| Note for 4.1 | The selected outcome method is validated for use in the local context. |
| 4.2 | PN |
| Note for 4.2 | No evidence to suggest differences in measurement between intervention and control |
| 4.3a | PY |
| Note for 4.3a | Participants (which were outcome assessors) were most likely aware of being in a trial |
| 4.3b | PY |
| Note for 4.3b | Participants (which were outcome assessors) were most likely aware of group allocation. |
| 4.4 | PY |
| 4.5 | PN |
| Note for 4.4&4.5 | Assessment could have been influenced by knowledge of the intervention but it is not likely that it did |
| 4.0 Assessor's Judgement | Some concerns |
| 4.0 General note |  |
| **Domain 5:**  **Risk of bias in selection of the reported result** |  |
| 5.1 | Y |
| Note for 5.1 | Protocol was available |
| 5.2 | PN |
| Note for 5.2 | No evidence to suggest outcomes selection. All outcomes mentioned in the methods and protocol were reported. |
| 5.3 | PN |
| Note for 5.3 | No evidence to suggest analyses selection. All analyses mentioned in the methods and results were reported. |
| 5.0 Assessor's Judgement | Low |
| 5.0 General note |  |
| **Overall Risk of Bias** |  |
| Assessor's overall Judgement | Some concerns |

| **Study ID** | **Lachman 2020** |
| --- | --- |
| *Experimental* | parenting programme & agribusiness training |
| *Comparator* | usual care |
| Outcome | Depression diagnosis |
| **Domain 1a:**  **Risk of bias arising from the randomization process** |  |
| 1a.1 | Y |
| 1a.2 | Y |
| Note for 1a.1&1a.2 | Quote: "Cluster randomisation was conducted at village level prior to baseline assessments, to reduce the likelihood of contamination between arms. An external researcher used concealed computer-generated codes to randomly allocate eight villages into three treatment arms and a control arm "; "The implementing partner notified the participating families of their allocation status after baseline data collection." |
| 1a.3 | NI |
| Note for 1a.3 | No useful information is reported to evaluate this element |
| 1a.0 Assessor's Judgement | Low |
| **Domain 1b: Risk of bias arising from the timing of identification or recruitment of participants** |  |
| 1b.1 | PN |
| Note for 1b.1 | randomization was conducted prior to the recruitment of participants |
| 1b.2 | PN |
| Note for 1b.2 | groups were comparable at baseline, see Table 1 |
| 1b.0 Assessor's Judgement | Low |
| **Domain 2: Risk of bias due to deviations from the intended interventions** |  |
| 2.1a | NI |
| Note for 2.1a | No information provided on whether participants knew that they were in a trial |
| 2.1b (2.1) | PY |
| 2.2 | PY |
| Note for 2.1b&2.2 | No information provided on whether participants knew that they were in a trial, due to the nature of the intervention they (as those delivering the intervention) were most likely aware of intervention allocation. |
| 2.3 | PN |
| Note for 2.3 | No evidence to suggest deviations from the protocol |
| 2.4 | NA |
| 2.5 | NA |
| 2.6 | Y |
| Note for 2.6 | Data was analysed on an intention-to-treat basis. |
| 2.7 | NA |
| 2.0 Assessor's Judgement | Low |
| 2.0 General Notes | Even if participants were most likely aware of allocation there was no evidence to suggest deviations from the trial context. Particiapnts were analyzed on an intention to treat basis. |
| **Domain 3: Missing outcome data** |  |
| 3.1a | PY |
| Note for 3.1a | all included clusters were analyzed |
| 3.1b | PY |
| Note for 3.1b | data were available for nearly all participants within the cluster.Quote:"Study retention was considerably higher than anticipated with 94.8% adults (n=235/248), 87.5% children (n=154/176) and 87.8% in their early childhood (n=122/139) assessments completed at post-treatment, with no differences between arms " |
| 3.2 | NA |
| 3.3 | NA |
| 3.4 | NA |
| 3.0 Assessor's judgement | Low |
| 3.0 General notes | Data was available for almost all participants, all clusters and participants have been analysed. |
| **Domain 4: Risk of bias in measurement of the outcome** |  |
| 4.1 | PN |
| Note for 4.1 | The outcome measure is widely established and validated for use across different contexts |
| 4.2 | PN |
| Note for 4.2 | No evidence to suggest differences in measurement between intervention and control. |
| 4.3a | NI |
| Note for 4.3a | No information provided on whether participants knew that they were in a trial |
| 4.3b | PY |
| Note for 4.3b | No information provided on whether participants knew that they were in a trial, participants (outcome assessors) were most likely aware of the group allocation due to the nature of the intervention. |
| 4.4 | PY |
| 4.5 | PN |
| Note for 4.4&4.5 | Knowledge of the assigned intervention could influence participant-reported outcomes, but there is no reason to believe that it did. |
| 4.0 Assessor's Judgement | Some concerns |
| 4.0 General note | There is no evidence to suggest that the outcome measure was inappropriate and that measurement could have differed between groups. No information is provided on whether participants were aware that a trial was taking place, but it is likely that they were aware of their assigned intervention. There is no reason to believe that this knowledge would have influenced outcome assessment. |
| **Domain 5:**  **Risk of bias in selection of the reported result** |  |
| 5.1 | PY |
| Note for 5.1 | No evidence to suggest otherwise, protocol and final article not show discrepancies. |
| 5.2 | PN |
| Note for 5.2 | No evidence to suggest outcome selection. Further outcomes than those mentioned in the protocol were reported. |
| 5.3 | PN |
| Note for 5.3 | No evidence to suggest analyses selection. All analyses mentioned in the methods and results were reported. |
| 5.0 Assessor's Judgement | Low |
| 5.0 General note | No evidence to suggest analysis or outcome selection. All outcomes and analyses mentioned in the methods and results were reported. |
| **Overall Risk of Bias** |  |
| Assessor's overall Judgement | Some concerns |

| **Study ID** | **Lachman 2020** |
| --- | --- |
| *Experimental* | Parenting programme & agribusiness training |
| *Comparator* | Usual care |
| Outcome | PTSD symptoms |
| **Domain 1a:**  **Risk of bias arising from the randomization process** |  |
| 1a.1 | Y |
| 1a.2 | Y |
| Note for 1a.1&1a.2 | Quote: "Cluster randomisation was conducted at village level prior to baseline assessments, to reduce the likelihood of contamination between arms. An external researcher used concealed computer-generated codes to randomly allocate eight villages into three treatment arms and a control arm "; "The implementing partner notified the participating families of their allocation status after baseline data collection." |
| 1a.3 | NI |
| Note for 1a.3 | No useful information is reported to evaluate this element |
| 1a.0 Assessor's Judgement | Low |
| **Domain 1b: Risk of bias arising from the timing of identification or recruitment of participants** |  |
| 1b.1 | PN |
| Note for 1b.1 | randomization was conducted prior to the recruitment of participants |
| 1b.2 | PN |
| Note for 1b.2 | groups were comparable at baseline, see Table 1 |
| 1b.0 Assessor's Judgement | Low |
| **Domain 2: Risk of bias due to deviations from the intended interventions** |  |
| 2.1a | NI |
| Note for 2.1a | No information provided on whether participants knew that they were in a trial |
| 2.1b (2.1) | PY |
| 2.2 | PY |
| Note for 2.1b&2.2 | No information provided on whether participants knew that they were in a trial, due to the nature of the intervention they (as those delivering the intervention) were most likely aware of intervention allocation. |
| 2.3 | PN |
| Note for 2.3 | No evidence to suggest deviations from the protocol |
| 2.4 | NA |
| 2.5 | NA |
| 2.6 | Y |
| Note for 2.6 | Data was analysed on an intention-to-treat basis. |
| 2.7 | NA |
| 2.0 Assessor's Judgement | Low |
| 2.0 General Notes | Even if participants were most likely aware of allocation there was no evidence to suggest deviations from the trial context. Particiapnts were analyzed on an intention to treat basis. |
| **Domain 3: Missing outcome data** |  |
| 3.1a | PY |
| Note for 3.1a | all included clusters were analyzed |
| 3.1b | PY |
| Note for 3.1b | data were available for nearly all participants within the cluster.Quote:"Study retention was considerably higher than anticipated with 94.8% adults (n=235/248), 87.5% children (n=154/176) and 87.8% in their early childhood (n=122/139) assessments completed at post-treatment, with no differences between arms " |
| 3.2 | NA |
| 3.3 | NA |
| 3.4 | NA |
| 3.0 Assessor's judgement | Low |
| 3.0 General notes | Data was available for almost all participants, all clusters and participants have been analysed. |
| **Domain 4: Risk of bias in measurement of the outcome** |  |
| 4.1 | PN |
| Note for 4.1 | The outcome measure is widely established and validated for use across different contexts |
| 4.2 | PN |
| Note for 4.2 | No evidence to suggest differences in measurement between intervention and control. |
| 4.3a | NI |
| Note for 4.3a | No information provided on whether participants knew that they were in a trial |
| 4.3b | PY |
| Note for 4.3b | No information provided on whether participants knew that they were in a trial, participants (outcome assessors) were most likely aware of the group allocation due to the nature of the intervention. |
| 4.4 | PY |
| 4.5 | PN |
| Note for 4.4&4.5 | Knowledge of the assigned intervention could influence participant-reported outcomes, but there is no reason to believe that it did. |
| 4.0 Assessor's Judgement | Some concerns |
| 4.0 General note | There is no evidence to suggest that the outcome measure was inappropriate and that measurement could have differed between groups. No information is provided on whether participants were aware that a trial was taking place, but it is likely that they were aware of their assigned intervention. There is no reason to believe that this knowledge would have influenced outcome assessment. |
| **Domain 5:**  **Risk of bias in selection of the reported result** |  |
| 5.1 | PY |
| Note for 5.1 | No evidence to suggest otherwise, protocol and final article not show discrepancies. |
| 5.2 | PN |
| Note for 5.2 | No evidence to suggest outcome selection. Further outcomes than those mentioned in the protocol were reported. |
| 5.3 | PN |
| Note for 5.3 | No evidence to suggest analyses selection. All analyses mentioned in the methods and results were reported. |
| 5.0 Assessor's Judgement | Low |
| 5.0 General note | No evidence to suggest analysis or outcome selection. All outcomes and analyses mentioned in the methods and results were reported. |
| **Overall Risk of Bias** |  |
| Assessor's overall Judgement | Some concerns |

| **Study ID** | **Lachman 2020** |
| --- | --- |
| *Experimental* | parenting programme & agribusiness training |
| *Comparator* | usual care |
| Outcome | PTSD diagnosis |
| **Domain 1a:**  **Risk of bias arising from the randomization process** |  |
| 1a.1 | Y |
| 1a.2 | Y |
| Note for 1a.1&1a.2 | Quote: "Cluster randomisation was conducted at village level prior to baseline assessments, to reduce the likelihood of contamination between arms. An external researcher used concealed computer-generated codes to randomly allocate eight villages into three treatment arms and a control arm "; "The implementing partner notified the participating families of their allocation status after baseline data collection." |
| 1a.3 | NI |
| Note for 1a.3 | No useful information is reported to evaluate this element |
| 1a.0 Assessor's Judgement | Low |
| **Domain 1b: Risk of bias arising from the timing of identification or recruitment of participants** |  |
| 1b.1 | PN |
| Note for 1b.1 | randomization was conducted prior to the recruitment of participants |
| 1b.2 | PN |
| Note for 1b.2 | groups were comparable at baseline, see Table 1 |
| 1b.0 Assessor's Judgement | Low |
| **Domain 2: Risk of bias due to deviations from the intended interventions** |  |
| 2.1a | NI |
| Note for 2.1a | No information provided on whether participants knew that they were in a trial |
| 2.1b (2.1) | PY |
| 2.2 | PY |
| Note for 2.1b&2.2 | No information provided on whether participants knew that they were in a trial, due to the nature of the intervention they (as those delivering the intervention) were most likely aware of intervention allocation. |
| 2.3 | PN |
| Note for 2.3 | No evidence to suggest deviations from the protocol |
| 2.4 | NA |
| 2.5 | NA |
| 2.6 | Y |
| Note for 2.6 | Data was analysed on an intention-to-treat basis. |
| 2.7 | NA |
| 2.0 Assessor's Judgement | Low |
| 2.0 General Notes | Even if participants were most likely aware of allocation there was no evidence to suggest deviations from the trial context. Particiapnts were analyzed on an intention to treat basis. |
| **Domain 3: Missing outcome data** |  |
| 3.1a | PY |
| Note for 3.1a | all included clusters were analyzed |
| 3.1b | PY |
| Note for 3.1b | data were available for nearly all participants within the cluster.Quote:"Study retention was considerably higher than anticipated with 94.8% adults (n=235/248), 87.5% children (n=154/176) and 87.8% in their early childhood (n=122/139) assessments completed at post-treatment, with no differences between arms " |
| 3.2 | NA |
| 3.3 | NA |
| 3.4 | NA |
| 3.0 Assessor's judgement | Low |
| 3.0 General notes | Data was available for almost all participants, all clusters and participants have been analysed. |
| **Domain 4: Risk of bias in measurement of the outcome** |  |
| 4.1 | PN |
| Note for 4.1 | The outcome measure is widely established and validated for use across different contexts |
| 4.2 | PN |
| Note for 4.2 | No evidence to suggest differences in measurement between intervention and control. |
| 4.3a | NI |
| Note for 4.3a | No information provided on whether participants knew that they were in a trial |
| 4.3b | PY |
| Note for 4.3b | No information provided on whether participants knew that they were in a trial, participants (outcome assessors) were most likely aware of the group allocation due to the nature of the intervention. |
| 4.4 | PY |
| 4.5 | PN |
| Note for 4.4&4.5 | Knowledge of the assigned intervention could influence participant-reported outcomes, but there is no reason to believe that it did. |
| 4.0 Assessor's Judgement | Some concerns |
| 4.0 General note | There is no evidence to suggest that the outcome measure was inappropriate and that measurement could have differed between groups. No information is provided on whether participants were aware that a trial was taking place, but it is likely that they were aware of their assigned intervention. There is no reason to believe that this knowledge would have influenced outcome assessment. |
| **Domain 5:**  **Risk of bias in selection of the reported result** |  |
| 5.1 | PY |
| Note for 5.1 | No evidence to suggest otherwise, protocol and final article not show discrepancies. |
| 5.2 | PN |
| Note for 5.2 | No evidence to suggest outcome selection. Further outcomes than those mentioned in the protocol were reported. |
| 5.3 | PN |
| Note for 5.3 | No evidence to suggest analyses selection. All analyses mentioned in the methods and results were reported. |
| 5.0 Assessor's Judgement | Low |
| 5.0 General note | No evidence to suggest analysis or outcome selection. All outcomes and analyses mentioned in the methods and results were reported. |
| **Overall Risk of Bias** |  |
| Assessor's overall Judgement | Some concerns |

**Appendix H** – **Analyses 1 – Combined interventions compared to control group in preventing mental conditions among adults at post-intervention**

**Diagnosis of depression**

**Diagnosis of PTSD**

**Depressive symptoms**

**Anxiety symptoms**

**PTSD symptoms**

**Appendix I** – **Analyses 2 – Combined interventions compared to control group in preventing mental conditions among children and adolescents at post-intervention**

**Diagnosis of depression**

**Diagnosis of PTSD**

**Depressive symptoms**

**Anxiety symptoms**

**PTSD symptoms**

**Appendix J** – **Analyses 3 – Combined interventions compared to control group in preventing mental conditions among adults at 1 to 6 months post-intervention**

**Depression diagnosis**

**Anxiety symptoms**

**PTSD symptoms**

**Appendix K** – **Analyses 4 – Combined interventions compared to control group in preventing mental conditions among children and adolescents at 1 to 6 months post-intervention**

**Depressive symptoms**

**Anxiety symptoms**

**PTSD symptoms**

**Appendix L** – **Analyses 5 – Combined interventions compared to control group in preventing mental conditions among adults at 7 to 24 months post-intervention**

**Depressive symptoms**

**PTSD symptoms**

**Appendix M** – **Analyses 6 – Combined interventions compared to control group in preventing mental conditions among children and adolescents at 7 to 24 months post-intervention**

**Depression diagnosis**

**Depressive symptoms**

**Anxiety symptoms**

**PTSD symptoms**

**Appendix N – Subgroup analysis – Combined interventions compared to control group in preventing mental conditions among adults at post-intervention**

**Country income**

**Gender of participants**

**Humanitarian setting**

**Social determinants domain**

**Type of control groups**

**Type of prevention category**

**Type of providers**

**Type of study design**

**Appendix O – Sensitive analysis – Combined interventions compared to control group in preventing mental conditions among adults at post-intervention**

**Appendix P - Dichotomization of depressive and PTSD symptoms at post intervention (depression, PTSD)**

**Depression**

**
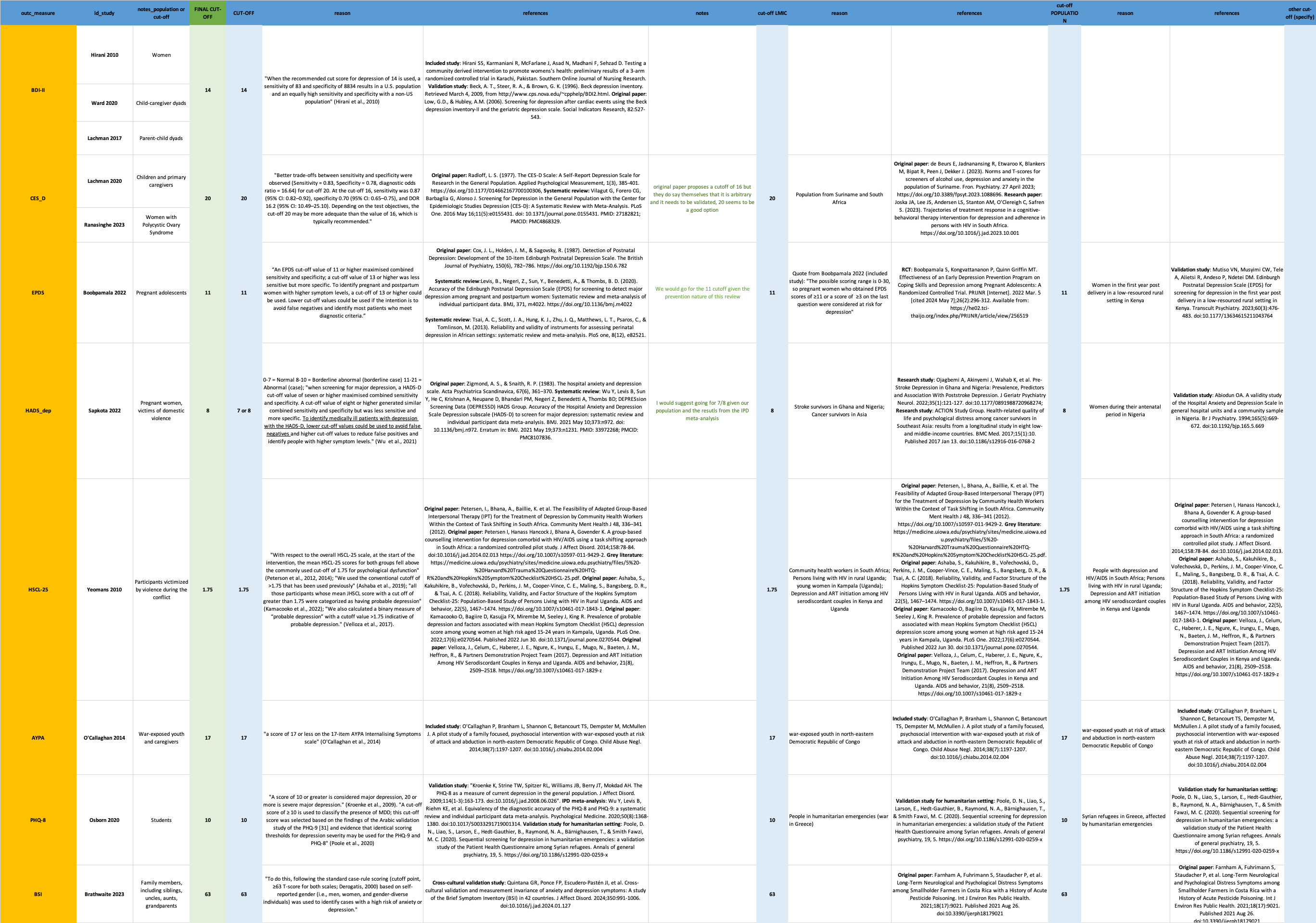
**

**
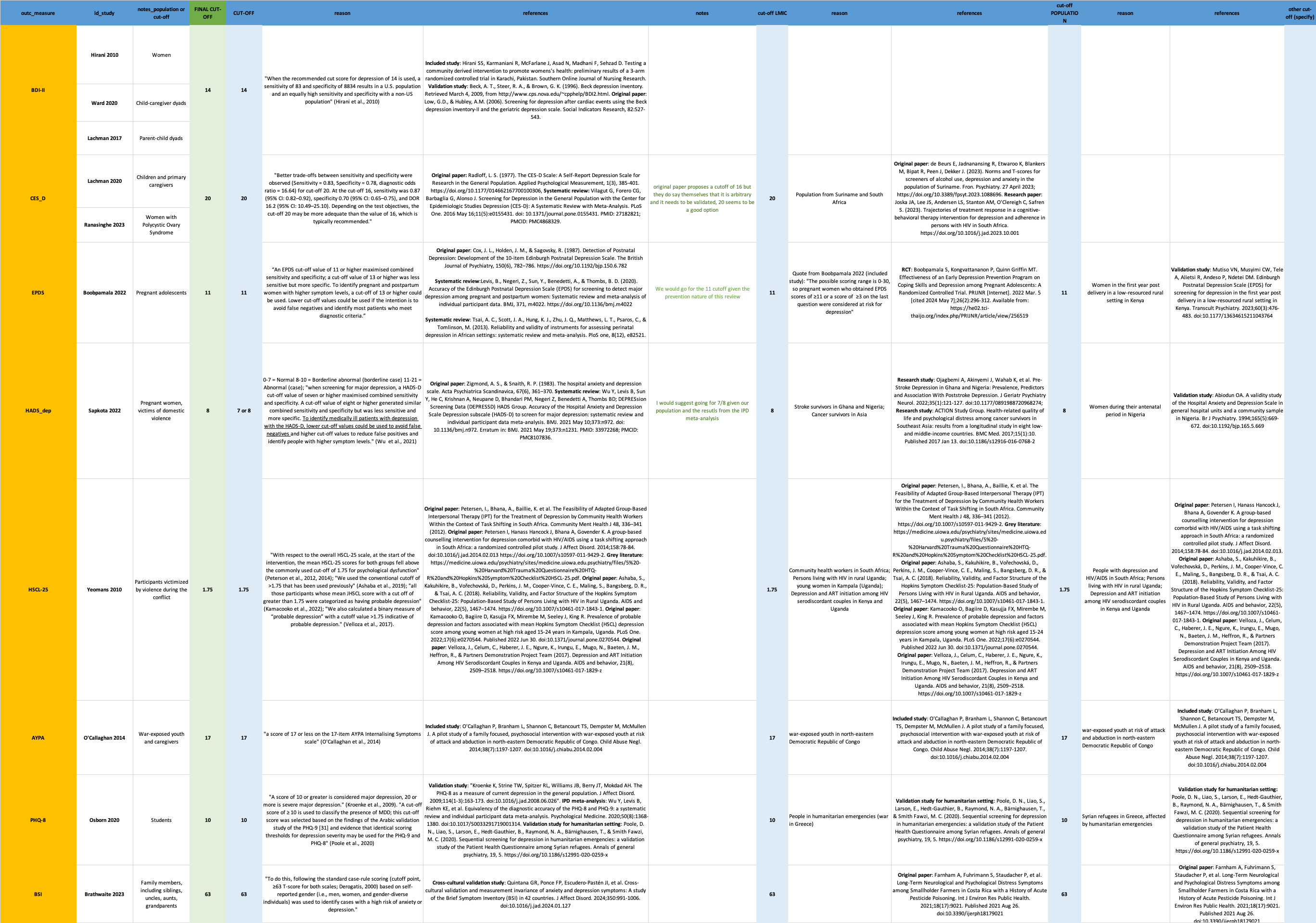
**

**PTSD**

**
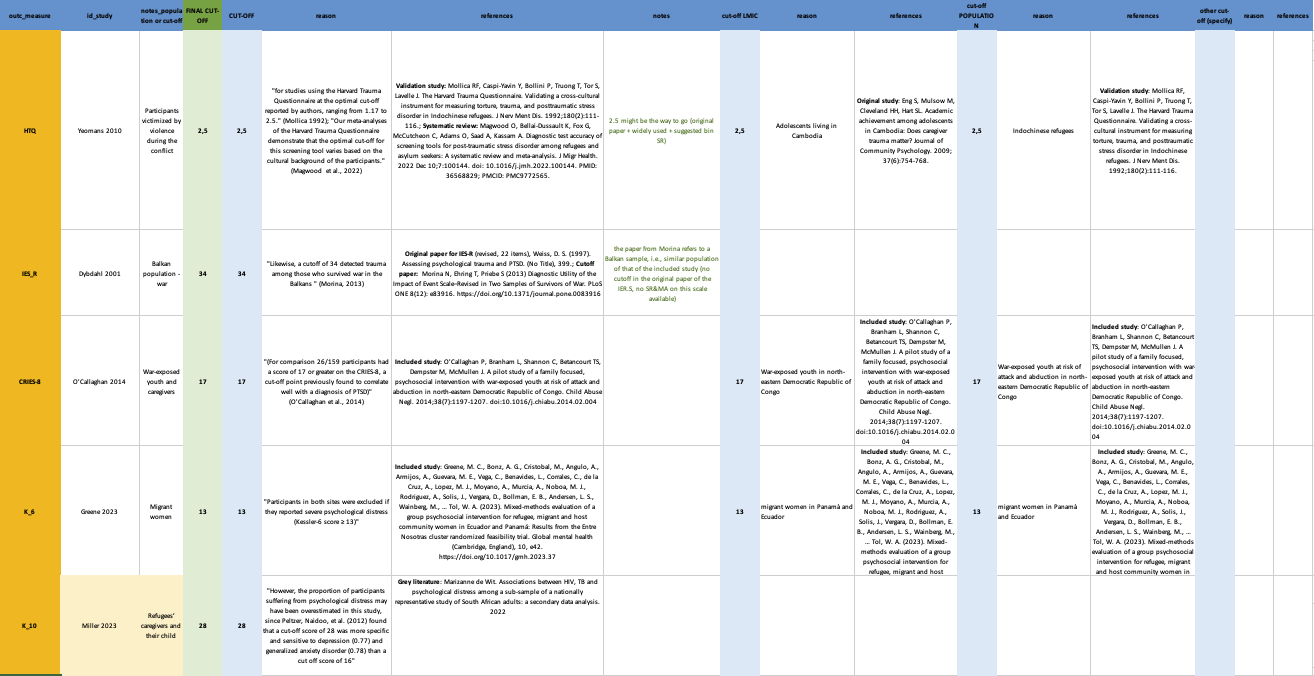
**

**
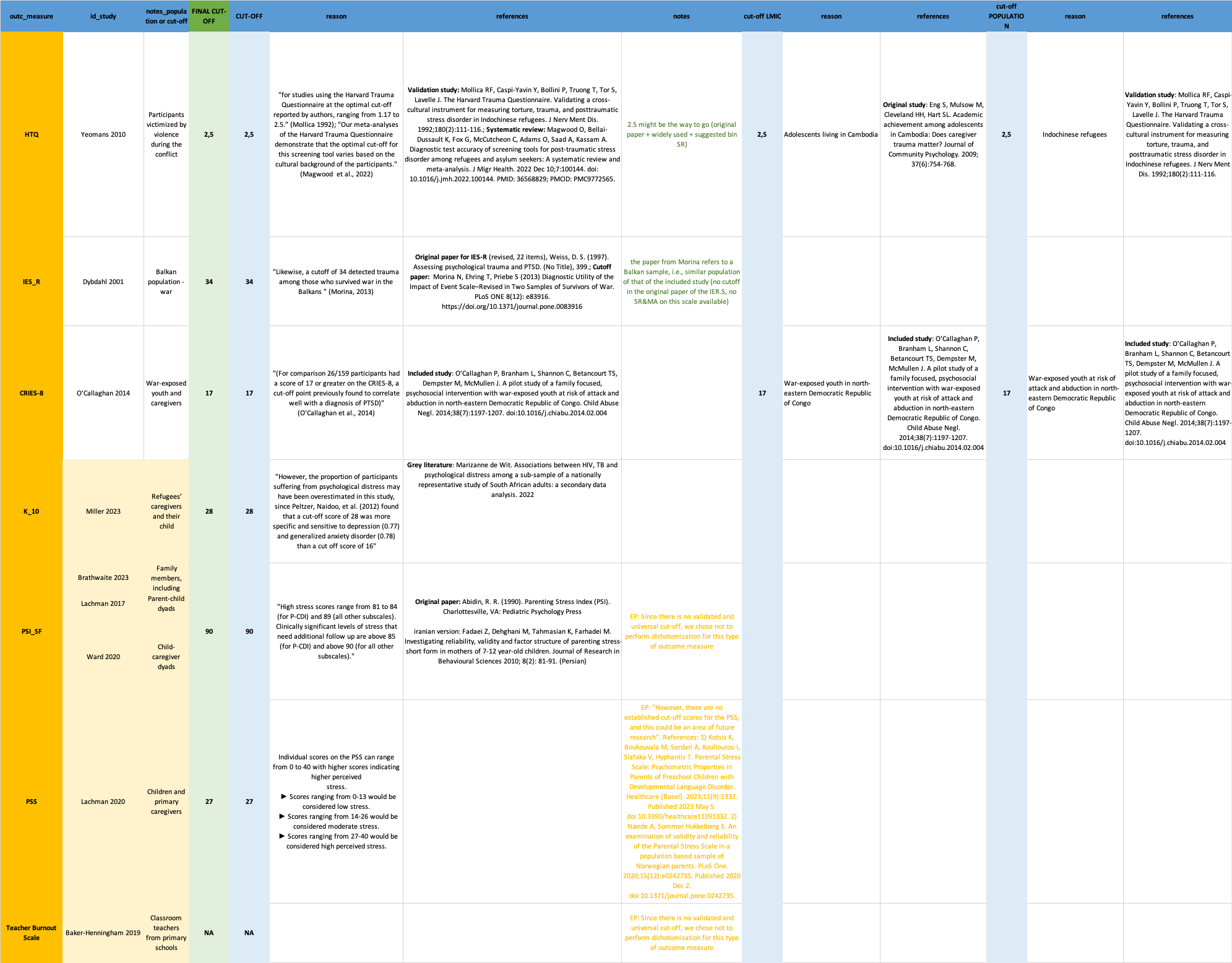
**

**Appendix Q – Narrative economic analysis**

| Author, year | Country | Type of economic analysis | Study population | Interventions | Intervention-specific costs and cost-effectiveness | Resources* |
| --- | --- | --- | --- | --- | --- | --- |
| Osborn 2020 | Kenya | Cost analysis | Adolescents (both male and females, 13 to 18 years of age) | Shamiri-Digital Wellness vs ac- tive control | Cost: USD 3.57 per student to deliver Shamiri-Digital | Health service cost: health service costs included equip- ment (computers, desks, chairs) with an hourly cost of USD 0.97, totalling USD 104.65 for the 9 months of the intervention |
| - | - | - | - | Self-help digi- tal-based inter- vention | Cost-effectiveness: depending on the definition of clinically meaningful improve- ment, 7.1 to 9.7 students needed to receive the intervention for 1 student to experience a clinically meaningful improvement, which translated to a cost of USD 25.35 to USD 34.62 per student | Patient cost: none reported |
| Bathia 2023 | India | Cost analysis | Family members reporting that the relative had been drinking problematically | Supporting Addiction Affected Families Effectively (SAFE) | Cost: 30 yuva saathi received INR 5000 monthly; four facilitators and two promoters got INR 15000 monthly each. INR 275,596 allocated for training, INR 223,637 for meetings and workshops, and INR 191,638 for activities. Annual cost: INR 4,966,270 | Health service and patient cost: none reported |

***Notes:*** USD: US dollar, INR: Indian Rupee

* i.e., costs to health services other than intervention costs; patient/society costs and productivity.

**Appendix R** – **GRADE Table**

**Question: Combined intervention compared to control condition for the prevention of common mental health conditions in low- and middle- income countries**

| **Certainty assessment** | | | | | | | **№ of patients** | | **Effect** | | **Certainty** | **Importance** |
| --- | --- | --- | --- | --- | --- | --- | --- | --- | --- | --- | --- | --- |
| **№ of studies** | **Study design** | **Risk of bias** | **Inconsistency** | **Indirectness** | **Imprecision** | **Other considerations** | **combined intervention** | **control condition** | **Relative (95% CI)** | **Absolute (95% CI)** |  |  |
| **Diagnosis at post-intervention (adults)** | | | | | | | | | | | | |
| 11 | randomised trials | serious^a^ | not serious | serious^b^ | not serious | none | 384/981 (39.1%) | 515/1016 (50.7%) | **RR 0.82** (0.73 to 0.93) | **91 fewer per 1,000** (from 137 fewer to 35 fewer) | ⨁⨁◯◯ Low | CRITICAL |
| **Diagnosis at post-intervention (children and adolescents)** | | | | | | | | | | | | |
| 4 | randomised trials | serious^a^ | not serious | serious^b^ | not serious | none | 35/194 (18.0%) | 55/193 (28.5%) | **RR 0.70** (0.49 to 0.99) | **85 fewer per 1,000** (from 145 fewer to 3 fewer) | ⨁⨁◯◯ Low | CRITICAL |

**CI:** confidence interval; **RR:** risk ratio

#### **Explanations**

a. Downgraded 1 level owing to study limitations (over 30% of RCTs had some concerns for some domains)

b. Downgraded 1 level owing to indirectness (outcome measures as proxy of diagnosis of mental disorder)

**Appendix S** – **Publication bias**

Funnel plot of combined intervention compared to control condition in preventing common mental health conditions at post intervention

**Appendix T – List of key social determinants of mental health based on Lund's theoretical framework**

| **Social determinants domains** | **Distal factors** | **Proximal factors** |
| --- | --- | --- |
| Demographic | - Community diversity - Population diversity - Longevity survival | - Age - Ethnicity - Gender |
| Economic | - Economic recessions - Economic inequality - Macroeconomic policy | - Income, Debt, Assets, Financial strain - Relative Deprivation - Unemployment - Food security |
| Neighbourhood | - Infrastructure - Neighbourhood deprivation - Built environment - Setting | - Safety and security - Housing structure - Overcrowding - Recreation |
| Environmental events | - Natural disasters, Industrial disasters - War or conflict - Climate change - Forced migration | - Trauma - Distress |
| Social and cultural | - Community social capital - Social stability - Cultural | - Individual social capital - Social participation - Social support - Education |

Reference:

Lund C, Brooke-Sumner C, Baingana F, Baron EC, Breuer E, Chandra P, et al. Social determinants of mental disorders and the Sustainable Development Goals: a systematic review of reviews. The Lancet Psychiatry. 2018 Apr;5(4):357–69.

**Appendix U – Characteristics of included studies by country, population, social determinants’ domain, interventions’ components, and outcomes.**

| **Study** | **Cluster**  **(Yes/No)** | **Sample size**  **(N randomized)** | **Country (Income)** | **Population** | **Psychological component** | **Social determinants component: demographic domain** | **Control condition** | **Outcomes** |
| --- | --- | --- | --- | --- | --- | --- | --- | --- |
| Lachman  2017^44^ | No | 68 | South Africa  (UM) | Child-caregiver dyads | Group family’s program on emotional communication | Families program on keeping children safe in communities characterized by violence | Usual care | Distress (parenting stress index-short form), depressive symptoms (BDI-II^1^) |
| Skar 2021^45^ | No | 119 | Colombia  (UM) | Parents | Activities for good caregiver-child interaction: emotions, communication, and regulation | Violence curriculum on safeguarding children and prevention planning | Usual care | Depression (SSQ^2^ > 8) |
| Ward 2020^46^ | No | 296 | South Africa  (UM) | Child-caregiver dyads | The first half of the program focused on positive relationships and reinforcement of desirable behaviors | The second half of the parenting program taught nonviolent discipline strategies | Usual care | Distress (PSI-SF^3^), Depressive symptoms (BDI-II^1^) |
| Brathwaite 2023^68^ | Yes | 437 | Uganda  (L) | Family members, including siblings, uncles, aunts, grandparents | Multiple family group targeting parenting stress, mental health and support systems for families | Model targeting rules, respectful communication, discipline practices, family connectedness, support | Usual care | Depressive symptoms (BSI^30^) and distress (PSI-SF^3^) |
| Comrie-Thomson 2022^47^ | Yes | 890 | Zimbabwe  (LM) | Couples | Participatory learning and action (PLA) cycles among women grounded in problem-solving therapy. | Men participated in group discussions, exploring gender-related challenges and men’s contributions to child health. | Usual care | Depression (EPDS > 12) and depressive symptoms (EPDS^4^) |
| Miller 2023^48^ | No | 480 | Lebanon  (UM) | Refugee caregivers and their child | Caregiver Support Intervention focus on caregiver wellbeing through stress management and mindfulness techniques | Program focuses on increasing positive parent-child interactions and decreasing the use of harsh parenting practices | Usual care | Distress (K10^5^) |
| Sapkota 2022^49^ | No | 143 | Nepal  (LM) | Pregnant women exposed to domestic violence | Single-session counselling based on a problem-solving approach and common stress management techniques | Educational intervention aimed to inform on domestic violence, its common types, and potential mental health impacts | Usual care | Anxiety and depressive symptoms (HADS^6^) |
| Donenberg 2021^50^ | No | 60 | South Africa  (UM) | Adolescent girls and their female caregivers | The curriculum emphasizes the impact of mental distress on sexual and reproductive health and teaches strategies to manage emotions | The curriculum is designed to strengthen family relationships, improve caregiver monitoring, and encourage gender empowerment | Usual care | Depression (PHQ-9^7^ > 10), Anxiety (GAD^8^), depressive (PHQ-9) and PTSD (PC-PTSD-5^9^) symptoms |
| Friedberg 2023*^51^ | Yes | 7711 | Kenya  (LM) | Adolescents living in informal settlements | The program involved empowerment, gender norms, and techniques for achieving goals | Program teaches skills like verbal confrontations and physical self-defense to reduce sexual assault | Usual care | Anxiety (BAI^10^), depressive (BDI^11^) and PTSD symptoms (CPSS^12^) |
| **Study** |  |  | **Country** | **Population** | **Psychological component** | **Social determinants component: economic domain** |  | **Outcomes** |
| Lachman 2020^52^ | Yes | 182 | Tanzania  (LM) | Child-caregiver dyads | Skillful program on parenting skills and child protection | Agribusiness training program, including credit for farm inputs, advice to improve farming and marketing techniques | Usual care | Depressive symptoms (CESD^13^) and distress (PSS^14^) |
| Hirani 2010^53^ | Yes | 17 | Pakistan  (L) | Women | Group counselling focused on stress-anger management, effective communication, active listening and supporting problem-solving | Economic skill-building intervention based on community, including skills for employment attainment and retention | Usual care | Depressive symptoms (BDI-II^1^) |
| Byansi 2022^54^ | Yes | 789 | Uganda  (L) | Adolescents’ girls and parents | Multiple family groups aim to strengthen family communication and reduce stigma by providing a safe space | Training on principles of financial management with funds for girls’ education or family-based income-generating activities | Usual care | Depressive symptoms (BDI^11^) |
| Massarwi 2022^74^ | Yes | 552 | South Africa  (UM) | Parents and primary caregivers of adolescents | Program based on social learning theory to improve family cohesion by using problem-solving techniques | Economic components designed to improve families’ financial conditions | Usual care | Depressive symptoms (CESD^13^) and distress (PSS^14^) |
| **Study** |  |  | **Country** | **Population** | **Psychological component** | **Social determinants component: neighbourhood domain** |  | **Outcomes** |
| Sherman 2009^55^ | No | 983 | Thailand  (LM) | Young methamphetamine users | Individual curriculum based on cognitive behavioral techniques, focusing on causes/consequences and stress of drug use | Educator network intervention to reduce drug use and risk with peers (e.g., drug users and/or sexual partners) | Usual care | Depressive symptoms (CESD^13^) |
| Shinde 2018^56^ | Yes | 8511 | India  (LM) | Students attending secondary schools | Individual student counselling activities based on problem-solving | Whole school activities addressing hygiene, bullying, substance use, sexual health, gender-based violence, rights | Usual care | Depression (PHQ-9^7^) |
| Jiang 2022^57^ | Yes | 392 | China  (UM) | Children with HIV-positive parent | Program based on the resilience framework enhancing coping, emotional regulations, and positive thinking | Intervention on HIV risk factors (e.g., poverty, stigma, and violence), positive parenting skills and community-based component | Usual care | Depressive (CES-CS^15^) and anxiety symptoms (CRS^16^) |
| Greene 2023^73^ | Yes | 225 | Ecuador  (UM) | Migrant women | Stress management component based on WHO SH+ intervention to address emotional distress and sadness | Intervention designed to strengthen community connectedness and collective action to promote migrant’s safety | Usual care | Distress (K6)^17^ |
| **Study** |  |  | **Country** | **Population** | **Psychological component** | **Social determinants component: environmental events domain** |  | **Outcomes** |
| Dybdahl 2001^58^ | No | 87 | Bosnia and Herzegovina  (LM) | War-exposed mothers and children | Psychoeducation on trauma, with an emphasis on strengthening participants’ coping strategies | Sensitize caregivers by creating a warm human environment | Usual care | Distress (IES^18^) |
| O’Callaghan 2014^59^ | No | 159 | Democratic Republic of Congo  (L) | War-exposed youth and caregivers | Psychoeducation on trauma and related stigma, relaxation techniques and effective parenting | Family intervention on the main problems of the community (e.g. war, hunger), how to solve them and the contribution of youth | Waiting list | Distress (CRIES^19^), depressive symptoms (AYPA^20^) |
| James 2019*^60^ | No | 480 | Haiti  (L) | Disaster-exposed participants | Mental health component teaching coping strategies for disaster-related stress (e.g. grounding, mindfulness) | Disaster preparedness and mental health peer support practice component | Waiting list | Depressive (ZLDSI^21^), PTSD (MPSS^22^) and anxiety symptoms (BAI^10^) |
| Dhital 2019^61^ | Yes | 1220 | Nepal  (LM) | Adolescents | Teacher-mediated school-based intervention that provides psychosocial support, coping, a sense of security, self-esteem, and hope | Psychosocial support in education in a post-earthquake setting, focusing on coping with crisis and recovering from trauma | Usual care | Depressive (Depression self-rating scale^23^) and PTSD symptoms (CPSS^12^) |
| Panter-Brick 2018*^62^ | No | 817 | Jordan  (LM) | Crisis-exposed adolescents | Advancing Adolescents program focuses on safe emotional spaces, managing stressors, healthy relationship | Psychosocial intervention based on safety, social support and group-based activities | Waiting list | PTSD symptoms (CRIES^19^) |
| Yeomans 2010^71^ | No | 83 | Burundi  (L) | Participants exposed to violence during armed conflict | Group-based intervention based on fostering interpersonal exchange, PTSD psychoeducation, and games to explore themes of trauma, loss, anger | Workshops focused on communication about trust, safety and security, discussing how the war has affected participants | Waiting list | Depressive (HSCL-25^24^) and PTSD symptoms (HTQ^25^) |
| **Study** |  |  | **Country** | **Population** | **Psychological component** | **Social determinant component: social and cultural domain** |  | **Outcomes** |
| Baker-Henningham 2019^63^ | Yes | 55 | Jamaica  (UM) | Classroom teachers from primary schools | Classroom Toolbox including positive behaviour, promoting children’s social-emotional competence | Violence prevention programme including teaching rules and routines, promoting children’s active participation | Usual care | Distress (Teacher Burnout Scale^26^) and depressive symptoms (CESD^13^) |
| Fabbri 2021^64^ | Yes | 4228 | Tanzania  (LM) | Students and teachers | Behavioral self-guided intervention focused on empathy, self-regulation techniques, wellbeing | Intervention generated social support through the group setting so that teachers could count on peers for support | Waiting list | Depressive symptoms (MFQ^27^) |
| Osborn 2020^65^ | No | 103 | Kenya  (LM) | Students | The intervention originated from psychotherapy is based on growth mindset, gratitude, and mindfulness | Study skills program based on learned skills designed to improve abilities to study and, ultimately academic performance | Usual care | Depressive (PHQ-8^28^) and anxiety symptoms (GAD^8^) |
| Langer 1996^66^ | No | 2235 | Brazil  (UM) | Pregnant women | Psychosocial support for reducing anxiety and stress, and the provision of emotional support | Psychosocial support, including reinforcement of social support network through participants’ “support person” | Usual care | Anxiety symptoms (STAI^29^) |
| Bhatia 2023^67^ | Yes | 3590 | India  (LM) | Family members of relatives who drink problematically | Sessions based on the identification of triggers, knowledge of drug use and coping responses | Sessions based on creating a social network diagram, explore new sources of support, improve family communication | Usual care | Distress (FMI^30^) |
| Ranasinghe 2022^72^ | No | 44 | Sri Lanka  (LM) | Women with Polycystic Ovary Syndrome | Peer-led support groups included understanding psychosocial issues, learning coping, problem-solving skills, negative emotions | Sessions included defining group dynamics, the concept of a support group, sharing thoughts on being part of the group, and identifying ways to maintain ongoing support | No treatment | Depressive symptoms (CESD^13^) |
| Metzler 2023^69^ | No | 849 | Uganda  (L) | South Sudanese refugee children | Toolkit intervention following emotional learning, well-being and coping “feeling good” | The intervention followed themes such as “building community”, “social support: my friends and family” | Waiting list | PTSD symptoms (CPSS^12^) |
| Boobpamala 2022^70^ | No | 80 | Thailand  (UM) | Pregnant adolescents | Early Depression Prevention Program based on problem-solving, covering physical and mental changes, self-care, and depressive symptoms | Intervention based on the social support theory. A family member or friend assists women during pregnancy | Usual care | Depressive symptoms (Antenatal Depression Scale^32^) |

**Notes**

*data were not included in the meta-analysis because they were not provided in the right format or were not available even after attempted author contact.

UM: upper-middle income, LM: lower-middle income, L: low income
